# LHBs can elevate the expression of MDR1 through HIF-1α in patients with CHB infection: a comparative proteomic study

**DOI:** 10.18632/oncotarget.13941

**Published:** 2016-12-15

**Authors:** Shiying Li, Yixuan Yang, Xiangchun Ding, Min Yang, Sha She, Hong Peng, Xiaoming Xu, Xiaoping Ran, Sanglin Li, Peng Hu, Huaidong Hu, Dazhi Zhang, Hong Ren

**Affiliations:** ^1^ Key Laboratory of Molecular Biology for Infectious Diseases (Ministry of Education), Institute for Viral Hepatitis, Department of Infectious Diseases, The Second Affiliated Hospital, Chongqing Medical University, Chongqing, PR China; ^2^ Department of Infectious Diseases, General Hospital of Ningxia Medical University, Yinchuan, Ningxia, PR China

**Keywords:** LHBs, MDR1, HIF-1α, iTRAQ, proteomics

## Abstract

**Background and Aims:**

Hepatitis B virus (HBV) infection is a major risk factor for liver cirrhosis and hepatocellular carcinoma (HCC). To gain a better understanding of the pathogenesis of HBV infection, this study aimed to investigate the differentially expressed proteins (DEPs) in liver tissues from patients with chronic hepatitis B (CHB) infection.

**Results:**

Seventy-one DEPs were identified. Overexpression of multi-drug resistance protein 1 (MDR1) was validated by RT-qPCR and western blot analyses. Moreover, its expression was increased at both the mRNA and protein levels in response to overexpression of HBV large surface protein (LHBs). Furthermore, screening of transcription factors suggested the possible involvement of hypoxia-inducible factor 1α (HIF-1α) in the interaction between LHBs and MDR1. The function of HIF-1α in the MDR1 activation was confirmed by EMSA and reporter gene analyses.

**Materials And Methods:**

Liver samples from CHB patients and controls without HBV infection were collected and subjected to isobaric tags for relative and absolute quantitation (iTRAQ) and mass spectrometric analysis.

**Conclusions:**

These results imply that LHBs, in association with HIF-1α, induces MDR1 overexpression, which may contribute to the pathogenic changes in CHB infection.

## INTRODUCTION

Hepatitis B virus (HBV) infection is one of the most common infectious diseases worldwide, and an estimated 788,000 deaths result from HBV-related end-stage liver disease (such as cirrhosis, liver failure, and hepatocellular carcinoma (HCC)) each year [1, 2], creating a significant socioeconomic burden to society. Effective antiviral therapy may slow down the progression of liver injury and prevent dismal complications, but the currently available drugs [3] do not produce a sustained viral response in all treated patients. In addition, significant side effects of interferon [4] and the development of drug resistance to nucleoside or nucleotide analogues limit the long-term treatment course that is required for sustained suppression of HBV replication [5]. Clearly, a better understanding of the pathogenesis of HBV infection is of high priority in order to facilitate an improved antiviral strategy.

Recently, approaches employed by comparative proteomic analysis hold promise for performing large-scale studies of protein profiles under various disease conditions. To identify the proteomic profiles of HBV infection, samples from HepG2.2.15 and HepAD38 cultured cell lines [6, 7], HBV transgenic mice [8], and blood from HBV-infected patients [9] have been analyzed. While these results provide important insights into the interaction between HBV and cellular proteins, they do not directly reflect the complexities of the interactions between HBV and HBV-infected hosts. In this regard, liver specimens obtained from patients with chronic hepatitis B (CHB) infection offer a unique opportunity to investigate the cellular molecular mechanisms involved in the pathogenesis of HBV infection. In this study, we utilized isobaric tags for relative and absolute quantitation (iTRAQ) coupled with liquid chromatography and two-dimensional tandem mass spectrometry (LC-MS/MS) analysis, an ultrasensitive and high-throughput proteomic technology [10], to quantify differentially expressed proteins (DEPs) unique to CHB infection.

## RESULTS

### Basic characteristics of the participants

A total of 128 individuals were included in this study, including 102 patients with CHB and 26 controls without HBV infection. As shown in Supplementary Table S1, the age of patients with CHB was 37 ± 11.4 years old, and 73.1% of them were male. In the control group, the mean age was 29 ± 14.1 years old, and 70.6% were male. Fifty-six CHB patients were HBeAg-positive and 46 were HBeAg-negative; the median HBV DNA level for all patients with CHB was 5.23 log copies/mL (range, 3.12 to 8.90 log copies/mL).

### iTRAQ identification and quantification of DEPs

**Figure 1:**
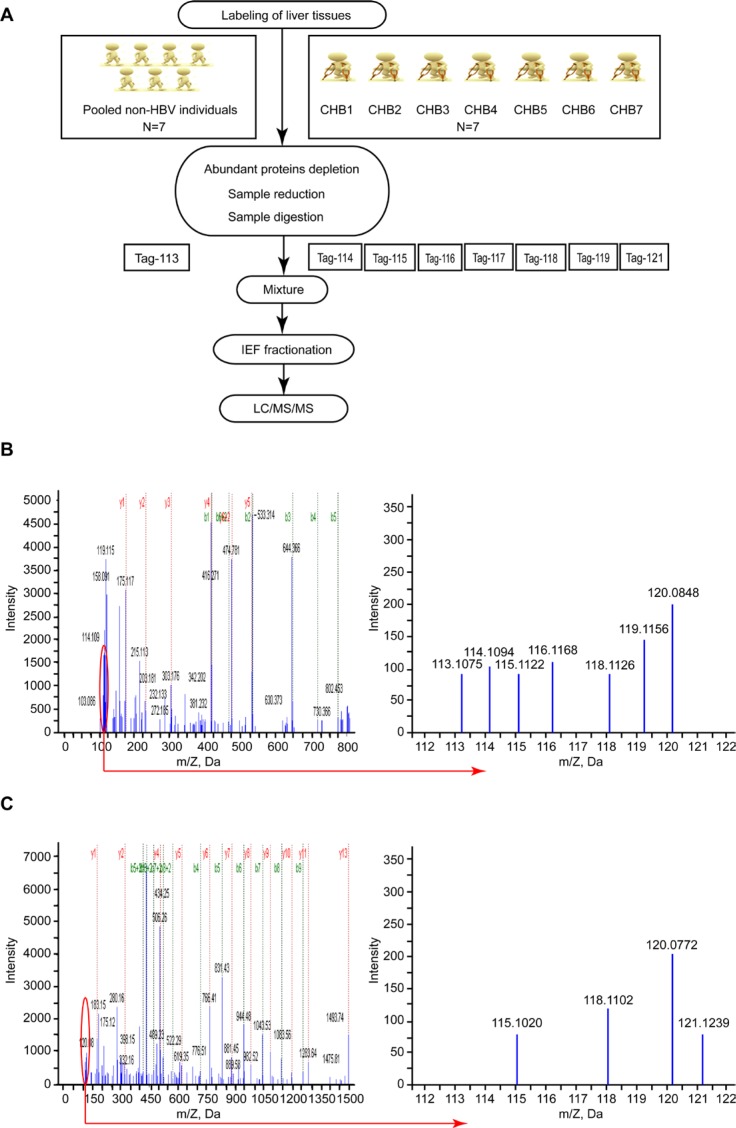
iTRAQ-based proteomic analysis for identification of DEPs in the liver tissues of patients with CHB infection (**A**) Flowchart of the iTRAQ proteomics procedure. The 8-plex iTRAQ approach was carried out twice: iTRAQ 1 was performed with HBeAg-positive CHB liver tissues, and iTRAQ 2 was performed with HBeAg-negative CHB liver tissues. (**B**) A representative MS/MS spectrum showing peptide signatures for MDR1 (peptide sequence: EIIGVVSQEPVLFATTIAENIR) in iTRAQ 1. (**C**) A representative MS/MS spectrum showing peptide signatures for MDR1 (peptide sequence: EIIGVVSQEPVLFATTIAENIR) in iTRAQ 2. The ratios of iTRAQ tags indicate the relative abundance of the MDR1 protein in liver tissues of CHB compared to samples of non-HBV individuals. Pooled liver samples from seven non-HBV individuals were labeled with iTRAQ 113 tag; CHB 1 was labeled with iTRAQ 114 tag; CHB 2 was labeled with iTRAQ 115 tag; CHB 3 was labeled with iTRAQ 116 tag; CHB 4 was labeled with iTRAQ 117 tag; CHB 5 was labeled with iTRAQ 118 tag; CHB 6 was labeled with iTRAQ 119 tag; and CHB 7 was labeled with iTRAQ 121 tag.

**Figure 2:**
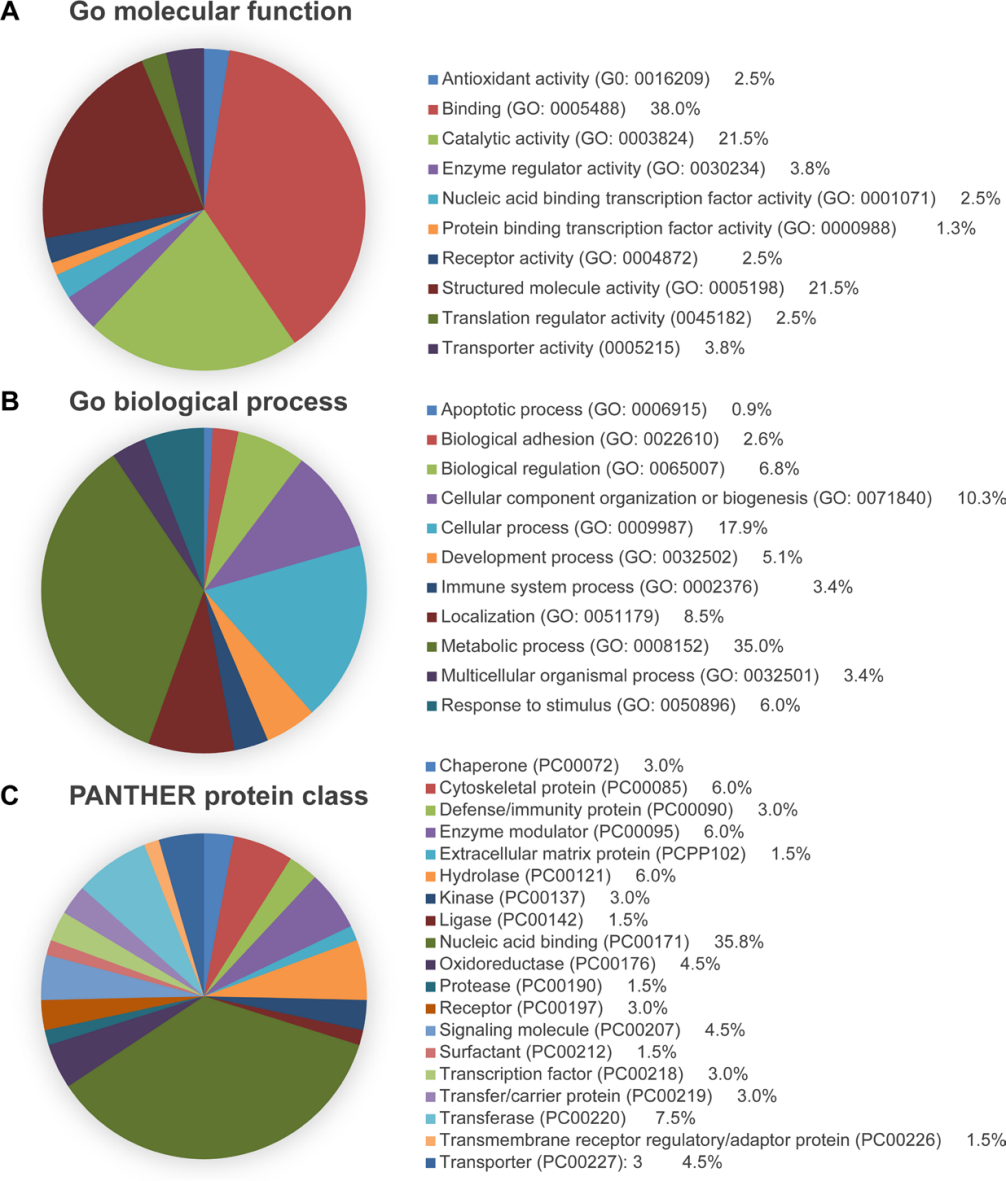
PANTHER analysis of the DEPs identified through iTRAQ proteomics Proteins were categorized by (**A**) molecular function, (**B**) biological process, and (**C**) protein class.

To identify the DEPs in the liver samples of patients with CHB, double 8-plex iTRAQ quantifications were performed to eliminate the effect of patients’ HBeAg status, each of which contains liver tissue samples from seven randomly selected patients with CHB (iTRAQ 1 was performed with liver tissues that were HBeAg-positive CHB, iTRAQ 2 was with liver tissues that were HBeAg-negative CHB) and seven non-HBV individuals (Figure 1A). Figure 1B and 1C show the representative mass spectrometric peaks of MDR1 in iTRAQ 1 and iTRAQ 2 quantification, respectively. The relative quantification for each of the seven CHB samples was referred to its corresponding pooled sample (113 tag) of seven controls, which were used as an internal reference, using ratios of 114:113, 115:113, 116:113, 117:113, 118:113, 119:113, and 121:113. This strategy resulted in the identification of 1486 unique proteins (Supplementary Table S2), among which 71 (Supplementary Table S3) displayed the same expression trend (elevated or reduced) in at least 12 CHB samples (53 upregulated and 18 downregulated proteins). The majority of these proteins were binding proteins, followed by catalytic and structured enzymes. They are known for many biological functions, such as metabolic processes, cellular processes, and localization (Figure 2).

### Validation of DEPs

**Figure 3:**
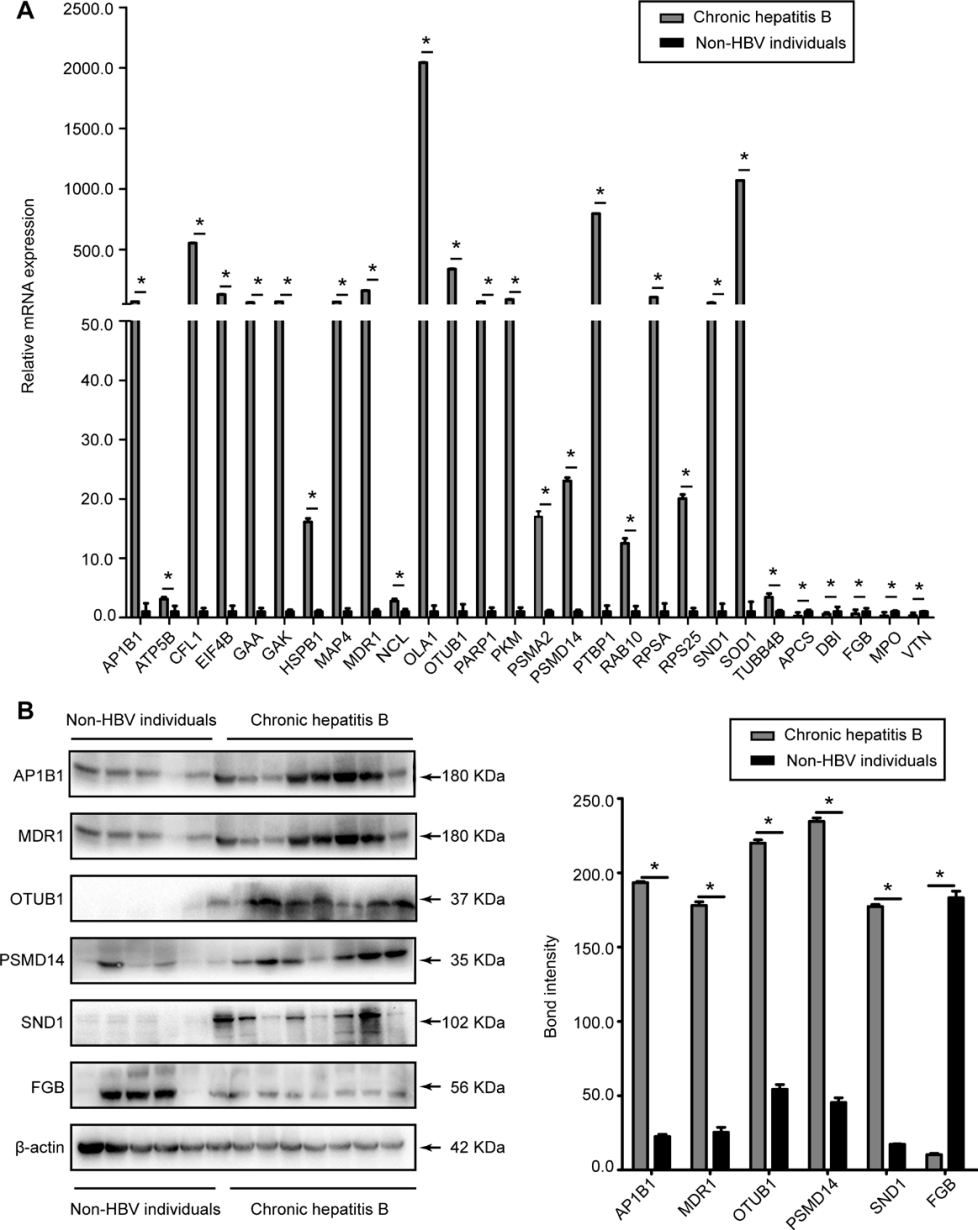
Evaluation of the DEPs in liver tissues of CHB patients and non-HBV individuals (**A**) The relative mRNA expression levels of APCS, AP1B1, ATP5B, CFL1, DBI, EIF4B, FGB, GAA, GAK, HSPB1, MAP4, MDR1, MPO, NCL, OLA1, OTUB1, PARP1, PKM, PSMA2, PSMD14, PTBP1, RAB10, RPSA, RPS25, SND1, SOD1, TUBB4B, and VTN were detected by RT-qPCR and normalized to GADPH. (**B**) Representative western blot analysis of AP1B1, FGB, MDR1, OTUB1, PSMD14, and SND1 expression levels in the liver tissues from CHB patients and non-HBV individuals. (Bars indicate SD, significant as compared to the control, *P < 0.05).

Through Uniprot, PANTHER, and Pubmed searches, 28 different proteins (APCS, AP1B1, ATP5B, CFL1, DBI, EIF4B, FGB, GAA, GAK, HSPB1, MAP4, MDR1, NCL, OLA1, OTUB1, PARP1, PKM, PSMA2, PSMD14, PTBP1, RAB10, RPSA, RPS25, SND1, SOD1,TUBB4B, MPO, and VTN) identified by MS analysis were chosen from 71 DEPs for validation. RT-qPCR was performed to detect their expression in liver tissues from ten CHB patients and seven controls. As shown in Figure 3A, the expression changes of their mRNAs were consistent with the MS analysis findings. In addition, six proteins (AP1B1, MDR1, OTUB1, PSMD14, SND1, and FGB) were chosen for further verification using western blot. This result showed that the expression of the first five proteins was significantly higher in CHB patients than that in controls, and the last one showed a marked downregulation in CHB patient samples (Figure 3B), which were also consistent with the MS analysis results (all *P* < 0.05).

### Differential MDR1 expression in hepatoma cell lines

**Figure 4:**
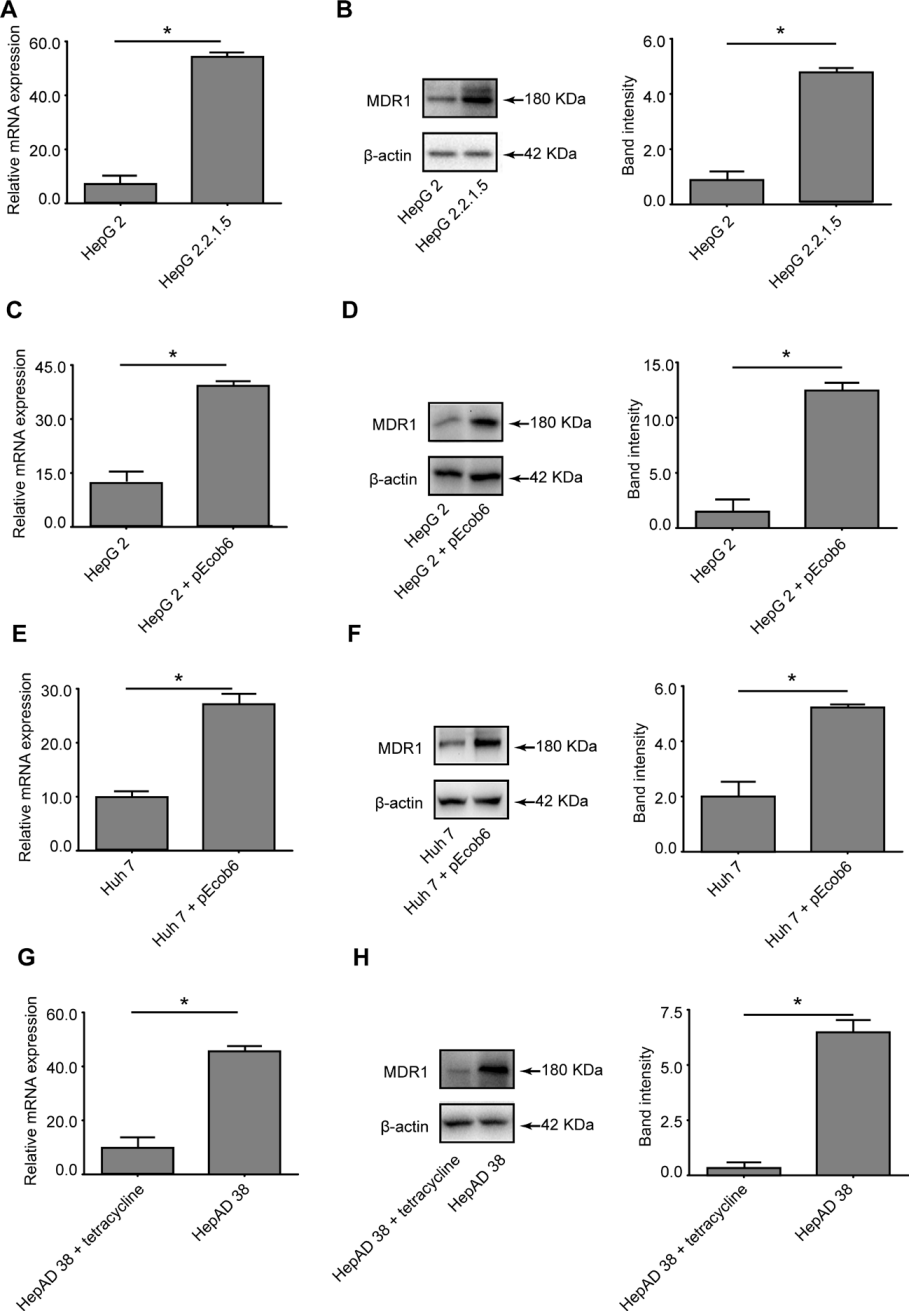
Evaluation of the differential expression of MDR1 in cell lines (**A**) RT-qPCR detection of the mRNA levels of MDR1 in HepG2 and HepG2.2.1.5 cells. (**B**) Western blot analysis of MDR1 in HepG2 and HepG2.2.15 cells. (**C**) RT-qPCR detection of the mRNA levels of MDR1 in HepG2 cells, with or without plasmid pEcob6 transfection. (**D**) Western blot analysis of MDR1 in HepG2 cells, with or without plasmid pEcob6 transfection. (**E**) RT-qPCR detection of the mRNA levels of MDR1 in Huh7 cells, with or without plasmid pEcob6 transfection. (**F**) Western blot analysis of MDR1 in Huh7 cells, with or without plasmid pEcob6 transfection. (**G**) RT-qPCR detection of the mRNA levels of MDR1 in HepAD 38 cells, with or without tetracycline. (**H**) Western blot analysis of MDR1 in HepAD 38 cells, with or without tetracycline. (Bars indicate SD, significant as compared to the control, *P < 0.05.)

To seek an additional indication for a possible link between MDR1 expression and HBV infection, MDR1 expression in several cell lines with or without HBV expression were analyzed by RT-qPCR and western blot. Figure 4A and 4B show that MDR1 expression was significantly elevated in HepG2.2.1.5 cells at both the mRNA and protein levels, compared to HepG2 cells. Parallel results were observed in HepG2 (Figure 4C and 4D) and Huh 7 (Figure 4E and 4F) cells, after transfecting with pEcob6 (1.0 μg) plasmid, which contained two copies of HBV DNA sequences in tandem. The mRNA and protein levels of MDR1 in HepAD 38 cells (Figure 4G and 4H), when the secretion of HBV was not blocked (cultured without tetracycline), were higher than when the secretion of HBV was blocked (with tetracycline) [11] (all *P* < 0.05).

### LHBs overexpression increased the expression level of MDR1

**Figure 5:**
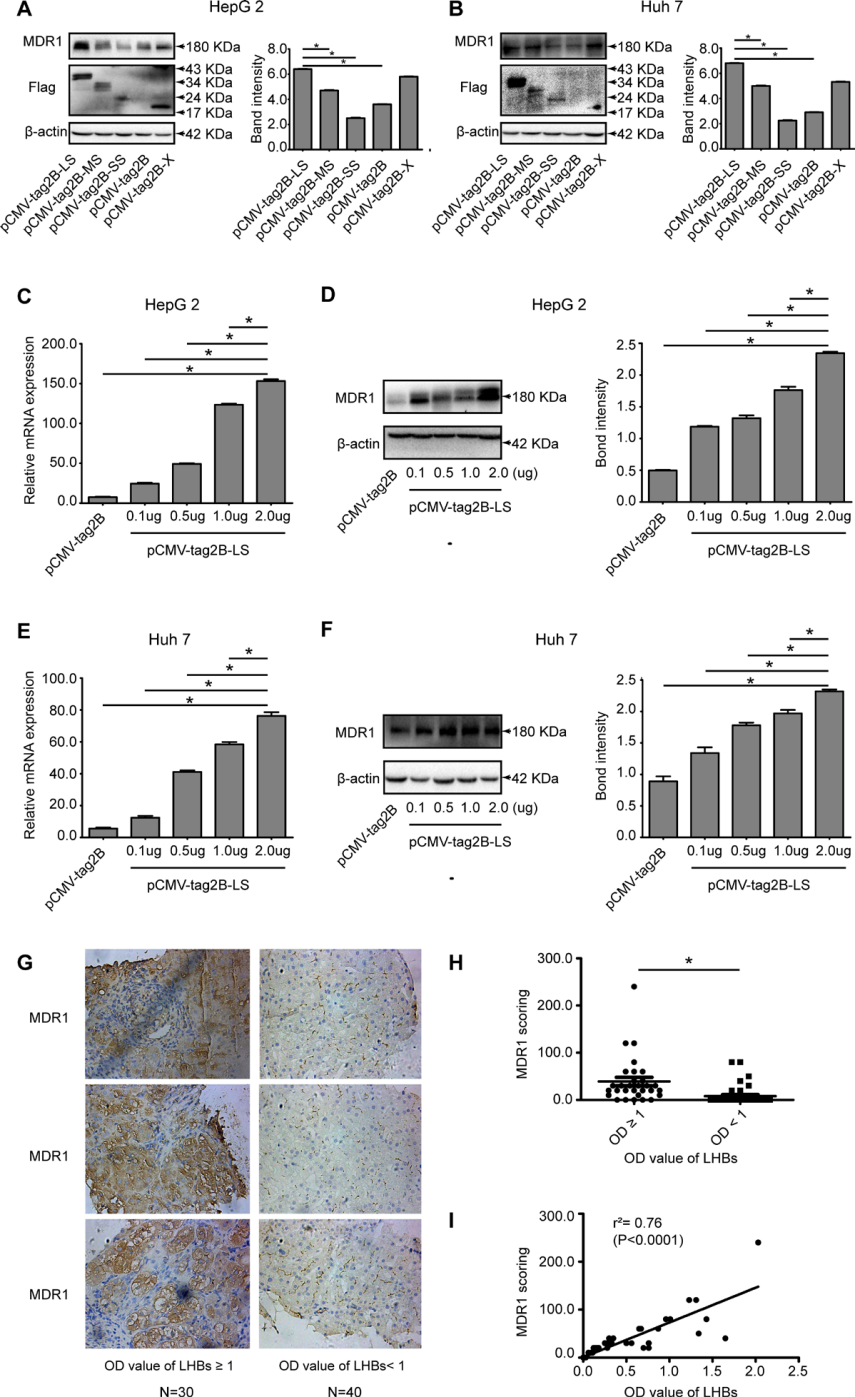
Evaluation of the relationship of LHBs and MDR1 in cells and liver tissues of CHB patients (**A**) Western blot analysis of MDR1 expression in pCMV-tag2B-LS, pCMV-tag2B-MS, pCMV-tag2B-SS, pCMV-tag2B-SS, or pCMV-tag2B plasmid-transfected HepG2 cells. (**B**) Western blot analysis of MDR1 expression in pCMV-tag2B-LS, pCMV-tag2B-MS, pCMV-tag2B-SS, pCMV-tag2B-SS, or pCMV-tag2B plasmid-transfected Huh7 cells. (**C**) RT-qPCR detection of the mRNA levels of MDR1 induced by different pCMV-tag2B-LS plasmid concentrations (0.1–2.0 μg) in HepG2 cells. (**D**) Western blot analysis of MDR1 expression induced by different pCMV-tag2B-LS plasmid concentrations (0.1–2.0 μg) in HepG2 cells. (**E**) RT-qPCR detection of the mRNA levels of MDR1 induced by different pCMV-tag2B-LS plasmid concentrations (0.1–2.0 μg) in Huh7 cells. (**F**) Western blot analysis of MDR1 expression induced by different pCMV-tag2B-LS plasmid concentrations (0.1–2.0 μg) in Huh7 cells. (**G**) Representative IHC images with hematoxylin counterstaining (400× magnification): the immmunohistochemical analysis of MDR1 expression in liver sections of 70 patients with CHB. (**H**) Corresponding scores of MDR1 from different LHBs level samples. (**I**) Linear regression of LHBs and MDR1 in IHC. (Bars indicate SD, *P < 0.05.)

Previous studies have reported that HBx protein transactivates the human MDR1 gene [11]. Moreover, LHBs also has been shown to be a strong transactivator [12, 13]. We wondered whether LHBs is involved in elevating MDR1 expression. Therefore, the pCMV-tag2B-LS, pCMV-tag2B-MS, pCMV-tag2B-SS, and pCMV-tag2B-X plasmids were respectively transfected into the HepG2 and Huh 7 cell lines, in parallel with control plasmid pCMV-tag2B transfection. As shown in Figure 5A and 5B, the anti-flag band showed the success of transfection and represented the corresponding overexpressed protein in each plasmid. MDR1 expression was remarkably increased in LHBs- and X protein-overexpressing cells, compared to the MHBs- and SHBs-overexpressing cells and the negative control (all *P* < 0.05). However, its levels in X protein-overexpressing cells seemed to be lower than that with LHBs-overexpression. In addition, this induction appeared to be LHBs level-dependent at both the mRNA (Figure 5C and 5E) and protein (Figure 5D and 5F) levels, independent of the HepG2 or Huh7 cell line.

### Relationship between serum LHBs and MDR1 expression in liver tissues from CHB patients

To further investigate the association between LHBs and MDR1, MDR1 was immunohistochemically stained in liver tissue samples from 70 CHB patients (Figure 5G), and the paired serum levels of LHBs were detected by ELISA. As shown in Figure 5H, the average MDR1 score in the group with higher LHBs concentrations (OD values of LHBs ≥ 1) was significantly higher than that in the group with lower LHBs concentrations (OD values of LHBs < 1) (47.67 ± 9.927 vs. 11.00 ± 4.249, *P* = 0.0004). The regression coefficient of MDR1 and LHBs was 0.76 (Figure 5I, P < 0.0001).

### Proteomic analysis of transcription factors

**Figure 6:**
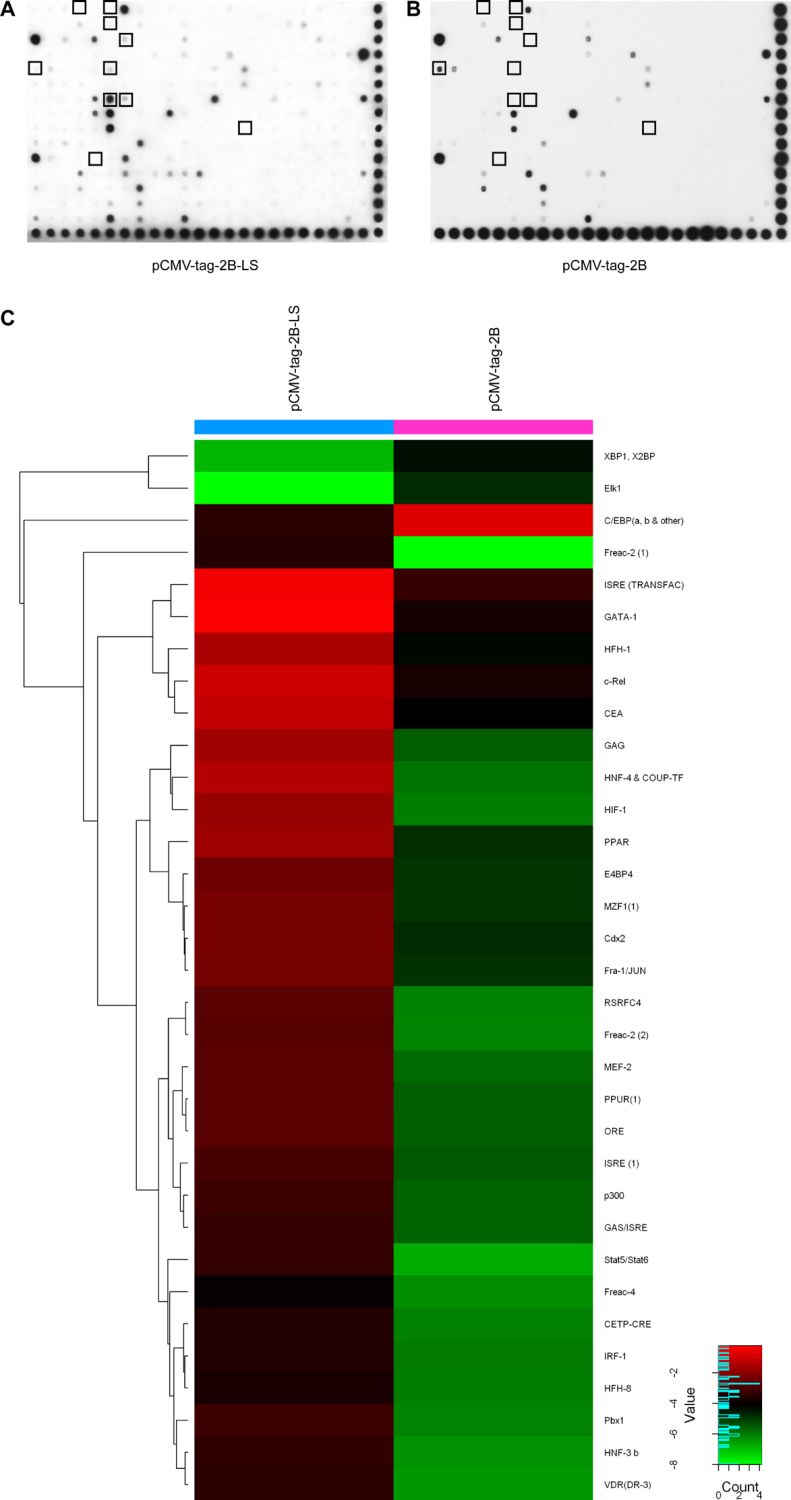
TranSignal Protein/DNA Array I analysis of HepG2 cells separately transfected with the pCMV-tag2B-LS (**A**) and pCMV-tag2B (**B**) plasmids. (**C**) Hierarchical clustering analysis of 5-fold dysregulated proteins between LHBs-overexpressing HepG2 cells and control.

Next, we characterized changes in the expression of transcription factors between HepG2 cells transfected with pCMV-tag2B-LS (1.0 μg) or pCMV-tag2B (1.0 μg) using the TranSignal Protein/DNA Array I. Of the 345 transcription factors analyzed (Figure 6A, 6B and Supplementary Table S4), 30 were upregulated and 3 were downregulated by 5-fold or more in LHBs-overexpressing HepG2 cells (Figure 6C and Supplementary Table S5). To narrow down the candidate transcription factors, ten transcriptional factors (seven ≥ 10-fold, three ≤ 0.2-fold) were selected for further study (Figure 6A, 6B and Supplementary Table S6).

### Validation of selected candidate transcription factors

**Figure 7:**
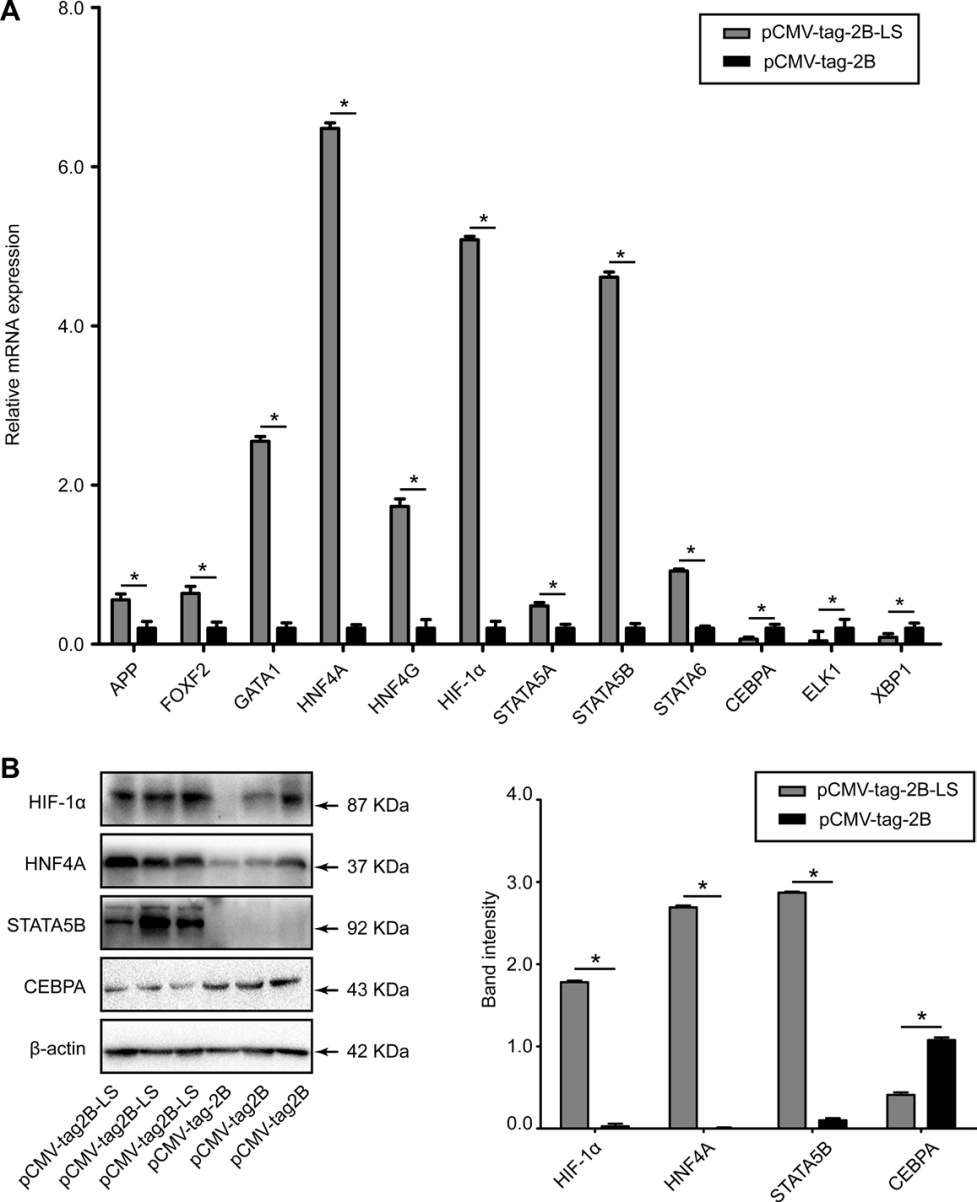
Evaluation of the differentially expressed transcription factors in HepG2 cells transfected with pCMV-tag2B-LS or pCMV-tag2B plasmid (**A**) RT-qPCR detection of the relative mRNA expression levels of APP, CEBPA, ELK1, FOXF2, GATA1, HNF4A, HNF4G, HIF-1α, STAT5A, STAT5B, STAT6, and XBP1, normalized to GADPH. (**B**) A representative western blot analysis of HNF4A, HIF-1α, STAT5B, and CEBPA expression levels in HepG2 cells transfected with the pCMV-tag2B-LS or pCMV-tag2B plasmid. (Bars indicate SD, significant as compared to the control, *P < 0.05.)

Figure 7A shows that the mRNA levels of APP, FOXF2, GATA1, HNF4A, HNF4G, HIF-1α, STAT5A, STAT5B, and STAT6 were upregulated, while CEBPA, ELK1, and XBP1 were downregulated in the HepG2 cells transfected with the pCMV-tag2B-LS plasmid, compared to HepG2 cells transfected with the pCMV-tag2B plasmid. Figure 7B shows upregulation of HNF4A, HIF-1α, and STAT5B, and a marked downregulation of CEBPA in the cells transfected with pCMV-tag2B-LS at the protein level (all *P* < 0.05). The results from the mRNA and protein analyses were similar to those shown by the protein/DNA array.

### Involvement of HIF-1α in LHBs-mediated MDR1 induction

**Figure 8:**
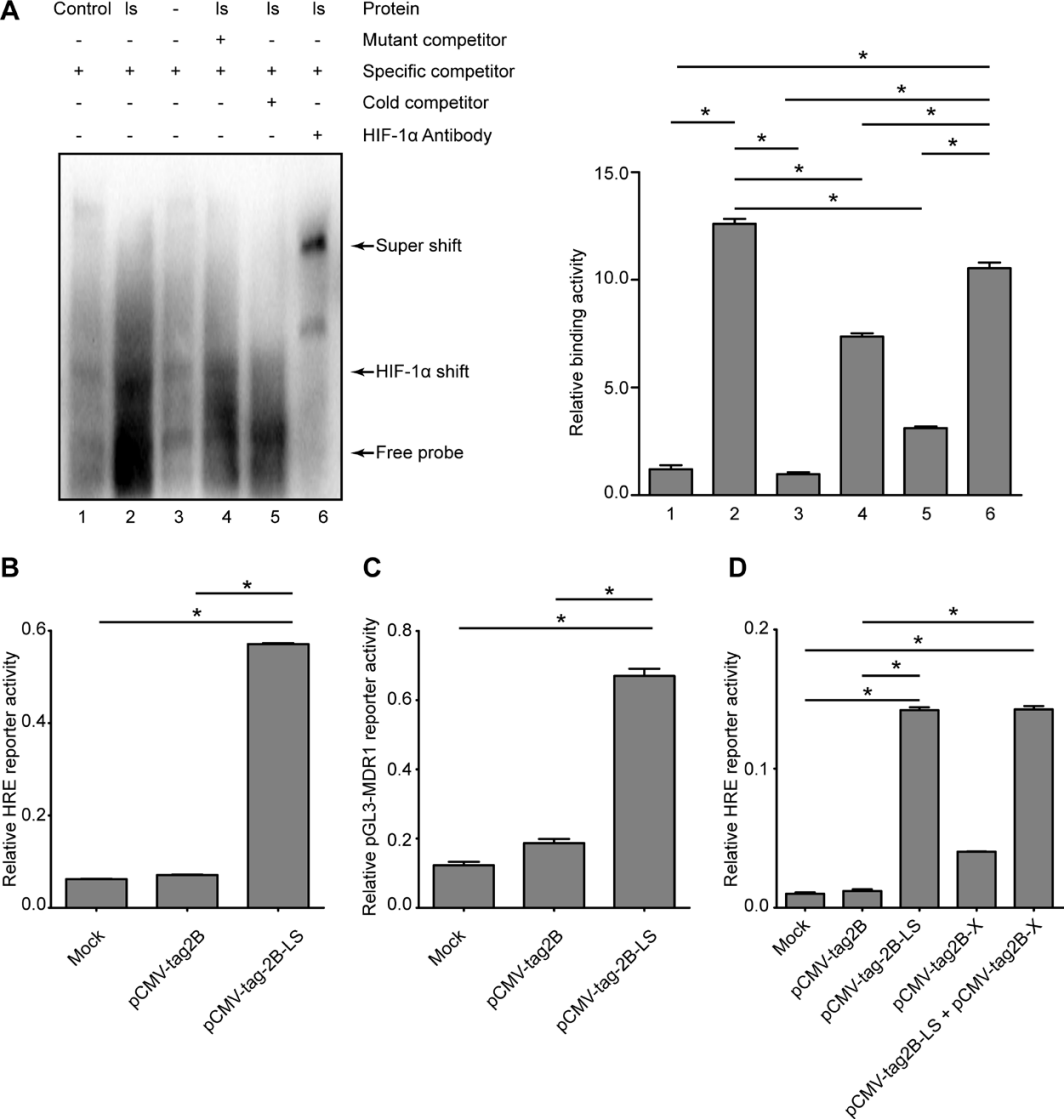
Detection of transcriptional activities of HIF-1α and MDR1 in LHBs-overexpressed HepG2 cells (**A**) Confirmation of DNA binding activity of the HIF-1α and nuclear extracts from the samples of LHBs-overexpressed cells using EMSA. (**B**) Detection of the relative HRE reporter activity by LHBs overexpression. (**C**) Detection of the relative pGL3-MDR1 reporter activity by LHBs overexpression. (**D**) Detection of the relative HRE reporter activity by LHBs and/or X protein overexpression. (Bars indicate SD, significant as compared to the control, *P < 0.05.)

HIF-1α was significantly elevated at both the mRNA (Figure 7A) and protein levels (Figure 7B) in the pCMV-tag2B-LS-transfected cells, suggesting that LHBs induced a high expression level of HIF-1α. To further confirm the effect of LHBs on HIF-1α activation, we performed an EMSA. As shown in Figure 8A, HIF-1α exhibited ten-fold higher activity in the LHBs-overexpressing cells than in the controls. These changes were in line with the array analysis results. Next, a coreporter gene assay by cotransfecing HepG2 cells with pCMV-tag2B-LS plasmid and the HRE reporter (HRE-Luc)/pGL3-MDR1 plasmid were carried out. Activation of the HRE and pGL3-MDR1 reporter activity were detected in LHBs-overexpressing cells (Figure 8B and 8C), which indicating that HIF-1α upregulation mediated by LHBs was a critical factor for MDR1 gene overexpression. Then, HepG2 cells were transfected with pCMV-tag2B-LS and pCMV-tag2B-X plasmid separately or mixed to explore the relationship between the overexpression and the induction of MDR1. Figure 8D shows that the HRE activity was 14-fold and 4-fold higher in LHBs- and X protein-overexpressed cells, respectively. Similarly, the HRE activity was elevated by 14-fold when the cells were cotransfected with the two plasmids.

## DISCUSSION

In this study, we used the iTRAQ proteomic method to identify DEPs in the liver tissues from CHB patients and controls without HBV infection. The expression levels of 71 proteins were significantly altered between the two groups. The altered expression levels of APCS, AP1B1, CFL1, DBI, EIF4B, FGB, HSPB1, MAP4, MDR1, MPO, NCL, OLA1, PSMD14, PTBP1, RPSA, RPS25, SND1, and VTN were confirmed by real-time RT-qPCR and western blot assays. First, we detected a consistent association between LHBs and an elevated expression of MDR1 in stably and transiently HBV DNA-transfected cells and liver tissues from CHB patients. Furthermore, we uncovered that the LHBs-induced upregulation of MDR1 expression was mediated through HIF-1α [14, 15].

MDR1 expression is preferably expressed in the liver, and a basic function of MDR1 is to detoxify hepatocytes through pumping out toxicants to protect liver cells. HBV infection produces a huge pool of HBsAg, including LHBs. These HBsAg particles cause pathological injury to infected cells in both experimental and clinical settings. One example is that the accumulation of the pre-S1 protein was observed in the livers of HBV transgenic mice that expressed the entire large HBsAg protein, which induced significant liver injury resembling the pathological changes of hepatitis [15]. Another example is the observation of ground-glass cells in HBV-infected livers, which give a distinct abnormal appearance that is caused by the intracellular accumulation of HBsAg [16].

However, as evidenced by the high level of serum HBsAg and that the majority of CHB patients do not have prominent liver injury, we assume that the efficiency of HBsAg particle secretion is generally sufficient. It is conceivable that active HBV replication may cause stress in infected cells that may trigger a cellular response in which MDR1 expression could be upregulated to deal with a high intracellular HBsAg concentration. Thus, it seems to us that the pathological changes generated by the accumulation of HBsAg could also induce MDR1 expression to respond to a viral insult. In our view, the elevated expression of MDR1 represents a natural cellular response to HBV infection, but it may undermine the efficacy of antiviral agents in the treatment of CHB infection.

Our investigation of transcription factors suggested the possible involvement of HIF-1α in mediating the elevation of MDR1 expression initiated by LHBs. The increased expression of HIF-1α in LHBs-overexpressing cells was validated by western blot and real-time PCR assays, and HIF-1α transcriptional activity was confirmed by EMSA and reporter gene analysis. HIF-1α has been reported to be a common pathway for the infection of human oncogenic viruses including HBV [17], suggesting that HIF-1α regulates the overexpression of MDR1. As previous studies have reported that HBx protein transactivates the human MDR1 gene through HIF-1α, which is thought to be responsible for chemotherapeutic resistance in HCC [13], we further studied the relationship of LHBs and X protein expression with this activation. LHBs appears to be stronger than X protein in activating HIF-1α.

OTUB1 was another upregulated protein found in this study. It is a hydrolase that can specifically remove ‘Lys-48’-conjugated ubiquitin from proteins, and it regulates the level of protein turnover by preventing degradation [18]. The recruitment of OTUB1 to tumor necrosis receptor-associated factor 3 (TRAF3), a ubiquitin ligase required for virus-triggered interferon response factor 3 (IRF3) and nuclear factor (NF)-κB activation, inhibited the ubiquitination of HSCARG, a newly reported negative regulator of NF-κB in viral infection [19]. The overexpression of OTUB1 inhibited virus-induced activation of IRF3 and NF-κB, whereas knockdown of OTUB1 had the opposite effects. These findings suggest that OTUB1 may negatively regulate virus-triggered type I interferon induction and the cellular antiviral response by deubiquitinating TRAF3. Further studies are needed to determine whether OTUB1 exerts a similar function during HBV infection.

SND1 was also found to be upregulated in this study. SND1 is related to pathways involved in Epstein–Barr virus infection [20] and viral carcinogenesis. In addition, immunohistochemical analysis of 109 HCC liver samples documented higher SND1 expression in 81 cases (74%), compared to the normal liver, and SND1 expression was gradually increased with the stages and grades of the disease. Moreover, the overexpression of SND1 downregulated the tumor suppressor mRNA levels in HCC cells, while knockdown of SND1 upregulated them [21]. In the current study, SND1 expression was higher in the liver tissues of HBV-infected patients, but the exact impact on the course of HBV infection and pathogenesis remains to be determined.

In this study, FGB was downregulated in the liver tissues of CHB patients. This finding was consistent with a published report, which showed that the level of FGB was significantly decreased in the plasma of 116 HBV-infected individuals by western blot analysis, compared with 70 normal subjects [22].

In conclusion, this study presented comparative proteomic profiles of liver tissues from CHB patients and individuals without HBV infection. Seventy-one differentially expressed proteins that are possibly associated with HBV infection were identified, and the correlation between MDR1 and HBV infection was further probed and confirmed. For the first time, we showed that wild-type LHBs can transcriptionally elevate MDR1 expression through increasing HIF-1α expression and activity. Therefore, an increased MDR1 expression in response to HBV infection may compromise the efficacy of antiviral agents in the treatment of CHB patients.

## MATERIALS AND METHODS

### Patients and tissue collection

Patients with CHB were diagnosed according to the guidelines of the American Association for the Study of Liver Diseases, the European Association for the Study of the Liver, and/or the Asian Pacific Association for the Study of the Liver [23–25]. Controls without HBV infection were recruited from those who tested negative for HBV infection but had a liver rupture, cyst, or angioma. Liver tissue samples were obtained through liver biopsy from the CHB patients and through hepatectomy from controls. All individuals in this study were negative for human immunodeficiency virus and hepatitis virus C infection. All the liver tissues were collected at the Second Affiliated Hospital of Chongqing Medical University from January 2013 to October 2015. This study was approved by the Ethics Committee of Chongqing Medical University in accordance with the principles stated in the Declaration of Helsinki, and written informed consent was obtained from each participant.

### Cell lines and culture conditions

The human HCC cell lines HepAD38, Huh 7, HepG2, and its derivative HepG2.2.1.5 cells were purchased from the American Type Culture Collection (Manassas, VA). All cells were maintained in high-glucose Dulbecco's modified Eagle's medium (DMEM, HyClone, Waltham, MA) supplemented with 10% fetal bovine serum (FBS) (Gibco, San Diego, CA), 100 IU/mL penicillin, and 100 μg/mL streptomycin at 37.0°C in 5% CO_2_. In addition, HepAD38 cells were cultured in the presence of 0.3 mg/mL tetracycline and 400 mg/mL G418 (GIBCO BRL/Life Technologies).

### Protein extraction, iTRAQ labeling, and peptide fractionation

Total protein from each liver tissue sample was extracted using a Sample Grinding Kit (Amersham Biosciences) that contained lysis buffer (7 M urea, 1 mg/mL DNase I, 1 mM Na_3_VO_4_, and 1 mM phenylmethylsulfonyl fluoride). The cell lysate was centrifuged at 15,000 rpm for 30 min. The supernatant was collected, and then the concentration of the total protein was determined by using a 2-D Quantification Kit (Amersham Biosciences). All extraction steps were carried out at 4°C.

Following the iTRAQ protocol (8-plex, Applied Biosystems, Foster City, CA), the prepared proteins (100 μg) for each pool were precipitated, redissolved, denatured, cysteine blocked, digested, and then labeled with the iTRAQ tags as follows: seven controls without HBV infection were pooled and labeled with the 113 tag; CHB (HBeAg-positive) patients 1 to 7 were labeled with the iTRAQ tags of 114, 115, 116, 117, 118, 119, and 121, respectively. In addition, duplicate labeling was performed to eliminate the effects of the patients’ HBeAg status, using the prepared proteins from another set of samples including seven controls without HBV infection and seven CHB (but HBeAg-negative) patients with the same order of iTRAQ tags. All labeled samples were selected randomly from each group.

The prepared peptides were fractionated by immobilized pH gradient (IPG) isoelectric focusing [9] as follows: the pre-mixed iTRAQ-labeled peptide samples were redissolved in 300 μL of 8 M urea and 1% Pharmalyte solution (Amersham Biosciences), and then rehydrated on an IPG strip (pH 3–10, 18 cm long, Amersham Biosciences) for 14 h at 30 V. Subsequently, the peptides were focused for a total of 68 kV·h with an IPGphor isoelectric focusing system (Amersham Biosciences). The strips were removed and quickly cut into 36 pieces of 5-mm fractions with 100 μL of Buffer A (2% acetonitrile and 0.1% formic acid) for 1 h. Then, these fractions were lyophilized and desalted with a C-18 Discovery^®^ DSC-18 SPE column (100 mg capacity, Supelco, Sigma-Aldrich). Thereafter, all these desalted fractions were lyophilized and stored at −20.0°C prior to mass spectrometric analysis.

### Mass spectrometry and database search

Mass spectrometry was carried out with a QStar Elite Hybrid ESI Quadrupole time-of-flight tandem mass spectrometer (Applied Biosystems, Framingham, MA), coupled to the online capillary liquid chromatography Dionex Ultimate 3000 system (Amsterdam, The Netherlands) [9]. In detail, purified peptide fractions were resuspended in 20 μL of Buffer A, half of which was injected into the liquid chromatography system. Subsequently, the peptide mixtures were separated by a C-18 PepMap column (Dionex) using a series of solvent gradients of 2–100% Buffer B (98% acetonitrile and 0.1% formic acid) at a flow rate of 0.3 μL/min. For data acquisition, positive ion survey scans were performed using a selected mass range of 300–1,800 *m/z*. The two most abundant charged peptides above 20 counts were selected for tandem mass spectrometry at a dynamic exclusion of 30 s with a mass tolerance of ± 50 mDa.

ProteinPilot software v2.0 (Applied Biosystems, MDS-Sciex) was used to identify and quantify the proteins using a threshold set at a 5% false discovery rate with 95% confidence, and then the MS/MS data were searched on the UniProt database (http://www.uniprot.org/) [9, 26]. Protein identification was based on a selection threshold of ProtScore > 1.3 (1.3/1) or < 0.77 (1/1.3), and at least three unique peptides with 95% confidence were required for quantitation. The relative quantification of proteins was determined on the MS/MS scans using the ratios of the peak areas of 113–119 Da and 121 Da, respectively. A *P-value* < 0.05 was considered statistically significant.

### Western blot analysis

Approximately 30 μg of protein in each lane was electrophoretically separated by sodium dodecyl sulfate–polyacrylamide gel electrophoresis and transferred onto a polyvinylidene fluoride membrane (Amersham Biosciences) for immunoblotting. Membranes were blocked for 1 h in 5% skim milk in Tris-buffered saline with Tween-20 (TBS-T) and incubated with primary antibodies (1:100–1:1000 dilution) against AP1B1, CEBPA, FGB, HIF-1α, HNF4A, MDR1, OTUB1, PSMD14, SND1, STAT5B, flag, and β-actin (Abcam, Cambridge, MA). After washing three times for 10 min with TBS-T buffer, the membranes were incubated with a horseradish peroxidase (HRP)-conjugated IgG antibody (goat anti-mouse, goat anti-rabbit, or rabbit anti-goat) (Amersham Biosciences, Uppsala, Sweden) as the secondary antibody (1:5000 dilution) for 1 h at room temperature. Finally, membranes were visualized on a ChemiDoc MP imaging system (Bio-Rad, Hercules, CA). Quantification of target proteins was normalized against β-actin and analyzed by density detection. All of the western blotting analyses were repeated at least three times.

### RNA extraction and quantitative reverse transcription–polymerase chain reaction (RT-qPCR)

Total RNA was extracted with Trizol (Gibco-BRL, Gaithersburg, MD), according to the manufacturer's instructions. First-strand cDNA was reverse-transcribed from 2 μg of total RNA using an A3500 Reverse Transcription System (Promega, Madison, WI). RT-qPCR was performed on an ABI 7300 system (Applied Biosystems, Foster City, CA) using a TaqMan^®^ TAMRA^™^ PROBE 5′FAM^TM^ Kit (Kapa Biosystems, Wilmington, MA) and gene-specific primers for the selected genes (Invitrogen, Carlsbad, CA), which are listed in Supplementary Table S7.

Relative quantification of gene expression was calculated with the 2^−ΔΔCT^ method [27]. RT-qPCR quantification of each of the targeted genes was repeated at least three times.

### Plasmid and transfecion

The plasmid pEcob6 was kindly provided by Tim Harrison (The Royal Free Hospital, Universtiy of London, UK), which contained two copies of tandem HBV DNA sequences. Four amplified fragments (Supplementary Table S8), following the template of pEcob6, were cloned into the N-terminal Flag-tagged mammalian expression vector pCMV-Tag2B (Stratagene, La Jolla, CA) to construct LHBs (pCMV-Tag2B-LS)-, MHBs (pCMV-Tag2B-MS)-, SHBs (pCMV-Tag2B-SS)- and X protein-expressed plasmids (pCMV-Tag2B-X), respectively.

Cells were seeded in 6-well plates one day before transfection at a density of 5 × 10^5^/well. Then, the plasmid was transfected into the prepared cells using Lipofectamine 2000 reagent (Invitrogen-Life Technologies, Carlsbad, CA) in the presence of serum-free medium. The transfected cells were harvested at 48 h post-transfection for further analysis.

### Immunohistochemical analysis (IHC) and enzyme-linked immunosorbent assay (ELISA)

IHC evaluation of MDR1 was performed on 70 liver biopsy tissues from patients with CHB. Sections were paraffin-embedded and baked at 60.0°C and then were incubated overnight at 4.0°C with antibodies against MDR1 (1:100 dilution). After incubation with HRP-conjugated secondary antibodies, the stained sections were detected with an Envision/HRP system (Dako-Cytomation, Glostrup, Denmark), evaluated, and scored, respectively, by three qualified pathologists who were blinded to the clinical data. The protein expression levels of these sections were analyzed by using a semi-quantitative scoring method that assessed both staining intensity (scale: 0–3) and the percentage of positive cells (0–100%), resulting in a final score ranging from 0 to 300 when multiplied.

The serum LHBs levels of patients whose liver tissue was analyzed by IHC were measured using an ELISA kit (Beijing Hotgen Biotech Co., Ltd., Beijing, China) [28], with a minimum detection level of 5 ng/mL. All measurements were carried out in triplicate.

### Protein/DNA array analysis

Proteomic/DNA analyses were performed by using a TranSignal Protein/DNA Array I (Panomics Inc., Redwood City, CA). Briefly, nuclear proteins from samples were extracted, quantified, incubated, and eluted. Then, the bound probes were hybridized to a membrane containing an array of 345 transcription factor consensus binding sequences (Spin Column version, Panomics, Freemont, CA). After washing, the DNA/protein array was incubated with a HRP-conjugated streptavidin solution (Pierce) and visualized by using a HRP Substrate Working Solution (Millipore, Billerica, MA). The images of the chemiluminescent signal were captured by a Syngene GBox Imaging System (Cambridge, UK) and quantitated with ScanAlyze software.

### Electrophoretic mobility shift assay (EMSA)

EMSA was performed following the manufacturer's protocol with the Chemiluminescent Nucleic Acid Detection Module (Pierce, Biotechnology, Inc., Rockford, IL, USA). In brief, 5 mg of nuclear extract was prepared identically as in the protein/DNA assay, mixed with 10 μL of binding buffer (10 mM Tris-HCl pH 7.5, 50 mM KCl, 1 mM DDT, 1 mg of poly dI–dC, 2.5% glycerol, 5 mM MgCl_2_, and 1 mM EDTA), and incubated with or without 1 μL of unlabeled competitor or mutant oligonucleotides of HIF-1α (5′-AGT TGA GGC GAC TTT CCC AGG C-3′) for 10 min at room temperature. Then, the oligonucleotides of biotin-labeled HIF-1α (5′-TCT GTA CGT GAC CAC ACT CAC CTC-3′) were added and co-incubated for another 20 min at room temperature. Samples were loaded onto a 4% polyacrylamide gel and separated in 0.5 × TBE at 4°C, followed by transfer to a PVDF membrane. After crosslinking under ultraviolet light, the membrane was probed with HRP-conjugated streptavidin solution (Pierce) at room temperature for 30 min. Finally, the membrane was visualized by using HRP Substrate Working Solution (Millipore) and imaged with a ChemiDoc MP imaging system (Bio-Rad, Hercules, CA).

### Cotransfection and reporter gene analysis

HepG2 cells were seeded in 24-well plates one day before transfection at 0.5 × 10^5^ cells/well and then transiently cotransfected with 0.5 μg of pCMV-tag2B (blank vector as a negative control)/pCMV-tag2B-LS and/or pCMV-tag2B-X/pGL3-MDR1 plasmid (Promega, Madison, WI), 0.5 μg of hypoxia response element (HRE) reporter plasmid (PGL4.42, Promega, Madison, WI), and 0.15 μg of pRL-SV40 plasmid, which expressed *Renilla luciferase* for normalization (Promega). The luciferase signal was detected and normalized with *Renilla luciferase* using a dual-luciferase reporter assay system (Promega, Madison, WI) 2 days after transfection. Each transfection experiment was repeated at least three times.

### Bioinformatics

The Gene Ontology was analyzed by the Evolutionary Relationships (PANTHER) Classification System (http://www.pantherdb.org/) for molecular functions, biological processes, and protein classes.

### Statistical analyses

Statistical analyses were carried out with Statistical Package for the Social Sciences software, version 20.0 (Chicago, IL). Quantitative variables are presented as mean ± standard deviation (SD) or median (range). Quantitative differences between groups were analyzed by the Student's *t-test*. Categorical variables were compared using Pearson's chi-squared test or Fisher's exact test, as appropriate. Pearson's correlation was used to evaluate possible associations. *P*-values < 0.05 were considered statistically significant, and all tests for significance were two-tailed.

## SUPPLEMENTARY MATERIALS FIGURES AND TABLES




