# Supplementary material for: LHBs can elevate the expression of MDR1 through HIF-1α in patients with CHB infection: a comparative proteomic study
Source: Oncotarget. 2016 Dec 15;8(3):4549–62. doi: 10.18632/oncotarget.13941 (PMC5354853; doi:10.18632/oncotarget.13941)
Supplement: Supplementary file 1 [file oncotarget-08-4549-s001.pdf]

LHBs can elevate the expression of MDR1 through HIF-1α in patients with CHB infection: a comparative proteomic study

Supplementary Materials

Supplementary Table S1: Demographic and clinic characteristics of individuals included in this study

Group	Total (*n*)	Age (year) mean ± SD	Gender (male/female)	ALT (U/L) (median, range)	HBsAg status (n) (positive/negative)	HBeAg status (*n*) (positive/negative)	HBV DNA (log copies/ml) (median, range)
Healthy controls	26	29 ± 14.1	19/7	23 (8,51)	0/26	NA	NA
Chronic hepatitis B	102	37 ± 11.4	72/30	154 (61,627)	102/0	56/46	5.23 (3.12, 8.90)

Abbreviations: ALT, alanine aminotransferase; HBsAg, hepatitis B surface antigen; HBeAg, hepatitis B e antigen; HBV DNA, hepatitis B virus deoxyribose nucleic acid; NA, not applicable.

Supplementary Table S2: List of DPSs between CHB patients and non-HBV infected individuals by iTRAQ. See Supplementary_Table_S2

Supplementary Table S3: Partial list of proteins found to be deferentially expressed between CHB patients and non-HBV infected individuals by iTRAQ. See Supplementary_Table_S3

Supplementary Table S4: List of the 345 transcriptional factors detected between pCMV-tag2B-LS and pCMV-tag2B plasmid transfected HepG2 cells. See Supplementary_Table_S4

Supplementary Table S5: List of differentially expressed transcriptional factors between pCMV-tag2B-LS and pCMV-tag2B plasmid transfected HepG2 cells (at least 5-fold). See Supplementary_Table_S5

Supplementary Table S6: List of differentially expressed transcriptional factors between pCMV-tag2B-LS and pCMV-tag2B plasmid transfected HepG2 cells (At least 10-fold in up-regulated and 5-fold in down-regulated). See Supplementary_Table_S6

Supplementary Table S7: List of gene-specific primers used in this study. See Supplementary_Table_S7

Supplementary Table S8: Primers, length of qRT-PCRproducts, and restriction enzyme cutting sites of LHBs, MHBs, SHBs and X protein of HBV

Protein name	Forward primer	Reverse primer	Length of PCR product (bp)	Restriction enzyme cutting site
Large surface protein of HBV (LHBs)	5′-CTGCAGGGGCAG AATCTTTCCACCA-3′	5′-AAGCTTTTAAATGT ATACCCAAAGACAA-3′	1,170	Pst I: CTGCAG Hand III: AAGCTT
Middle surface protein of HBV (MHBs)	5′-CTGCAGCAGTGG AATTCCACAACCT-3′	5′-AAGCTTTTAAATGT ATACCCAAAGACAA-3′	843	Pst I: CTGCAG Hand III: AAGCTT
Small surface protein of HBV (SHBs)	5′-GAATTCGAGAA CATCGCATCAGGA-3′	5′-CTCGAGTTAAATG TATACCCAAAGACA-3′	679	EcoR I :GAATTC Xho I: CTCGAG
X protein of HBV (HBx)	5′-CCGCTCGAGATGG CTGCTAGGGTGTG-3′	5′-CGGAATTCCTAGGC AGAGGTGAAAAAGT-3′	465	Xho I: CTCGAG EcoR I :GAATTC
