# Supplementary material for: LHBs can elevate the expression of MDR1 through HIF-1α in patients with CHB infection: a comparative proteomic study
Source: Oncotarget. 2016 Dec 15;8(3):4549–62. doi: 10.18632/oncotarget.13941 (PMC5354853; doi:10.18632/oncotarget.13941)
Supplement: Supplementary file 2 [file oncotarget-08-4549-s002.docx]

**Supplementary Table 2.List of DPSs between CHB patients and non-HBV infected individuals by iTRAQ.**

| N | Entry | Protein names | Gene names | Peptides(95%)  (HBeAg+) | Peptides(95%)  (HBeAg-) | Chronic hepatitis B: pooled healthy controls | | | | | | | | | | | | | | | | | | | | | | | | | | | |
| --- | --- | --- | --- | --- | --- | --- | --- | --- | --- | --- | --- | --- | --- | --- | --- | --- | --- | --- | --- | --- | --- | --- | --- | --- | --- | --- | --- | --- | --- | --- | --- | --- | --- |
|  |  |  |  |  |  | 114:113 | | | | 115:113 | | | | 116:113 | | | | 117:113 | | | | 118:113 | | | | 119:113 | | | | 121:113 | | | |
|  |  |  |  |  |  | HBeAg(+) | Pval | HBeAg(-) | PVal | HBeAg(+) | Pval | HBeAg(-) | PVal | HBeAg(+) | Pval | HBeAg(-) | PVal | HBeAg(+) | Pval | HBeAg(-) | PVal | HBeAg(+) | Pval | HBeAg(-) | PVal | HBeAg(+) | Pval | HBeAg(-) | PVal | HBeAg(+) | Pval | HBeAg(-) | PVal |
| 1 | Q15942 | Zyxin (Zyxin-2) | ZYX | 7 | 5 | 0.7447 | 0.8913 | 0.6730 | 0.2811 | 1.0666 | 0.9657 | 1.2706 | 0.1893 | 0.7727 | 0.4814 | 1.1803 | 0.9436 | 1.0000 | 0.9400 | 1.3804 | 0.6286 | 1.5276 | 0.4019 | 0.5346 | 0.5913 | 3.3729 | 0.0017 | 1.1066 | 0.9543 | 2.3335 | 0.0446 | 2.2080 | 0.0990 |
| 2 | Q7Z2W4 | Zinc finger CCCH-type antiviral protein 1 (ADP-ribosyltransferase diphtheria toxin-like 13) (ARTD13) (Zinc finger CCCH domain-containing protein 2) (Zinc finger antiviral protein) (ZAP) | ZC3HAV1 ZC3HDC2 PRO1677 | 8 | 6 | 2.9648 | 0.8973 | 2.2284 | 0.2872 | 2.5351 | 0.5429 | 3.0761 | 0.2122 | 2.1478 | 0.4640 | 0.8630 | 0.7796 | 1.3804 | 0.2019 | 0.7727 | 0.6384 | 2.6062 | 0.7877 | 1.5704 | 0.4488 | 3.4041 | 0.9665 | 3.4356 | 0.1936 | 2.5586 | 0.5623 | 1.7378 | 0.3851 |
| 3 | P63104 | 14-3-3 protein zeta/delta (Protein kinase C inhibitor protein 1) (KCIP-1) | YWHAZ | 27 | 22 | 0.3733 | 0.0341 | 0.5598 | 0.2972 | 0.9290 | 0.8409 | 0.5970 | 0.0752 | 0.6026 | 0.0711 | 1.2589 | 0.0444 | 0.6792 | 0.1942 | 0.7943 | 0.4730 | 0.7516 | 0.3657 | 1.1588 | 0.1706 | 1.4997 | 0.1862 | 0.8395 | 0.3882 | 0.9204 | 0.6032 | 2.2080 | 0.0026 |
| 4 | P27348 | 14-3-3 protein theta (14-3-3 protein T-cell) (14-3-3 protein tau) (Protein HS1) | YWHAQ | 21 | 23 | 0.7244 | 0.8830 | 0.5346 | 0.5612 | 1.2823 | 0.5830 | 0.9462 | 0.7156 | 0.6310 | 0.9975 | 1.4060 | 0.2422 | 1.8535 | 0.4041 | 1.1588 | 0.9378 | 2.8054 | 0.0999 | 1.0375 | 0.6260 | 3.8371 | 0.0221 | 0.6368 | 0.6966 | 2.8054 | 0.0566 | 0.9908 | 0.2538 |
| 5 | Q04917 | 14-3-3 protein eta (Protein AS1) | YWHAH YWHA1 | 18 | 16 | 0.6918 | 0.6110 | 1.3428 | 0.9269 | 3.9084 | 0.3360 | 2.2080 | 0.1661 | 2.9376 | 0.3839 | 3.0479 | 0.4047 | 3.8726 | 0.3772 | 2.8314 | 0.3225 | 2.7797 | 0.4432 | 2.8314 | 0.5235 | 2.9923 | 0.3687 | 3.5318 | 0.1416 | 1.4723 | 0.5147 | 4.1687 | 0.1571 |
| 6 | P61981 | 14-3-3 protein gamma (Protein kinase C inhibitor protein 1) (KCIP-1) [Cleaved into: 14-3-3 protein gamma, N-terminally processed] | YWHAG | 27 | 20 | 0.3436 | 0.2382 | 0.8241 | 0.8879 | 1.0375 | 0.9877 | 1.3305 | 0.3029 | 1.1912 | 0.6145 | 1.3677 | 0.1113 | 1.1169 | 0.9052 | 1.0666 | 0.8860 | 0.4831 | 0.1062 | 1.3062 | 0.4666 | 1.1376 | 0.3472 | 1.5276 | 0.1569 | 0.7311 | 0.3554 | 1.4454 | 0.1876 |
| 7 | P62258 | 14-3-3 protein epsilon (14-3-3E) | YWHAE | 27 | 26 | 0.3373 | 0.0077 | 0.2858 | 0.0012 | 0.7244 | 0.2773 | 0.5970 | 0.1463 | 0.8872 | 0.0445 | 0.7178 | 0.6321 | 0.6427 | 0.0501 | 0.3342 | 0.0019 | 0.6486 | 0.0320 | 0.5702 | 0.1226 | 0.5649 | 0.0048 | 0.6668 | 0.2215 | 0.6668 | 0.0259 | 0.8017 | 0.3567 |
| 8 | P31946 | 14-3-3 protein beta/alpha (Protein 1054) (Protein kinase C inhibitor protein 1) (KCIP-1) [Cleaved into: 14-3-3 protein beta/alpha, N-terminally processed] | YWHAB | 29 | 21 | 0.4742 | 0.5274 | 0.6427 | 0.5416 | 0.9638 | 0.6633 | 0.6310 | 0.4525 | 0.8551 | 0.5056 | 1.2134 | 0.7530 | 0.7244 | 0.4893 | 0.7112 | 0.5034 | 0.6918 | 0.4798 | 1.1169 | 0.7596 | 1.7539 | 0.2498 | 1.0375 | 0.7611 | 1.4191 | 0.4010 | 1.2706 | 0.5691 |
| 9 | P07947 | Tyrosine-protein kinase Yes (EC 2.7.10.2) (Proto-oncogene c-Yes) (p61-Yes) | YES1 YES | 4 | 3 | 0.1660 | 0.0134 | 1.5276 | 0.7205 | 0.6918 | 0.1475 | 0.9036 | 0.7993 | 0.0497 | 0.0108 | 1.5849 | 0.6625 | 0.4613 | 0.0807 | 1.5849 | 0.7777 | 0.7379 | 0.1934 | 1.3062 | 0.9504 | 0.7047 | 0.2317 | 0.4613 | 0.4856 | 0.7379 | 0.1404 | 2.3121 | 0.4030 |
| 10 | P67809 | Nuclease-sensitive element-binding protein 1 (CCAAT-binding transcription factor I subunit A) (CBF-A) (DNA-binding protein B) (DBPB) (Enhancer factor I subunit A) (EFI-A) (Y-box transcription factor) (Y-box-binding protein 1) (YB-1) | YBX1 NSEP1 YB1 | 10 | 5 | 1.1912 | 0.2721 | 1.0280 | 0.6439 | 0.9204 | 0.5115 | 0.3565 | 0.6008 | 1.0864 | 0.4114 | 3.4356 | 0.0553 | 1.0093 | 0.7950 | 1.3932 | 0.6698 | 1.1803 | 0.6427 | 2.5119 | 0.1658 | 1.2474 | 0.0476 | 2.5119 | 0.1041 | 1.2474 | 0.0931 | 2.2080 | 0.0839 |
| 11 | P54577 | Tyrosine--tRNA ligase, cytoplasmic (EC 6.1.1.1) (Tyrosyl-tRNA synthetase) (TyrRS) [Cleaved into: Tyrosine--tRNA ligase, cytoplasmic, N-terminally processed] | YARS | 10 | 6 | 0.8630 | 0.3141 | 0.9550 | 0.2821 | 0.9817 | 0.9545 | 0.9908 | 0.9073 | 0.9550 | 0.7255 | 0.9204 | 0.1112 | 1.0000 | 0.7012 | 0.9638 | 0.3207 | 0.9817 | 0.8835 | 0.9817 | 0.5194 | 1.0666 | 0.3984 | 0.9727 | 0.6100 | 1.0864 | 0.4768 | 1.0666 | 0.4864 |
| 12 | O75191 | Xylulose kinase (Xylulokinase) (EC 2.7.1.17) | XYLB | 5 | 8 | 1.6444 | 0.9568 | 1.1066 | 0.8676 | 1.0864 | 0.6635 | 1.0471 | 0.9661 | 0.7727 | 0.4667 | 0.7727 | 0.8767 | 0.8630 | 0.5906 | 0.2443 | 0.2287 | 1.2023 | 0.8424 | 0.3945 | 0.0154 | 0.6194 | 0.9285 | 1.3062 | 0.4352 | 0.6607 | 0.5118 | 0.1614 | 0.0509 |
| 13 | P12956 | X-ray repair cross-complementing protein 6 (EC 3.6.4.-) (EC 4.2.99.-) (5'-deoxyribose-5-phosphate lyase Ku70) (5'-dRP lyase Ku70) (70 kDa subunit of Ku antigen) (ATP-dependent DNA helicase 2 subunit 1) (ATP-dependent DNA helicase II 70 kDa subunit) (CTC box-binding factor 75 kDa subunit) (CTC75) (CTCBF) (DNA repair protein XRCC6) (Lupus Ku autoantigen protein p70) (Ku70) (Thyroid-lupus autoantigen) (TLAA) (X-ray repair complementing defective repair in Chinese hamster cells 6) | XRCC6 G22P1 | 15 | 18 | 0.9462 | 0.8257 | 1.2359 | 0.6280 | 0.9817 | 0.9322 | 1.0280 | 0.9649 | 1.2246 | 0.7636 | 1.3062 | 0.7600 | 0.8790 | 0.8010 | 1.2359 | 0.8664 | 1.2023 | 0.9081 | 1.5136 | 0.1246 | 1.5849 | 0.0168 | 1.0864 | 0.7277 | 1.1169 | 0.5168 | 1.3932 | 0.2434 |
| 14 | P13010 | X-ray repair cross-complementing protein 5 (EC 3.6.4.-) (86 kDa subunit of Ku antigen) (ATP-dependent DNA helicase 2 subunit 2) (ATP-dependent DNA helicase II 80 kDa subunit) (CTC box-binding factor 85 kDa subunit) (CTC85) (CTCBF) (DNA repair protein XRCC5) (Ku80) (Ku86) (Lupus Ku autoantigen protein p86) (Nuclear factor IV) (Thyroid-lupus autoantigen) (TLAA) (X-ray repair complementing defective repair in Chinese hamster cells 5 (double-strand-break rejoining)) | XRCC5 G22P2 | 20 | 14 | 0.7178 | 0.0781 | 0.8954 | 0.6510 | 0.5916 | 0.0621 | 0.7311 | 0.1542 | 0.6138 | 0.1523 | 1.2023 | 0.3932 | 0.8872 | 0.2076 | 0.6792 | 0.1876 | 0.9908 | 0.7178 | 1.0093 | 0.6686 | 1.5136 | 0.1646 | 0.8790 | 0.3318 | 1.3804 | 0.4509 | 1.1482 | 0.5998 |
| 15 | O14980 | Exportin-1 (Exp1) (Chromosome region maintenance 1 protein homolog) | XPO1 CRM1 | 3 | 9 | 1.6444 | 0.2355 | 1.5560 | 0.4115 | 1.6444 | 0.1546 | 3.4041 | 0.1062 | 0.8790 | 0.6660 | 4.2073 | 0.0304 | 1.0186 | 0.9625 | 2.0893 | 0.1615 | 1.4322 | 0.2626 | 3.0479 | 0.0359 | 0.9120 | 0.4655 | 2.6792 | 0.0653 | 0.5861 | 0.4875 | 3.1333 | 0.0705 |
| 16 | Q9NQH7 | Probable Xaa-Pro aminopeptidase 3 (X-Pro aminopeptidase 3) (EC 3.4.11.9) (Aminopeptidase P3) (APP3) | XPNPEP3 | 3 | 5 | 0.9727 | 0.8648 | 0.3532 | 0.1389 | 2.4660 | 0.4197 | 0.5105 | 0.4439 | 2.4434 | 0.4549 | 0.6546 | 0.4155 | 0.6138 | 0.4430 | 0.3873 | 0.2617 | 2.3121 | 0.4740 | 0.4169 | 0.2149 | 1.1803 | 0.9776 | 0.8017 | 0.5604 | 1.3677 | 0.9756 | 0.2312 | 0.1183 |
| 17 | P47989 | Xanthine dehydrogenase/oxidase [Includes: Xanthine dehydrogenase (XD) (EC 1.17.1.4); Xanthine oxidase (XO) (EC 1.17.3.2) (Xanthine oxidoreductase) (XOR)] | XDH XDHA | 17 | 21 | 0.3698 | 0.0048 | 0.8872 | 0.6992 | 0.7943 | 0.4911 | 1.3428 | 0.8031 | 0.7870 | 0.2332 | 0.8091 | 0.3637 | 0.8710 | 0.7131 | 1.6596 | 0.3593 | 0.8395 | 0.3890 | 1.3552 | 0.6909 | 0.2938 | 0.0001 | 1.4723 | 0.7013 | 0.4699 | 0.0078 | 1.2706 | 0.7217 |
| 18 | O75083 | WD repeat-containing protein 1 (Actin-interacting protein 1) (AIP1) (NORI-1) | WDR1 | 21 | 24 | 0.5970 | 0.0548 | 0.9036 | 0.9143 | 0.9638 | 0.7793 | 1.1066 | 0.8545 | 1.0765 | 0.6799 | 1.3552 | 0.0800 | 0.9036 | 0.7365 | 0.9376 | 0.5179 | 1.0093 | 0.9293 | 1.5996 | 0.0097 | 1.3677 | 0.0532 | 1.2942 | 0.1959 | 0.9036 | 0.8683 | 1.4723 | 0.0314 |
| 19 | P23381 | Tryptophan--tRNA ligase, cytoplasmic (EC 6.1.1.2) (Interferon-induced protein 53) (IFP53) (Tryptophanyl-tRNA synthetase) (TrpRS) (hWRS) [Cleaved into: T1-TrpRS; T2-TrpRS] | WARS IFI53 WRS | 10 | 12 | 0.7516 | 0.5918 | 1.4322 | 0.5857 | 0.6427 | 0.9311 | 1.3305 | 0.7943 | 0.8551 | 0.2607 | 2.0137 | 0.5068 | 1.1695 | 0.4655 | 1.9588 | 0.3969 | 0.8472 | 0.7571 | 2.8314 | 0.1381 | 2.4210 | 0.0017 | 0.8166 | 0.6675 | 3.0479 | 0.0006 | 1.7378 | 0.1139 |
| 20 | P04004 | Vitronectin (VN) (S-protein) (Serum-spreading factor) (V75) [Cleaved into: Vitronectin V65 subunit; Vitronectin V10 subunit; Somatomedin-B] | VTN | 12 | 12 | 0.1076 | 0.0022 | 0.2355 | 0.0021 | 1.0765 | 0.3958 | 0.9638 | 0.6713 | 0.3311 | 0.0069 | 0.4920 | 0.0019 | 0.3532 | 0.0180 | 0.4875 | 0.0136 | 0.3342 | 0.0070 | 0.6138 | 0.0554 | 0.3048 | 0.0068 | 0.5058 | 0.0026 | 0.3837 | 0.0031 | 0.5445 | 0.0128 |
| 21 | Q96QK1 | Vacuolar protein sorting-associated protein 35 (hVPS35) (Maternal-embryonic 3) (Vesicle protein sorting 35) | VPS35 MEM3 TCCCTA00141 | 11 | 10 | 1.3183 | 0.8574 | 0.5200 | 0.1700 | 1.3305 | 0.8376 | 0.5495 | 0.3353 | 1.0568 | 0.8077 | 0.7516 | 0.9051 | 0.9290 | 0.7619 | 0.6252 | 0.9380 | 0.8091 | 0.4905 | 1.2589 | 0.4237 | 2.7542 | 0.0525 | 0.2729 | 0.1496 | 2.0512 | 0.2904 | 0.6982 | 0.5029 |
| 22 | P08670 | Vimentin | VIM | 104 | 78 | 0.2582 | 0.0000 | 1.0280 | 0.6575 | 2.1878 | 0.0000 | 1.3305 | 0.9541 | 0.7870 | 0.1031 | 1.6904 | 0.0633 | 1.1272 | 0.7769 | 0.3664 | 0.0001 | 1.2474 | 0.3628 | 1.7219 | 0.0233 | 2.9376 | 0.0000 | 0.5248 | 0.0041 | 1.3932 | 0.0237 | 2.0893 | 0.0004 |
| 23 | P09327 | Villin-1 | VIL1 VIL | 4 | 7 | 0.6252 | 0.2815 | 0.4130 | 0.0991 | 0.2992 | 0.0759 | 1.0965 | 0.4819 | 0.3105 | 0.0766 | 0.0946 | 0.0144 | 0.2780 | 0.0733 | 0.4130 | 0.2369 | 0.6982 | 0.3739 | 0.4613 | 0.1009 | 0.4831 | 0.1515 | 0.7727 | 0.3711 | 0.3373 | 0.0926 | 0.1117 | 0.0156 |
| 24 | Q9Y277 | Voltage-dependent anion-selective channel protein 3 (VDAC-3) (hVDAC3) (Outer mitochondrial membrane protein porin 3) | VDAC3 | 13 | 16 | 1.0864 | 0.4063 | 1.5560 | 0.2976 | 0.9462 | 0.8675 | 1.3428 | 0.5757 | 0.7311 | 0.4886 | 1.0568 | 0.6026 | 0.9120 | 0.9297 | 1.5136 | 0.2185 | 1.1912 | 0.3972 | 1.3428 | 0.2665 | 1.2706 | 0.1576 | 0.7379 | 0.5259 | 0.9727 | 0.8099 | 1.1169 | 0.7404 |
| 25 | P21796 | Voltage-dependent anion-selective channel protein 1 (VDAC-1) (hVDAC1) (Outer mitochondrial membrane protein porin 1) (Plasmalemmal porin) (Porin 31HL) (Porin 31HM) | VDAC1 VDAC | 39 | 33 | 1.3804 | 0.3995 | 0.5395 | 0.1066 | 1.2359 | 0.8189 | 0.6918 | 0.1048 | 1.0765 | 0.9712 | 0.8091 | 0.3839 | 1.8197 | 0.1413 | 0.7798 | 0.3944 | 1.9953 | 0.0487 | 0.7047 | 0.4596 | 1.9231 | 0.1899 | 0.8091 | 0.3216 | 0.6081 | 0.3110 | 0.8395 | 0.5615 |
| 26 | P55072 | Transitional endoplasmic reticulum ATPase (TER ATPase) (EC 3.6.4.6) (15S Mg(2+)-ATPase p97 subunit) (Valosin-containing protein) (VCP) | VCP | 52 | 49 | 1.2134 | 0.0137 | 1.0186 | 0.2358 | 1.7061 | 0.0026 | 0.7311 | 0.8730 | 1.9588 | 0.0002 | 1.2023 | 0.2403 | 1.0093 | 0.6012 | 1.3062 | 0.0884 | 0.5297 | 0.5577 | 1.4859 | 0.0213 | 1.9231 | 0.0034 | 1.5417 | 0.0401 | 1.2023 | 0.1288 | 1.3183 | 0.0855 |
| 27 | P18206 | Vinculin (Metavinculin) (MV) | VCL | 60 | 49 | 0.5346 | 0.0141 | 0.6368 | 0.0924 | 1.8707 | 0.0032 | 1.9770 | 0.0033 | 0.7447 | 0.0759 | 1.1169 | 0.6690 | 0.8872 | 0.4315 | 0.7047 | 0.0573 | 1.1912 | 0.5408 | 1.1482 | 0.7944 | 1.0765 | 0.5065 | 0.5754 | 0.0152 | 0.8241 | 0.3351 | 1.2706 | 0.2597 |
| 28 | Q99536 | Synaptic vesicle membrane protein VAT-1 homolog (EC 1.-.-.-) | VAT1 | 8 | 8 | 1.0093 | 0.9607 | 0.5445 | 0.2548 | 1.1482 | 0.4741 | 0.7798 | 0.6958 | 0.9290 | 0.8201 | 0.7379 | 0.5613 | 0.9462 | 0.8562 | 0.6982 | 0.2016 | 1.0000 | 0.9272 | 0.6368 | 0.1560 | 1.0375 | 0.8563 | 0.8166 | 0.1295 | 1.0765 | 0.7327 | 0.7178 | 0.6904 |
| 29 | P26640 | Valine--tRNA ligase (EC 6.1.1.9) (Protein G7a) (Valyl-tRNA synthetase) (ValRS) | VARS G7A VARS2 | 20 | 12 | 1.3305 | 0.5112 | 0.3945 | 0.4094 | 1.4191 | 0.6024 | 1.4322 | 0.4973 | 1.0471 | 0.9999 | 1.6444 | 0.2740 | 1.6293 | 0.3280 | 1.1376 | 0.8837 | 0.7727 | 0.4622 | 0.8954 | 0.8386 | 2.1281 | 0.1397 | 0.5012 | 0.5327 | 1.0765 | 0.7631 | 2.1878 | 0.0241 |
| 30 | Q9P0L0 | Vesicle-associated membrane protein-associated protein A (VAMP-A) (VAMP-associated protein A) (VAP-A) (33 kDa VAMP-associated protein) (VAP-33) | VAPA VAP33 | 5 | 4 | 1.0375 | 0.9879 | 0.5754 | 0.7954 | 0.8091 | 0.6272 | 1.0280 | 0.5217 | 1.0093 | 0.7854 | 1.2474 | 0.5888 | 1.1912 | 0.8343 | 0.9908 | 0.8633 | 0.9376 | 0.9015 | 1.0965 | 0.8825 | 1.0568 | 0.6731 | 1.1803 | 0.5911 | 0.7656 | 0.5434 | 1.4588 | 0.1922 |
| 31 | P46939 | Utrophin (Dystrophin-related protein 1) (DRP-1) | UTRN DMDL DRP1 | 6 | 4 | 1.0765 | 0.5096 | 1.1066 | 0.7989 | 1.1588 | 0.1045 | 0.9462 | 0.8134 | 1.1066 | 0.4239 | 1.1695 | 0.4604 | 1.0471 | 0.7423 | 1.0568 | 0.7313 | 1.0471 | 0.3724 | 0.7943 | 0.2795 | 0.9908 | 0.9884 | 0.9204 | 0.6913 | 1.0666 | 0.7736 | 0.8630 | 0.5161 |
| 32 | Q93008 | Probable ubiquitin carboxyl-terminal hydrolase FAF-X (EC 3.4.19.12) (Deubiquitinating enzyme FAF-X) (Fat facets in mammals) (hFAM) (Fat facets protein-related, X-linked) (Ubiquitin thioesterase FAF-X) (Ubiquitin-specific protease 9, X chromosome) (Ubiquitin-specific-processing protease FAF-X) | USP9X DFFRX FAM USP9 | 3 | 5 | 0.9462 | 0.9848 | 1.0000 | 0.9613 | 0.9727 | 0.9298 | 1.0965 | 0.6446 | 1.1482 | 0.1872 | 0.7516 | 0.6057 | 1.1066 | 0.6026 | 1.1803 | 0.8618 | 1.0093 | 0.8090 | 1.2823 | 0.3907 | 0.9550 | 0.7387 | 0.9290 | 0.8839 | 0.9727 | 0.9388 | 0.8954 | 0.6082 |
| 33 | Q93009 | Ubiquitin carboxyl-terminal hydrolase 7 (EC 3.4.19.12) (Deubiquitinating enzyme 7) (Herpesvirus-associated ubiquitin-specific protease) (Ubiquitin thioesterase 7) (Ubiquitin-specific-processing protease 7) | USP7 HAUSP | 3 | 3 | 1.1588 | 0.4871 | 1.3062 | 0.5840 | 0.6982 | 0.1993 | 0.8630 | 0.7616 | 0.9727 | 0.9734 | 1.0965 | 0.8227 | 1.0280 | 0.8140 | 1.8535 | 0.3392 | 0.6918 | 0.1785 | 1.0186 | 0.9477 | 0.9120 | 0.6238 | 0.7870 | 0.6329 | 1.1066 | 0.5921 | 1.6749 | 0.3861 |
| 34 | P45974 | Ubiquitin carboxyl-terminal hydrolase 5 (EC 3.4.19.12) (Deubiquitinating enzyme 5) (Isopeptidase T) (Ubiquitin thioesterase 5) (Ubiquitin-specific-processing protease 5) | USP5 ISOT | 10 | 17 | 1.0864 | 0.8219 | 2.0137 | 0.0066 | 0.9908 | 0.7380 | 1.2246 | 0.3531 | 1.0000 | 0.8467 | 2.0512 | 0.0433 | 0.8790 | 0.2276 | 2.0701 | 0.0569 | 1.0186 | 0.7620 | 1.9055 | 0.1063 | 0.9376 | 0.5118 | 2.4210 | 0.0343 | 1.0864 | 0.4867 | 2.1478 | 0.0146 |
| 35 | P54578 | Ubiquitin carboxyl-terminal hydrolase 14 (EC 3.4.19.12) (Deubiquitinating enzyme 14) (Ubiquitin thioesterase 14) (Ubiquitin-specific-processing protease 14) | USP14 TGT | 5 | 3 | 0.7112 | 0.2374 | 0.6252 | 0.3809 | 1.0864 | 0.7800 | 0.5395 | 0.2130 | 0.8091 | 0.6033 | 1.1482 | 0.7570 | 1.1482 | 0.4088 | 1.0280 | 0.6099 | 0.5598 | 0.1922 | 1.0000 | 0.9734 | 1.4191 | 0.2668 | 1.0471 | 0.8050 | 0.9727 | 0.9387 | 0.7379 | 0.7481 |
| 36 | O60763 | General vesicular transport factor p115 (Protein USO1 homolog) (Transcytosis-associated protein) (TAP) (Vesicle-docking protein) | USO1 VDP | 17 | 16 | 1.4859 | 0.2859 | 1.8880 | 0.1411 | 1.4588 | 0.0912 | 0.9204 | 0.8812 | 2.0512 | 0.0787 | 1.5560 | 0.3434 | 2.1086 | 0.1548 | 1.2589 | 0.4627 | 1.6144 | 0.1084 | 1.6293 | 0.3437 | 2.3550 | 0.0086 | 0.8395 | 0.7709 | 1.7378 | 0.0566 | 2.3335 | 0.0242 |
| 37 | Q96N76 | Urocanate hydratase (Urocanase) (EC 4.2.1.49) (Imidazolonepropionate hydrolase) | UROC1 | 23 | 24 | 0.9908 | 0.6756 | 1.5276 | 0.0092 | 1.7219 | 0.2061 | 1.4859 | 0.1946 | 1.9953 | 0.0447 | 0.5702 | 0.2903 | 2.4660 | 0.0014 | 1.0375 | 0.1477 | 2.6062 | 0.0070 | 0.8630 | 0.6606 | 0.4246 | 0.2420 | 0.2729 | 0.0528 | 1.1588 | 0.8069 | 0.8551 | 0.5345 |
| 38 | P07919 | Cytochrome b-c1 complex subunit 6, mitochondrial (Complex III subunit 6) (Complex III subunit VIII) (Cytochrome c1 non-heme 11 kDa protein) (Mitochondrial hinge protein) (Ubiquinol-cytochrome c reductase complex 11 kDa protein) | UQCRH | 9 | 7 | 0.5105 | 0.5372 | 1.1272 | 0.4415 | 0.7112 | 0.3392 | 1.2246 | 0.2486 | 0.2858 | 0.2962 | 1.0093 | 0.9995 | 0.6252 | 0.3649 | 1.0864 | 0.7574 | 0.9462 | 0.9752 | 1.1066 | 0.4981 | 0.6138 | 0.2291 | 1.1376 | 0.3879 | 0.2109 | 0.1640 | 1.0375 | 0.8178 |
| 39 | P47985 | Cytochrome b-c1 complex subunit Rieske, mitochondrial (EC 1.10.2.2) (Complex III subunit 5) (Cytochrome b-c1 complex subunit 5) (Rieske iron-sulfur protein) (RISP) (Ubiquinol-cytochrome c reductase iron-sulfur subunit) [Cleaved into: Cytochrome b-c1 complex subunit 11 (Complex III subunit IX) (Ubiquinol-cytochrome c reductase 8 kDa protein)] | UQCRFS1 | 10 | 13 | 1.3804 | 0.4175 | 1.0280 | 0.5111 | 1.1695 | 0.5465 | 0.7727 | 0.4488 | 0.6194 | 0.1441 | 0.6982 | 0.2356 | 1.6596 | 0.2345 | 0.8790 | 0.5745 | 1.1695 | 0.9177 | 0.9908 | 0.7427 | 0.5916 | 0.1826 | 0.5808 | 0.0481 | 1.1376 | 0.6093 | 0.9638 | 0.9970 |
| 40 | P22695 | Cytochrome b-c1 complex subunit 2, mitochondrial (Complex III subunit 2) (Core protein II) (Ubiquinol-cytochrome-c reductase complex core protein 2) | UQCRC2 | 35 | 31 | 0.7447 | 0.5325 | 0.9550 | 0.8674 | 1.1169 | 0.9901 | 0.9550 | 0.8316 | 0.5395 | 0.2066 | 0.9036 | 0.5037 | 1.0568 | 0.6165 | 0.9290 | 0.8941 | 0.9550 | 0.6195 | 0.9376 | 0.9665 | 0.7047 | 0.2576 | 0.9036 | 0.7108 | 0.7047 | 0.2069 | 0.9036 | 0.5954 |
| 41 | P31930 | Cytochrome b-c1 complex subunit 1, mitochondrial (Complex III subunit 1) (Core protein I) (Ubiquinol-cytochrome-c reductase complex core protein 1) | UQCRC1 | 30 | 30 | 1.3932 | 0.6922 | 1.1482 | 0.8294 | 1.2474 | 0.9423 | 1.5560 | 0.1175 | 0.6310 | 0.2313 | 1.7378 | 0.3856 | 1.7865 | 0.7899 | 1.4859 | 0.4120 | 1.6144 | 0.8156 | 1.2823 | 0.6783 | 0.3873 | 0.0183 | 1.7219 | 0.5304 | 0.4966 | 0.1603 | 1.4588 | 0.3949 |
| 42 | P14927 | Cytochrome b-c1 complex subunit 7 (Complex III subunit 7) (Complex III subunit VII) (QP-C) (Ubiquinol-cytochrome c reductase complex 14 kDa protein) | UQCRB UQBP | 6 | 6 | 1.4191 | 0.8659 | 0.7178 | 0.3660 | 1.0093 | 0.2512 | 0.6607 | 0.1004 | 0.6792 | 0.2426 | 0.5012 | 0.0442 | 1.2706 | 0.9751 | 0.4365 | 0.0258 | 1.1588 | 0.6124 | 0.6982 | 0.1011 | 0.7870 | 0.1312 | 0.7656 | 0.2024 | 1.2023 | 0.5390 | 0.6252 | 0.0526 |
| 43 | Q92900 | Regulator of nonsense transcripts 1 (EC 3.6.4.-) (ATP-dependent helicase RENT1) (Nonsense mRNA reducing factor 1) (NORF1) (Up-frameshift suppressor 1 homolog) (hUpf1) | UPF1 KIAA0221 RENT1 | 7 | 3 | 1.0568 | 0.6161 | 1.2134 | 0.7606 | 0.9727 | 0.7923 | 1.6904 | 0.0922 | 1.0965 | 0.6506 | 0.9817 | 0.4200 | 1.0568 | 0.4029 | 1.1588 | 0.5349 | 1.0093 | 0.9308 | 1.7061 | 0.3232 | 1.0375 | 0.7263 | 1.4588 | 0.4067 | 1.0666 | 0.6070 | 0.8790 | 0.9599 |
| 44 | Q9UBR1 | Beta-ureidopropionase (EC 3.5.1.6) (BUP-1) (Beta-alanine synthase) (N-carbamoyl-beta-alanine amidohydrolase) | UPB1 BUP1 | 29 | 28 | 0.6792 | 0.0393 | 0.8241 | 0.3410 | 0.7244 | 0.0899 | 1.0000 | 0.8814 | 1.1272 | 0.8164 | 0.8395 | 0.2119 | 0.5248 | 0.0019 | 1.3305 | 0.5847 | 0.5702 | 0.0009 | 0.4920 | 0.0313 | 0.1660 | 0.0000 | 0.9036 | 0.9429 | 0.2535 | 0.0000 | 0.1259 | 0.0000 |
| 45 | P11172 | Uridine 5'-monophosphate synthase (UMP synthase) [Includes: Orotate phosphoribosyltransferase (OPRT) (OPRTase) (EC 2.4.2.10); Orotidine 5'-phosphate decarboxylase (ODC) (EC 4.1.1.23) (OMPdecase)] | UMPS OK/SW-cl.21 | 7 | 3 | 0.9727 | 0.8883 | 1.2246 | 0.5490 | 0.9817 | 0.8879 | 1.0375 | 0.9203 | 1.1376 | 0.5797 | 0.9727 | 0.7242 | 1.0186 | 0.8052 | 1.0471 | 0.8681 | 0.9550 | 0.9160 | 1.1376 | 0.8083 | 1.1376 | 0.6162 | 1.0765 | 0.8523 | 1.4060 | 0.2088 | 1.2359 | 0.5885 |
| 46 | P16662 | UDP-glucuronosyltransferase 2B7 (UDPGT 2B7) (EC 2.4.1.17) (3,4-catechol estrogen-specific UDPGT) (UDP-glucuronosyltransferase 2B9) (UDPGT 2B9) (UDPGTh-2) | UGT2B7 UGTB2B9 | 41 | 36 | 2.8054 | 0.0105 | 3.2211 | 0.0013 | 0.9376 | 0.5539 | 1.7701 | 0.4278 | 2.0324 | 0.2320 | 0.6368 | 0.2072 | 1.7061 | 0.3915 | 2.1478 | 0.1211 | 2.0324 | 0.1207 | 1.5136 | 0.0808 | 0.4246 | 0.0826 | 0.4169 | 0.0733 | 1.2359 | 0.6746 | 0.7943 | 0.1437 |
| 47 | P06133 | UDP-glucuronosyltransferase 2B4 (UDPGT 2B4) (EC 2.4.1.17) (HLUG25) (Hyodeoxycholic acid-specific UDPGT) (UDPGTh-1) | UGT2B4 UGT2B11 | 30 | 30 | 2.2491 | 0.0103 | 1.4060 | 0.2480 | 1.6596 | 0.5671 | 0.8710 | 0.6209 | 0.9908 | 0.8346 | 1.4191 | 0.4691 | 1.1912 | 0.4150 | 1.8880 | 0.2192 | 1.6904 | 0.0431 | 1.7865 | 0.0914 | 0.7943 | 0.3237 | 0.7379 | 0.6014 | 1.4859 | 0.2064 | 0.7586 | 0.5351 |
| 48 | P54855 | UDP-glucuronosyltransferase 2B15 (UDPGT 2B15) (EC 2.4.1.17) (HLUG4) (UDP-glucuronosyltransferase 2B8) (UDPGT 2B8) (UDPGTh-3) | UGT2B15 UGT2B8 | 20 | 18 | 3.5318 | 0.1296 | 6.2517 | 0.0257 | 1.0864 | 0.8637 | 4.9204 | 0.0192 | 1.6144 | 0.7862 | 0.8872 | 0.8985 | 0.8091 | 0.4357 | 6.3096 | 0.0178 | 1.5996 | 0.3561 | 1.5996 | 0.2023 | 0.8091 | 0.7221 | 1.2823 | 0.9856 | 1.7061 | 0.5796 | 3.4041 | 0.0376 |
| 49 | P36537 | UDP-glucuronosyltransferase 2B10 (UDPGT 2B10) (EC 2.4.1.17) | UGT2B10 | 20 | 20 | 0.4571 | 0.4798 | 1.3183 | 0.5427 | 0.3467 | 0.4264 | 0.3698 | 0.0117 | 1.3932 | 0.8947 | 0.3908 | 0.0172 | 0.9204 | 0.8924 | 1.2706 | 0.9742 | 0.9376 | 0.8241 | 1.1695 | 0.7178 | 0.2466 | 0.3489 | 1.0280 | 0.5442 | 0.5346 | 0.4989 | 1.1169 | 0.8365 |
| 50 | Q6UWM9 | UDP-glucuronosyltransferase 2A3 (UDPGT 2A3) (EC 2.4.1.17) | UGT2A3 UNQ2559/PRO6239 | 7 | 8 | 1.0666 | 0.8324 | 0.9462 | 0.7287 | 0.8091 | 0.6100 | 1.3183 | 0.8151 | 0.3664 | 0.4394 | 1.3428 | 0.6873 | 0.9204 | 0.7196 | 1.9231 | 0.4351 | 0.7447 | 0.5881 | 1.2589 | 0.8362 | 1.5136 | 0.4510 | 1.7219 | 0.2971 | 0.7798 | 0.7581 | 2.6792 | 0.1164 |
| 51 | O60656 | UDP-glucuronosyltransferase 1-9 (UDPGT 1-9) (UGT1*9) (UGT1-09) (UGT1.9) (EC 2.4.1.17) (UDP-glucuronosyltransferase 1-I) (UGT-1I) (UGT1I) (UDP-glucuronosyltransferase 1A9) (lugP4) | UGT1A9 GNT1 UGT1 | 27 | 20 | 1.2359 | 0.4025 | 1.0864 | 0.5685 | 1.1066 | 0.9190 | 2.0701 | 0.3957 | 0.7244 | 0.4867 | 1.7378 | 0.4454 | 0.7244 | 0.4655 | 1.3677 | 0.5180 | 1.1803 | 0.3647 | 1.6749 | 0.3660 | 1.3183 | 0.3588 | 1.5704 | 0.4490 | 0.7943 | 0.9377 | 1.9409 | 0.3858 |
| 52 | P19224 | UDP-glucuronosyltransferase 1-6 (UDPGT 1-6) (UGT1*6) (UGT1-06) (UGT1.6) (EC 2.4.1.17) (Phenol-metabolizing UDP-glucuronosyltransferase) (UDP-glucuronosyltransferase 1-F) (UGT-1F) (UGT1F) (UDP-glucuronosyltransferase 1A6) | UGT1A6 GNT1 UGT1 | 24 | 19 | 1.2359 | 0.5301 | 0.5200 | 0.5582 | 0.4699 | 0.4136 | 0.2466 | 0.0900 | 0.6194 | 0.4460 | 0.9908 | 0.9791 | 0.1905 | 0.0358 | 1.5417 | 0.2801 | 0.7047 | 0.7660 | 0.7870 | 0.4919 | 1.1376 | 0.2556 | 0.9036 | 0.9060 | 1.1695 | 0.7577 | 0.6730 | 0.9725 |
| 53 | P22310 | UDP-glucuronosyltransferase 1-4 (UDPGT 1-4) (UGT1*4) (UGT1-04) (UGT1.4) (EC 2.4.1.17) (Bilirubin-specific UDPGT isozyme 2) (hUG-BR2) (UDP-glucuronosyltransferase 1-D) (UGT-1D) (UGT1D) (UDP-glucuronosyltransferase 1A4) | UGT1A4 GNT1 UGT1 | 31 | 22 | 2.1086 | 0.0121 | 0.2512 | 0.0064 | 1.6444 | 0.0255 | 1.6444 | 0.1343 | 1.7539 | 0.0935 | 0.5395 | 0.0456 | 2.1478 | 0.0147 | 0.9376 | 0.9931 | 1.3804 | 0.0724 | 1.0471 | 0.5100 | 1.2359 | 0.4094 | 0.4613 | 0.0469 | 1.1695 | 0.8690 | 0.1225 | 0.0025 |
| 54 | P22309 | UDP-glucuronosyltransferase 1-1 (UDPGT 1-1) (UGT1*1) (UGT1-01) (UGT1.1) (EC 2.4.1.17) (Bilirubin-specific UDPGT isozyme 1) (hUG-BR1) (UDP-glucuronosyltransferase 1-A) (UGT-1A) (UGT1A) (UDP-glucuronosyltransferase 1A1) | UGT1A1 GNT1 UGT1 | 37 | 33 | 2.5823 | 0.0405 | 0.5346 | 0.7395 | 2.4889 | 0.0704 | 3.6308 | 0.3344 | 1.2474 | 0.2593 | 8.4723 | 0.0102 | 0.4487 | 0.1869 | 3.4995 | 0.1985 | 2.6303 | 0.0228 | 4.4055 | 0.0399 | 0.9036 | 0.9059 | 1.8197 | 0.7170 | 2.9923 | 0.0216 | 5.4450 | 0.0264 |
| 55 | Q16851 | UTP--glucose-1-phosphate uridylyltransferase (EC 2.7.7.9) (UDP-glucose pyrophosphorylase) (UDPGP) (UGPase) | UGP2 UGP1 | 122 | 122 | 0.8017 | 0.0190 | 0.7870 | 0.5285 | 0.8710 | 0.2759 | 0.4055 | 0.0073 | 0.8395 | 0.0830 | 1.7865 | 0.0336 | 1.1803 | 0.8088 | 1.2942 | 0.4855 | 1.0000 | 0.6256 | 1.5417 | 0.1578 | 0.0964 | 0.0000 | 1.7701 | 0.0013 | 0.1959 | 0.0000 | 1.2589 | 0.2295 |
| 56 | Q9NYU2 | UDP-glucose:glycoprotein glucosyltransferase 1 (UGT1) (hUGT1) (EC 2.4.1.-) (UDP--Glc:glycoprotein glucosyltransferase) (UDP-glucose ceramide glucosyltransferase-like 1) | UGGT1 GT UGCGL1 UGGT UGT1 UGTR | 25 | 27 | 1.2246 | 0.8305 | 1.0186 | 0.7838 | 1.0000 | 0.6002 | 1.3428 | 0.8099 | 0.7379 | 0.1745 | 0.4613 | 0.1553 | 1.6904 | 0.0583 | 0.6792 | 0.2729 | 0.8551 | 0.3137 | 0.6546 | 0.2607 | 1.0965 | 0.8487 | 0.6368 | 0.4644 | 0.9638 | 0.4908 | 0.8395 | 0.4932 |
| 57 | O60701 | UDP-glucose 6-dehydrogenase (UDP-Glc dehydrogenase) (UDP-GlcDH) (UDPGDH) (EC 1.1.1.22) | UGDH | 45 | 39 | 0.8091 | 0.0330 | 0.5598 | 0.0688 | 2.0512 | 0.1936 | 1.2134 | 0.7229 | 1.0093 | 0.1542 | 1.5276 | 0.4968 | 1.6444 | 0.8473 | 1.4191 | 0.6385 | 0.7943 | 0.0189 | 1.0093 | 0.4859 | 0.5970 | 0.0003 | 3.7670 | 0.0002 | 1.1803 | 0.1098 | 1.9055 | 0.2280 |
| 58 | Q92575 | UBX domain-containing protein 4 (Erasin) (UBX domain-containing protein 2) | UBXN4 KIAA0242 UBXD2 UBXDC1 | 4 | 4 | 0.8630 | 0.7471 | 1.2134 | 0.7869 | 1.0093 | 0.4329 | 0.7586 | 0.3924 | 1.0375 | 0.8961 | 1.2246 | 0.4254 | 1.0965 | 0.4130 | 1.8707 | 0.4198 | 1.0765 | 0.4389 | 1.6904 | 0.3331 | 0.9638 | 0.8311 | 1.4723 | 0.4834 | 0.9817 | 0.7498 | 1.3183 | 0.8048 |
| 59 | Q13404 | Ubiquitin-conjugating enzyme E2 variant 1 (UEV-1) (CROC-1) (TRAF6-regulated IKK activator 1 beta Uev1A) | UBE2V1 CROC1 UBE2V UEV1 P/OKcl.19 | 11 | 12 | 0.5754 | 0.1338 | 0.6855 | 0.5096 | 0.8017 | 0.4501 | 0.8872 | 0.8035 | 0.8790 | 0.9873 | 0.8395 | 0.7211 | 0.5702 | 0.2484 | 0.8551 | 0.7273 | 0.8954 | 0.6547 | 0.8395 | 0.7486 | 0.4406 | 0.1057 | 0.8318 | 0.7242 | 0.4571 | 0.0435 | 0.8166 | 0.6472 |
| 60 | P61088 | Ubiquitin-conjugating enzyme E2 N (EC 2.3.2.23) (Bendless-like ubiquitin-conjugating enzyme) (E2 ubiquitin-conjugating enzyme N) (Ubc13) (UbcH13) (Ubiquitin carrier protein N) (Ubiquitin-protein ligase N) | UBE2N BLU | 6 | 7 | 0.9727 | 0.8338 | 1.4859 | 0.5486 | 0.9204 | 0.6337 | 1.0568 | 0.7031 | 1.1482 | 0.2031 | 0.8166 | 0.7982 | 0.8790 | 0.5960 | 0.8091 | 0.9085 | 1.0471 | 0.8364 | 1.0666 | 0.4997 | 0.9462 | 0.5178 | 1.0568 | 0.3564 | 0.9908 | 0.8958 | 1.1588 | 0.5985 |
| 61 | P68036 | Ubiquitin-conjugating enzyme E2 L3 (EC 2.3.2.23) (E2 ubiquitin-conjugating enzyme L3) (L-UBC) (UbcH7) (Ubiquitin carrier protein L3) (Ubiquitin-conjugating enzyme E2-F1) (Ubiquitin-protein ligase L3) | UBE2L3 UBCE7 UBCH7 | 11 | 6 | 0.6607 | 0.4074 | 0.9290 | 0.9518 | 1.0093 | 0.8021 | 0.9550 | 0.3912 | 1.4322 | 0.5437 | 0.9817 | 0.8120 | 1.5276 | 0.1889 | 0.9204 | 0.2762 | 0.8091 | 0.4192 | 0.8710 | 0.2471 | 1.2134 | 0.8625 | 0.9817 | 0.9054 | 0.8954 | 0.9933 | 0.8472 | 0.0635 |
| 62 | P61086 | Ubiquitin-conjugating enzyme E2 K (EC 2.3.2.23) (E2 ubiquitin-conjugating enzyme K) (Huntingtin-interacting protein 2) (HIP-2) (Ubiquitin carrier protein) (Ubiquitin-conjugating enzyme E2-25 kDa) (Ubiquitin-conjugating enzyme E2(25K)) (Ubiquitin-conjugating enzyme E2-25K) (Ubiquitin-protein ligase) | UBE2K HIP2 LIG | 4 | 6 | 0.9727 | 0.7463 | 0.9290 | 0.9901 | 0.4920 | 0.1369 | 0.8472 | 0.2056 | 0.4018 | 0.2263 | 0.9638 | 0.8934 | 1.1482 | 0.8046 | 0.8872 | 0.6401 | 0.7727 | 0.2302 | 0.8954 | 0.7665 | 0.8630 | 0.5573 | 0.9036 | 0.6668 | 0.7178 | 0.2138 | 0.9376 | 0.4920 |
| 63 | P41226 | Ubiquitin-like modifier-activating enzyme 7 (Ubiquitin-activating enzyme 7) (D8) (Ubiquitin-activating enzyme E1 homolog) | UBA7 UBE1L UBE2 | 3 | 7 | 0.9376 | 0.7865 | 1.4060 | 0.4253 | 1.4454 | 0.2363 | 0.9550 | 0.5379 | 1.8197 | 0.1203 | 5.9704 | 0.1969 | 1.5136 | 0.2061 | 2.1281 | 0.2710 | 1.4322 | 0.2562 | 2.3768 | 0.3542 | 1.6596 | 0.1534 | 2.4889 | 0.3341 | 1.7219 | 0.1388 | 5.6494 | 0.2266 |
| 64 | P22314 | Ubiquitin-like modifier-activating enzyme 1 (Protein A1S9) (Ubiquitin-activating enzyme E1) | UBA1 A1S9T UBE1 | 51 | 47 | 0.6310 | 0.1178 | 0.6368 | 0.3246 | 1.4322 | 0.3147 | 0.6607 | 0.0402 | 1.2023 | 0.8059 | 1.2823 | 0.4816 | 0.9817 | 0.2092 | 0.8395 | 0.1261 | 0.7244 | 0.0808 | 1.2942 | 0.4494 | 1.0000 | 0.6275 | 1.7539 | 0.1696 | 0.7311 | 0.0188 | 0.9036 | 0.2699 |
| 65 | Q16222 | UDP-N-acetylhexosamine pyrophosphorylase (Antigen X) (AGX) (Sperm-associated antigen 2) [Includes: UDP-N-acetylgalactosamine pyrophosphorylase (EC 2.7.7.83) (AGX-1); UDP-N-acetylglucosamine pyrophosphorylase (EC 2.7.7.23) (AGX-2)] | UAP1 SPAG2 | 6 | 8 | 1.5704 | 0.5488 | 0.9550 | 0.8441 | 0.7112 | 0.3106 | 0.9462 | 0.8741 | 0.9727 | 0.3496 | 0.9204 | 0.7433 | 0.8017 | 0.3866 | 1.2023 | 0.4720 | 0.6194 | 0.1561 | 1.0765 | 0.4828 | 1.0471 | 0.3263 | 1.0765 | 0.6815 | 0.5808 | 0.2095 | 1.0965 | 0.4040 |
| 66 | P19971 | Thymidine phosphorylase (TP) (EC 2.4.2.4) (Gliostatin) (Platelet-derived endothelial cell growth factor) (PD-ECGF) (TdRPase) | TYMP ECGF1 | 29 | 32 | 1.4322 | 0.7796 | 3.1046 | 0.2261 | 1.5996 | 0.4257 | 0.9376 | 0.4660 | 1.8880 | 0.1447 | 6.9823 | 0.0240 | 1.9231 | 0.0891 | 0.6982 | 0.8666 | 1.9055 | 0.1903 | 3.5318 | 0.1300 | 3.0479 | 0.0005 | 2.7040 | 0.2074 | 4.0551 | 0.0000 | 4.5290 | 0.0858 |
| 67 | O43396 | Thioredoxin-like protein 1 (32 kDa thioredoxin-related protein) | TXNL1 TRP32 TXL TXNL | 5 | 6 | 0.5200 | 0.3857 | 1.8707 | 0.0642 | 1.1272 | 0.1950 | 1.1588 | 0.4623 | 1.2134 | 0.1856 | 2.9376 | 0.0043 | 1.3305 | 0.5174 | 2.5351 | 0.0100 | 1.4454 | 0.1873 | 1.5849 | 0.2309 | 1.4454 | 0.1792 | 1.5276 | 0.1679 | 1.5136 | 0.4023 | 2.1281 | 0.0697 |
| 68 | Q8NBS9 | Thioredoxin domain-containing protein 5 (Endoplasmic reticulum resident protein 46) (ER protein 46) (ERp46) (Thioredoxin-like protein p46) | TXNDC5 TLP46 UNQ364/PRO700 | 16 | 17 | 0.4699 | 0.0114 | 0.5346 | 0.0238 | 1.1803 | 0.6320 | 0.4831 | 0.0240 | 1.0765 | 0.8170 | 0.5297 | 0.0510 | 0.8091 | 0.1733 | 0.2729 | 0.0021 | 0.8872 | 0.6212 | 1.0568 | 0.7700 | 1.3552 | 0.3554 | 0.8318 | 0.6640 | 1.1588 | 0.7687 | 1.2134 | 0.3180 |
| 69 | Q9BRA2 | Thioredoxin domain-containing protein 17 (14 kDa thioredoxin-related protein) (TRP14) (Protein 42-9-9) (Thioredoxin-like protein 5) | TXNDC17 TXNL5 | 4 | 5 | 0.7112 | 0.6289 | 0.9036 | 0.9249 | 1.0186 | 0.5627 | 1.0186 | 0.9502 | 1.2246 | 0.5832 | 2.1086 | 0.3912 | 1.7539 | 0.3893 | 1.5560 | 0.5815 | 0.8630 | 0.7842 | 1.6904 | 0.4134 | 1.0186 | 0.9685 | 1.1482 | 0.6775 | 0.8790 | 0.8512 | 0.9550 | 0.8994 |
| 70 | O95881 | Thioredoxin domain-containing protein 12 (EC 1.8.4.2) (Endoplasmic reticulum resident protein 18) (ER protein 18) (ERp18) (Endoplasmic reticulum resident protein 19) (ER protein 19) (ERp19) (Thioredoxin-like protein p19) (hTLP19) | TXNDC12 TLP19 UNQ713/PRO1376 | 5 | 3 | 0.9376 | 0.7762 | 0.8091 | 0.4887 | 1.0965 | 0.9615 | 0.8318 | 0.5439 | 1.2023 | 0.8960 | 0.8241 | 0.5157 | 1.0864 | 0.9402 | 0.6252 | 0.1921 | 1.4322 | 0.6699 | 0.6918 | 0.2780 | 1.5417 | 0.5946 | 0.8395 | 0.5683 | 1.2474 | 0.8007 | 0.7870 | 0.4392 |
| 71 | P10599 | Thioredoxin (Trx) (ATL-derived factor) (ADF) (Surface-associated sulphydryl protein) (SASP) | TXN TRDX TRX TRX1 | 15 | 5 | 0.2679 | 0.0059 | 0.2512 | 0.0842 | 0.6310 | 0.1587 | 0.4446 | 0.0763 | 0.4966 | 0.3940 | 0.8551 | 0.5879 | 0.7178 | 0.3478 | 0.4018 | 0.1075 | 0.5916 | 0.1685 | 0.5808 | 0.1412 | 1.2823 | 0.5389 | 0.8395 | 0.7497 | 0.9120 | 0.9113 | 0.6855 | 0.9834 |
| 72 | Q6IBS0 | Twinfilin-2 (A6-related protein) (hA6RP) (Protein tyrosine kinase 9-like) (Twinfilin-1-like protein) | TWF2 PTK9L MSTP011 | 4 | 8 | 0.9550 | 0.9995 | 1.0280 | 0.8431 | 0.5702 | 0.1154 | 1.2246 | 0.7073 | 0.8241 | 0.9606 | 0.8710 | 0.3884 | 0.8241 | 0.4698 | 1.0864 | 0.7498 | 0.8630 | 0.7614 | 1.1066 | 0.4667 | 0.9376 | 0.7778 | 1.2023 | 0.4831 | 0.9462 | 0.7298 | 1.1695 | 0.6166 |
| 73 | Q12792 | Twinfilin-1 (Protein A6) (Protein tyrosine kinase 9) | TWF1 PTK9 | 3 | 3 | 1.1376 | 0.8942 | 1.2023 | 0.4597 | 0.8318 | 0.5707 | 0.8472 | 0.4132 | 1.0093 | 0.8945 | 1.0471 | 0.8927 | 1.2023 | 0.9655 | 1.3932 | 0.2496 | 1.0965 | 0.7814 | 1.0000 | 1.0000 | 1.0000 | 0.9113 | 1.1376 | 0.5693 | 0.9817 | 0.7674 | 1.1376 | 0.6039 |
| 74 | P49411 | Elongation factor Tu, mitochondrial (EF-Tu) (P43) | TUFM | 36 | 30 | 1.5560 | 0.0873 | 1.4060 | 0.2821 | 1.0000 | 0.8754 | 1.3428 | 0.6438 | 1.0568 | 0.5124 | 0.7379 | 0.0631 | 1.9231 | 0.0517 | 1.2246 | 0.5722 | 1.7219 | 0.0428 | 1.1169 | 0.6748 | 0.5395 | 0.0706 | 1.6596 | 0.1015 | 1.1482 | 0.9720 | 1.2589 | 0.8116 |
| 75 | Q9BUF5 | Tubulin beta-6 chain (Tubulin beta class V) | TUBB6 | 51 | 31 | 2.1086 | 0.3236 | 0.3467 | 0.2276 | 1.7701 | 0.3997 | 0.6486 | 0.4746 | 2.0512 | 0.3355 | 0.6081 | 0.4275 | 1.1272 | 0.7451 | 0.4207 | 0.2733 | 1.3428 | 0.6990 | 0.5395 | 0.3635 | 1.7219 | 0.4105 | 0.5105 | 0.3385 | 1.1482 | 0.9395 | 0.4742 | 0.3094 |
| 76 | P68371 | Tubulin beta-4B chain (Tubulin beta-2 chain) (Tubulin beta-2C chain) | TUBB4B TUBB2C | 82 | 70 | 2.2491 | 0.6416 | 1.5560 | 0.7252 | 2.3988 | 0.4636 | 1.0186 | 0.9679 | 1.7219 | 0.4151 | 2.3550 | 0.3767 | 2.3335 | 0.5614 | 2.7040 | 0.4912 | 1.9055 | 0.5067 | 3.1333 | 0.3061 | 2.6792 | 0.4047 | 2.9107 | 0.3339 | 2.6062 | 0.4399 | 3.4995 | 0.2139 |
| 77 | Q13885 | Tubulin beta-2A chain (Tubulin beta class IIa) | TUBB2A TUBB2 | 78 | 62 | 1.0864 | 0.9814 | 1.5560 | 0.4625 | 1.0186 | 0.8191 | 0.9204 | 0.9409 | 2.0512 | 0.2532 | 2.0893 | 0.3001 | 1.2706 | 0.5579 | 2.3768 | 0.1722 | 1.2942 | 0.7668 | 2.6792 | 0.1405 | 1.0375 | 0.7596 | 2.7542 | 0.1312 | 0.7943 | 0.6406 | 2.5351 | 0.1754 |
| 78 | P68366 | Tubulin alpha-4A chain (Alpha-tubulin 1) (Testis-specific alpha-tubulin) (Tubulin H2-alpha) (Tubulin alpha-1 chain) | TUBA4A TUBA1 | 54 | 58 | 2.9376 | 0.2840 | 2.6062 | 0.6668 | 1.0280 | 0.7689 | 2.6546 | 0.4758 | 0.9204 | 0.9303 | 1.2589 | 0.6283 | 3.8371 | 0.1538 | 1.7701 | 0.8743 | 3.1046 | 0.1948 | 1.1803 | 0.7802 | 0.5105 | 0.3757 | 2.0893 | 0.8681 | 0.6138 | 0.2196 | 1.3552 | 0.8004 |
| 79 | Q9BQE3 | Tubulin alpha-1C chain (Alpha-tubulin 6) (Tubulin alpha-6 chain) | TUBA1C TUBA6 | 55 | 54 | 0.9036 | 0.5406 | 0.8395 | 0.7875 | 0.7943 | 0.2759 | 0.9376 | 0.9636 | 0.8630 | 0.4214 | 0.8710 | 0.7899 | 1.0000 | 0.6250 | 1.0280 | 0.7723 | 0.7516 | 0.1989 | 0.9204 | 0.8939 | 1.0000 | 0.8403 | 1.1169 | 0.7329 | 0.8790 | 0.4997 | 0.9120 | 0.8989 |
| 80 | P68363 | Tubulin alpha-1B chain (Alpha-tubulin ubiquitous) (Tubulin K-alpha-1) (Tubulin alpha-ubiquitous chain) | TUBA1B | 62 | 62 |  |  |  |  |  |  |  |  |  |  |  |  |  |  |  |  |  |  |  |  |  |  |  |  |  |  |  |  |
| 81 | Q71U36 | Tubulin alpha-1A chain (Alpha-tubulin 3) (Tubulin B-alpha-1) (Tubulin alpha-3 chain) | TUBA1A TUBA3 | 56 | 60 | 0.8551 | 0.8007 | 1.5996 | 0.4326 | 1.2474 | 0.5533 | 1.6444 | 0.3834 | 1.5849 | 0.3683 | 1.3305 | 0.5964 | 1.1588 | 0.7963 | 2.3335 | 0.2724 | 1.0280 | 0.9106 | 0.7447 | 0.8423 | 0.9036 | 0.8459 | 0.6252 | 0.6207 | 1.0000 | 0.9396 | 1.0375 | 0.5984 |
| 82 | P49638 | Alpha-tocopherol transfer protein (Alpha-TTP) | TTPA TPP1 | 6 | 5 | 0.7311 | 0.8201 | 2.2080 | 0.2081 | 0.8318 | 0.7446 | 2.8054 | 0.0982 | 0.5916 | 0.0566 | 1.1066 | 0.4359 | 1.1066 | 0.5109 | 1.8197 | 0.3468 | 0.6668 | 0.1005 | 2.2699 | 0.2538 | 0.5012 | 0.3254 | 2.0701 | 0.2946 | 0.9908 | 0.8961 | 1.1803 | 0.6956 |
| 83 | Q14166 | Tubulin--tyrosine ligase-like protein 12 | TTLL12 KIAA0153 | 4 | 7 | 0.8091 | 0.6357 | 0.5395 | 0.3274 | 0.7943 | 0.7312 | 0.8872 | 0.6085 | 0.5105 | 0.4515 | 0.7516 | 0.7418 | 0.2070 | 0.2442 | 0.7112 | 0.9447 | 1.6293 | 0.4394 | 0.5200 | 0.2548 | 1.3062 | 0.5372 | 1.0568 | 0.9316 | 1.1588 | 0.9460 | 1.0186 | 0.8050 |
| 84 | Q5R3I4 | Tetratricopeptide repeat protein 38 (TPR repeat protein 38) | TTC38 | 14 | 13 | 0.9908 | 0.6334 | 0.6730 | 0.0311 | 1.0186 | 0.9920 | 1.1272 | 0.3501 | 0.7798 | 0.1121 | 0.7516 | 0.0544 | 1.3183 | 0.4819 | 1.0765 | 0.4180 | 0.9550 | 0.5908 | 0.8472 | 0.0666 | 0.3733 | 0.0058 | 1.0093 | 0.2213 | 0.9462 | 0.5367 | 0.7047 | 0.0322 |
| 85 | A6NLP5 | Tetratricopeptide repeat protein 36 (TPR repeat protein 36) (HSP70-binding protein 21) | TTC36 HBP21 | 9 | 7 | 2.7797 | 0.1599 | 3.6308 | 0.1760 | 2.5823 | 0.1055 | 2.1478 | 0.4591 | 2.8840 | 0.1202 | 1.7865 | 0.5887 | 2.1281 | 0.4208 | 3.7670 | 0.1535 | 1.1803 | 0.6981 | 0.7244 | 0.9874 | 0.7112 | 0.2576 | 4.6559 | 0.0460 | 1.0280 | 0.4758 | 0.7870 | 0.9958 |
| 86 | Q13630 | GDP-L-fucose synthase (EC 1.1.1.271) (GDP-4-keto-6-deoxy-D-mannose-3,5-epimerase-4-reductase) (Protein FX) (Red cell NADP(H)-binding protein) (Short-chain dehydrogenase/reductase family 4E member 1) | TSTA3 SDR4E1 | 6 | 5 | 0.5808 | 0.4769 | 0.6026 | 0.3622 | 0.8710 | 0.5758 | 0.4207 | 0.1111 | 0.3251 | 0.1113 | 0.8395 | 0.5307 | 0.7943 | 0.8796 | 0.3311 | 0.1165 | 0.6486 | 0.4307 | 0.4487 | 0.1727 | 0.6982 | 0.6398 | 0.5346 | 0.4951 | 0.9036 | 0.9353 | 1.0000 | 0.7031 |
| 87 | Q16762 | Thiosulfate sulfurtransferase (EC 2.8.1.1) (Rhodanese) | TST | 42 | 38 | 0.9817 | 0.8984 | 1.9055 | 0.0405 | 0.9120 | 0.3090 | 1.1695 | 0.5329 | 0.8017 | 0.8567 | 1.8030 | 0.0450 | 1.1066 | 0.2796 | 1.4588 | 0.0988 | 1.6904 | 0.0009 | 1.1376 | 0.6518 | 0.5970 | 0.0736 | 2.2080 | 0.0318 | 0.8954 | 0.8079 | 2.0324 | 0.0117 |
| 88 | Q13263 | Transcription intermediary factor 1-beta (TIF1-beta) (E3 SUMO-protein ligase TRIM28) (EC 6.3.2.-) (KRAB-associated protein 1) (KAP-1) (KRAB-interacting protein 1) (KRIP-1) (Nuclear corepressor KAP-1) (RING finger protein 96) (Tripartite motif-containing protein 28) | TRIM28 KAP1 RNF96 TIF1B | 9 | 3 | 1.4588 | 0.3195 | 1.0765 | 0.8132 | 1.3062 | 0.6209 | 1.6144 | 0.4448 | 1.3804 | 0.9404 | 2.0701 | 0.1669 | 1.4454 | 0.3721 | 0.9817 | 0.7109 | 2.0137 | 0.0425 | 1.5276 | 0.3775 | 2.7290 | 0.0051 | 1.7865 | 0.1294 | 1.9953 | 0.0472 | 1.4997 | 0.0701 |
| 89 | Q14258 | E3 ubiquitin/ISG15 ligase TRIM25 (EC 6.3.2.n3) (Estrogen-responsive finger protein) (RING finger protein 147) (RING-type E3 ubiquitin transferase) (EC 2.3.2.27) (Tripartite motif-containing protein 25) (Ubiquitin/ISG15-conjugating enzyme TRIM25) (Zinc finger protein 147) | TRIM25 EFP RNF147 ZNF147 | 4 | 4 | 0.5808 | 0.2310 | 1.0471 | 0.7631 | 0.5649 | 0.2931 | 0.9550 | 0.8974 | 0.8551 | 0.4897 | 1.2023 | 0.3625 | 0.5395 | 0.1763 | 0.9638 | 0.8395 | 0.5598 | 0.1081 | 1.0965 | 0.5747 | 1.1272 | 0.8110 | 1.2359 | 0.3196 | 0.8551 | 0.5064 | 1.2942 | 0.2314 |
| 90 | Q12931 | Heat shock protein 75 kDa, mitochondrial (HSP 75) (TNFR-associated protein 1) (Tumor necrosis factor type 1 receptor-associated protein) (TRAP-1) | TRAP1 HSP75 | 22 | 21 | 1.1066 | 0.9508 | 0.8630 | 0.7468 | 0.8166 | 0.8003 | 1.1482 | 0.5828 | 0.8091 | 0.2256 | 0.5598 | 0.3929 | 1.5417 | 0.1474 | 1.5704 | 0.1109 | 0.9120 | 0.4278 | 1.0375 | 0.7179 | 1.1376 | 0.8631 | 0.9120 | 0.8075 | 1.2474 | 0.8451 | 1.2359 | 0.4057 |
| 91 | P62995 | Transformer-2 protein homolog beta (TRA-2 beta) (TRA2-beta) (hTRA2-beta) (Splicing factor, arginine/serine-rich 10) (Transformer-2 protein homolog B) | TRA2B SFRS10 | 3 | 3 | 2.6792 | 0.3163 | 0.4966 | 0.7300 | 2.2699 | 0.3610 | 0.7586 | 0.8251 | 2.2491 | 0.6054 | 1.4322 | 0.4446 | 1.7701 | 0.5474 | 0.5546 | 0.9233 | 2.1478 | 0.5334 | 1.1482 | 0.7158 | 2.4889 | 0.5187 | 1.2706 | 0.5491 | 1.1272 | 0.9373 | 0.7178 | 0.9433 |
| 92 | P13693 | Translationally-controlled tumor protein (TCTP) (Fortilin) (Histamine-releasing factor) (HRF) (p23) | TPT1 | 4 | 4 | 0.4966 | 0.5975 | 0.9550 | 0.2255 | 0.9120 | 0.9140 | 1.1169 | 0.5677 | 1.4060 | 0.4697 | 1.0000 | 0.4035 | 0.8790 | 0.8020 | 0.9908 | 0.9386 | 0.6081 | 0.6496 | 1.0375 | 0.7527 | 1.3062 | 0.8277 | 0.9817 | 0.6440 | 1.2134 | 0.8378 | 1.0000 | 0.6885 |
| 93 | P29144 | Tripeptidyl-peptidase 2 (TPP-2) (EC 3.4.14.10) (Tripeptidyl aminopeptidase) (Tripeptidyl-peptidase II) (TPP-II) | TPP2 | 9 | 12 | 0.9550 | 0.7103 | 0.5495 | 0.0630 | 1.0666 | 0.8976 | 0.8472 | 0.4781 | 1.0666 | 0.8434 | 1.3305 | 0.2377 | 0.9462 | 0.5289 | 0.5808 | 0.0188 | 1.0568 | 0.8922 | 0.9908 | 0.7892 | 0.7586 | 0.0394 | 1.2246 | 0.4344 | 1.1803 | 0.7177 | 1.1169 | 0.7891 |
| 94 | O14773 | Tripeptidyl-peptidase 1 (TPP-1) (EC 3.4.14.9) (Cell growth-inhibiting gene 1 protein) (Lysosomal pepstatin-insensitive protease) (LPIC) (Tripeptidyl aminopeptidase) (Tripeptidyl-peptidase I) (TPP-I) | TPP1 CLN2 GIG1 UNQ267/PRO304 | 20 | 19 | 0.5970 | 0.3819 | 0.7656 | 0.5605 | 0.9376 | 0.7996 | 0.5702 | 0.3879 | 0.6982 | 0.8764 | 1.4588 | 0.5032 | 0.8017 | 0.8444 | 1.3552 | 0.5304 | 0.7311 | 0.6550 | 1.3552 | 0.3188 | 0.8091 | 0.8179 | 2.6303 | 0.0329 | 0.3342 | 0.0486 | 0.5495 | 0.5033 |
| 95 | P51580 | Thiopurine S-methyltransferase (EC 2.1.1.67) (Thiopurine methyltransferase) | TPMT | 5 | 6 | 1.2474 | 0.7092 | 0.7870 | 0.4564 | 0.6546 | 0.2790 | 0.9817 | 0.6181 | 1.0864 | 0.9316 | 1.0765 | 0.8335 | 1.6904 | 0.1111 | 1.0864 | 0.7987 | 0.8017 | 0.4941 | 0.6855 | 0.3304 | 0.8872 | 0.3185 | 0.8318 | 0.5760 | 1.1376 | 0.5615 | 1.1588 | 0.7006 |
| 96 | P67936 | Tropomyosin alpha-4 chain (TM30p1) (Tropomyosin-4) | TPM4 | 25 | 19 | 0.6730 | 0.1225 | 0.4018 | 0.0527 | 1.2823 | 0.4391 | 0.5702 | 0.2285 | 0.5754 | 0.0724 | 0.5152 | 0.1624 | 0.5058 | 0.0539 | 0.4920 | 0.1816 | 1.2942 | 0.7807 | 0.5445 | 0.2314 | 1.3804 | 0.0585 | 0.2355 | 0.0173 | 1.2246 | 0.4172 | 1.0864 | 0.6730 |
| 97 | P06753 | Tropomyosin alpha-3 chain (Gamma-tropomyosin) (Tropomyosin-3) (Tropomyosin-5) (hTM5) | TPM3 | 34 | 25 | 1.1169 | 0.8802 | 2.6303 | 0.0791 | 1.3677 | 0.5162 | 1.7061 | 0.1807 | 1.9409 | 0.1463 | 2.4210 | 0.0444 | 1.2246 | 0.7846 | 1.4997 | 0.3942 | 2.2284 | 0.1198 | 2.4434 | 0.1277 | 2.4660 | 0.0146 | 2.1478 | 0.0734 | 2.2284 | 0.0479 | 1.7865 | 0.0884 |
| 98 | P09493 | Tropomyosin alpha-1 chain (Alpha-tropomyosin) (Tropomyosin-1) | TPM1 C15orf13 TMSA | 22 | 19 | 0.2831 | 0.4683 | 1.3932 | 0.3696 | 3.1333 | 0.0019 | 1.5996 | 0.4834 | 1.2246 | 0.6701 | 1.8197 | 0.4579 | 1.4859 | 0.3147 | 0.6668 | 0.4800 | 1.5560 | 0.2877 | 1.5849 | 0.3348 | 2.5119 | 0.0219 | 1.1803 | 0.7349 | 1.3062 | 0.3946 | 0.7178 | 0.6627 |
| 99 | P60174 | Triosephosphate isomerase (TIM) (EC 5.3.1.1) (Triose-phosphate isomerase) | TPI1 TPI | 58 | 50 | 0.4055 | 0.0037 | 0.8790 | 0.2434 | 0.6252 | 0.0442 | 1.1482 | 0.7997 | 0.9120 | 0.6683 | 1.6444 | 0.0143 | 0.9817 | 0.9210 | 0.4246 | 0.0003 | 0.8395 | 0.4410 | 1.1272 | 0.4982 | 0.4093 | 0.0019 | 1.6144 | 0.0460 | 0.2421 | 0.0003 | 0.9120 | 0.9765 |
| 100 | O43399 | Tumor protein D54 (hD54) (Tumor protein D52-like 2) | TPD52L2 | 3 | 5 | 1.2359 | 0.4140 | 1.1912 | 0.5678 | 1.4723 | 0.2595 | 0.9727 | 0.9359 | 1.6293 | 0.2684 | 1.1695 | 0.6362 | 1.6596 | 0.2587 | 0.9550 | 0.8929 | 2.0701 | 0.0473 | 1.1376 | 0.6685 | 1.8707 | 0.1907 | 0.9290 | 0.6897 | 1.3552 | 0.2806 | 1.3804 | 0.2032 |
| 101 | O94826 | Mitochondrial import receptor subunit TOM70 (Mitochondrial precursor proteins import receptor) (Translocase of outer membrane 70 kDa subunit) | TOMM70A KIAA0719 TOM70 | 13 | 9 | 0.5012 | 0.1368 | 0.5970 | 0.3806 | 0.8091 | 0.3879 | 1.0864 | 0.7073 | 0.6607 | 0.1791 | 1.3305 | 0.4028 | 0.6138 | 0.0175 | 1.1272 | 0.8608 | 0.6792 | 0.0726 | 1.3932 | 0.3514 | 1.0375 | 0.8401 | 1.4588 | 0.3933 | 0.7311 | 0.2871 | 1.3804 | 0.2366 |
| 102 | O60784 | Target of Myb protein 1 | TOM1 | 8 | 5 | 0.8318 | 0.6860 | 0.9036 | 0.7790 | 1.5276 | 0.1458 | 0.6918 | 0.6266 | 1.4588 | 0.0713 | 1.3552 | 0.5158 | 0.7798 | 0.7758 | 1.7219 | 0.5140 | 0.4406 | 0.3841 | 0.4406 | 0.6351 | 0.7870 | 0.4783 | 1.2823 | 0.7385 | 0.7870 | 0.8881 | 1.3677 | 0.6406 |
| 103 | P22105 | Tenascin-X (TN-X) (Hexabrachion-like protein) | TNXB HXBL TNX TNXB1 TNXB2 XB | 33 | 38 | 0.9727 | 0.6356 | 3.9084 | 0.1177 | 2.0701 | 0.0000 | 4.3652 | 0.0808 | 0.7047 | 0.4293 | 1.2359 | 0.6159 | 0.8166 | 0.3848 | 1.9055 | 0.5396 | 1.9588 | 0.0003 | 1.2359 | 0.8746 | 0.8872 | 0.8588 | 0.5152 | 0.4050 | 1.0093 | 0.6839 | 2.3988 | 0.1750 |
| 104 | Q9HBL0 | Tensin-1 | TNS1 TNS | 9 | 14 | 0.8710 | 0.9506 | 1.9231 | 0.0926 | 1.7865 | 0.0107 | 1.2474 | 0.7187 | 1.0568 | 0.7198 | 1.4191 | 0.6993 | 0.8630 | 0.6958 | 2.6792 | 0.1166 | 0.9550 | 0.9646 | 0.9908 | 0.9211 | 0.6982 | 0.6994 | 0.7516 | 0.4315 | 0.8551 | 0.6693 | 0.9462 | 0.3226 |
| 105 | Q92973 | Transportin-1 (Importin beta-2) (Karyopherin beta-2) (M9 region interaction protein) (MIP) | TNPO1 KPNB2 MIP1 TRN | 5 | 8 | 0.8790 | 0.8544 | 1.4454 | 0.4558 | 2.0512 | 0.4465 | 1.5276 | 0.3032 | 0.8472 | 0.6458 | 1.7219 | 0.2152 | 1.9770 | 0.3666 | 1.2359 | 0.3515 | 0.6855 | 0.3686 | 3.4995 | 0.0664 | 1.5417 | 0.7744 | 0.6855 | 0.5313 | 1.5704 | 0.7661 | 2.2699 | 0.1531 |
| 106 | Q9C0C2 | 182 kDa tankyrase-1-binding protein | TNKS1BP1 KIAA1741 TAB182 | 8 | 10 | 0.9376 | 0.5013 | 0.8954 | 0.7657 | 0.9204 | 0.6030 | 1.0568 | 0.8662 | 0.8790 | 0.3537 | 0.9638 | 0.7952 | 0.9376 | 0.4909 | 1.2134 | 0.7437 | 0.9204 | 0.4973 | 1.4322 | 0.1309 | 0.9290 | 0.6384 | 1.3677 | 0.5544 | 1.0186 | 0.8004 | 1.8365 | 0.0851 |
| 107 | P24821 | Tenascin (TN) (Cytotactin) (GMEM) (GP 150-225) (Glioma-associated-extracellular matrix antigen) (Hexabrachion) (JI) (Myotendinous antigen) (Neuronectin) (Tenascin-C) (TN-C) | TNC HXB | 12 | 10 | 0.9550 | 0.8497 | 2.1677 | 0.0309 | 2.0137 | 0.0813 | 2.7542 | 0.0121 | 1.1695 | 0.5530 | 1.4454 | 0.3866 | 1.3305 | 0.6729 | 2.2491 | 0.0437 | 2.3121 | 0.0274 | 1.8535 | 0.2754 | 1.5849 | 0.0787 | 1.2474 | 0.7051 | 1.5276 | 0.0866 | 2.8054 | 0.0225 |
| 108 | P42167 | Lamina-associated polypeptide 2, isoforms beta/gamma (Thymopoietin, isoforms beta/gamma) (TP beta/gamma) (Thymopoietin-related peptide isoforms beta/gamma) (TPRP isoforms beta/gamma) [Cleaved into: Thymopoietin (TP) (Splenin); Thymopentin (TP5)] | TMPO LAP2 | 6 | 6 | 0.5346 | 0.7919 | 0.8954 | 0.2786 | 1.2134 | 0.6122 | 0.8395 | 0.0496 | 0.1977 | 0.2534 | 0.8954 | 0.2287 | 0.7516 | 0.4883 | 0.8710 | 0.1991 | 0.7178 | 0.8318 | 0.7586 | 0.0733 | 1.1169 | 0.7112 | 0.8318 | 0.2481 | 1.3183 | 0.6742 | 0.9036 | 0.3388 |
| 109 | Q9NYL9 | Tropomodulin-3 (Ubiquitous tropomodulin) (U-Tmod) | TMOD3 | 10 | 5 | 1.4997 | 0.9382 | 1.9231 | 0.5891 | 1.6596 | 0.8293 | 3.2509 | 0.1942 | 2.9376 | 0.1777 | 3.3419 | 0.0514 | 1.6444 | 0.8125 | 2.5119 | 0.3046 | 2.9923 | 0.1120 | 1.5704 | 0.7290 | 1.2134 | 0.7764 | 1.8030 | 0.6108 | 0.8395 | 0.4226 | 0.9462 | 0.8785 |
| 110 | Q6UW68 | Transmembrane protein 205 | TMEM205 UNQ501/PRO1018 | 4 | 5 | 1.5704 | 0.2309 | 1.3062 | 0.4952 | 1.1376 | 0.9021 | 1.2359 | 0.4788 | 0.8395 | 0.8840 | 1.0280 | 0.8421 | 1.0093 | 0.7055 | 0.9550 | 0.9018 | 1.4723 | 0.2349 | 0.9908 | 0.8397 | 0.8472 | 0.7604 | 1.0765 | 0.9424 | 0.7516 | 0.5285 | 1.1272 | 0.7239 |
| 111 | Q9BVK6 | Transmembrane emp24 domain-containing protein 9 (GMP25) (Glycoprotein 25L2) (p24 family protein alpha-2) (p24alpha2) (p25) | TMED9 GP25L2 | 8 | 8 | 0.7178 | 0.5073 | 0.7244 | 0.4435 | 0.8395 | 0.3162 | 0.9550 | 0.8530 | 0.4831 | 0.3263 | 0.8872 | 0.7671 | 0.8241 | 0.7088 | 1.0375 | 0.8810 | 0.4571 | 0.0477 | 0.9817 | 0.7741 | 0.7870 | 0.5181 | 1.2589 | 0.6654 | 0.8395 | 0.4320 | 1.0568 | 0.9422 |
| 112 | Q9Y3B3 | Transmembrane emp24 domain-containing protein 7 (p24 family protein gamma-3) (p24gamma3) (p27) | TMED7 CGI-109 | 6 | 5 | 0.5152 | 0.3855 | 0.8091 | 0.8224 | 0.7311 | 0.8797 | 0.2512 | 0.8217 | 1.2134 | 0.6331 | 0.4656 | 0.2689 | 0.9462 | 0.6195 | 1.0666 | 0.9650 | 0.3698 | 0.2654 | 0.4966 | 0.5500 | 0.7870 | 0.9564 | 0.4055 | 0.7044 | 1.0375 | 0.9880 | 0.9908 | 0.3348 |
| 113 | P49755 | Transmembrane emp24 domain-containing protein 10 (21 kDa transmembrane-trafficking protein) (S31III125) (S31I125) (Tmp-21-I) (Transmembrane protein Tmp21) (p23) (p24 family protein delta-1) (p24delta1) (p24delta) | TMED10 TMP21 | 10 | 9 | 0.7870 | 0.0245 | 1.0864 | 0.5094 | 0.9290 | 0.6598 | 1.1695 | 0.3294 | 1.0000 | 0.7280 | 1.0471 | 0.5385 | 0.8790 | 0.3259 | 1.0375 | 0.2851 | 0.9204 | 0.5291 | 1.1588 | 0.1527 | 0.9550 | 0.5448 | 1.2134 | 0.1674 | 0.9462 | 0.5553 | 1.1912 | 0.1469 |
| 114 | Q9Y490 | Talin-1 | TLN1 KIAA1027 TLN | 99 | 97 | 1.0864 | 0.5879 | 0.9036 | 0.7704 | 2.2909 | 0.0010 | 1.2474 | 0.0272 | 1.4322 | 0.2542 | 1.2474 | 0.1003 | 1.0965 | 0.8683 | 1.0765 | 0.2014 | 1.2023 | 0.8414 | 1.3428 | 0.0698 | 2.2699 | 0.0019 | 0.9120 | 0.9273 | 1.8197 | 0.0623 | 1.4322 | 0.0021 |
| 115 | P29401 | Transketolase (TK) (EC 2.2.1.1) | TKT | 33 | 28 | 0.4207 | 0.0654 | 0.8551 | 0.2798 | 0.9036 | 0.6008 | 0.5754 | 0.0059 | 0.9817 | 0.7134 | 1.1695 | 0.8447 | 0.7047 | 0.0975 | 0.8472 | 0.3434 | 0.9727 | 0.8919 | 0.9290 | 0.3487 | 2.4434 | 0.0004 | 0.8318 | 0.1358 | 1.8365 | 0.0117 | 1.4859 | 0.0942 |
| 116 | Q07157 | Tight junction protein ZO-1 (Tight junction protein 1) (Zona occludens protein 1) (Zonula occludens protein 1) | TJP1 ZO1 | 3 | 4 | 1.2706 | 0.5537 | 1.0471 | 0.7441 | 1.5136 | 0.1160 | 0.9462 | 0.7355 | 0.9908 | 0.9836 | 1.0666 | 0.7906 | 1.2134 | 0.2954 | 0.9550 | 0.9448 | 1.4322 | 0.1341 | 0.9290 | 0.6555 | 1.3305 | 0.1965 | 0.8954 | 0.5399 | 1.1912 | 0.5955 | 0.9727 | 0.8637 |
| 117 | Q3ZCQ8 | Mitochondrial import inner membrane translocase subunit TIM50 | TIMM50 TIM50 PRO1512 | 6 | 3 | 0.9120 | 0.6648 | 0.2992 | 0.0945 | 1.0186 | 0.6540 | 0.4055 | 0.5161 | 1.0471 | 0.6098 | 0.8395 | 0.4925 | 1.2942 | 0.3158 | 0.7516 | 0.8206 | 1.2134 | 0.3896 | 0.6368 | 0.2779 | 0.8954 | 0.6620 | 0.2858 | 0.1709 | 1.3305 | 0.2981 | 0.8166 | 0.6619 |
| 118 | Q9Y5L4 | Mitochondrial import inner membrane translocase subunit Tim13 | TIMM13 TIM13B TIMM13A TIMM13B | 5 | 4 | 1.1912 | 0.5071 | 4.5709 | 0.1316 | 1.1695 | 0.4995 | 3.0200 | 0.1725 | 1.4997 | 0.3543 | 2.8576 | 0.2671 | 1.2823 | 0.3998 | 3.6308 | 0.1929 | 1.4723 | 0.3957 | 3.4995 | 0.2255 | 1.0864 | 0.5802 | 5.2000 | 0.1030 | 1.0375 | 0.6783 | 3.2509 | 0.1720 |
| 119 | P52888 | Thimet oligopeptidase (EC 3.4.24.15) (Endopeptidase 24.15) (MP78) | THOP1 | 7 | 4 | 0.9036 | 0.7098 | 1.1695 | 0.5386 | 1.2823 | 0.6112 | 0.7943 | 0.3398 | 1.1588 | 0.5492 | 1.0864 | 0.7848 | 0.8954 | 0.6649 | 0.9036 | 0.6890 | 0.7656 | 0.4351 | 1.3428 | 0.4586 | 1.2134 | 0.5710 | 1.0280 | 0.8594 | 1.8030 | 0.1870 | 0.6368 | 0.4535 |
| 120 | Q86V81 | THO complex subunit 4 (Tho4) (Ally of AML-1 and LEF-1) (Aly/REF export factor) (Transcriptional coactivator Aly/REF) (bZIP-enhancing factor BEF) | ALYREF ALY BEF THOC4 | 4 | 6 | 1.5996 | 0.4110 | 4.4875 | 0.1822 | 1.4997 | 0.3568 | 2.3988 | 0.3241 | 1.8365 | 0.2317 | 4.0179 | 0.1299 | 1.5276 | 0.3920 | 3.1915 | 0.0986 | 1.3804 | 0.3975 | 7.9433 | 0.0899 | 1.8030 | 0.4979 | 5.9704 | 0.0909 | 1.4060 | 0.7686 | 3.4995 | 0.1126 |
| 121 | Q8IYQ7 | Threonine synthase-like 1 (TSH1) | THNSL1 | 4 | 5 | 1.0568 | 0.9035 | 1.2359 | 0.7078 | 0.5495 | 0.1481 | 0.8630 | 0.6043 | 0.5546 | 0.2626 | 0.4285 | 0.1968 | 0.6252 | 0.1989 | 1.2134 | 0.8357 | 1.0186 | 0.9407 | 0.4169 | 0.5120 | 0.5754 | 0.3070 | 0.8017 | 0.1597 | 0.3076 | 0.1478 | 0.4406 | 0.0581 |
| 122 | P21980 | Protein-glutamine gamma-glutamyltransferase 2 (EC 2.3.2.13) (Tissue transglutaminase) (Transglutaminase C) (TG(C)) (TGC) (TGase C) (Transglutaminase H) (TGase H) (Transglutaminase-2) (TGase-2) | TGM2 | 34 | 34 | 0.3733 | 0.8366 | 0.2858 | 0.0770 | 0.8954 | 0.2936 | 1.0000 | 0.9836 | 0.6855 | 0.5466 | 1.1588 | 0.4396 | 2.0512 | 0.0649 | 1.2023 | 0.2370 | 0.5248 | 0.4816 | 1.1066 | 0.5483 | 1.1169 | 0.2379 | 1.0000 | 0.7527 | 0.8954 | 0.2281 | 1.7061 | 0.0154 |
| 123 | Q15582 | Transforming growth factor-beta-induced protein ig-h3 (Beta ig-h3) (Kerato-epithelin) (RGD-containing collagen-associated protein) (RGD-CAP) | TGFBI BIGH3 | 27 | 32 | 1.2246 | 0.3874 | 1.2589 | 0.3371 | 2.9376 | 0.0005 | 1.8365 | 0.0284 | 0.8954 | 0.7151 | 2.1878 | 0.0513 | 0.5495 | 0.5682 | 1.1588 | 0.7046 | 3.0479 | 0.0001 | 0.8395 | 0.3297 | 1.5136 | 0.0653 | 0.3873 | 0.0465 | 1.5417 | 0.1665 | 2.5119 | 0.0004 |
| 124 | Q9UP52 | Transferrin receptor protein 2 (TfR2) | TFR2 | 10 | 9 | 1.2706 | 0.5784 | 2.2909 | 0.1124 | 0.7870 | 0.8951 | 2.4434 | 0.1769 | 0.7447 | 0.9206 | 2.1677 | 0.1927 | 1.8197 | 0.1339 | 2.5586 | 0.1002 | 1.1695 | 0.6199 | 1.0864 | 0.7193 | 0.3597 | 0.4588 | 1.9953 | 0.2502 | 1.6749 | 0.2249 | 3.0761 | 0.0734 |
| 125 | Q92734 | Protein TFG (TRK-fused gene protein) | TFG | 4 | 7 | 1.8880 | 0.5821 | 0.6427 | 0.9772 | 1.6596 | 0.2878 | 1.2246 | 0.5830 | 1.2359 | 0.6043 | 2.6062 | 0.3086 | 0.9120 | 0.8025 | 2.5823 | 0.2477 | 2.4660 | 0.1405 | 1.4859 | 0.5001 | 0.8710 | 0.8464 | 1.9409 | 0.4503 | 2.2699 | 0.1218 | 1.7378 | 0.2912 |
| 126 | P02787 | Serotransferrin (Transferrin) (Beta-1 metal-binding globulin) (Siderophilin) | TF PRO1400 | 62 | 50 | 0.3076 | 0.0005 | 0.6081 | 0.0803 | 1.5560 | 0.0017 | 0.6138 | 0.0010 | 2.4660 | 0.0000 | 0.8872 | 0.3056 | 0.4571 | 0.0046 | 0.9550 | 0.4018 | 1.5417 | 0.1551 | 1.9409 | 0.0008 | 0.7047 | 0.0102 | 0.8954 | 0.2511 | 0.7178 | 0.1100 | 1.3932 | 0.0116 |
| 127 | P17987 | T-complex protein 1 subunit alpha (TCP-1-alpha) (CCT-alpha) | TCP1 CCT1 CCTA | 21 | 23 | 0.9376 | 0.4257 | 1.6144 | 0.9055 | 1.5136 | 0.8169 | 1.4454 | 0.9484 | 2.1677 | 0.1638 | 2.7797 | 0.1379 | 1.3062 | 0.9366 | 2.3335 | 0.1769 | 1.7865 | 0.9416 | 2.8840 | 0.0240 | 1.5136 | 0.2137 | 2.4434 | 0.5535 | 1.4060 | 0.8636 | 3.1046 | 0.0217 |
| 128 | O75347 | Tubulin-specific chaperone A (TCP1-chaperonin cofactor A) (Tubulin-folding cofactor A) (CFA) | TBCA | 5 | 7 | 0.5495 | 0.2382 | 2.4660 | 0.3190 | 0.8710 | 0.5322 | 1.4723 | 0.7131 | 1.6144 | 0.5098 | 2.5586 | 0.3008 | 1.4588 | 0.8074 | 2.6062 | 0.1391 | 0.8710 | 0.3709 | 2.5351 | 0.2878 | 1.2589 | 0.9894 | 2.6546 | 0.0906 | 1.3428 | 0.9976 | 2.3988 | 0.2497 |
| 129 | P26639 | Threonine--tRNA ligase, cytoplasmic (EC 6.1.1.3) (Threonyl-tRNA synthetase) (ThrRS) | TARS | 10 | 11 | 1.5136 | 0.0271 | 0.5754 | 0.3281 | 1.0568 | 0.8537 | 0.5012 | 0.2419 | 0.7798 | 0.2411 | 1.7219 | 0.1331 | 1.1272 | 0.8879 | 0.5702 | 0.3621 | 0.9462 | 0.4333 | 1.4191 | 0.5383 | 1.1376 | 0.8131 | 0.5649 | 0.0136 | 0.6918 | 0.3815 | 0.6486 | 0.3302 |
| 130 | Q13148 | TAR DNA-binding protein 43 (TDP-43) | TARDBP TDP43 | 4 | 5 | 0.8017 | 0.2978 | 0.7447 | 0.6439 | 0.9638 | 0.7232 | 0.7870 | 0.7914 | 1.5136 | 0.1601 | 1.2942 | 0.5229 | 1.2823 | 0.8993 | 1.0000 | 0.9602 | 1.0093 | 0.9091 | 1.0765 | 0.8690 | 1.3932 | 0.6982 | 1.2023 | 0.7809 | 1.0186 | 0.9167 | 0.4406 | 0.4329 |
| 131 | P37837 | Transaldolase (EC 2.2.1.2) | TALDO1 TAL TALDO TALDOR | 21 | 17 | 0.5598 | 0.0021 | 0.3281 | 0.0482 | 0.5754 | 0.0292 | 0.6730 | 0.2141 | 0.4246 | 0.0013 | 0.3945 | 0.0990 | 0.6138 | 0.0093 | 0.5970 | 0.0894 | 0.7379 | 0.1142 | 0.4966 | 0.0129 | 0.7870 | 0.1285 | 0.5546 | 0.1734 | 0.4786 | 0.0712 | 0.3436 | 0.0027 |
| 132 | P37802 | Transgelin-2 (Epididymis tissue protein Li 7e) (SM22-alpha homolog) | TAGLN2 KIAA0120 CDABP0035 | 23 | 23 | 0.3076 | 0.1096 | 0.6252 | 0.4411 | 2.2909 | 0.0402 | 1.2474 | 0.6822 | 2.0701 | 0.1812 | 2.4434 | 0.0038 | 1.3552 | 0.8396 | 1.1272 | 0.6001 | 1.5276 | 0.7944 | 2.3768 | 0.0123 | 4.3251 | 0.0001 | 0.5970 | 0.3050 | 2.0512 | 0.0495 | 3.1623 | 0.0002 |
| 133 | Q01995 | Transgelin (22 kDa actin-binding protein) (Protein WS3-10) (Smooth muscle protein 22-alpha) (SM22-alpha) | TAGLN SM22 WS3-10 | 9 | 11 | 0.1871 | 0.0054 | 0.2754 | 0.1104 | 1.3552 | 0.5149 | 2.5586 | 0.0004 | 0.9376 | 0.8498 | 0.8710 | 0.8879 | 0.2559 | 0.0006 | 1.1272 | 0.1480 | 0.5248 | 0.0124 | 0.4246 | 0.0569 | 1.2134 | 0.9499 | 0.1585 | 0.0102 | 0.5702 | 0.1365 | 1.1169 | 0.5997 |
| 134 | O60506 | Heterogeneous nuclear ribonucleoprotein Q (hnRNP Q) (Glycine- and tyrosine-rich RNA-binding protein) (GRY-RBP) (NS1-associated protein 1) (Synaptotagmin-binding, cytoplasmic RNA-interacting protein) | SYNCRIP HNRPQ NSAP1 | 11 | 11 | 0.8551 | 0.9952 | 0.4831 | 0.1724 | 0.8630 | 0.5774 | 0.9908 | 0.9256 | 1.3804 | 0.0777 | 1.2706 | 0.2170 | 1.0000 | 0.9426 | 0.7311 | 0.9274 | 0.5702 | 0.6260 | 0.8472 | 0.7067 | 1.0375 | 0.4969 | 1.2359 | 0.0228 | 0.8790 | 0.8251 | 0.9727 | 0.4691 |
| 135 | O15260 | Surfeit locus protein 4 | SURF4 SURF-4 | 7 | 6 | 1.0666 | 0.9622 | 1.4723 | 0.4618 | 0.9120 | 0.2164 | 1.4060 | 0.5859 | 0.9376 | 0.8766 | 0.8954 | 0.7429 | 0.9550 | 0.4427 | 1.1588 | 0.8097 | 0.9204 | 0.8005 | 1.0666 | 0.8860 | 0.9638 | 0.6138 | 1.4454 | 0.4943 | 0.9462 | 0.5328 | 1.0093 | 0.8192 |
| 136 | P51687 | Sulfite oxidase, mitochondrial (EC 1.8.3.1) | SUOX | 9 | 17 | 0.8790 | 0.9703 | 1.1376 | 0.5069 | 1.1695 | 0.3890 | 1.3552 | 0.2687 | 1.2823 | 0.3909 | 1.0375 | 0.8111 | 1.0093 | 0.6430 | 1.3677 | 0.2066 | 1.0093 | 0.6734 | 1.0280 | 0.9108 | 0.2805 | 0.0048 | 1.0375 | 0.3134 | 0.7516 | 0.3169 | 0.7311 | 0.3707 |
| 137 | Q06520 | Bile salt sulfotransferase (EC 2.8.2.14) (Dehydroepiandrosterone sulfotransferase) (DHEA-ST) (Hydroxysteroid Sulfotransferase) (HST) (ST2) (ST2A3) (Sulfotransferase 2A1) (ST2A1) | SULT2A1 HST STD | 43 | 35 | 1.0186 | 0.5623 | 2.1086 | 0.0682 | 1.3305 | 0.3162 | 2.3768 | 0.0095 | 0.1977 | 0.0000 | 2.1281 | 0.0008 | 0.6138 | 0.0469 | 1.3428 | 0.6434 | 2.1281 | 0.0083 | 0.6982 | 0.3402 | 1.1803 | 0.6325 | 0.5546 | 0.6269 | 1.0093 | 0.9036 | 0.9817 | 0.4693 |
| 138 | P50225 | Sulfotransferase 1A1 (ST1A1) (EC 2.8.2.1) (Aryl sulfotransferase 1) (HAST1/HAST2) (Phenol sulfotransferase 1) (Phenol-sulfating phenol sulfotransferase 1) (P-PST 1) (ST1A3) (Thermostable phenol sulfotransferase) (Ts-PST) | SULT1A1 STP STP1 OK/SW-cl.88 | 28 | 27 | 2.5823 | 0.0001 | 0.3945 | 0.0302 | 0.7943 | 0.1538 | 1.1376 | 0.7609 | 0.9817 | 0.7209 | 0.2355 | 0.0017 | 0.9462 | 0.1431 | 0.3664 | 0.0039 | 0.4571 | 0.0013 | 0.5754 | 0.0222 | 0.3436 | 0.0232 | 0.5200 | 0.0140 | 0.5297 | 0.0010 | 0.7798 | 0.3629 |
| 139 | Q96I99 | Succinyl-CoA ligase [GDP-forming] subunit beta, mitochondrial (EC 6.2.1.4) (GTP-specific succinyl-CoA synthetase subunit beta) (Succinyl-CoA synthetase beta-G chain) (SCS-betaG) | SUCLG2 | 35 | 38 | 1.2474 | 0.7624 | 1.7865 | 0.0210 | 0.9817 | 0.2538 | 1.1588 | 0.2570 | 1.1169 | 0.5885 | 1.1066 | 0.7677 | 1.5849 | 0.2556 | 0.9817 | 0.9189 | 1.9231 | 0.0744 | 1.2134 | 0.4689 | 0.2938 | 0.0001 | 1.0765 | 0.8613 | 0.6138 | 0.0024 | 1.2359 | 0.8508 |
| 140 | P53597 | Succinyl-CoA ligase [ADP/GDP-forming] subunit alpha, mitochondrial (EC 6.2.1.4) (EC 6.2.1.5) (Succinyl-CoA synthetase subunit alpha) (SCS-alpha) | SUCLG1 | 17 | 14 | 0.6668 | 0.2165 | 1.1588 | 0.6423 | 0.7379 | 0.2166 | 1.1482 | 0.7222 | 0.4786 | 0.1265 | 1.0965 | 0.6210 | 1.0666 | 0.8320 | 0.8872 | 0.2841 | 0.8166 | 0.6033 | 1.1066 | 0.8304 | 0.3251 | 0.0054 | 1.0666 | 0.9895 | 0.2884 | 0.0027 | 0.9817 | 0.6048 |
| 141 | P53999 | Activated RNA polymerase II transcriptional coactivator p15 (Positive cofactor 4) (PC4) (SUB1 homolog) (p14) | SUB1 PC4 RPO2TC1 | 4 | 5 | 1.1376 | 0.7866 | 1.9055 | 0.0563 | 1.4322 | 0.3334 | 1.2589 | 0.0673 | 0.8091 | 0.8879 | 1.4060 | 0.1014 | 1.2359 | 0.7885 | 0.8472 | 0.1483 | 1.4997 | 0.5350 | 1.8880 | 0.0821 | 2.5586 | 0.1952 | 1.4859 | 0.1358 | 1.7219 | 0.5662 | 2.3335 | 0.0486 |
| 142 | Q8TCJ2 | Dolichyl-diphosphooligosaccharide--protein glycosyltransferase subunit STT3B (Oligosaccharyl transferase subunit STT3B) (STT3-B) (EC 2.4.99.18) (Source of immunodominant MHC-associated peptides homolog) | STT3B SIMP | 6 | 5 | 0.9204 | 0.7392 | 1.1695 | 0.2755 | 1.0568 | 0.9679 | 1.1066 | 0.4715 | 0.6918 | 0.1188 | 1.0965 | 0.4375 | 0.8241 | 0.5789 | 1.0000 | 0.7885 | 0.9638 | 0.9721 | 1.1272 | 0.2985 | 1.2023 | 0.5257 | 1.0765 | 0.4974 | 1.2134 | 0.5858 | 1.0568 | 0.6248 |
| 143 | P46977 | Dolichyl-diphosphooligosaccharide--protein glycosyltransferase subunit STT3A (Oligosaccharyl transferase subunit STT3A) (STT3-A) (EC 2.4.99.18) (B5) (Integral membrane protein 1) (Transmembrane protein TMC) | STT3A ITM1 TMC | 7 | 7 | 0.9817 | 0.8898 | 0.9908 | 0.9102 | 0.7244 | 0.1672 | 0.9462 | 0.4305 | 0.5598 | 0.0284 | 0.8872 | 0.2021 | 1.0000 | 0.9546 | 0.8872 | 0.3370 | 0.9462 | 0.8811 | 0.9290 | 0.3427 | 1.1376 | 0.3766 | 0.8790 | 0.1976 | 0.9908 | 0.8425 | 0.9290 | 0.3840 |
| 144 | Q9UJZ1 | Stomatin-like protein 2, mitochondrial (SLP-2) (EPB72-like protein 2) (Paraprotein target 7) (Paratarg-7) | STOML2 SLP2 HSPC108 | 14 | 12 | 1.1272 | 0.7135 | 1.0864 | 0.4250 | 1.0471 | 0.7049 | 0.9204 | 0.8839 | 0.9550 | 0.7786 | 0.9817 | 0.8995 | 1.1376 | 0.3835 | 0.9817 | 0.7230 | 1.0965 | 0.5125 | 0.9638 | 0.9886 | 1.0568 | 0.5285 | 1.0666 | 0.5939 | 1.1482 | 0.3408 | 1.0093 | 0.7663 |
| 145 | P31948 | Stress-induced-phosphoprotein 1 (STI1) (Hsc70/Hsp90-organizing protein) (Hop) (Renal carcinoma antigen NY-REN-11) (Transformation-sensitive protein IEF SSP 3521) | STIP1 | 8 | 5 | 0.8710 | 0.5050 | 1.1169 | 0.7375 | 1.3183 | 0.8631 | 1.0000 | 0.8531 | 1.4997 | 0.3640 | 1.2359 | 0.1635 | 0.9638 | 0.6171 | 1.0765 | 0.6107 | 1.2134 | 0.8423 | 1.1169 | 0.5086 | 1.4723 | 0.1828 | 1.1482 | 0.3373 | 1.2942 | 0.6751 | 1.0000 | 0.7517 |
| 146 | Q13586 | Stromal interaction molecule 1 | STIM1 GOK | 3 | 8 | 0.5495 | 0.5849 | 0.8091 | 0.8285 | 0.2466 | 0.2683 | 1.3932 | 0.5585 | 0.4018 | 0.6173 | 1.3305 | 0.8474 | 0.2780 | 0.3667 | 3.1333 | 0.1832 | 0.7656 | 0.4402 | 1.3305 | 0.9508 | 0.3945 | 0.1006 | 2.3550 | 0.4372 | 1.1912 | 0.5717 | 3.1915 | 0.1759 |
| 147 | Q658P3 | Metalloreductase STEAP3 (EC 1.16.1.-) (Dudulin-2) (Six-transmembrane epithelial antigen of prostate 3) (Tumor suppressor-activated pathway protein 6) (hTSAP6) (pHyde) (hpHyde) | STEAP3 TSAP6 | 6 | 3 | 1.2023 | 0.5644 | 0.7178 | 0.4087 | 1.1376 | 0.4104 | 0.7311 | 0.2701 | 1.4454 | 0.1175 | 1.2706 | 0.8636 | 1.4997 | 0.1204 | 0.6252 | 0.1453 | 1.3305 | 0.1729 | 1.1695 | 0.7898 | 1.1066 | 0.7606 | 0.9908 | 0.7359 | 1.0186 | 0.9488 | 1.3183 | 0.5106 |
| 148 | O95210 | Starch-binding domain-containing protein 1 (Genethonin-1) (Glycophagy cargo receptor STBD1) | STBD1 GENX-3414 | 10 | 11 | 1.2823 | 0.8894 | 1.2246 | 0.7319 | 1.4060 | 0.7467 | 2.4889 | 0.3133 | 1.1695 | 0.1790 | 1.9770 | 0.2765 | 0.9204 | 0.9093 | 0.7047 | 0.8628 | 1.8030 | 0.2958 | 1.5704 | 0.5771 | 0.2291 | 0.0998 | 1.4454 | 0.6620 | 0.4446 | 0.2235 | 0.7516 | 0.6775 |
| 149 | P40763 | Signal transducer and activator of transcription 3 (Acute-phase response factor) | STAT3 APRF | 6 | 5 | 0.7311 | 0.3635 | 0.3565 | 0.3570 | 0.9290 | 0.7356 | 0.5346 | 0.6390 | 0.9290 | 0.3533 | 0.8241 | 0.9423 | 1.1912 | 0.9952 | 1.1066 | 0.8496 | 0.8241 | 0.2599 | 1.1588 | 0.7804 | 0.9550 | 0.5119 | 0.7244 | 0.7314 | 1.0375 | 0.7643 | 1.6596 | 0.7934 |
| 150 | P42224 | Signal transducer and activator of transcription 1-alpha/beta (Transcription factor ISGF-3 components p91/p84) | STAT1 | 20 | 33 | 1.1066 | 0.4655 | 2.7797 | 0.0224 | 1.0375 | 0.9935 | 0.7178 | 0.1263 | 1.6749 | 0.0235 | 11.2720 | 0.0000 | 0.8472 | 0.8184 | 1.3677 | 0.6092 | 2.5823 | 0.0007 | 5.2481 | 0.0000 | 4.6559 | 0.0000 | 2.0512 | 0.0032 | 4.5709 | 0.0000 | 6.4269 | 0.0000 |
| 151 | Q9Y365 | PCTP-like protein (PCTP-L) (Antigen NY-CO-28) (START domain-containing protein 10) (StARD10) (Serologically defined colon cancer antigen 28) (StAR-related lipid transfer protein 10) | STARD10 SDCCAG28 CGI-52 | 7 | 11 | 2.1478 | 0.0249 | 4.0179 | 0.0220 | 1.8365 | 0.0520 | 3.3113 | 0.1022 | 1.9770 | 0.1152 | 3.1046 | 0.0336 | 1.2706 | 0.1708 | 4.6559 | 0.0039 | 2.2284 | 0.0357 | 4.8306 | 0.0382 | 1.1272 | 0.2272 | 4.3251 | 0.0049 | 1.2942 | 0.3844 | 3.4995 | 0.0499 |
| 152 | P50502 | Hsc70-interacting protein (Hip) (Aging-associated protein 2) (Progesterone receptor-associated p48 protein) (Protein FAM10A1) (Putative tumor suppressor ST13) (Renal carcinoma antigen NY-REN-33) (Suppression of tumorigenicity 13 protein) | ST13 AAG2 FAM10A1 HIP SNC6 | 11 | 10 | 0.9376 | 0.9718 | 0.9638 | 0.7550 | 1.5417 | 0.5140 | 0.8710 | 0.6943 | 1.8365 | 0.2220 | 1.1482 | 0.7875 | 1.6144 | 0.5272 | 1.4060 | 0.4635 | 0.7244 | 0.7224 | 0.6855 | 0.2006 | 0.9462 | 0.7747 | 1.2823 | 0.6425 | 0.8551 | 0.3130 | 1.3804 | 0.8580 |
| 153 | P51571 | Translocon-associated protein subunit delta (TRAP-delta) (Signal sequence receptor subunit delta) (SSR-delta) | SSR4 TRAPD | 3 | 4 | 1.1272 | 0.7920 | 1.2023 | 0.3808 | 1.0471 | 0.9058 | 1.1066 | 0.5597 | 1.1376 | 0.7511 | 1.0864 | 0.5321 | 1.3183 | 0.8911 | 1.0000 | 0.8585 | 1.1912 | 0.9486 | 1.0965 | 0.7011 | 1.3932 | 0.7241 | 1.0666 | 0.6698 | 1.3932 | 0.8567 | 1.1272 | 0.6551 |
| 154 | P43307 | Translocon-associated protein subunit alpha (TRAP-alpha) (Signal sequence receptor subunit alpha) (SSR-alpha) | SSR1 TRAPA PSEC0262 | 3 | 4 | 1.0471 | 0.9568 | 2.1281 | 0.7887 | 1.4060 | 0.6465 | 2.0137 | 0.8835 | 1.4060 | 0.5492 | 1.0471 | 0.7614 | 1.3305 | 0.7504 | 2.0512 | 0.7542 | 1.2706 | 0.6825 | 2.1478 | 0.8759 | 1.4723 | 0.5213 | 1.6293 | 0.7603 | 1.3305 | 0.7238 | 2.1086 | 0.8032 |
| 155 | Q04837 | Single-stranded DNA-binding protein, mitochondrial (Mt-SSB) (MtSSB) (PWP1-interacting protein 17) | SSBP1 SSBP | 7 | 10 | 1.0000 | 0.7072 | 1.4723 | 0.0880 | 0.9204 | 0.3079 | 1.3062 | 0.3010 | 0.8954 | 0.8062 | 1.4060 | 0.1082 | 1.1066 | 0.2890 | 0.8954 | 0.6248 | 1.0568 | 0.7591 | 1.4859 | 0.3715 | 1.1272 | 0.2387 | 1.2706 | 0.4619 | 1.0765 | 0.4820 | 1.0000 | 0.7300 |
| 156 | Q16629 | Serine/arginine-rich splicing factor 7 (Splicing factor 9G8) (Splicing factor, arginine/serine-rich 7) | SRSF7 SFRS7 | 5 | 4 | 0.5152 | 0.3422 | 0.7798 | 0.5229 | 0.3221 | 0.4175 | 0.6982 | 0.2989 | 1.1169 | 0.8579 | 0.6252 | 0.2924 | 0.7311 | 0.5094 | 0.9204 | 0.6955 | 0.9908 | 0.9058 | 0.8472 | 0.8511 | 1.5996 | 0.3158 | 0.8872 | 0.4532 | 1.0765 | 0.8151 | 0.7870 | 0.6537 |
| 157 | Q13247 | Serine/arginine-rich splicing factor 6 (Pre-mRNA-splicing factor SRP55) (Splicing factor, arginine/serine-rich 6) | SRSF6 SFRS6 SRP55 | 4 | 5 | 0.8790 | 0.7084 | 0.6855 | 0.7535 | 0.9638 | 0.9255 | 0.5200 | 0.2987 | 0.9638 | 0.7175 | 0.8630 | 0.9127 | 0.8872 | 0.9522 | 0.6081 | 0.2622 | 1.0375 | 0.7600 | 0.7943 | 0.7709 | 1.1482 | 0.3531 | 0.8241 | 0.8958 | 0.9727 | 0.8520 | 0.9036 | 0.3652 |
| 158 | P84103 | Serine/arginine-rich splicing factor 3 (Pre-mRNA-splicing factor SRP20) (Splicing factor, arginine/serine-rich 3) | SRSF3 SFRS3 SRP20 | 6 | 7 | 1.1482 | 0.7810 | 0.3597 | 0.4869 | 2.0137 | 0.6812 | 0.5808 | 0.8576 | 2.1677 | 0.6310 | 0.9036 | 0.9588 | 1.4997 | 0.7151 | 0.4742 | 0.7995 | 2.5586 | 0.5940 | 0.8790 | 0.8991 | 3.5975 | 0.4205 | 1.1803 | 0.7471 | 2.0137 | 0.5157 | 0.6252 | 0.8039 |
| 159 | Q01130 | Serine/arginine-rich splicing factor 2 (Protein PR264) (Splicing component, 35 kDa) (Splicing factor SC35) (SC-35) (Splicing factor, arginine/serine-rich 2) | SRSF2 SFRS2 | 7 | 7 | 0.6368 | 0.4333 | 0.4786 | 0.1365 | 1.1376 | 0.9475 | 0.3565 | 0.0646 | 1.0000 | 0.7661 | 0.6668 | 0.4583 | 0.9550 | 0.5360 | 0.6668 | 0.1851 | 0.6730 | 0.2560 | 0.8395 | 0.2843 | 2.0701 | 0.2439 | 1.0471 | 0.8295 | 1.9409 | 0.3514 | 1.4454 | 0.6309 |
| 160 | Q07955 | Serine/arginine-rich splicing factor 1 (Alternative-splicing factor 1) (ASF-1) (Splicing factor, arginine/serine-rich 1) (pre-mRNA-splicing factor SF2, P33 subunit) | SRSF1 ASF SF2 SF2P33 SFRS1 OK/SW-cl.3 | 5 | 6 | 0.9462 | 0.8594 | 0.8710 | 0.5618 | 1.5136 | 0.3217 | 0.5445 | 0.0534 | 1.6144 | 0.4943 | 1.0568 | 0.6729 | 1.1169 | 0.8434 | 0.7656 | 0.3094 | 1.4191 | 0.4892 | 0.8241 | 0.6555 | 2.4210 | 0.0562 | 0.9290 | 0.5874 | 1.7219 | 0.0912 | 0.5861 | 0.2003 |
| 161 | Q9Y5M8 | Signal recognition particle receptor subunit beta (SR-beta) (Protein APMCF1) | SRPRB PSEC0230 | 3 | 4 | 1.3305 | 0.4539 | 0.9817 | 0.7567 | 0.9638 | 0.8184 | 1.0568 | 0.0421 | 1.6749 | 0.1282 | 0.7244 | 0.0192 | 1.8197 | 0.1575 | 0.9727 | 0.8095 | 1.2474 | 0.4592 | 1.3183 | 0.1565 | 1.6904 | 0.1366 | 0.8630 | 0.0186 | 1.5849 | 0.2426 | 0.8091 | 0.0569 |
| 162 | P08240 | Signal recognition particle receptor subunit alpha (SR-alpha) (Docking protein alpha) (DP-alpha) | SRPR | 7 | 10 | 1.2706 | 0.5375 | 1.0186 | 0.7643 | 0.8630 | 0.7330 | 0.9817 | 0.9433 | 0.6368 | 0.4155 | 0.9817 | 0.9357 | 0.9727 | 0.9188 | 1.1272 | 0.1215 | 0.5649 | 0.3911 | 1.1695 | 0.5763 | 0.8551 | 0.4102 | 0.9908 | 0.9865 | 0.7178 | 0.2902 | 0.9638 | 0.6506 |
| 163 | Q9UHB9 | Signal recognition particle subunit SRP68 (SRP68) (Signal recognition particle 68 kDa protein) | SRP68 | 3 | 5 | 1.4322 | 0.1340 | 1.1066 | 0.6459 | 1.2589 | 0.2178 | 0.8630 | 0.4581 | 1.0666 | 0.7930 | 0.9638 | 0.9189 | 1.5136 | 0.1063 | 1.0280 | 0.8333 | 1.1272 | 0.4418 | 0.9908 | 0.7601 | 1.0666 | 0.5416 | 1.0965 | 0.8458 | 1.1482 | 0.3655 | 0.8954 | 0.6251 |
| 164 | P37108 | Signal recognition particle 14 kDa protein (SRP14) (18 kDa Alu RNA-binding protein) | SRP14 | 3 | 3 | 1.4322 | 0.2780 | 1.2246 | 0.5227 | 1.6749 | 0.0978 | 1.6144 | 0.4021 | 1.5417 | 0.2676 | 2.0137 | 0.2300 | 1.7219 | 0.1152 | 1.2942 | 0.5345 | 1.8535 | 0.1123 | 1.8030 | 0.2255 | 1.6904 | 0.0432 | 1.9409 | 0.3831 | 1.7219 | 0.3335 | 2.2284 | 0.1425 |
| 165 | Q9Y6N5 | Sulfide:quinone oxidoreductase, mitochondrial (SQOR) (EC 1.8.5.-) | SQRDL CGI-44 | 12 | 14 | 0.5058 | 0.1740 | 1.7061 | 0.0652 | 1.5560 | 0.0922 | 0.6310 | 0.0624 | 0.3908 | 0.0094 | 2.1677 | 0.0005 | 1.2474 | 0.0721 | 1.6444 | 0.0977 | 0.4699 | 0.0282 | 1.0375 | 0.6537 | 1.1803 | 0.2458 | 0.9908 | 0.9052 | 1.3677 | 0.1572 | 1.0568 | 0.9493 |
| 166 | O15020 | Spectrin beta chain, non-erythrocytic 2 (Beta-III spectrin) (Spinocerebellar ataxia 5 protein) | SPTBN2 KIAA0302 SCA5 | 16 | 26 | 2.3121 | 0.1889 | 0.9204 | 0.9770 | 0.9817 | 0.5236 | 0.8395 | 0.7952 | 2.3335 | 0.1991 | 0.3133 | 0.1070 | 1.2359 | 0.5828 | 0.9462 | 0.8214 | 1.4060 | 0.3969 | 0.5248 | 0.3449 | 0.5970 | 0.8562 | 0.6668 | 0.0873 | 1.9588 | 0.2369 | 0.7112 | 0.1829 |
| 167 | Q01082 | Spectrin beta chain, non-erythrocytic 1 (Beta-II spectrin) (Fodrin beta chain) (Spectrin, non-erythroid beta chain 1) | SPTBN1 SPTB2 | 91 | 92 | 1.2823 | 0.7726 | 1.3804 | 0.5052 | 1.9409 | 0.4452 | 1.3677 | 0.8397 | 1.3183 | 0.8365 | 1.4060 | 0.5028 | 1.2706 | 0.7827 | 1.5560 | 0.6130 | 1.1482 | 0.9897 | 1.8880 | 0.2948 | 0.9376 | 0.8670 | 1.8197 | 0.3501 | 1.0000 | 0.5505 | 1.0864 | 0.9519 |
| 168 | Q13813 | Spectrin alpha chain, non-erythrocytic 1 (Alpha-II spectrin) (Fodrin alpha chain) (Spectrin, non-erythroid alpha subunit) | SPTAN1 NEAS SPTA2 | 127 | 123 | 0.7943 | 0.0793 | 0.7447 | 0.1077 | 1.4454 | 0.3758 | 0.8395 | 0.5191 | 1.0864 | 0.4921 | 1.6144 | 0.0008 | 1.0375 | 0.4977 | 0.6607 | 0.0698 | 1.3183 | 0.4323 | 1.0186 | 0.8216 | 0.9638 | 0.1470 | 1.6749 | 0.0001 | 0.5495 | 0.0000 | 0.9817 | 0.7443 |
| 169 | Q8WW59 | SPRY domain-containing protein 4 | SPRYD4 | 18 | 14 | 1.1066 | 0.8003 | 1.5704 | 0.8338 | 0.9638 | 0.7451 | 1.5560 | 0.3635 | 0.8318 | 0.3189 | 1.0186 | 0.4839 | 1.5276 | 0.2096 | 1.3305 | 0.7105 | 1.0864 | 0.8995 | 1.1169 | 0.6196 | 0.4966 | 0.0072 | 1.2942 | 0.4962 | 0.6855 | 0.0448 | 0.5754 | 0.1249 |
| 170 | P35270 | Sepiapterin reductase (SPR) (EC 1.1.1.153) | SPR | 12 | 11 | 0.5200 | 0.3184 | 1.0568 | 0.4810 | 1.4191 | 0.8490 | 2.0701 | 0.2969 | 1.0186 | 0.5362 | 1.1066 | 0.3445 | 1.9770 | 0.3314 | 1.7539 | 0.3675 | 1.1066 | 0.6433 | 1.0568 | 0.9365 | 0.1169 | 0.0016 | 1.4060 | 0.7257 | 0.7244 | 0.2797 | 0.9462 | 0.3630 |
| 171 | Q15005 | Signal peptidase complex subunit 2 (EC 3.4.-.-) (Microsomal signal peptidase 25 kDa subunit) (SPase 25 kDa subunit) | SPCS2 KIAA0102 SPC25 | 4 | 3 | 0.8472 | 0.8067 | 1.4588 | 0.3994 | 0.7447 | 0.5805 | 1.3183 | 0.5067 | 0.6486 | 0.5079 | 1.1169 | 0.7085 | 0.9638 | 0.8951 | 1.2823 | 0.5778 | 0.8790 | 0.8037 | 1.3062 | 0.5475 | 0.9120 | 0.8149 | 1.1695 | 0.7068 | 0.8241 | 0.6338 | 2.0701 | 0.3036 |
| 172 | Q00796 | Sorbitol dehydrogenase (EC 1.1.1.14) (L-iditol 2-dehydrogenase) | SORD | 51 | 51 | 1.1588 | 0.6678 | 1.3677 | 0.6929 | 1.1912 | 0.7427 | 2.6062 | 0.0153 | 1.3305 | 0.1570 | 1.2474 | 0.8030 | 1.0375 | 0.3253 | 2.1086 | 0.0958 | 1.2823 | 0.9527 | 0.9727 | 0.1746 | 0.0425 | 0.0000 | 1.0765 | 0.5647 | 0.1127 | 0.0000 | 0.1629 | 0.0004 |
| 173 | O60504 | Vinexin (SH3-containing adapter molecule 1) (SCAM-1) (Sorbin and SH3 domain-containing protein 3) | SORBS3 SCAM1 | 3 | 3 | 0.5152 | 0.3111 | 0.8395 | 0.5180 | 0.9908 | 0.8299 | 1.0568 | 0.9997 | 1.3183 | 0.4841 | 0.9204 | 0.6385 | 0.1941 | 0.2224 | 0.8630 | 0.4534 | 1.2023 | 0.8452 | 0.9462 | 0.5408 | 1.3062 | 0.5375 | 0.8241 | 0.4525 | 1.0093 | 0.9887 | 1.0471 | 0.8236 |
| 174 | O94875 | Sorbin and SH3 domain-containing protein 2 (Arg-binding protein 2) (ArgBP2) (Arg/Abl-interacting protein 2) (Sorbin) | SORBS2 ARGBP2 KIAA0777 | 8 | 9 | 1.2706 | 0.6622 | 0.8318 | 0.9071 | 1.1695 | 0.2726 | 0.9550 | 0.8082 | 1.3183 | 0.3687 | 0.7244 | 0.8943 | 1.8030 | 0.1352 | 0.7943 | 0.6521 | 1.1272 | 0.9407 | 1.1272 | 0.3397 | 0.9204 | 0.7058 | 1.9770 | 0.0234 | 1.2942 | 0.9585 | 0.5649 | 0.5198 |
| 175 | Q9BX66 | Sorbin and SH3 domain-containing protein 1 (Ponsin) (SH3 domain protein 5) (SH3P12) (c-Cbl-associated protein) (CAP) | SORBS1 KIAA0894 KIAA1296 SH3D5 | 12 | 15 | 1.0375 | 0.8983 | 1.2942 | 0.5043 | 1.4859 | 0.1301 | 1.5849 | 0.3077 | 1.0965 | 0.1036 | 0.7178 | 0.7559 | 1.1912 | 0.1207 | 1.2706 | 0.6002 | 0.8395 | 0.8829 | 1.2246 | 0.8791 | 0.6546 | 0.4905 | 1.0568 | 0.8948 | 0.7870 | 0.9808 | 1.2474 | 0.8255 |
| 176 | P04179 | Superoxide dismutase [Mn], mitochondrial (EC 1.15.1.1) | SOD2 | 23 | 24 | 0.6730 | 0.0975 | 0.4246 | 0.0010 | 0.4966 | 0.0175 | 0.5445 | 0.0006 | 0.3076 | 0.0002 | 0.4699 | 0.0031 | 1.5560 | 0.5078 | 0.2291 | 0.0000 | 0.7311 | 0.0432 | 0.9036 | 0.6416 | 1.1482 | 0.5181 | 0.7870 | 0.0268 | 0.3221 | 0.0002 | 0.6792 | 0.0594 |
| 177 | P00441 | Superoxide dismutase [Cu-Zn] (EC 1.15.1.1) (Superoxide dismutase 1) (hSod1) | SOD1 | 36 | 34 | 4.9204 | 0.0004 | 14.0605 | 0.0050 | 3.4041 | 0.0068 | 17.2187 | 0.0008 | 4.4463 | 0.0001 | 13.4277 | 0.0019 | 6.0256 | 0.0001 | 17.2187 | 0.0154 | 4.6559 | 0.0008 | 14.4544 | 0.0136 | 0.9817 | 0.3937 | 15.9956 | 0.0008 | 1.0666 | 0.8932 | 6.2517 | 0.2666 |
| 178 | Q9Y5X3 | Sorting nexin-5 | SNX5 | 6 | 3 | 1.0000 | 0.8147 | 1.1376 | 0.2646 | 1.2134 | 0.4107 | 0.9462 | 0.6989 | 1.0000 | 0.8390 | 1.0093 | 0.9928 | 1.0093 | 0.8029 | 1.0093 | 0.8607 | 1.2023 | 0.3524 | 1.1376 | 0.1902 | 1.3305 | 0.3506 | 0.9204 | 0.6346 | 0.9550 | 0.9998 | 1.0280 | 0.8260 |
| 179 | O60493 | Sorting nexin-3 (Protein SDP3) | SNX3 | 5 | 4 | 1.7061 | 0.6947 | 0.9550 | 0.7024 | 2.6303 | 0.1736 | 0.8710 | 0.8362 | 1.8707 | 0.2167 | 1.0765 | 0.2795 | 1.8535 | 0.4306 | 0.9204 | 0.8954 | 1.6144 | 0.6258 | 1.0965 | 0.2832 | 2.1281 | 0.1635 | 0.8790 | 0.7803 | 1.9588 | 0.3352 | 1.0666 | 0.5792 |
| 180 | O60749 | Sorting nexin-2 (Transformation-related gene 9 protein) (TRG-9) | SNX2 TRG9 | 5 | 9 | 0.7516 | 0.6182 | 1.1482 | 0.8272 | 0.7586 | 0.3248 | 0.9550 | 0.9757 | 1.0186 | 0.7292 | 1.2589 | 0.7273 | 0.8630 | 0.6115 | 1.2246 | 0.9530 | 0.7516 | 0.2564 | 0.9817 | 0.4389 | 1.3062 | 0.3008 | 1.6596 | 0.4362 | 1.1272 | 0.4936 | 0.8395 | 0.9371 |
| 181 | Q13596 | Sorting nexin-1 | SNX1 | 6 | 4 | 0.9462 | 0.8640 | 0.8166 | 0.6976 | 1.5849 | 0.1341 | 0.6607 | 0.4641 | 1.7865 | 0.0950 | 0.9727 | 0.7626 | 1.2823 | 0.2807 | 0.7727 | 0.5297 | 1.0000 | 0.9854 | 0.7178 | 0.3575 | 1.2589 | 0.2988 | 0.6855 | 0.4921 | 1.2023 | 0.3741 | 0.7586 | 0.5293 |
| 182 | Q13884 | Beta-1-syntrophin (59 kDa dystrophin-associated protein A1 basic component 1) (DAPA1B) (BSYN2) (Syntrophin-2) (Tax interaction protein 43) (TIP-43) | SNTB1 SNT2B1 | 13 | 11 | 0.5248 | 0.4219 | 0.9120 | 0.3926 | 0.9462 | 0.9340 | 1.0000 | 0.9335 | 0.8790 | 0.8166 | 0.9638 | 0.4304 | 0.6546 | 0.3791 | 0.9550 | 0.5438 | 1.1169 | 0.5723 | 0.9638 | 0.5396 | 0.8790 | 0.5971 | 1.0093 | 0.7111 | 0.4613 | 0.1924 | 0.9462 | 0.4698 |
| 183 | P14678 | Small nuclear ribonucleoprotein-associated proteins B and B' (snRNP-B) (Sm protein B/B') (Sm-B/B') (SmB/B') | SNRPB COD SNRPB1 | 3 | 3 | 0.8630 | 0.6717 | 0.8318 | 0.0300 | 0.8630 | 0.8988 | 1.0471 | 0.0753 | 0.9204 | 0.8684 | 1.0000 | 0.1132 | 1.1482 | 0.9850 | 1.0471 | 0.1020 | 1.4191 | 0.6409 | 1.0666 | 0.0439 | 2.1281 | 0.2141 | 1.0471 | 0.0876 | 1.4322 | 0.6697 | 0.9817 | 0.1257 |
| 184 | P09661 | U2 small nuclear ribonucleoprotein A' (U2 snRNP A') | SNRPA1 | 3 | 3 | 1.0471 | 0.4022 | 0.7447 | 0.3731 | 1.4859 | 0.2083 | 0.8091 | 0.3151 | 1.3428 | 0.4997 | 0.9462 | 0.8487 | 1.0965 | 0.5679 | 1.0186 | 0.8919 | 0.8241 | 0.8241 | 0.9550 | 0.8127 | 2.0893 | 0.0518 | 0.8630 | 0.3142 | 1.2134 | 0.3080 | 1.0280 | 0.7539 |
| 185 | P08621 | U1 small nuclear ribonucleoprotein 70 kDa (U1 snRNP 70 kDa) (U1-70K) (snRNP70) | SNRNP70 RNPU1Z RPU1 SNRP70 U1AP1 | 4 | 4 | 3.7670 | 0.0873 | 0.7047 | 0.0194 | 1.9770 | 0.5610 | 0.8710 | 0.3942 | 1.2134 | 0.7744 | 0.8630 | 0.4786 | 1.2246 | 0.8851 | 0.7798 | 0.1393 | 1.2589 | 0.6836 | 0.7379 | 0.1389 | 3.5318 | 0.2250 | 0.7943 | 0.2517 | 4.4055 | 0.1692 | 0.7379 | 0.1344 |
| 186 | O75643 | U5 small nuclear ribonucleoprotein 200 kDa helicase (EC 3.6.4.13) (Activating signal cointegrator 1 complex subunit 3-like 1) (BRR2 homolog) (U5 snRNP-specific 200 kDa protein) (U5-200KD) | SNRNP200 ASCC3L1 HELIC2 KIAA0788 | 6 | 14 | 0.7656 | 0.4455 | 0.8954 | 0.9588 | 0.9727 | 0.7967 | 0.5495 | 0.2386 | 0.9817 | 0.8685 | 1.1066 | 0.5307 | 0.5445 | 0.1796 | 0.8091 | 0.6298 | 1.2474 | 0.4779 | 1.0186 | 0.9453 | 1.1803 | 0.6145 | 1.0471 | 0.6156 | 1.3183 | 0.4394 | 0.8091 | 0.8417 |
| 187 | P61247 | 40S ribosomal protein S3a (v-fos transformation effector protein) (Fte-1) | RPS3A FTE1 MFTL | 19 | 15 | 1.0280 | 0.4758 | 0.8630 | 0.6013 | 1.7219 | 0.4124 | 0.8472 | 0.8195 | 1.1169 | 0.6760 | 1.3183 | 0.4122 | 1.9953 | 0.3235 | 0.9908 | 0.9063 | 1.2023 | 0.8513 | 1.2823 | 0.4971 | 2.6546 | 0.0039 | 1.2942 | 0.7560 | 1.4454 | 0.9880 | 1.7378 | 0.0369 |
| 188 | P62750 | 60S ribosomal protein L23a | RPL23A | 8 | 8 | 1.3183 | 0.5257 | 1.2474 | 0.2392 | 1.2823 | 0.4438 | 0.8790 | 0.7236 | 2.0512 | 0.0409 | 1.7865 | 0.0867 | 2.2080 | 0.0497 | 0.9550 | 0.1796 | 1.4723 | 0.2466 | 1.6444 | 0.0515 | 1.7701 | 0.0276 | 1.8707 | 0.0285 | 1.1695 | 0.6092 | 1.9588 | 0.0355 |
| 189 | P40429 | 60S ribosomal protein L13a (23 kDa highly basic protein) | RPL13A | 5 | 6 | 1.6293 | 0.5844 | 1.2246 | 0.8610 | 1.1695 | 0.6587 | 1.1272 | 0.8165 | 1.1169 | 0.6123 | 0.8241 | 0.9875 | 1.7701 | 0.1464 | 0.8241 | 0.5873 | 2.9107 | 0.0159 | 0.8790 | 0.9051 | 3.0761 | 0.0139 | 1.0280 | 0.8122 | 3.4041 | 0.0104 | 2.1878 | 0.0336 |
| 190 | P62424 | 60S ribosomal protein L7a (PLA-X polypeptide) (Surfeit locus protein 3) | RPL7A SURF-3 SURF3 | 15 | 15 | 2.4660 | 0.6160 | 1.2474 | 0.8396 | 1.8197 | 0.8908 | 1.7219 | 0.4152 | 3.1623 | 0.0601 | 1.7378 | 0.1761 | 2.9376 | 0.1083 | 1.9409 | 0.6090 | 2.1478 | 0.2860 | 2.2699 | 0.1921 | 4.0179 | 0.0022 | 3.3729 | 0.0180 | 1.9588 | 0.6309 | 2.9923 | 0.0157 |
| 191 | P46777 | 60S ribosomal protein L5 | RPL5 MSTP030 | 8 | 5 | 0.4169 | 0.1067 | 0.6607 | 0.1212 | 0.9550 | 0.9979 | 0.7586 | 0.2135 | 0.7178 | 0.9256 | 0.9290 | 0.5280 | 1.1169 | 0.2758 | 0.6138 | 0.2263 | 0.8017 | 0.8505 | 1.0666 | 0.7537 | 1.4060 | 0.0907 | 0.8954 | 0.4005 | 0.8318 | 0.7525 | 1.0375 | 0.6208 |
| 192 | O00567 | Nucleolar protein 56 (Nucleolar protein 5A) | NOP56 NOL5A | 3 | 5 | 2.5823 | 0.2039 | 2.4889 | 0.0319 | 3.4356 | 0.0248 | 3.1623 | 0.0113 | 1.0186 | 0.9157 | 3.1046 | 0.0063 | 2.6792 | 0.0527 | 1.7865 | 0.0599 | 2.1086 | 0.1550 | 2.7542 | 0.0420 | 1.9055 | 0.1993 | 2.9107 | 0.0073 | 2.3335 | 0.1573 | 2.0701 | 0.0337 |
| 193 | P24534 | Elongation factor 1-beta (EF-1-beta) | EEF1B2 EEF1B EF1B | 10 | 7 | 0.8017 | 0.8385 | 1.6144 | 0.6104 | 0.6310 | 0.2563 | 0.9638 | 0.9358 | 1.1376 | 0.7091 | 2.5586 | 0.2879 | 1.3428 | 0.4021 | 1.4723 | 0.9274 | 0.8472 | 0.8710 | 2.4660 | 0.2558 | 1.1482 | 0.5596 | 1.8535 | 0.5549 | 1.1695 | 0.5862 | 2.9648 | 0.2210 |
| 194 | Q7KZF4 | Staphylococcal nuclease domain-containing protein 1 (100 kDa coactivator) (EBNA2 coactivator p100) (Tudor domain-containing protein 11) (p100 co-activator) | SND1 TDRD11 | 50 | 46 | 1.0965 | 0.7519 | 1.5276 | 0.1855 | 1.7378 | 0.2244 | 1.5417 | 0.2239 | 1.5704 | 0.0570 | 1.8707 | 0.1072 | 2.0893 | 0.0201 | 1.7378 | 0.3275 | 1.1912 | 0.7816 | 1.8880 | 0.0053 | 1.8197 | 0.0152 | 2.6792 | 0.0005 | 2.5351 | 0.0010 | 2.3988 | 0.0035 |
| 195 | Q15599 | Na(+)/H(+) exchange regulatory cofactor NHE-RF2 (NHERF-2) (NHE3 kinase A regulatory protein E3KARP) (SRY-interacting protein 1) (SIP-1) (Sodium-hydrogen exchanger regulatory factor 2) (Solute carrier family 9 isoform A3 regulatory factor 2) (Tyrosine kinase activator protein 1) (TKA-1) | SLC9A3R2 NHERF2 | 3 | 4 | 1.0666 | 0.8585 | 1.1588 | 0.8144 | 1.0471 | 0.9261 | 1.7219 | 0.4492 | 1.0666 | 0.7031 | 0.8241 | 0.8245 | 1.0765 | 0.7637 | 1.4997 | 0.5587 | 1.3183 | 0.6544 | 0.8630 | 0.8068 | 1.0093 | 0.8863 | 1.5136 | 0.6401 | 1.2134 | 0.7641 | 0.7244 | 0.8963 |
| 196 | O14745 | Na(+)/H(+) exchange regulatory cofactor NHE-RF1 (NHERF-1) (Ezrin-radixin-moesin-binding phosphoprotein 50) (EBP50) (Regulatory cofactor of Na(+)/H(+) exchanger) (Sodium-hydrogen exchanger regulatory factor 1) (Solute carrier family 9 isoform A3 regulatory factor 1) | SLC9A3R1 NHERF NHERF1 | 8 | 7 | 1.8880 | 0.0572 | 1.1695 | 0.5640 | 1.3804 | 0.6001 | 1.3804 | 0.1578 | 1.5704 | 0.3116 | 2.1086 | 0.0977 | 1.5560 | 0.4620 | 1.5417 | 0.4168 | 1.1482 | 0.5149 | 1.8707 | 0.1975 | 1.6144 | 0.1178 | 1.4322 | 0.2160 | 1.6749 | 0.4671 | 1.5560 | 0.3640 |
| 197 | P02730 | Band 3 anion transport protein (Anion exchange protein 1) (AE 1) (Anion exchanger 1) (Solute carrier family 4 member 1) (CD antigen CD233) | SLC4A1 AE1 DI EPB3 | 7 | 11 | 0.9550 | 0.7059 | 0.8166 | 0.7078 | 1.3552 | 0.6554 | 0.4875 | 0.0803 | 2.4434 | 0.1092 | 0.8872 | 0.9622 | 0.1629 | 0.0338 | 0.9908 | 0.7203 | 0.7178 | 0.5001 | 0.4406 | 0.0954 | 0.3342 | 0.1118 | 0.7112 | 0.4217 | 0.6427 | 0.5235 | 0.1330 | 0.0103 |
| 198 | Q15043 | Zinc transporter ZIP14 (LIV-1 subfamily of ZIP zinc transporter 4) (LZT-Hs4) (Solute carrier family 39 member 14) (Zrt- and Irt-like protein 14) (ZIP-14) | SLC39A14 KIAA0062 ZIP14 | 4 | 4 | 1.0093 | 0.6075 | 0.6918 | 0.6441 | 0.8472 | 0.2623 | 1.1066 | 0.6763 | 1.0093 | 0.5611 | 1.1695 | 0.6894 | 1.0186 | 0.7963 | 0.3342 | 0.2289 | 1.1169 | 0.6708 | 0.8166 | 0.9993 | 0.9462 | 0.7498 | 0.9908 | 0.8739 | 1.0093 | 0.6222 | 0.3251 | 0.3677 |
| 199 | P11168 | Solute carrier family 2, facilitated glucose transporter member 2 (Glucose transporter type 2, liver) (GLUT-2) | SLC2A2 GLUT2 | 12 | 5 | 2.0324 | 0.4029 | 2.4660 | 0.9249 | 1.2359 | 0.8959 | 1.2134 | 0.2061 | 1.8365 | 0.2486 | 2.5119 | 0.3946 | 0.7244 | 0.4757 | 2.0324 | 0.5861 | 1.4060 | 0.2005 | 2.6546 | 0.5019 | 0.1871 | 0.4837 | 1.9588 | 0.3430 | 0.4365 | 0.9695 | 0.2965 | 0.0357 |
| 200 | Q9Y2P5 | Bile acyl-CoA synthetase (BACS) (EC 6.2.1.7) (Bile acid-CoA ligase) (BA-CoA ligase) (BAL) (Cholate--CoA ligase) (Fatty acid transport protein 5) (FATP-5) (Fatty-acid-coenzyme A ligase, very long-chain 3) (Solute carrier family 27 member 5) (Very long-chain acyl-CoA synthetase homolog 2) (VLCS-H2) (VLCSH2) (Very long-chain acyl-CoA synthetase-related protein) (VLACS-related) (VLACSR) | SLC27A5 ACSB ACSVL6 FACVL3 FATP5 | 42 | 43 | 1.7865 | 0.0019 | 1.5560 | 0.0659 | 1.1588 | 0.2146 | 2.2491 | 0.0003 | 1.0568 | 0.2131 | 0.6138 | 0.0208 | 1.1588 | 0.1911 | 1.1588 | 0.6765 | 1.2023 | 0.0962 | 0.4285 | 0.0015 | 0.1038 | 0.0000 | 0.8630 | 0.3254 | 0.3020 | 0.0005 | 0.1500 | 0.0000 |
| 201 | O14975 | Very long-chain acyl-CoA synthetase (VLACS) (VLCS) (EC 6.2.1.-) (Fatty acid transport protein 2) (FATP-2) (Fatty-acid-coenzyme A ligase, very long-chain 1) (Long-chain-fatty-acid--CoA ligase) (EC 6.2.1.3) (Solute carrier family 27 member 2) (THCA-CoA ligase) (Very long-chain-fatty-acid-CoA ligase) | SLC27A2 ACSVL1 FACVL1 FATP2 VLACS | 17 | 18 | 2.0701 | 0.0326 | 0.9817 | 0.4724 | 0.8472 | 0.6930 | 1.3804 | 0.1513 | 1.1588 | 0.4262 | 1.2823 | 0.5404 | 1.8535 | 0.0749 | 0.8166 | 0.2620 | 1.5704 | 0.1865 | 1.1169 | 0.9027 | 0.9120 | 0.4426 | 0.9204 | 0.5569 | 0.4406 | 0.2317 | 0.3664 | 0.0047 |
| 202 | P12236 | ADP/ATP translocase 3 (ADP,ATP carrier protein 3) (ADP,ATP carrier protein, isoform T2) (ANT 2) (Adenine nucleotide translocator 3) (ANT 3) (Solute carrier family 25 member 6) [Cleaved into: ADP/ATP translocase 3, N-terminally processed] | SLC25A6 ANT3 CDABP0051 | 20 | 17 | 0.9638 | 0.9660 | 1.9953 | 0.3764 | 1.1066 | 0.8317 | 1.5136 | 0.5633 | 0.9462 | 0.8611 | 1.1912 | 0.9131 | 1.1482 | 0.7498 | 1.1803 | 0.8575 | 1.0093 | 0.9373 | 1.9953 | 0.3921 | 1.0864 | 0.7766 | 1.1482 | 0.9732 | 1.1066 | 0.7629 | 2.2284 | 0.3429 |
| 203 | P05141 | ADP/ATP translocase 2 (ADP,ATP carrier protein 2) (ADP,ATP carrier protein, fibroblast isoform) (Adenine nucleotide translocator 2) (ANT 2) (Solute carrier family 25 member 5) [Cleaved into: ADP/ATP translocase 2, N-terminally processed] | SLC25A5 ANT2 | 30 | 24 | 2.3121 | 0.9639 | 1.4191 | 0.7698 | 1.5560 | 0.5213 | 1.2706 | 0.6526 | 1.7061 | 0.4946 | 1.2023 | 0.6095 | 2.6062 | 0.7492 | 1.2134 | 0.7767 | 2.5351 | 0.8193 | 1.3062 | 0.5470 | 1.8707 | 0.9101 | 1.2134 | 0.6256 | 1.8365 | 0.7556 | 1.3552 | 0.4209 |
| 204 | P12235 | ADP/ATP translocase 1 (ADP,ATP carrier protein 1) (ADP,ATP carrier protein, heart/skeletal muscle isoform T1) (Adenine nucleotide translocator 1) (ANT 1) (Solute carrier family 25 member 4) | SLC25A4 ANT1 | 21 | 16 | 0.9550 | 0.9171 | 0.8166 | 0.7829 | 0.4169 | 0.2054 | 1.0864 | 0.9664 | 0.4920 | 0.2897 | 0.7727 | 0.6401 | 0.8551 | 0.7316 | 0.2729 | 0.3898 | 1.2589 | 0.6292 | 1.1272 | 0.9922 | 0.3532 | 0.3264 | 0.6546 | 0.5840 | 0.6668 | 0.5992 | 1.2942 | 0.8295 |
| 205 | Q00325 | Phosphate carrier protein, mitochondrial (Phosphate transport protein) (PTP) (Solute carrier family 25 member 3) | SLC25A3 PHC OK/SW-cl.48 | 10 | 11 | 0.8872 | 0.9695 | 0.3733 | 0.0474 | 0.7943 | 0.1319 | 0.6855 | 0.0627 | 0.5598 | 0.0892 | 0.2606 | 0.0006 | 0.8395 | 0.6674 | 0.6427 | 0.0190 | 0.8954 | 0.8603 | 0.8241 | 0.3410 | 1.2942 | 0.3320 | 0.7727 | 0.1254 | 0.8551 | 0.5656 | 1.2134 | 0.7844 |
| 206 | O43772 | Mitochondrial carnitine/acylcarnitine carrier protein (Carnitine/acylcarnitine translocase) (CAC) (Solute carrier family 25 member 20) | SLC25A20 CAC CACT | 13 | 14 | 0.8091 | 0.7063 | 0.5808 | 0.6562 | 0.6138 | 0.1254 | 0.8630 | 0.1420 | 0.3048 | 0.0024 | 0.3076 | 0.2370 | 0.7943 | 0.6479 | 0.7943 | 0.8142 | 0.8395 | 0.3240 | 1.0666 | 0.6507 | 0.3733 | 0.0171 | 0.4831 | 0.5577 | 0.4487 | 0.0011 | 0.3221 | 0.1527 |
| 207 | Q9H1K4 | Mitochondrial glutamate carrier 2 (GC-2) (Glutamate/H(+) symporter 2) (Solute carrier family 25 member 18) | SLC25A18 GC2 | 6 | 8 | 0.6081 | 0.3891 | 0.4742 | 0.3423 | 0.5808 | 0.5186 | 1.3428 | 0.5985 | 0.4130 | 0.2277 | 0.4325 | 0.3433 | 0.7311 | 0.6454 | 0.2805 | 0.1898 | 0.7798 | 0.5750 | 0.4742 | 0.3347 | 0.8091 | 0.7507 | 0.6252 | 0.4352 | 0.9036 | 0.7995 | 0.8954 | 0.7227 |
| 208 | Q9Y619 | Mitochondrial ornithine transporter 1 (Solute carrier family 25 member 15) | SLC25A15 ORNT1 SP1855 | 5 | 6 | 1.6144 | 0.3144 | 0.2992 | 0.0310 | 1.2706 | 0.5688 | 0.7798 | 0.4425 | 0.5012 | 0.5432 | 0.5598 | 0.2427 | 1.7865 | 0.1250 | 1.2589 | 0.7226 | 1.1803 | 0.5652 | 0.7656 | 0.6056 | 0.6730 | 0.7514 | 0.6368 | 0.4019 | 0.4325 | 0.7393 | 1.0864 | 0.7969 |
| 209 | Q9UJS0 | Calcium-binding mitochondrial carrier protein Aralar2 (Citrin) (Mitochondrial aspartate glutamate carrier 2) (Solute carrier family 25 member 13) | SLC25A13 ARALAR2 | 46 | 40 | 1.3428 | 0.4062 | 1.6144 | 0.1061 | 1.0186 | 0.5488 | 1.1272 | 0.2318 | 0.9036 | 0.2480 | 1.3183 | 0.6645 | 1.1803 | 0.8156 | 1.8880 | 0.6180 | 1.3062 | 0.3756 | 1.8535 | 0.0530 | 0.2559 | 0.0000 | 1.0965 | 0.2637 | 0.5546 | 0.0000 | 0.6486 | 0.1200 |
| 210 | Q02978 | Mitochondrial 2-oxoglutarate/malate carrier protein (OGCP) (Solute carrier family 25 member 11) | SLC25A11 SLC20A4 | 8 | 10 | 0.9290 | 0.5720 | 0.3048 | 0.0003 | 0.6792 | 0.2765 | 0.6427 | 0.0647 | 0.4571 | 0.2101 | 0.5248 | 0.1323 | 1.6144 | 0.4252 | 0.7112 | 0.6050 | 1.4859 | 0.7196 | 0.7244 | 0.8738 | 0.8954 | 0.2596 | 0.7047 | 0.2216 | 0.7447 | 0.2084 | 0.3532 | 0.0470 |
| 211 | Q9UBX3 | Mitochondrial dicarboxylate carrier (Solute carrier family 25 member 10) | SLC25A10 DIC | 9 | 11 | 1.6749 | 0.4792 | 0.6252 | 0.1549 | 1.4454 | 0.5854 | 0.9290 | 0.6371 | 0.9727 | 0.7135 | 1.3552 | 0.6756 | 0.9817 | 0.6540 | 1.3677 | 0.7026 | 1.7061 | 0.4006 | 0.9120 | 0.4847 | 0.5702 | 0.7353 | 1.4322 | 0.6936 | 0.4966 | 0.7008 | 0.8241 | 0.7400 |
| 212 | Q9Y694 | Solute carrier family 22 member 7 (Novel liver transporter) (Organic anion transporter 2) (hOAT2) | SLC22A7 NLT OAT2 | 8 | 4 | 0.9290 | 0.5720 | 0.6486 | 0.4412 | 0.6792 | 0.2765 | 0.3945 | 0.2262 | 0.4571 | 0.2101 | 1.2823 | 0.6203 | 1.6144 | 0.4252 | 1.1695 | 0.7370 | 1.4859 | 0.7196 | 0.7798 | 0.6355 | 0.8954 | 0.2596 | 0.7244 | 0.5301 | 0.7447 | 0.2084 | 1.0375 | 0.9258 |
| 213 | P63208 | S-phase kinase-associated protein 1 (Cyclin-A/CDK2-associated protein p19) (p19A) (Organ of Corti protein 2) (OCP-2) (Organ of Corti protein II) (OCP-II) (RNA polymerase II elongation factor-like protein) (SIII) (Transcription elongation factor B polypeptide 1-like) (p19skp1) | SKP1 EMC19 OCP2 SKP1A TCEB1L | 9 | 7 | 1.6749 | 0.4792 | 1.0093 | 0.3442 | 1.4454 | 0.5854 | 0.5546 | 0.4018 | 0.9727 | 0.7135 | 1.7219 | 0.3243 | 0.9817 | 0.6540 | 1.3428 | 0.7241 | 1.7061 | 0.4006 | 0.5598 | 0.4445 | 0.5702 | 0.7353 | 0.9290 | 0.9631 | 0.4966 | 0.7008 | 1.5560 | 0.3566 |
| 214 | Q99720 | Sigma non-opioid intracellular receptor 1 (Aging-associated gene 8 protein) (SR31747-binding protein) (SR-BP) (Sigma 1-type opioid receptor) (SIG-1R) (Sigma1-receptor) (Sigma1R) (hSigmaR1) | SIGMAR1 OPRS1 SRBP AAG8 | 4 | 4 | 0.9376 | 0.8509 | 0.9036 | 0.8681 | 0.8551 | 0.8632 | 1.5704 | 0.4740 | 1.2474 | 0.3294 | 0.9462 | 0.8926 | 1.0186 | 0.7086 | 1.3183 | 0.5912 | 1.0471 | 0.6726 | 1.0864 | 0.9044 | 0.9462 | 0.8820 | 1.4060 | 0.5716 | 1.0375 | 0.8235 | 1.1066 | 0.8654 |
| 215 | Q9UHJ6 | Sedoheptulokinase (SHK) (EC 2.7.1.14) (Carbohydrate kinase-like protein) | SHPK CARKL | 8 | 7 | 0.4018 | 0.5231 | 3.7670 | 0.2795 | 0.5152 | 0.0836 | 3.0479 | 0.3848 | 0.6081 | 0.7867 | 2.2080 | 0.5054 | 0.5546 | 0.6135 | 2.4660 | 0.2454 | 0.9817 | 0.9630 | 3.1915 | 0.3055 | 1.0568 | 0.8537 | 2.3121 | 0.6200 | 1.0280 | 0.6010 | 1.4060 | 0.6075 |
| 216 | P34897 | Serine hydroxymethyltransferase, mitochondrial (SHMT) (EC 2.1.2.1) (Glycine hydroxymethyltransferase) (Serine methylase) | SHMT2 | 6 | 34 | 0.8710 | 0.7540 | 0.5346 | 0.0656 | 0.8395 | 0.7009 | 0.8472 | 0.5787 | 0.7943 | 0.6056 | 0.5546 | 0.0585 | 0.8166 | 0.6623 | 1.2023 | 0.5476 | 0.9290 | 0.9040 | 1.2942 | 0.2791 | 0.9036 | 0.8152 | 1.0000 | 0.7972 | 0.6855 | 0.3574 | 0.7586 | 0.1080 |
| 217 | P34896 | Serine hydroxymethyltransferase, cytosolic (SHMT) (EC 2.1.2.1) (Glycine hydroxymethyltransferase) (Serine methylase) | SHMT1 | 3 | 49 | 0.7798 | 0.8689 | 0.6486 | 0.0818 | 0.4446 | 0.6726 | 0.4055 | 0.0004 | 0.8872 | 0.9552 | 0.2399 | 0.0000 | 0.5248 | 0.6420 | 1.3932 | 0.0567 | 1.1588 | 0.8411 | 1.0471 | 0.4050 | 1.1588 | 0.7940 | 0.8395 | 0.1718 | 2.1281 | 0.4221 | 0.2228 | 0.0000 |
| 218 | O43765 | Small glutamine-rich tetratricopeptide repeat-containing protein alpha (Alpha-SGT) (Vpu-binding protein) (UBP) | SGTA SGT SGT1 | 34 | 3 | 0.8551 | 0.5172 | 0.2228 | 0.5871 | 0.5649 | 0.1055 | 0.6427 | 0.9061 | 0.6252 | 0.0272 | 0.7943 | 0.7699 | 0.0982 | 0.0000 | 0.5058 | 0.4679 | 0.6982 | 0.2165 | 2.9376 | 0.6909 | 0.5105 | 0.0484 | 0.6026 | 0.9926 | 0.7178 | 0.0686 | 2.8576 | 0.7515 |
| 219 | O95470 | Sphingosine-1-phosphate lyase 1 (S1PL) (SP-lyase 1) (SPL 1) (hSPL) (EC 4.1.2.27) (Sphingosine-1-phosphate aldolase) | SGPL1 KIAA1252 | 43 | 3 | 0.5546 | 0.0002 | 1.4322 | 0.0810 | 1.4588 | 0.0352 | 1.1376 | 0.2430 | 0.9638 | 0.2630 | 1.9231 | 0.0388 | 0.3311 | 0.0000 | 0.7112 | 0.0323 | 0.4613 | 0.0000 | 1.1482 | 0.2119 | 0.0429 | 0.0000 | 1.6444 | 0.0552 | 0.3373 | 0.0000 | 1.4859 | 0.0736 |
| 220 | Q8TD22 | Sideroflexin-5 | SFXN5 | 4 | 4 | 0.4831 | 0.8180 | 0.4246 | 0.2668 | 1.2023 | 0.2656 | 0.5916 | 0.4067 | 1.2359 | 0.3846 | 0.4055 | 0.2441 | 0.8241 | 0.7687 | 0.3908 | 0.1866 | 0.3565 | 0.1742 | 0.6138 | 0.4426 | 1.4322 | 0.2395 | 0.4699 | 0.2594 | 1.5276 | 0.3793 | 1.0186 | 0.9786 |
| 221 | Q9H9B4 | Sideroflexin-1 (Tricarboxylate carrier protein) (TCC) | SFXN1 | 3 | 18 | 1.0375 | 0.8230 | 0.5297 | 0.1660 | 1.3062 | 0.2235 | 1.0000 | 0.8435 | 1.1912 | 0.7990 | 0.4875 | 0.0573 | 1.0864 | 0.7626 | 0.5649 | 0.0485 | 1.2823 | 0.7451 | 1.2942 | 0.7139 | 1.1588 | 0.4105 | 0.8710 | 0.2247 | 1.1912 | 0.5758 | 0.4365 | 0.0019 |
| 222 | P23246 | Splicing factor, proline- and glutamine-rich (100 kDa DNA-pairing protein) (hPOMp100) (DNA-binding p52/p100 complex, 100 kDa subunit) (Polypyrimidine tract-binding protein-associated-splicing factor) (PSF) (PTB-associated-splicing factor) | SFPQ PSF | 4 | 17 | 1.2589 | 0.9086 | 1.3552 | 0.4900 | 1.2474 | 0.7896 | 1.7701 | 0.4435 | 0.9727 | 0.6495 | 1.6596 | 0.2133 | 1.6749 | 0.4490 | 0.9462 | 0.7335 | 1.0000 | 0.5089 | 1.9055 | 0.0836 | 0.3698 | 0.1073 | 1.4859 | 0.3393 | 0.6252 | 0.3328 | 1.9953 | 0.1293 |
| 223 | Q15393 | Splicing factor 3B subunit 3 (Pre-mRNA-splicing factor SF3b 130 kDa subunit) (SF3b130) (STAF130) (Spliceosome-associated protein 130) (SAP 130) | SF3B3 KIAA0017 SAP130 | 21 | 4 | 1.2706 | 0.6591 | 0.8790 | 0.8867 | 1.3552 | 0.5334 | 1.1695 | 0.4883 | 1.0093 | 0.6566 | 1.1066 | 0.5619 | 1.5849 | 0.1793 | 1.2023 | 0.6197 | 1.0280 | 0.6390 | 0.6918 | 0.4688 | 0.2208 | 0.0105 | 0.7586 | 0.6149 | 0.5012 | 0.1160 | 0.8551 | 0.5256 |
| 224 | O75533 | Splicing factor 3B subunit 1 (Pre-mRNA-splicing factor SF3b 155 kDa subunit) (SF3b155) (Spliceosome-associated protein 155) (SAP 155) | SF3B1 SAP155 | 14 | 8 | 0.4246 | 0.2086 | 0.9817 | 0.9270 | 0.4246 | 0.2837 | 1.2474 | 0.9357 | 1.1169 | 0.8076 | 0.1770 | 0.0526 | 0.6607 | 0.6263 | 0.6026 | 0.3816 | 0.8630 | 0.2636 | 0.3565 | 0.3112 | 1.6293 | 0.4774 | 0.3908 | 0.2252 | 1.1376 | 0.9754 | 1.2942 | 0.8647 |
| 225 | Q01105 | Protein SET (HLA-DR-associated protein II) (Inhibitor of granzyme A-activated DNase) (IGAAD) (PHAPII) (Phosphatase 2A inhibitor I2PP2A) (I-2PP2A) (Template-activating factor I) (TAF-I) | SET | 5 | 5 | 1.2023 | 0.6616 | 1.2246 | 0.2835 | 0.8872 | 0.8940 | 1.0666 | 0.4319 | 0.8472 | 0.8115 | 1.6444 | 0.1419 | 0.8630 | 0.8495 | 0.7656 | 0.6622 | 0.6855 | 0.5450 | 1.3552 | 0.2082 | 1.3062 | 0.2024 | 1.1912 | 0.3190 | 1.0864 | 0.7463 | 1.5704 | 0.1793 |
| 226 | P50454 | Serpin H1 (47 kDa heat shock protein) (Arsenic-transactivated protein 3) (AsTP3) (Cell proliferation-inducing gene 14 protein) (Collagen-binding protein) (Colligin) (Rheumatoid arthritis-related antigen RA-A47) | SERPINH1 CBP1 CBP2 HSP47 SERPINH2 PIG14 | 4 | 15 | 0.6194 | 0.7790 | 1.3932 | 0.9076 | 0.8872 | 0.3003 | 1.0965 | 0.8259 | 0.4571 | 0.4371 | 1.2589 | 0.4524 | 0.8017 | 0.7957 | 0.7311 | 0.3447 | 1.9953 | 0.1817 | 1.5849 | 0.4547 | 1.5996 | 0.4840 | 0.6427 | 0.0730 | 0.5346 | 0.6661 | 2.8576 | 0.0226 |
| 227 | P05155 | Plasma protease C1 inhibitor (C1 Inh) (C1Inh) (C1 esterase inhibitor) (C1-inhibiting factor) (Serpin G1) | SERPING1 C1IN C1NH | 5 | 5 | 0.9290 | 0.6405 | 0.8091 | 0.9265 | 0.9638 | 0.8046 | 0.8017 | 0.6209 | 0.9638 | 0.8935 | 0.2051 | 0.1528 | 1.0093 | 0.8522 | 1.0186 | 0.8774 | 1.0666 | 0.5631 | 0.7311 | 0.7874 | 1.1272 | 0.6880 | 0.3192 | 0.0460 | 1.0000 | 0.9475 | 0.6918 | 0.1487 |
| 228 | P01008 | Antithrombin-III (ATIII) (Serpin C1) | SERPINC1 AT3 PRO0309 | 11 | 10 | 1.4191 | 0.3252 | 2.3988 | 0.1068 | 2.3988 | 0.2495 | 2.0512 | 0.1371 | 1.6144 | 0.3037 | 1.0864 | 0.9979 | 0.7870 | 0.8410 | 2.1086 | 0.0514 | 2.8840 | 0.0214 | 1.3677 | 0.7226 | 6.3680 | 0.0000 | 1.3183 | 0.6406 | 2.0893 | 0.0313 | 1.7378 | 0.4772 |
| 229 | P35237 | Serpin B6 (Cytoplasmic antiproteinase) (CAP) (Peptidase inhibitor 6) (PI-6) (Placental thrombin inhibitor) | SERPINB6 PI6 PTI | 4 | 10 | 0.7516 | 0.6594 | 2.6062 | 0.0015 | 1.2134 | 0.8480 | 2.6546 | 0.0023 | 0.9376 | 0.6473 | 2.4889 | 0.0418 | 1.1588 | 0.8188 | 2.2699 | 0.0132 | 0.7516 | 0.5093 | 2.4434 | 0.0156 | 0.9638 | 0.9046 | 2.1478 | 0.0045 | 1.3062 | 0.4030 | 2.5586 | 0.0076 |
| 230 | P30740 | Leukocyte elastase inhibitor (LEI) (Monocyte/neutrophil elastase inhibitor) (EI) (M/NEI) (Peptidase inhibitor 2) (PI-2) (Serpin B1) | SERPINB1 ELANH2 MNEI PI2 | 11 | 7 | 1.1482 | 0.8902 | 0.2831 | 0.1272 | 2.1086 | 0.0155 | 0.2582 | 0.0280 | 2.0324 | 0.0917 | 0.9727 | 0.8742 | 1.6144 | 0.1611 | 0.9727 | 0.5484 | 0.6855 | 0.7555 | 1.3677 | 0.2866 | 0.3981 | 0.1337 | 0.8318 | 0.7008 | 0.7112 | 0.6562 | 1.4997 | 0.1696 |
| 231 | P05154 | Plasma serine protease inhibitor (Acrosomal serine protease inhibitor) (Plasminogen activator inhibitor 3) (PAI-3) (PAI3) (Protein C inhibitor) (PCI) (Serpin A5) | SERPINA5 PCI PLANH3 PROCI | 8 | 5 | 5.3951 | 0.3974 | 0.9120 | 0.8099 | 11.3763 | 0.0021 | 1.8880 | 0.1636 | 11.0662 | 0.0016 | 1.2823 | 0.6189 | 7.1779 | 0.0220 | 1.2942 | 0.8836 | 4.6132 | 0.4417 | 1.4322 | 0.7563 | 12.3595 | 0.0024 | 0.9204 | 0.8390 | 12.3595 | 0.0041 | 2.0324 | 0.3820 |
| 232 | P01011 | Alpha-1-antichymotrypsin (ACT) (Cell growth-inhibiting gene 24/25 protein) (Serpin A3) [Cleaved into: Alpha-1-antichymotrypsin His-Pro-less] | SERPINA3 AACT GIG24 GIG25 | 9 | 12 | 2.1086 | 0.0928 | 1.1482 | 0.2819 | 0.8551 | 0.6804 | 1.0280 | 0.2411 | 1.8365 | 0.1320 | 1.5849 | 0.1320 | 1.5560 | 0.1825 | 0.8472 | 0.7102 | 1.4454 | 0.2399 | 1.3428 | 0.3490 | 2.1086 | 0.1036 | 1.0666 | 0.6160 | 2.5351 | 0.0151 | 0.4487 | 0.1738 |
| 233 | P01009 | Alpha-1-antitrypsin (Alpha-1 protease inhibitor) (Alpha-1-antiproteinase) (Serpin A1) [Cleaved into: Short peptide from AAT (SPAAT)] | SERPINA1 AAT PI PRO0684 PRO2209 | 4 | 39 | 0.8472 | 0.9568 | 0.4169 | 0.0019 | 1.3305 | 0.7700 | 0.4365 | 0.0011 | 1.5996 | 0.6209 | 0.8790 | 0.2595 | 0.2655 | 0.3532 | 0.9120 | 0.3031 | 1.5704 | 0.6014 | 1.1066 | 0.6919 | 1.2134 | 0.8958 | 0.9290 | 0.2349 | 1.8880 | 0.4961 | 1.1272 | 0.2459 |
| 234 | Q8NC51 | Plasminogen activator inhibitor 1 RNA-binding protein (PAI1 RNA-binding protein 1) (PAI-RBP1) (SERPINE1 mRNA-binding protein 1) | SERBP1 PAIRBP1 CGI-55 | 13 | 10 | 1.7378 | 0.4058 | 0.5105 | 0.3309 | 1.6144 | 0.3777 | 1.0568 | 0.9223 | 1.9231 | 0.0812 | 1.0471 | 0.9072 | 1.9770 | 0.1216 | 1.0965 | 0.9998 | 0.8551 | 0.6289 | 1.2706 | 0.6593 | 1.4454 | 0.1933 | 0.9550 | 0.9752 | 1.1482 | 0.3580 | 0.8472 | 0.9378 |
| 235 | Q9UHD8 | Septin-9 (MLL septin-like fusion protein MSF-A) (MLL septin-like fusion protein) (Ovarian/Breast septin) (Ov/Br septin) (Septin D1) | SEPT9 KIAA0991 MSF | 46 | 9 | 0.3404 | 0.0000 | 0.7178 | 0.8737 | 1.3804 | 0.2151 | 0.6194 | 0.4013 | 1.5996 | 0.0762 | 0.8790 | 0.9196 | 1.5560 | 0.2852 | 0.5808 | 0.3885 | 0.3698 | 0.0002 | 1.2589 | 0.4046 | 1.3062 | 0.4956 | 1.1695 | 0.4540 | 1.2246 | 0.8583 | 1.3552 | 0.1286 |
| 236 | Q16181 | Septin-7 (CDC10 protein homolog) | SEPT7 CDC10 | 7 | 9 | 0.5248 | 0.8961 | 1.1695 | 0.7936 | 1.1376 | 0.6174 | 1.3552 | 0.6743 | 2.1478 | 0.2662 | 1.4997 | 0.3056 | 1.9588 | 0.3976 | 1.3804 | 0.8216 | 1.3062 | 0.9039 | 1.2246 | 0.9719 | 2.6792 | 0.1846 | 1.1695 | 0.8222 | 1.6444 | 0.3790 | 1.4322 | 0.5461 |
| 237 | Q15019 | Septin-2 (Neural precursor cell expressed developmentally down-regulated protein 5) (NEDD-5) | SEPT2 DIFF6 KIAA0158 NEDD5 | 8 | 12 | 0.9727 | 0.9994 | 1.1588 | 0.5444 | 1.1169 | 0.8439 | 1.2589 | 0.1820 | 1.0965 | 0.4893 | 1.1272 | 0.4517 | 0.7516 | 0.5913 | 1.1482 | 0.3402 | 0.8241 | 0.6629 | 1.2359 | 0.2091 | 1.4060 | 0.1108 | 1.1376 | 0.5425 | 1.3305 | 0.3711 | 1.3428 | 0.0346 |
| 238 | Q9NVA2 | Septin-11 | 11-Sep | 8 | 4 | 1.1803 | 0.3617 | 0.4966 | 0.3827 | 2.6792 | 0.0372 | 1.0471 | 0.9366 | 0.8472 | 0.8969 | 1.1803 | 0.9468 | 1.2589 | 0.3423 | 0.5861 | 0.4570 | 2.6303 | 0.0543 | 0.8166 | 0.6787 | 2.9648 | 0.0142 | 0.7727 | 0.7320 | 2.9376 | 0.0696 | 1.3183 | 0.7094 |
| 239 | Q99611 | Selenide, water dikinase 2 (EC 2.7.9.3) (Selenium donor protein 2) (Selenophosphate synthase 2) | SEPHS2 SPS2 | 13 | 7 | 0.6252 | 0.3066 | 0.6310 | 0.8891 | 1.6293 | 0.1186 | 1.4723 | 0.2086 | 0.6607 | 0.7049 | 0.6855 | 0.8180 | 0.8790 | 0.7039 | 1.0864 | 0.9463 | 1.4191 | 0.2612 | 1.0000 | 0.8280 | 1.2706 | 0.1312 | 1.3552 | 0.2913 | 1.0093 | 0.7479 | 0.6138 | 0.0966 |
| 240 | Q9BVL4 | Selenoprotein O (SelO) | SELO | 5 | 4 | 0.3373 | 0.1572 | 0.6918 | 0.2260 | 0.9638 | 0.6217 | 1.2134 | 0.9737 | 0.6607 | 0.2945 | 1.1169 | 0.5566 | 0.4446 | 0.4004 | 1.2942 | 0.1629 | 1.0765 | 0.9813 | 0.7379 | 0.1097 | 0.6982 | 0.5512 | 1.4322 | 0.1680 | 0.9290 | 0.4984 | 1.2134 | 0.6918 |
| 241 | Q13228 | Selenium-binding protein 1 (56 kDa selenium-binding protein) (SBP56) (SP56) | SELENBP1 SBP | 4 | 51 | 1.2474 | 0.7324 | 1.3932 | 0.0383 | 1.2589 | 0.6427 | 1.0186 | 0.7579 | 1.1803 | 0.9148 | 0.8551 | 0.2822 | 1.6596 | 0.2192 | 0.8551 | 0.4322 | 1.0765 | 0.9799 | 1.0375 | 0.9758 | 0.6368 | 0.4817 | 0.9462 | 0.5276 | 0.8872 | 0.4704 | 0.4786 | 0.0004 |
| 242 | P61619 | Protein transport protein Sec61 subunit alpha isoform 1 (Sec61 alpha-1) | SEC61A1 SEC61A | 5 | 7 | 6.3680 | 0.0451 | 2.6792 | 0.0999 | 5.7544 | 0.2288 | 1.4191 | 0.5552 | 2.9923 | 0.3968 | 1.1066 | 0.5848 | 2.8840 | 0.2148 | 2.1478 | 0.2309 | 6.9183 | 0.0682 | 2.5351 | 0.1232 | 2.0324 | 0.7037 | 0.4055 | 0.4104 | 2.6303 | 0.3200 | 1.5417 | 0.5092 |
| 243 | O94979 | Protein transport protein Sec31A (ABP125) (ABP130) (SEC31-like protein 1) (SEC31-related protein A) (Web1-like protein) | SEC31A KIAA0905 SEC31L1 HSPC275 HSPC334 | 56 | 15 | 1.1066 | 0.7285 | 1.0471 | 0.4452 | 0.9462 | 0.1801 | 0.9120 | 0.8890 | 1.3552 | 0.0358 | 1.5996 | 0.6603 | 0.9817 | 0.1490 | 1.1912 | 0.6024 | 0.9462 | 0.3092 | 2.1478 | 0.1696 | 0.1432 | 0.0000 | 1.4454 | 0.3269 | 0.4055 | 0.0000 | 1.7701 | 0.1876 |
| 244 | P53992 | Protein transport protein Sec24C (SEC24-related protein C) | SEC24C KIAA0079 | 4 | 5 | 4.5290 | 0.1000 | 0.5970 | 0.3159 | 1.7865 | 0.1002 | 0.6668 | 0.3239 | 1.1588 | 0.5908 | 1.0666 | 0.5406 | 2.4660 | 0.0794 | 1.7219 | 0.6162 | 3.4674 | 0.1266 | 0.6081 | 0.4870 | 3.8019 | 0.0337 | 1.7865 | 0.5466 | 2.2909 | 0.2676 | 1.5136 | 0.3119 |
| 245 | O95487 | Protein transport protein Sec24B (SEC24-related protein B) | SEC24B | 15 | 5 | 1.2474 | 0.6076 | 0.9727 | 0.6967 | 0.9817 | 0.8956 | 0.9908 | 0.7795 | 1.4723 | 0.2177 | 0.6252 | 0.1538 | 1.3183 | 0.5878 | 0.6918 | 0.3747 | 1.4454 | 0.4704 | 0.7311 | 0.3736 | 1.9055 | 0.1580 | 0.8872 | 0.4296 | 1.6444 | 0.4671 | 1.0280 | 0.8072 |
| 246 | O95486 | Protein transport protein Sec24A (SEC24-related protein A) | SEC24A | 9 | 6 | 0.5702 | 0.3235 | 0.3597 | 0.4145 | 0.8017 | 0.7308 | 0.8017 | 0.8788 | 0.7047 | 0.3013 | 0.7870 | 0.8884 | 0.6855 | 0.9980 | 0.6427 | 0.8533 | 0.9376 | 0.9911 | 2.4889 | 0.2222 | 0.9204 | 0.6659 | 1.1588 | 0.6735 | 1.1376 | 0.4326 | 2.1878 | 0.4423 |
| 247 | Q9Y6Y8 | SEC23-interacting protein (p125) | SEC23IP MSTP053 | 4 | 3 | 0.9462 | 0.7528 | 0.7112 | 0.1489 | 0.9376 | 0.7540 | 0.7244 | 0.3394 | 0.9290 | 0.7520 | 0.7586 | 0.2312 | 0.9462 | 0.7662 | 0.7798 | 0.1407 | 1.0280 | 0.8418 | 0.8166 | 0.2735 | 0.9462 | 0.8536 | 0.8241 | 0.3197 | 1.0568 | 0.8973 | 0.9120 | 0.5692 |
| 248 | Q15436 | Protein transport protein Sec23A (SEC23-related protein A) | SEC23A | 6 | 16 | 0.9290 | 0.9524 | 1.0765 | 0.1509 | 1.0568 | 0.8823 | 1.0093 | 0.3897 | 0.9908 | 0.9346 | 1.1482 | 0.1453 | 1.0568 | 0.8140 | 1.0666 | 0.4332 | 1.1376 | 0.6596 | 1.1272 | 0.5495 | 1.2474 | 0.4927 | 1.1588 | 0.3525 | 0.8790 | 0.8048 | 1.1695 | 0.0256 |
| 249 | O75396 | Vesicle-trafficking protein SEC22b (ER-Golgi SNARE of 24 kDa) (ERS-24) (ERS24) (SEC22 vesicle-trafficking protein homolog B) (SEC22 vesicle-trafficking protein-like 1) | SEC22B SEC22L1 | 3 | 10 | 1.4322 | 0.3127 | 1.3677 | 0.0067 | 1.2589 | 0.4231 | 1.3305 | 0.0293 | 1.2134 | 0.4567 | 1.1588 | 0.0924 | 0.9204 | 0.8733 | 1.2134 | 0.0535 | 1.1482 | 0.5468 | 1.2474 | 0.0217 | 1.3552 | 0.3681 | 1.3062 | 0.0235 | 1.3305 | 0.3650 | 1.2823 | 0.0093 |
| 250 | O76054 | SEC14-like protein 2 (Alpha-tocopherol-associated protein) (TAP) (hTAP) (Squalene transfer protein) (Supernatant protein factor) (SPF) | SEC14L2 C22orf6 KIAA1186 KIAA1658 | 17 | 28 | 1.1803 | 0.6774 | 1.2246 | 0.1668 | 2.2080 | 0.0993 | 1.2823 | 0.1275 | 2.6546 | 0.1307 | 0.5649 | 0.0903 | 1.3305 | 0.9968 | 0.8630 | 0.9895 | 1.3932 | 0.7281 | 1.2589 | 0.3252 | 2.4889 | 0.0761 | 0.9290 | 0.6900 | 2.5586 | 0.1188 | 0.5395 | 0.0739 |
| 251 | P55735 | Protein SEC13 homolog (SEC13-like protein 1) (SEC13-related protein) | SEC13 D3S1231E SEC13L1 SEC13R | 12 | 4 | 0.9376 | 0.7069 | 0.7178 | 0.2544 | 1.1482 | 0.2805 | 0.9462 | 0.6766 | 1.2359 | 0.3501 | 0.9462 | 0.6077 | 1.1169 | 0.3830 | 0.7516 | 0.7712 | 0.7870 | 0.9611 | 1.0568 | 0.3811 | 1.6144 | 0.1712 | 0.9817 | 0.5933 | 2.3335 | 0.1007 | 1.1482 | 0.3070 |
| 252 | Q96GA7 | Serine dehydratase-like (L-serine deaminase) (L-serine dehydratase/L-threonine deaminase) (L-threonine dehydratase) (TDH) (EC 4.3.1.19) (Serine dehydratase 2) (SDH 2) (EC 4.3.1.17) | SDSL | 30 | 7 | 1.2823 | 0.1022 | 2.5119 | 0.3106 | 0.8710 | 0.3721 | 3.6308 | 0.1746 | 1.2359 | 0.2384 | 2.2699 | 0.1824 | 0.5105 | 0.1256 | 3.0200 | 0.1476 | 1.1803 | 0.3778 | 2.6792 | 0.1539 | 0.1159 | 0.0001 | 1.0965 | 0.9255 | 0.2729 | 0.0000 | 4.0551 | 0.0192 |
| 253 | P20132 | L-serine dehydratase/L-threonine deaminase (SDH) (EC 4.3.1.17) (L-serine deaminase) (L-threonine dehydratase) (TDH) (EC 4.3.1.19) | SDS SDH | 4 | 3 | 0.8872 | 0.7435 | 0.1977 | 0.0063 | 1.0375 | 0.8487 | 2.3550 | 0.0246 | 0.8630 | 0.6740 | 0.1393 | 0.0336 | 0.8790 | 0.7199 | 0.6368 | 0.1884 | 0.6792 | 0.3043 | 0.8790 | 0.3927 | 0.7798 | 0.4298 | 0.2754 | 0.0287 | 0.8551 | 0.6352 | 0.3597 | 0.2194 |
| 254 | Q9NRG7 | Epimerase family protein SDR39U1 (EC 1.1.1.-) (Short-chain dehydrogenase/reductase family 39U member 1) | SDR39U1 C14orf124 HCDI | 9 | 3 | 5.9156 | 0.1054 | 1.0280 | 0.2264 | 7.5858 | 0.0466 | 5.5976 | 0.4267 | 4.4875 | 0.1432 | 4.2073 | 0.7482 | 9.0365 | 0.0024 | 2.3988 | 0.6240 | 2.4889 | 0.2244 | 0.8790 | 0.1988 | 5.7544 | 0.0067 | 0.5297 | 0.3026 | 2.7290 | 0.5318 | 0.0817 | 0.0760 |
| 255 | P21912 | Succinate dehydrogenase [ubiquinone] iron-sulfur subunit, mitochondrial (EC 1.3.5.1) (Iron-sulfur subunit of complex II) (Ip) | SDHB SDH SDH1 | 7 | 11 | 0.9204 | 0.5444 | 0.7727 | 0.8088 | 0.5495 | 0.1974 | 0.4875 | 0.1397 | 1.5417 | 0.3907 | 0.7656 | 0.7650 | 1.0093 | 0.8715 | 0.7047 | 0.4601 | 0.1406 | 0.0699 | 0.7516 | 0.2680 | 0.6918 | 0.6071 | 1.1482 | 0.5072 | 0.1644 | 0.1172 | 0.6668 | 0.3265 |
| 256 | P31040 | Succinate dehydrogenase [ubiquinone] flavoprotein subunit, mitochondrial (EC 1.3.5.1) (Flavoprotein subunit of complex II) (Fp) | SDHA SDH2 SDHF | 3 | 28 | 6.3096 | 0.2774 | 1.2589 | 0.9491 | 2.3768 | 0.2754 | 1.0186 | 0.9886 | 1.4723 | 0.8445 | 1.7701 | 0.7277 | 6.2517 | 0.2773 | 0.6792 | 0.7482 | 3.9811 | 0.3694 | 1.3305 | 0.8032 | 3.4674 | 0.6049 | 2.8314 | 0.4458 | 0.5916 | 0.9996 | 1.1803 | 0.7263 |
| 257 | Q96FV2 | Secernin-2 | SCRN2 | 11 | 24 | 1.0375 | 0.7581 | 0.6138 | 0.4687 | 0.8630 | 0.9984 | 0.6138 | 0.5005 | 0.6310 | 0.1003 | 0.7656 | 0.8400 | 1.2589 | 0.2699 | 0.6252 | 0.5157 | 0.8551 | 0.2866 | 0.7311 | 0.8167 | 0.4285 | 0.0150 | 0.7870 | 0.5564 | 0.6194 | 0.2316 | 0.8551 | 0.8779 |
| 258 | O14595 | Carboxy-terminal domain RNA polymerase II polypeptide A small phosphatase 2 (EC 3.1.3.16) (Nuclear LIM interactor-interacting factor 2) (NLI-interacting factor 2) (Protein OS-4) (Small C-terminal domain phosphatase 2) (Small CTD phosphatase 2) (SCP2) | CTDSP2 NIF2 OS4 SCP2 | 32 | 7 | 1.6749 | 0.4443 | 3.0200 | 0.0433 | 1.5996 | 0.4194 | 1.1588 | 0.6808 | 1.8365 | 0.3565 | 1.1376 | 0.6004 | 1.1588 | 0.6017 | 1.2359 | 0.2439 | 1.6444 | 0.3832 | 1.3932 | 0.6474 | 0.5297 | 0.4237 | 1.5136 | 0.4485 | 1.0280 | 0.9723 | 0.4875 | 0.7196 |
| 259 | P22307 | Non-specific lipid-transfer protein (NSL-TP) (EC 2.3.1.176) (Propanoyl-CoA C-acyltransferase) (SCP-chi) (SCPX) (Sterol carrier protein 2) (SCP-2) (Sterol carrier protein X) (SCP-X) | SCP2 | 30 | 56 | 0.6138 | 0.4381 | 1.6144 | 0.1052 | 0.7516 | 0.6448 | 0.4130 | 0.0915 | 0.7586 | 0.5596 | 3.3729 | 0.0000 | 0.7870 | 0.5635 | 2.5119 | 0.0022 | 0.5754 | 0.3823 | 2.2491 | 0.0015 | 0.5970 | 0.3970 | 1.3552 | 0.4322 | 0.5445 | 0.3526 | 1.6144 | 0.0549 |
| 260 | O75880 | Protein SCO1 homolog, mitochondrial | SCO1 SCOD1 | 5 | 4 | 1.0568 | 0.5794 | 1.4997 | 0.4381 | 1.0666 | 0.5311 | 1.4723 | 0.4709 | 1.0186 | 0.6606 | 1.6596 | 0.6463 | 0.8472 | 0.2227 | 2.7290 | 0.3513 | 1.0965 | 0.7810 | 1.9770 | 0.4438 | 0.7379 | 0.2050 | 3.4356 | 0.3177 | 1.0093 | 0.9310 | 2.2284 | 0.5588 |
| 261 | Q96I15 | Selenocysteine lyase (hSCL) (EC 4.4.1.16) | SCLY SCL | 62 | 7 | 1.1588 | 0.4724 | 0.8872 | 0.8640 | 1.3305 | 0.7709 | 0.4365 | 0.5018 | 1.9231 | 0.0312 | 1.7539 | 0.0619 | 1.2474 | 0.8731 | 0.7047 | 0.7526 | 1.7539 | 0.2450 | 0.8630 | 0.6606 | 0.1368 | 0.0000 | 0.9727 | 0.6338 | 0.6252 | 0.0354 | 0.4742 | 0.1447 |
| 262 | Q8NBX0 | Saccharopine dehydrogenase-like oxidoreductase (EC 1.-.-.-) | SCCPDH CGI-49 | 3 | 17 | 3.0200 | 0.2755 | 1.5417 | 0.2052 | 3.6644 | 0.1164 | 0.7311 | 0.2525 | 1.7378 | 0.2174 | 0.6138 | 0.5212 | 3.5975 | 0.1181 | 0.9376 | 0.8303 | 2.7797 | 0.1883 | 0.8241 | 0.3834 | 0.5916 | 0.0228 | 1.2589 | 0.3036 | 0.2399 | 0.0705 | 0.4613 | 0.0200 |
| 263 | Q14108 | Lysosome membrane protein 2 (85 kDa lysosomal membrane sialoglycoprotein) (LGP85) (CD36 antigen-like 2) (Lysosome membrane protein II) (LIMP II) (Scavenger receptor class B member 2) (CD antigen CD36) | SCARB2 CD36L2 LIMP2 LIMPII | 7 | 10 | 1.3062 | 0.5620 | 0.9120 | 0.4537 | 1.3932 | 0.4624 | 0.5248 | 0.2253 | 1.4454 | 0.1156 | 0.6138 | 0.3422 | 0.9908 | 0.8693 | 0.5395 | 0.3557 | 1.2589 | 0.4308 | 0.9036 | 0.5074 | 0.2655 | 0.0840 | 1.4723 | 0.6163 | 0.2965 | 0.2017 | 1.4191 | 0.5135 |
| 264 | Q96F10 | Diamine acetyltransferase 2 (EC 2.3.1.57) (Polyamine N-acetyltransferase 2) (Spermidine/spermine N(1)-acetyltransferase 2) (Thialysine N-epsilon-acetyltransferase) | SAT2 SSAT2 | 14 | 4 | 1.6749 | 0.2811 | 1.3804 | 0.7052 | 2.9923 | 0.0853 | 1.2023 | 0.7708 | 2.0137 | 0.3422 | 0.7379 | 0.6262 | 2.3768 | 0.1921 | 0.8472 | 0.4353 | 2.3988 | 0.1665 | 1.4322 | 0.5871 | 0.2168 | 0.0238 | 0.5649 | 0.5136 | 0.9550 | 0.3551 | 0.7112 | 0.5416 |
| 265 | Q9NP81 | Serine--tRNA ligase, mitochondrial (EC 6.1.1.11) (SerRSmt) (Seryl-tRNA synthetase) (SerRS) (Seryl-tRNA(Ser/Sec) synthetase) | SARS2 SARSM | 7 | 3 | 0.5012 | 0.3698 | 0.9638 | 0.8506 | 0.6310 | 0.2736 | 1.1482 | 0.5874 | 0.8710 | 0.9249 | 1.1803 | 0.4822 | 0.8872 | 0.8596 | 1.1376 | 0.5250 | 0.2249 | 0.0472 | 1.0864 | 0.6969 | 0.9204 | 0.8547 | 1.0093 | 0.8726 | 1.1695 | 0.2940 | 0.9908 | 0.9041 |
| 266 | P49591 | Serine--tRNA ligase, cytoplasmic (EC 6.1.1.11) (Seryl-tRNA synthetase) (SerRS) (Seryl-tRNA(Ser/Sec) synthetase) | SARS SERS | 3 | 6 | 0.4831 | 0.2975 | 0.8166 | 0.5140 | 0.8872 | 0.8175 | 0.7311 | 0.3110 | 1.1272 | 0.7803 | 1.2474 | 0.5579 | 0.9290 | 0.8831 | 0.9908 | 0.9311 | 1.1803 | 0.7179 | 1.2589 | 0.6183 | 0.2965 | 0.1807 | 1.0280 | 0.9272 | 0.4093 | 0.2421 | 1.3183 | 0.5479 |
| 267 | Q9UL12 | Sarcosine dehydrogenase, mitochondrial (SarDH) (EC 1.5.8.3) (BPR-2) | SARDH DMGDHL1 | 3 | 30 | 1.2023 | 0.6402 | 0.6668 | 0.0829 | 0.9204 | 0.8210 | 1.6904 | 0.0033 | 1.1912 | 0.6873 | 0.5495 | 0.0023 | 0.8954 | 0.9594 | 0.8395 | 0.5279 | 1.0765 | 0.7191 | 0.6368 | 0.0380 | 1.0965 | 0.8996 | 0.5808 | 0.0057 | 1.3428 | 0.5794 | 0.0964 | 0.0000 |
| 268 | Q9Y6B6 | GTP-binding protein SAR1b (GTP-binding protein B) (GTBPB) | SAR1B SARA2 SARB | 7 | 5 | 0.7656 | 0.8545 | 0.9462 | 0.5408 | 0.9120 | 0.8039 | 0.5861 | 0.9812 | 0.6668 | 0.4732 | 1.0568 | 0.8199 | 1.4191 | 0.1732 | 0.6138 | 0.8612 | 1.0280 | 0.6742 | 1.1588 | 0.5562 | 0.8318 | 0.7139 | 1.1169 | 0.5087 | 0.7047 | 0.4757 | 0.6368 | 0.8061 |
| 269 | Q9NR31 | GTP-binding protein SAR1a (COPII-associated small GTPase) | SAR1A SAR1 SARA SARA1 | 25 | 11 | 0.6607 | 0.0029 | 0.9727 | 0.8277 | 1.2023 | 0.9612 | 0.5808 | 0.1509 | 0.6368 | 0.0103 | 0.6918 | 0.1985 | 1.0375 | 0.8508 | 1.0864 | 0.7672 | 1.1169 | 0.9753 | 0.6310 | 0.2696 | 0.0912 | 0.0000 | 0.8091 | 0.2341 | 0.2032 | 0.0000 | 0.6486 | 0.4080 |
| 270 | Q9Y3Z3 | Deoxynucleoside triphosphate triphosphohydrolase SAMHD1 (dNTPase) (EC 3.1.5.-) (Dendritic cell-derived IFNG-induced protein) (DCIP) (Monocyte protein 5) (MOP-5) (SAM domain and HD domain-containing protein 1) | SAMHD1 MOP5 | 11 | 10 | 0.7379 | 0.8190 | 0.8241 | 0.5381 | 0.8395 | 0.9940 | 0.4920 | 0.0034 | 1.0093 | 0.6426 | 1.4454 | 0.1489 | 0.4613 | 0.4096 | 0.4656 | 0.2287 | 0.7112 | 0.8505 | 1.2134 | 0.3472 | 0.4207 | 0.1936 | 0.7178 | 0.1270 | 0.4966 | 0.5116 | 1.9055 | 0.0089 |
| 271 | Q9NTJ5 | Phosphatidylinositide phosphatase SAC1 (EC 3.1.3.-) (Suppressor of actin mutations 1-like protein) | SACM1L KIAA0851 | 7 | 4 | 1.0471 | 0.8136 | 1.8197 | 0.2805 | 0.9550 | 0.8543 | 1.4454 | 0.4737 | 1.0375 | 0.4122 | 1.8030 | 0.2889 | 1.0471 | 0.5709 | 1.5849 | 0.5172 | 0.9908 | 0.9639 | 1.8880 | 0.0794 | 1.0568 | 0.3903 | 1.4723 | 0.5790 | 1.0765 | 0.4278 | 0.8091 | 0.6893 |
| 272 | P06703 | Protein S100-A6 (Calcyclin) (Growth factor-inducible protein 2A9) (MLN 4) (Prolactin receptor-associated protein) (PRA) (S100 calcium-binding protein A6) | S100A6 CACY | 12 | 4 | 0.7586 | 0.7991 | 1.0000 | 0.5564 | 1.0280 | 0.4798 | 1.5996 | 0.5861 | 0.8551 | 0.5871 | 0.4831 | 0.2185 | 0.7379 | 0.6850 | 0.9727 | 0.6940 | 0.7798 | 0.4203 | 0.8551 | 0.6665 | 3.1333 | 0.0023 | 0.6486 | 0.4740 | 1.9770 | 0.0497 | 1.2359 | 0.7820 |
| 273 | Q99584 | Protein S100-A13 (S100 calcium-binding protein A13) | S100A13 | 5 | 4 | 1.2359 | 0.4023 | 1.1376 | 0.7357 | 0.9204 | 0.9921 | 1.3183 | 0.1226 | 0.9462 | 0.8925 | 0.7379 | 0.3997 | 1.0666 | 0.6574 | 0.4325 | 0.2318 | 1.0965 | 0.6463 | 0.8630 | 0.5394 | 0.9290 | 0.9817 | 0.6855 | 0.3005 | 1.1272 | 0.3049 | 1.1066 | 0.6391 |
| 274 | P31949 | Protein S100-A11 (Calgizzarin) (Metastatic lymph node gene 70 protein) (MLN 70) (Protein S100-C) (S100 calcium-binding protein A11) [Cleaved into: Protein S100-A11, N-terminally processed] | S100A11 MLN70 S100C | 6 | 5 | 0.3105 | 0.4561 | 0.5808 | 0.2455 | 1.3552 | 0.7301 | 0.9638 | 0.6605 | 0.7447 | 0.7479 | 1.4997 | 0.2293 | 0.6194 | 0.6282 | 0.4325 | 0.0546 | 1.1482 | 0.9700 | 1.3932 | 0.3969 | 0.8954 | 0.9018 | 0.5346 | 0.1387 | 0.9727 | 0.9395 | 1.5996 | 0.1349 |
| 275 | P60903 | Protein S100-A10 (Calpactin I light chain) (Calpactin-1 light chain) (Cellular ligand of annexin II) (S100 calcium-binding protein A10) (p10 protein) (p11) | S100A10 ANX2LG CAL1L CLP11 | 3 | 3 | 0.1995 | 0.0778 | 0.4699 | 0.1930 | 0.8630 | 0.5626 | 0.5916 | 0.2004 | 0.5598 | 0.1320 | 0.6546 | 0.1776 | 0.4613 | 0.0856 | 0.4446 | 0.2070 | 1.2134 | 0.4037 | 0.4169 | 0.1343 | 1.0093 | 0.8953 | 0.3467 | 0.2049 | 0.4446 | 0.0880 | 1.1482 | 0.6928 |
| 276 | Q9Y230 | RuvB-like 2 (EC 3.6.4.12) (48 kDa TATA box-binding protein-interacting protein) (48 kDa TBP-interacting protein) (51 kDa erythrocyte cytosolic protein) (ECP-51) (INO80 complex subunit J) (Repressing pontin 52) (Reptin 52) (TIP49b) (TIP60-associated protein 54-beta) (TAP54-beta) | RUVBL2 INO80J TIP48 TIP49B CGI-46 | 5 | 4 | 0.3945 | 0.1412 | 0.9290 | 0.6025 | 1.1272 | 0.6095 | 0.8954 | 0.6713 | 0.3664 | 0.0786 | 1.0280 | 0.7477 | 0.9727 | 0.5457 | 1.0666 | 0.6572 | 0.7178 | 0.5609 | 0.9817 | 0.9004 | 3.2211 | 0.0200 | 1.0471 | 0.7567 | 1.1912 | 0.5171 | 0.9290 | 0.8118 |
| 277 | Q9NQC3 | Reticulon-4 (Foocen) (Neurite outgrowth inhibitor) (Nogo protein) (Neuroendocrine-specific protein) (NSP) (Neuroendocrine-specific protein C homolog) (RTN-x) (Reticulon-5) | RTN4 KIAA0886 NOGO My043 SP1507 | 4 | 6 | 0.1837 | 0.2139 | 0.9817 | 0.9982 | 0.8091 | 0.8028 | 0.6368 | 0.6845 | 0.3945 | 0.3389 | 0.5754 | 0.3950 | 0.6668 | 0.5530 | 0.9036 | 0.9653 | 0.9120 | 0.9863 | 0.6427 | 0.4527 | 1.2359 | 0.6042 | 0.4169 | 0.5545 | 0.3733 | 0.4227 | 0.9290 | 0.8201 |
| 278 | Q15404 | Ras suppressor protein 1 (RSP-1) (Rsu-1) | RSU1 RSP1 | 3 | 5 | 1.3552 | 0.3645 | 2.1281 | 0.3561 | 0.6252 | 0.3431 | 1.0375 | 0.9287 | 1.6444 | 0.1782 | 1.0666 | 0.6947 | 1.0280 | 0.8747 | 1.1588 | 0.9829 | 1.1588 | 0.4238 | 0.6368 | 0.4873 | 1.3804 | 0.4042 | 1.6596 | 0.7212 | 1.6596 | 0.2075 | 1.0965 | 0.4041 |
| 279 | Q9P2E9 | Ribosome-binding protein 1 (180 kDa ribosome receptor homolog) (RRp) (ES/130-related protein) (Ribosome receptor protein) | RRBP1 KIAA1398 | 5 | 83 | 0.6918 | 0.0516 | 0.7311 | 0.1564 | 0.8472 | 0.3414 | 0.7447 | 0.1360 | 0.4875 | 0.0778 | 1.7061 | 0.0096 | 0.9817 | 0.1899 | 1.0765 | 0.5064 | 0.8551 | 0.3172 | 1.1803 | 0.7975 | 0.6138 | 0.4380 | 1.6444 | 0.0127 | 0.8017 | 0.1837 | 1.5996 | 0.0013 |
| 280 | P08865 | 40S ribosomal protein SA (37 kDa laminin receptor precursor) (37LRP) (37/67 kDa laminin receptor) (LRP/LR) (67 kDa laminin receptor) (67LR) (Colon carcinoma laminin-binding protein) (Laminin receptor 1) (LamR) (Laminin-binding protein precursor p40) (LBP/p40) (Multidrug resistance-associated protein MGr1-Ag) (NEM/1CHD4) | RPSA LAMBR LAMR1 | 3 | 18 | 1.1695 | 0.0031 | 3.1046 | 0.0698 | 1.2474 | 0.0032 | 2.4210 | 0.0964 | 1.0375 | 0.0037 | 2.6792 | 0.0118 | 1.0471 | 0.0040 | 2.3988 | 0.0861 | 1.2359 | 0.0032 | 2.9923 | 0.0670 | 1.2023 | 0.0030 | 2.4889 | 0.1404 | 1.0965 | 0.0031 | 2.7542 | 0.2194 |
| 281 | P46781 | 40S ribosomal protein S9 | RPS9 | 104 | 10 | 1.2359 | 0.9715 | 0.9290 | 0.9546 | 0.9908 | 0.7626 | 0.8872 | 0.7381 | 1.2823 | 0.1499 | 0.9727 | 0.9410 | 1.9953 | 0.0031 | 0.7798 | 0.8144 | 1.5417 | 0.2298 | 1.0666 | 0.3131 | 2.2491 | 0.0001 | 0.6982 | 0.8620 | 0.9462 | 0.4286 | 1.4997 | 0.0586 |
| 282 | P62241 | 40S ribosomal protein S8 | RPS8 OK/SW-cl.83 | 25 | 17 | 2.8314 | 0.5025 | 1.3305 | 0.9733 | 3.6644 | 0.0928 | 1.1912 | 0.9373 | 3.0200 | 0.1381 | 1.1695 | 0.6325 | 4.2073 | 0.0475 | 1.2474 | 0.9295 | 3.4356 | 0.3820 | 1.6596 | 0.6235 | 3.0761 | 0.0482 | 1.9588 | 0.2333 | 2.8314 | 0.2150 | 1.8030 | 0.1697 |
| 283 | P62081 | 40S ribosomal protein S7 | RPS7 | 10 | 11 | 0.9550 | 0.4838 | 1.5849 | 0.4641 | 0.9204 | 0.5366 | 1.6293 | 0.2113 | 0.5012 | 0.0133 | 1.6144 | 0.1795 | 1.4060 | 0.1352 | 1.7701 | 0.0395 | 0.9638 | 0.7061 | 1.8880 | 0.0920 | 1.3428 | 0.5165 | 1.9770 | 0.0994 | 1.2359 | 0.9136 | 1.4191 | 0.6290 |
| 284 | P62753 | 40S ribosomal protein S6 (Phosphoprotein NP33) | RPS6 OK/SW-cl.2 | 12 | 9 | 1.2589 | 0.2911 | 1.0568 | 0.5440 | 1.3677 | 0.3160 | 0.6194 | 0.2199 | 1.5276 | 0.1623 | 1.0186 | 0.7723 | 1.8197 | 0.0643 | 1.1169 | 0.9063 | 1.6596 | 0.1016 | 0.9376 | 0.5401 | 2.4889 | 0.0156 | 0.8551 | 0.4223 | 1.2359 | 0.1662 | 1.1376 | 0.9861 |
| 285 | P46782 | 40S ribosomal protein S5 [Cleaved into: 40S ribosomal protein S5, N-terminally processed] | RPS5 | 10 | 11 | 3.8371 | 0.0198 | 0.6546 | 0.4550 | 1.9953 | 0.1230 | 0.8790 | 0.5584 | 2.7797 | 0.0907 | 0.7656 | 0.8059 | 4.2855 | 0.0088 | 0.9120 | 0.6937 | 3.2509 | 0.0170 | 1.0965 | 0.4062 | 2.3768 | 0.2869 | 0.8710 | 0.7950 | 1.0000 | 0.9696 | 1.4588 | 0.1431 |
| 286 | P62701 | 40S ribosomal protein S4, X isoform (SCR10) (Single copy abundant mRNA protein) | RPS4X CCG2 RPS4 SCAR | 8 | 15 | 0.6486 | 0.2015 | 1.1066 | 0.4032 | 0.7656 | 0.3836 | 1.1066 | 0.3066 | 0.7447 | 0.6842 | 1.7539 | 0.7503 | 0.8241 | 0.9799 | 1.5704 | 0.6847 | 0.6368 | 0.2932 | 2.1086 | 0.2400 | 1.1482 | 0.4587 | 1.9770 | 0.5933 | 0.5248 | 0.1287 | 2.0512 | 0.5170 |
| 287 | P23396 | 40S ribosomal protein S3 (EC 4.2.99.18) | RPS3 OK/SW-cl.26 | 9 | 20 | 0.6194 | 0.5463 | 0.9290 | 0.1818 | 1.2589 | 0.4982 | 1.0568 | 0.2902 | 0.9638 | 0.9778 | 1.3552 | 0.7692 | 1.3804 | 0.6566 | 1.1376 | 0.3795 | 0.9550 | 0.8919 | 1.3305 | 0.6741 | 1.8030 | 0.2099 | 0.8091 | 0.1167 | 1.1482 | 0.8807 | 1.6144 | 0.0992 |
| 288 | P62857 | 40S ribosomal protein S28 | RPS28 | 17 | 5 | 0.8790 | 0.3484 | 2.0324 | 0.2261 | 1.3677 | 0.8706 | 1.9409 | 0.2458 | 0.9290 | 0.7741 | 2.4434 | 0.1423 | 2.4660 | 0.0914 | 1.9231 | 0.2321 | 1.7378 | 0.6269 | 2.2699 | 0.1737 | 2.0701 | 0.2112 | 2.1878 | 0.1353 | 2.1086 | 0.1345 | 2.0512 | 0.1356 |
| 289 | P62851 | 40S ribosomal protein S25 | RPS25 | 21 | 3 | 0.9638 | 0.7933 | 4.3652 | 0.1549 | 1.1803 | 0.7074 | 4.0926 | 0.1841 | 1.0666 | 0.7771 | 2.3121 | 0.2258 | 1.5704 | 0.1380 | 3.1915 | 0.1781 | 1.5849 | 0.1730 | 4.2462 | 0.1276 | 1.8030 | 0.0034 | 4.9204 | 0.1257 | 1.7865 | 0.2460 | 3.7670 | 0.1123 |
| 290 | P62847 | 40S ribosomal protein S24 | RPS24 | 3 | 4 | 4.7863 | 0.2866 | 1.3552 | 0.4365 | 3.3419 | 0.2885 | 1.2246 | 0.3297 | 2.9107 | 0.2583 | 1.2706 | 0.7471 | 5.1523 | 0.1939 | 1.2823 | 0.4394 | 4.4463 | 0.3348 | 1.5276 | 0.1909 | 4.6559 | 0.2223 | 1.4060 | 0.3427 | 5.2966 | 0.2045 | 1.5849 | 0.2917 |
| 291 | P62266 | 40S ribosomal protein S23 | RPS23 | 3 | 12 | 2.1478 | 0.5775 | 5.8614 | 0.1194 | 2.1677 | 0.3615 | 4.3251 | 0.4320 | 1.8197 | 0.6170 | 1.4723 | 0.4388 | 2.8314 | 0.0816 | 3.9811 | 0.2948 | 2.1478 | 0.2265 | 2.6062 | 0.5958 | 2.8576 | 0.0947 | 5.8076 | 0.1006 | 2.0893 | 0.4392 | 1.5417 | 0.4528 |
| 292 | P60866 | 40S ribosomal protein S20 | RPS20 | 6 | 5 | 4.1687 | 0.2488 | 1.6444 | 0.1677 | 3.1333 | 0.4397 | 1.1588 | 0.9875 | 3.4674 | 0.2905 | 1.3552 | 0.5913 | 3.6644 | 0.3933 | 1.2942 | 0.4189 | 2.0137 | 0.8179 | 1.5560 | 0.4780 | 3.4995 | 0.2902 | 1.9409 | 0.2424 | 2.3335 | 0.7113 | 1.9055 | 0.1617 |
| 293 | P15880 | 40S ribosomal protein S2 (40S ribosomal protein S4) (Protein LLRep3) | RPS2 RPS4 | 9 | 13 | 10.8643 | 0.0184 | 0.8872 | 0.6155 | 12.0226 | 0.0175 | 1.0965 | 0.8161 | 12.5892 | 0.0139 | 1.2134 | 0.6562 | 16.1436 | 0.0103 | 0.9290 | 0.7368 | 13.3045 | 0.0135 | 1.2359 | 0.3288 | 5.5976 | 0.0166 | 1.0186 | 0.9551 | 2.4434 | 0.9331 | 1.1695 | 0.6936 |
| 294 | P39019 | 40S ribosomal protein S19 | RPS19 | 6 | 10 | 1.1272 | 0.7308 | 2.7040 | 0.2027 | 1.9231 | 0.5027 | 2.3335 | 0.2660 | 1.5849 | 0.7227 | 2.3550 | 0.5106 | 1.9409 | 0.4253 | 2.6546 | 0.2549 | 1.6596 | 0.6074 | 2.1677 | 0.4523 | 1.8197 | 0.5488 | 3.1915 | 0.1085 | 1.4588 | 0.7627 | 2.8314 | 0.1085 |
| 295 | P62269 | 40S ribosomal protein S18 (Ke-3) (Ke3) | RPS18 D6S218E | 11 | 8 | 0.9204 | 0.5140 | 1.4997 | 0.9935 | 1.1482 | 0.7464 | 1.1169 | 0.9019 | 1.2246 | 0.9600 | 1.1803 | 0.5602 | 1.3804 | 0.1460 | 0.5200 | 0.1471 | 1.2706 | 0.8483 | 1.5704 | 0.3745 | 1.5276 | 0.2267 | 1.3183 | 0.6234 | 0.8710 | 0.4171 | 2.0701 | 0.3935 |
| 296 | P08708 | 40S ribosomal protein S17 | RPS17 RPS17L | 10 | 10 | 1.6144 | 0.4366 | 0.8091 | 0.7634 | 1.9231 | 0.1326 | 1.1588 | 0.5095 | 1.8707 | 0.1053 | 1.1695 | 0.3173 | 2.2284 | 0.0453 | 0.8630 | 0.6044 | 1.9953 | 0.0937 | 1.0864 | 0.7281 | 2.1677 | 0.0125 | 1.3183 | 0.1684 | 2.0512 | 0.0502 | 0.9290 | 0.6224 |
| 297 | P62249 | 40S ribosomal protein S16 | RPS16 | 11 | 12 | 1.6749 | 0.2405 | 1.3305 | 0.3236 | 1.8197 | 0.0714 | 0.9462 | 0.8566 | 0.9817 | 0.4080 | 0.7379 | 0.6305 | 1.7378 | 0.0730 | 1.3804 | 0.2778 | 1.5849 | 0.1335 | 1.2589 | 0.0854 | 2.4889 | 0.0308 | 0.9638 | 0.6054 | 1.8030 | 0.0408 | 1.8030 | 0.0446 |
| 298 | P62244 | 40S ribosomal protein S15a | RPS15A OK/SW-cl.82 | 11 | 11 | 1.1272 | 0.5389 | 0.5445 | 0.0897 | 1.1066 | 0.6069 | 0.7586 | 0.4692 | 1.5276 | 0.2622 | 0.7943 | 0.4343 | 1.5849 | 0.3003 | 0.4831 | 0.1346 | 1.2134 | 0.5130 | 0.8872 | 0.5029 | 1.6144 | 0.1958 | 0.6252 | 0.2284 | 0.8790 | 0.8809 | 0.4786 | 0.7582 |
| 299 | P62841 | 40S ribosomal protein S15 (RIG protein) | RPS15 RIG | 11 | 15 | 1.3804 | 0.9400 | 2.9648 | 0.4901 | 0.9120 | 0.4413 | 2.4889 | 0.4828 | 0.5916 | 0.3396 | 3.0479 | 0.9188 | 1.7219 | 0.3269 | 1.9770 | 0.6960 | 0.8710 | 0.3775 | 4.1305 | 0.3724 | 1.9953 | 0.0362 | 5.2000 | 0.3284 | 1.2023 | 0.4678 | 8.2414 | 0.2797 |
| 300 | P62263 | 40S ribosomal protein S14 | RPS14 PRO2640 | 12 | 11 | 0.5861 | 0.1897 | 0.7244 | 0.8674 | 0.8017 | 0.2095 | 1.4060 | 0.1432 | 0.3221 | 0.0162 | 1.0375 | 0.6448 | 1.0000 | 0.6646 | 1.1482 | 0.9567 | 0.5495 | 0.2178 | 1.2246 | 0.5753 | 1.4723 | 0.2405 | 0.5445 | 0.9209 | 0.4613 | 0.1413 | 0.8630 | 0.4051 |
| 301 | P62277 | 40S ribosomal protein S13 | RPS13 | 15 | 9 | 0.2965 | 0.3378 | 1.1066 | 0.7343 | 0.4529 | 0.3144 | 0.9290 | 0.8155 | 1.9055 | 0.6071 | 1.0375 | 0.9370 | 0.3631 | 0.2876 | 0.7311 | 0.3837 | 1.9770 | 0.5677 | 1.2823 | 0.2512 | 0.4487 | 0.9104 | 1.3062 | 0.1563 | 1.1066 | 0.8951 | 1.4997 | 0.0216 |
| 302 | P25398 | 40S ribosomal protein S12 | RPS12 | 7 | 6 | 5.4954 | 0.0179 | 0.4018 | 0.3524 | 2.7797 | 0.0799 | 1.1376 | 0.7683 | 1.6293 | 0.2629 | 1.5417 | 0.7917 | 4.3652 | 0.0696 | 0.6668 | 0.3877 | 2.1281 | 0.0979 | 1.3183 | 0.9283 | 3.8371 | 0.0653 | 1.5996 | 0.7931 | 4.2855 | 0.0233 | 1.5417 | 0.5095 |
| 303 | P62280 | 40S ribosomal protein S11 | RPS11 | 8 | 8 | 1.7701 | 0.3377 | 0.7656 | 0.3562 | 1.8707 | 0.0672 | 1.1912 | 0.8071 | 1.2134 | 0.9128 | 1.6444 | 0.0868 | 2.8054 | 0.0030 | 1.3932 | 0.8157 | 2.0512 | 0.0322 | 1.5560 | 0.2352 | 3.1915 | 0.0010 | 1.6444 | 0.2370 | 2.0893 | 0.1352 | 1.8880 | 0.0558 |
| 304 | P46783 | 40S ribosomal protein S10 | RPS10 | 9 | 7 | 0.7943 | 0.3314 | 1.7219 | 0.1775 | 0.9638 | 0.6771 | 1.4859 | 0.2466 | 1.3428 | 0.3835 | 1.0280 | 0.9661 | 1.3062 | 0.4458 | 0.7311 | 0.4415 | 0.9550 | 0.8997 | 1.4997 | 0.6092 | 1.0965 | 0.6995 | 1.2589 | 0.6128 | 0.7516 | 0.4725 | 1.2023 | 0.7942 |
| 305 | P04844 | Dolichyl-diphosphooligosaccharide--protein glycosyltransferase subunit 2 (EC 2.4.99.18) (Dolichyl-diphosphooligosaccharide--protein glycosyltransferase 63 kDa subunit) (RIBIIR) (Ribophorin II) (RPN-II) (Ribophorin-2) | RPN2 | 7 | 20 | 1.2589 | 0.8978 | 1.2359 | 0.2671 | 1.1482 | 0.9589 | 1.2706 | 0.3331 | 1.5136 | 0.1905 | 1.3804 | 0.3784 | 1.5276 | 0.2063 | 1.5704 | 0.0699 | 1.4588 | 0.2934 | 1.3677 | 0.0789 | 2.0512 | 0.0003 | 0.9462 | 0.7673 | 1.6144 | 0.0364 | 0.8630 | 0.3168 |
| 306 | P04843 | Dolichyl-diphosphooligosaccharide--protein glycosyltransferase subunit 1 (EC 2.4.99.18) (Dolichyl-diphosphooligosaccharide--protein glycosyltransferase 67 kDa subunit) (Ribophorin I) (RPN-I) (Ribophorin-1) | RPN1 | 7 | 33 | 0.9462 | 0.8263 | 1.2359 | 0.8676 | 1.0965 | 0.4726 | 1.1169 | 0.6375 | 0.9204 | 0.6690 | 1.3804 | 0.5587 | 1.3062 | 0.2688 | 0.8395 | 0.1365 | 1.0765 | 0.8616 | 1.2474 | 0.7715 | 1.5996 | 0.0250 | 1.7378 | 0.0931 | 1.0471 | 0.7680 | 1.2823 | 0.8262 |
| 307 | P05387 | 60S acidic ribosomal protein P2 (Renal carcinoma antigen NY-REN-44) | RPLP2 D11S2243E RPP2 | 25 | 12 | 1.3183 | 0.2239 | 1.6596 | 0.3535 | 1.8535 | 0.0450 | 1.2942 | 0.7821 | 1.3183 | 0.0654 | 1.9055 | 0.2703 | 1.8707 | 0.0139 | 1.4859 | 0.5544 | 2.0701 | 0.0506 | 1.5849 | 0.2958 | 2.0512 | 0.0275 | 2.6546 | 0.1161 | 1.3932 | 0.1955 | 0.6918 | 0.3767 |
| 308 | P05386 | 60S acidic ribosomal protein P1 | RPLP1 RRP1 | 39 | 4 | 1.7061 | 0.5869 | 0.8472 | 0.9171 | 1.5849 | 0.3661 | 1.2246 | 0.9180 | 1.7701 | 0.0729 | 1.6293 | 0.8085 | 2.3121 | 0.0418 | 0.8091 | 0.8686 | 2.3121 | 0.0285 | 1.4723 | 0.8599 | 1.2823 | 0.8368 | 1.4060 | 0.7961 | 1.3804 | 0.7927 | 1.6596 | 0.6908 |
| 309 | P05388 | 60S acidic ribosomal protein P0 (60S ribosomal protein L10E) | RPLP0 | 20 | 15 | 0.8710 | 0.6042 | 0.5495 | 0.4287 | 1.4060 | 0.5053 | 1.0765 | 0.6502 | 1.6749 | 0.1601 | 1.1912 | 0.9907 | 1.6293 | 0.4962 | 1.0864 | 0.9848 | 1.3428 | 0.5038 | 1.3305 | 0.6023 | 1.0093 | 0.2252 | 1.1066 | 0.9806 | 0.7870 | 0.6676 | 1.0765 | 0.8167 |
| 310 | P32969 | 60S ribosomal protein L9 | RPL9 OK/SW-cl.103; RPL9P7; RPL9P8; RPL9P9 | 9 | 10 | 0.6730 | 0.5925 | 1.9231 | 0.3917 | 1.1066 | 0.7840 | 1.4997 | 0.3830 | 0.9908 | 0.8313 | 2.3335 | 0.1077 | 1.2246 | 0.7725 | 1.3552 | 0.7236 | 1.1272 | 0.9909 | 2.1086 | 0.1478 | 0.9290 | 0.9323 | 2.0893 | 0.1513 | 0.5546 | 0.6151 | 2.2491 | 0.2182 |
| 311 | P62917 | 60S ribosomal protein L8 | RPL8 | 19 | 6 | 1.3428 | 0.6665 | 3.5975 | 0.2396 | 1.6904 | 0.3057 | 2.9648 | 0.0419 | 1.3305 | 0.9733 | 1.3305 | 0.6408 | 2.2284 | 0.0550 | 1.8880 | 0.3482 | 1.4322 | 0.4923 | 3.3113 | 0.2498 | 2.1878 | 0.0015 | 3.2810 | 0.3083 | 1.1803 | 0.8729 | 2.5586 | 0.2835 |
| 312 | P18124 | 60S ribosomal protein L7 | RPL7 | 8 | 6 | 1.5704 | 0.6198 | 1.5276 | 0.9303 | 0.8630 | 0.8451 | 1.3677 | 0.7359 | 1.2942 | 0.9757 | 1.5849 | 0.5071 | 1.9231 | 0.2278 | 1.2589 | 0.1660 | 1.3677 | 0.6348 | 1.5704 | 0.6360 | 2.2284 | 0.0160 | 1.3932 | 0.7660 | 1.3932 | 0.2414 | 2.2909 | 0.0235 |
| 313 | Q02878 | 60S ribosomal protein L6 (Neoplasm-related protein C140) (Tax-responsive enhancer element-binding protein 107) (TaxREB107) | RPL6 TXREB1 | 5 | 11 | 5.1051 | 0.0601 | 1.0375 | 0.6663 | 4.3652 | 0.0500 | 0.7311 | 0.2073 | 3.6644 | 0.0317 | 0.7943 | 0.8332 | 4.8306 | 0.0084 | 0.3981 | 0.0194 | 4.6559 | 0.0353 | 1.0280 | 0.6156 | 3.9811 | 0.0718 | 0.7447 | 0.3244 | 3.6308 | 0.2255 | 1.5560 | 0.1173 |
| 314 | P36578 | 60S ribosomal protein L4 (60S ribosomal protein L1) | RPL4 RPL1 | 13 | 18 | 1.1482 | 0.4079 | 2.2699 | 0.0917 | 0.5546 | 0.0272 | 1.7061 | 0.2206 | 0.8872 | 0.0926 | 2.1677 | 0.0447 | 1.7701 | 0.4369 | 1.3677 | 0.7376 | 1.1588 | 0.2920 | 2.7542 | 0.0031 | 2.0512 | 0.0163 | 2.1281 | 0.1907 | 1.9770 | 0.1460 | 2.7290 | 0.0022 |
| 315 | P42766 | 60S ribosomal protein L35 | RPL35 | 12 | 4 | 0.4285 | 0.0905 | 4.3251 | 0.2456 | 0.7656 | 0.2804 | 3.0200 | 0.3374 | 0.2884 | 0.0553 | 0.6138 | 0.7957 | 0.9120 | 0.7143 | 2.8576 | 0.3247 | 1.0000 | 0.9269 | 3.3729 | 0.2518 | 1.3677 | 0.3196 | 3.0761 | 0.3418 | 1.1482 | 0.8004 | 3.2509 | 0.3863 |
| 316 | P49207 | 60S ribosomal protein L34 | RPL34 | 21 | 3 | 1.2246 | 0.8447 | 0.7870 | 0.9004 | 1.6596 | 0.7685 | 0.4875 | 0.3411 | 1.9953 | 0.5735 | 0.9908 | 0.9832 | 1.9588 | 0.3997 | 0.2148 | 0.1769 | 2.3988 | 0.1751 | 0.6081 | 0.3135 | 3.0761 | 0.0024 | 0.3311 | 0.1163 | 2.1478 | 0.0739 | 1.7219 | 0.3539 |
| 317 | P62899 | 60S ribosomal protein L31 | RPL31 | 4 | 5 | 3.2211 | 0.1400 | 3.1046 | 0.1120 | 2.1478 | 0.3086 | 3.0479 | 0.1182 | 4.1305 | 0.1068 | 1.6904 | 0.0482 | 4.1305 | 0.1000 | 2.5119 | 0.2119 | 3.7325 | 0.0628 | 3.4356 | 0.0109 | 3.4995 | 0.1266 | 4.2073 | 0.0069 | 3.6308 | 0.1714 | 3.0200 | 0.0812 |
| 318 | P62888 | 60S ribosomal protein L30 | RPL30 | 4 | 8 | 1.0471 | 0.6170 | 3.3729 | 0.1620 | 1.5996 | 0.9977 | 3.2211 | 0.1305 | 0.5808 | 0.4033 | 2.1478 | 0.1522 | 1.9409 | 0.6888 | 2.2080 | 0.2047 | 1.5560 | 0.8050 | 3.6644 | 0.1065 | 3.2810 | 0.1065 | 1.9231 | 0.4071 | 2.1086 | 0.3740 | 2.7542 | 0.0691 |
| 319 | P47914 | 60S ribosomal protein L29 (Cell surface heparin-binding protein HIP) | RPL29 | 8 | 3 | 2.6062 | 0.5515 | 1.6596 | 0.6953 | 2.3550 | 0.3597 | 2.6062 | 0.6454 | 3.0761 | 0.1693 | 3.1915 | 0.4259 | 2.6792 | 0.3033 | 3.2211 | 0.5744 | 3.6308 | 0.0742 | 2.0324 | 0.7058 | 2.0512 | 0.4842 | 2.2080 | 0.6363 | 3.2211 | 0.1862 | 2.9376 | 0.4653 |
| 320 | P46779 | 60S ribosomal protein L28 | RPL28 | 7 | 4 | 0.8395 | 0.4774 | 0.5495 | 0.2307 | 1.0186 | 0.5369 | 0.3597 | 0.1921 | 1.2589 | 0.1492 | 0.6668 | 0.5944 | 1.5276 | 0.1617 | 0.1180 | 0.0534 | 1.2023 | 0.3291 | 0.8551 | 0.7895 | 1.4322 | 0.2467 | 0.5916 | 0.2691 | 0.7516 | 0.6573 | 0.8395 | 0.9104 |
| 321 | P46776 | 60S ribosomal protein L27a | RPL27A | 4 | 5 | 2.6062 | 0.3807 | 4.6559 | 0.0040 | 1.5704 | 0.9239 | 1.7378 | 0.3037 | 1.7061 | 0.4797 | 3.4995 | 0.0078 | 2.2491 | 0.3885 | 2.2491 | 0.4306 | 1.9770 | 0.7098 | 4.7863 | 0.0047 | 2.6062 | 0.2527 | 4.4463 | 0.0671 | 1.9231 | 0.5768 | 4.9204 | 0.0028 |
| 322 | P61353 | 60S ribosomal protein L27 | RPL27 | 3 | 5 | 0.5970 | 0.1772 | 4.4055 | 0.0284 | 0.6486 | 0.1592 | 4.4055 | 0.0646 | 0.5346 | 0.0719 | 3.0479 | 0.1174 | 0.5702 | 0.3154 | 4.4463 | 0.0595 | 0.9204 | 0.5516 | 5.3951 | 0.0875 | 1.2246 | 0.8673 | 6.6681 | 0.0239 | 0.6081 | 0.3991 | 5.7544 | 0.0233 |
| 323 | Q9UNX3 | 60S ribosomal protein L26-like 1 | RPL26L1 RPL26P1 | 7 | 4 | 2.1677 | 0.0534 | 1.7378 | 0.6612 | 1.8535 | 0.0806 | 2.1478 | 0.6867 | 2.4889 | 0.0137 | 0.6730 | 0.2346 | 2.5823 | 0.0304 | 1.3062 | 0.6825 | 2.4660 | 0.0300 | 2.6792 | 0.1802 | 2.5351 | 0.0067 | 2.4210 | 0.1410 | 2.0701 | 0.1890 | 2.7797 | 0.1202 |
| 324 | P83731 | 60S ribosomal protein L24 (60S ribosomal protein L30) | RPL24 | 6 | 4 | 3.4041 | 0.0313 | 2.1478 | 0.9444 | 4.2073 | 0.0485 | 2.9648 | 0.4115 | 3.2810 | 0.0860 | 2.0137 | 0.8126 | 3.7670 | 0.0885 | 1.5417 | 0.7376 | 3.6308 | 0.2053 | 2.7290 | 0.3711 | 4.4875 | 0.0153 | 4.2462 | 0.2247 | 4.2073 | 0.0679 | 3.1333 | 0.1989 |
| 325 | P62829 | 60S ribosomal protein L23 (60S ribosomal protein L17) | RPL23 | 5 | 7 | 1.9055 | 0.2452 | 1.4859 | 0.1559 | 2.1086 | 0.1355 | 3.0761 | 0.0586 | 2.2909 | 0.1264 | 2.2080 | 0.1529 | 2.0512 | 0.2443 | 1.5417 | 0.7787 | 1.7701 | 0.2067 | 3.0479 | 0.0876 | 2.5351 | 0.1227 | 3.1046 | 0.0936 | 1.2942 | 0.4445 | 1.6749 | 0.1855 |
| 326 | P35268 | 60S ribosomal protein L22 (EBER-associated protein) (EAP) (Epstein-Barr virus small RNA-associated protein) (Heparin-binding protein HBp15) | RPL22 | 9 | 8 | 3.6983 | 0.3162 | 1.9953 | 0.3013 | 4.6989 | 0.1836 | 1.0093 | 0.4746 | 4.0179 | 0.2189 | 1.0375 | 0.5853 | 5.2000 | 0.2922 | 2.1281 | 0.3215 | 4.1305 | 0.3828 | 1.0965 | 0.2880 | 3.5645 | 0.0832 | 1.7219 | 0.3290 | 2.6303 | 0.4088 | 1.8707 | 0.2918 |
| 327 | P46778 | 60S ribosomal protein L21 | RPL21 | 6 | 6 | 2.5823 | 0.4623 | 0.9036 | 0.7587 | 4.3251 | 0.1307 | 1.0280 | 0.8602 | 3.2211 | 0.2499 | 1.1272 | 0.8318 | 4.9204 | 0.0871 | 1.2023 | 0.8441 | 3.0479 | 0.2962 | 1.3062 | 0.5732 | 3.4995 | 0.1613 | 1.7701 | 0.3471 | 0.9908 | 0.6260 | 1.8030 | 0.2542 |
| 328 | P84098 | 60S ribosomal protein L19 | RPL19 | 8 | 3 | 1.0000 | 0.7762 | 2.2491 | 0.5631 | 1.7219 | 0.2448 | 1.6749 | 0.7889 | 2.2080 | 0.0718 | 2.1878 | 0.6094 | 2.4660 | 0.1788 | 0.9036 | 0.5176 | 1.3552 | 0.5961 | 1.3804 | 0.6154 | 2.2909 | 0.0249 | 2.3988 | 0.8868 | 1.9231 | 0.2268 | 4.2855 | 0.1410 |
| 329 | Q07020 | 60S ribosomal protein L18 | RPL18 | 7 | 6 | 1.3305 | 0.8978 | 2.8576 | 0.5285 | 1.6144 | 0.6846 | 2.0512 | 0.8700 | 3.4356 | 0.7245 | 3.8726 | 0.1332 | 3.8019 | 0.5504 | 3.2810 | 0.1949 | 2.6546 | 0.7903 | 2.9376 | 0.3943 | 4.9204 | 0.3105 | 3.0479 | 0.4798 | 3.0479 | 0.5905 | 5.8614 | 0.0362 |
| 330 | P61313 | 60S ribosomal protein L15 | RPL15 EC45 TCBAP0781 | 6 | 5 | 1.4191 | 0.8533 | 1.1376 | 0.8648 | 0.6368 | 0.2369 | 0.7727 | 0.3756 | 1.2706 | 0.8346 | 0.5861 | 0.4969 | 1.5704 | 0.9051 | 0.4406 | 0.2177 | 1.2359 | 0.9148 | 0.7379 | 0.9105 | 2.1086 | 0.4461 | 0.5754 | 0.2798 | 1.6444 | 0.8860 | 1.6144 | 0.2586 |
| 331 | P50914 | 60S ribosomal protein L14 (CAG-ISL 7) | RPL14 | 7 | 7 | 0.6026 | 0.6538 | 2.1878 | 0.1752 | 0.8091 | 0.7583 | 1.8365 | 0.3890 | 0.8318 | 0.9371 | 1.8197 | 0.2164 | 0.9376 | 0.9434 | 1.4191 | 0.4254 | 0.9727 | 0.8999 | 1.7061 | 0.3706 | 1.6444 | 0.0990 | 2.7797 | 0.0602 | 0.4613 | 0.2510 | 2.3550 | 0.0709 |
| 332 | P26373 | 60S ribosomal protein L13 (Breast basic conserved protein 1) | RPL13 BBC1 OK/SW-cl.46 | 5 | 14 | 0.6194 | 0.4727 | 2.5586 | 0.2776 | 0.3802 | 0.1689 | 2.6062 | 0.3602 | 0.2512 | 0.2087 | 1.8707 | 0.4952 | 0.9462 | 0.7484 | 2.3768 | 0.6257 | 0.8017 | 0.9606 | 3.1623 | 0.2078 | 1.9953 | 0.0983 | 3.1915 | 0.1455 | 1.1272 | 0.6604 | 4.0551 | 0.0488 |
| 333 | P30050 | 60S ribosomal protein L12 | RPL12 | 6 | 11 | 0.5598 | 0.2253 | 1.0765 | 0.9723 | 1.7701 | 0.6404 | 1.3552 | 0.9451 | 1.4859 | 0.7778 | 1.5849 | 0.4451 | 1.9770 | 0.2980 | 1.4723 | 0.5048 | 1.5276 | 0.6239 | 1.6293 | 0.5671 | 1.8030 | 0.1525 | 1.6596 | 0.4601 | 1.1912 | 0.9339 | 1.6904 | 0.3037 |
| 334 | P62913 | 60S ribosomal protein L11 (CLL-associated antigen KW-12) | RPL11 | 13 | 3 | 2.5351 | 0.0287 | 0.4920 | 0.3319 | 2.1878 | 0.1237 | 0.5861 | 0.3222 | 2.9376 | 0.0059 | 0.9638 | 0.6758 | 2.5586 | 0.0374 | 0.7943 | 0.3425 | 2.7290 | 0.0178 | 0.8318 | 0.4837 | 3.9084 | 0.0006 | 0.8790 | 0.9596 | 2.8576 | 0.0221 | 1.0666 | 0.9234 |
| 335 | P62906 | 60S ribosomal protein L10a (CSA-19) (Neural precursor cell expressed developmentally down-regulated protein 6) (NEDD-6) | RPL10A NEDD6 | 10 | 5 | 1.0765 | 0.7882 | 1.2359 | 0.4743 | 1.1482 | 0.9600 | 1.5136 | 0.5945 | 1.4454 | 0.3555 | 1.5996 | 0.7820 | 1.6904 | 0.7857 | 1.8707 | 0.6677 | 1.2823 | 0.6957 | 1.6444 | 0.8322 | 1.4997 | 0.2883 | 1.9770 | 0.8631 | 1.1482 | 0.6526 | 1.5996 | 0.5398 |
| 336 | P27635 | 60S ribosomal protein L10 (Laminin receptor homolog) (Protein QM) (Tumor suppressor QM) | RPL10 DXS648E QM | 3 | 7 | 0.5598 | 0.7763 | 0.9290 | 0.4553 | 0.5754 | 0.4218 | 0.4966 | 0.1313 | 1.1482 | 0.9789 | 0.8954 | 0.3330 | 1.3062 | 0.8256 | 0.5445 | 0.0584 | 0.8166 | 0.8119 | 0.8241 | 0.6946 | 1.6596 | 0.2034 | 1.7378 | 0.9130 | 1.0280 | 0.9744 | 2.0137 | 0.5120 |
| 337 | O75116 | Rho-associated protein kinase 2 (EC 2.7.11.1) (Rho kinase 2) (Rho-associated, coiled-coil-containing protein kinase 2) (Rho-associated, coiled-coil-containing protein kinase II) (ROCK-II) (p164 ROCK-2) | ROCK2 KIAA0619 | 9 | 3 | 1.1803 | 0.9902 | 1.2134 | 0.5086 | 1.3677 | 0.5443 | 0.5546 | 0.1526 | 1.2589 | 0.9567 | 1.4588 | 0.2786 | 1.7865 | 0.8018 | 1.2246 | 0.4945 | 1.3932 | 0.8804 | 1.2134 | 0.5084 | 2.6546 | 0.1104 | 0.9638 | 0.9498 | 1.7219 | 0.5087 | 1.4454 | 0.2879 |
| 338 | Q9H4A4 | Aminopeptidase B (AP-B) (EC 3.4.11.6) (Arginine aminopeptidase) (Arginyl aminopeptidase) | RNPEP APB | 9 | 16 | 0.7311 | 0.0886 | 0.8630 | 0.7967 | 0.9817 | 0.4542 | 0.3076 | 0.0131 | 0.7586 | 0.3333 | 0.9817 | 0.4120 | 1.2246 | 0.5770 | 0.5248 | 0.1273 | 0.9817 | 0.7846 | 0.4966 | 0.0181 | 1.5417 | 0.0419 | 0.8395 | 0.8741 | 0.7586 | 0.1784 | 0.6792 | 0.5202 |
| 339 | P13489 | Ribonuclease inhibitor (Placental ribonuclease inhibitor) (Placental RNase inhibitor) (Ribonuclease/angiogenin inhibitor 1) (RAI) | RNH1 PRI RNH | 3 | 16 | 0.6546 | 0.4804 | 1.7378 | 0.8767 | 1.2134 | 0.6912 | 1.5704 | 0.5847 | 0.8166 | 0.7119 | 3.1623 | 0.2433 | 0.7586 | 0.6184 | 2.0324 | 0.2676 | 0.9376 | 0.9146 | 1.7378 | 0.8271 | 0.8017 | 0.6838 | 2.5119 | 0.3180 | 0.6668 | 0.4963 | 3.3419 | 0.0809 |
| 340 | Q9NPQ8 | Synembryn-A (Protein Ric-8A) | RIC8A | 14 | 4 | 0.9908 | 0.2924 |  |  | 0.9204 | 0.2004 |  |  | 0.9638 | 0.3991 |  |  | 0.9290 | 0.3630 |  |  | 0.8954 | 0.1757 |  |  | 0.9462 | 0.2605 |  |  | 1.0864 | 0.7068 |  |  |
| 341 | P61586 | Transforming protein RhoA (Rho cDNA clone 12) (h12) | RHOA ARH12 ARHA RHO12 | 17 | 11 | 1.0280 | 0.7039 | 0.9817 | 0.8771 | 0.9727 | 0.2640 | 1.0000 | 0.8171 | 1.0864 | 0.9662 | 0.9908 | 0.8206 | 1.0186 | 0.6549 | 0.9290 | 0.9586 | 0.9550 | 0.1334 | 1.2246 | 0.6332 | 1.0471 | 0.4102 | 1.0965 | 0.8815 | 0.9908 | 0.4243 | 1.1272 | 0.8345 |
| 342 | Q15493 | Regucalcin (RC) (Gluconolactonase) (GNL) (EC 3.1.1.17) (Senescence marker protein 30) (SMP-30) | RGN SMP30 | 3 | 20 | 0.7178 | 0.5629 | 0.7244 | 0.8158 | 0.9120 | 0.8663 | 1.0093 | 0.5368 | 1.9055 | 0.3431 | 1.1588 | 0.1426 | 1.1912 | 0.7231 | 0.7379 | 0.7693 | 2.2080 | 0.2899 | 1.3305 | 0.1183 | 1.1695 | 0.7432 | 1.8535 | 0.0006 | 2.1677 | 0.2957 | 0.4325 | 0.0181 |
| 343 | Q6NUM9 | All-trans-retinol 13,14-reductase (EC 1.3.99.23) (All-trans-13,14-dihydroretinol saturase) (RetSat) (PPAR-alpha-regulated and starvation-induced gene protein) | RETSAT PPSIG UNQ439/PRO872 | 9 | 4 | 0.8318 | 0.3498 | 1.0186 | 0.5734 | 1.2589 | 0.8851 | 1.3932 | 0.4749 | 0.8241 | 0.3067 | 1.2359 | 0.4936 | 0.8872 | 0.2100 | 1.3677 | 0.4867 | 0.9462 | 0.3322 | 1.6293 | 0.4470 | 1.9953 | 0.1794 | 1.1588 | 0.5064 | 1.7061 | 0.7669 | 1.1912 | 0.5130 |
| 344 | Q96HR9 | Receptor expression-enhancing protein 6 (Polyposis locus protein 1-like 1) | REEP6 C19orf32 DP1L1 | 20 | 4 | 0.9376 | 0.7881 | 0.9727 | 0.9234 | 1.0186 | 0.7362 | 1.0965 | 0.9244 | 1.9953 | 0.0005 | 0.7727 | 0.3812 | 0.7047 | 0.5619 | 1.4588 | 0.4893 | 1.6749 | 0.0053 | 1.3677 | 0.5965 | 0.0724 | 0.0000 | 2.0893 | 0.0577 | 0.3251 | 0.0087 | 1.6144 | 0.3645 |
| 345 | Q00765 | Receptor expression-enhancing protein 5 (Polyposis locus protein 1) (Protein TB2) | REEP5 C5orf18 DP1 TB2 | 3 | 4 | 1.3932 | 0.3312 | 2.6062 | 0.1942 | 1.0093 | 0.8658 | 1.0765 | 0.6156 | 1.3552 | 0.4110 | 1.2706 | 0.8501 | 1.0000 | 0.6842 | 2.6303 | 0.2022 | 0.4529 | 0.1448 | 1.4588 | 0.8569 | 0.8630 | 0.9656 | 2.2909 | 0.3556 | 1.0093 | 0.8876 | 1.8880 | 0.4885 |
| 346 | P35241 | Radixin | RDX | 5 | 23 | 1.4859 | 0.6981 | 0.8954 | 0.8152 | 1.6293 | 0.4081 | 1.0000 | 0.9654 | 1.3552 | 0.7730 | 1.2134 | 0.3893 | 1.2706 | 0.6705 | 1.1695 | 0.7622 | 1.1272 | 0.9355 | 1.2023 | 0.7608 | 0.4742 | 0.2725 | 1.4060 | 0.2640 | 1.1272 | 0.8466 | 0.7943 | 0.3361 |
| 347 | O75452 | Retinol dehydrogenase 16 (EC 1.1.-.-) (Microsomal NAD(+)-dependent retinol dehydrogenase 4) (RoDH-4) (Short chain dehydrogenase/reductase family 9C member 8) (Sterol/retinol dehydrogenase) | RDH16 RODH4 SDR9C8 | 3 | 25 | 1.0965 | 0.1936 | 0.8017 | 0.2645 | 1.3183 | 0.4785 | 1.6293 | 0.0117 | 1.2023 | 0.3645 | 0.5861 | 0.0566 | 1.5996 | 0.1016 | 0.5200 | 0.0017 | 1.5136 | 0.1372 | 0.6194 | 0.1315 | 0.7943 | 0.8775 | 0.4742 | 0.0017 | 0.8166 | 0.7476 | 0.1393 | 0.0000 |
| 348 | Q8NBN7 | Retinol dehydrogenase 13 (EC 1.1.1.-) (Short chain dehydrogenase/reductase family 7C member 3) | RDH13 SDR7C3 PSEC0082 UNQ736/PRO1430 | 24 | 4 | 0.6918 | 0.0662 | 1.9231 | 0.2309 | 0.8241 | 0.3975 | 1.2823 | 0.6001 | 0.8790 | 0.7005 | 0.4656 | 0.6912 | 0.5754 | 0.0723 | 1.7219 | 0.6205 | 0.6546 | 0.0536 | 1.4859 | 0.4432 | 0.3981 | 0.0014 | 1.8535 | 0.4948 | 0.4246 | 0.0006 | 3.3729 | 0.0771 |
| 349 | Q8TC12 | Retinol dehydrogenase 11 (EC 1.1.1.300) (Androgen-regulated short-chain dehydrogenase/reductase 1) (HCV core-binding protein HCBP12) (Prostate short-chain dehydrogenase/reductase 1) (Retinal reductase 1) (RalR1) (Short chain dehydrogenase/reductase family 7C member 1) | RDH11 ARSDR1 PSDR1 SDR7C1 CGI-82 | 25 | 8 | 0.3597 | 0.0001 | 1.2474 | 0.0576 | 0.9290 | 0.4978 | 1.0765 | 0.4971 | 0.7379 | 0.0696 | 1.0666 | 0.3401 | 1.0186 | 0.7951 | 1.0765 | 0.6435 | 1.1588 | 0.3599 | 1.1912 | 0.1260 | 0.1259 | 0.0000 | 1.2246 | 0.0887 | 0.1585 | 0.0000 | 1.0965 | 0.5196 |
| 350 | Q15293 | Reticulocalbin-1 | RCN1 RCN | 3 | 7 | 0.5105 | 0.4834 | 0.6310 | 0.1125 | 0.4656 | 0.5171 | 0.6607 | 0.1176 | 0.7447 | 0.4928 | 0.9462 | 0.6999 | 1.4191 | 0.6295 | 0.5598 | 0.0909 | 1.1912 | 0.7437 | 0.6026 | 0.1115 | 0.3767 | 0.5176 | 0.6855 | 0.2126 | 0.7870 | 0.6036 | 0.6792 | 0.1858 |
| 351 | P02753 | Retinol-binding protein 4 (Plasma retinol-binding protein) (PRBP) (RBP) [Cleaved into: Plasma retinol-binding protein(1-182); Plasma retinol-binding protein(1-181); Plasma retinol-binding protein(1-179); Plasma retinol-binding protein(1-176)] | RBP4 PRO2222 | 3 | 14 | 0.2399 | 0.4114 | 1.0471 | 0.5153 | 0.7943 | 0.6172 | 0.5152 | 0.4710 | 1.0765 | 0.9658 | 1.6749 | 0.2722 | 0.2655 | 0.1687 | 0.6252 | 0.5216 | 0.3467 | 0.2313 | 1.5417 | 0.3231 | 0.4207 | 0.2101 | 1.2359 | 0.6389 | 0.2148 | 0.0628 | 1.3932 | 0.2850 |
| 352 | O95153 | Peripheral-type benzodiazepine receptor-associated protein 1 (PRAX-1) (Peripheral benzodiazepine receptor-interacting protein) (PBR-IP) (RIMS-binding protein 1) (RIM-BP1) | BZRAP1 KIAA0612 RBP1 RIMBP1 | 6 | 6 | 0.9550 | 0.7822 | 0.5649 | 0.1503 | 0.9817 | 0.7690 | 0.6081 | 0.2796 | 1.1588 | 0.4154 | 0.8710 | 0.5453 | 1.0965 | 0.6758 | 0.5152 | 0.2683 | 1.1066 | 0.7224 | 0.8166 | 0.9391 | 0.9638 | 0.4978 | 0.5808 | 0.1442 | 0.9376 | 0.6115 | 0.9638 | 0.3040 |
| 353 | P09455 | Retinol-binding protein 1 (Cellular retinol-binding protein) (CRBP) (Cellular retinol-binding protein I) (CRBP-I) | RBP1 CRBP1 | 18 | 5 | 0.9462 | 0.3184 | 1.2589 | 0.4161 | 1.8535 | 0.0944 | 1.3552 | 0.4076 | 0.5297 | 0.2198 | 1.3305 | 0.3515 | 2.3550 | 0.0095 | 0.6792 | 0.8002 | 0.5445 | 0.2790 | 1.1272 | 0.3332 | 1.0093 | 0.7917 | 1.2823 | 0.4713 | 0.3076 | 0.2658 | 0.7244 | 0.5884 |
| 354 | Q96PK6 | RNA-binding protein 14 (Paraspeckle protein 2) (PSP2) (RNA-binding motif protein 14) (RRM-containing coactivator activator/modulator) (Synaptotagmin-interacting protein) (SYT-interacting protein) | RBM14 SIP | 7 | 5 | 0.1888 | 0.1820 | 1.2246 | 0.1708 | 1.7378 | 0.7001 | 1.0093 | 0.7634 | 0.6194 | 0.3501 | 0.9376 | 0.6597 | 0.5598 | 0.3281 | 1.2134 | 0.3181 | 1.0375 | 0.9499 | 1.1803 | 0.2256 | 1.2134 | 0.9089 | 0.9204 | 0.9631 | 1.0666 | 0.9232 | 1.1272 | 0.9622 |
| 355 | P54136 | Arginine--tRNA ligase, cytoplasmic (EC 6.1.1.19) (Arginyl-tRNA synthetase) (ArgRS) | RARS | 4 | 13 | 1.0864 | 0.5149 | 1.1588 | 0.6596 | 1.1482 | 0.2526 | 0.8166 | 0.5820 | 1.1912 | 0.5281 | 1.7061 | 0.1492 | 1.0864 | 0.6807 | 0.8551 | 0.7037 | 1.1695 | 0.5115 | 1.4191 | 0.5708 | 1.1272 | 0.1605 | 1.1272 | 0.5684 | 1.0965 | 0.4509 | 1.2134 | 0.3514 |
| 356 | P61224 | Ras-related protein Rap-1b (GTP-binding protein smg p21B) | RAP1B OK/SW-cl.11 | 4 | 11 | 1.8535 | 0.0596 | 2.5351 | 0.1108 | 1.1695 | 0.5502 | 1.4859 | 0.1092 | 1.2359 | 0.6031 | 1.8030 | 0.1590 | 0.9908 | 0.9879 | 2.2080 | 0.1031 | 0.8872 | 0.6746 | 2.4889 | 0.0366 | 1.1376 | 0.6464 | 1.5276 | 0.3634 | 1.6144 | 0.1150 | 1.1066 | 0.6527 |
| 357 | P62826 | GTP-binding nuclear protein Ran (Androgen receptor-associated protein 24) (GTPase Ran) (Ras-like protein TC4) (Ras-related nuclear protein) | RAN ARA24 OK/SW-cl.81 | 14 | 3 | 2.1478 | 0.0449 | 0.2754 | 0.0390 | 1.5276 | 0.5161 | 0.5058 | 0.2612 | 2.0324 | 0.0702 | 0.6026 | 0.3772 | 1.5996 | 0.3966 | 0.6252 | 0.1808 | 2.0701 | 0.0503 | 0.7943 | 0.6233 | 1.7219 | 0.1623 | 0.6546 | 0.4346 | 2.2909 | 0.0460 | 0.7656 | 0.7357 |
| 358 | Q92878 | DNA repair protein RAD50 (hRAD50) (EC 3.6.-.-) | RAD50 | 17 | 3 | 1.6596 | 0.9733 | 1.0186 | 0.9516 | 1.9231 | 0.2865 | 0.7586 | 0.6105 | 1.5849 | 0.9683 | 0.7244 | 0.5645 | 1.8030 | 0.8606 | 0.8872 | 0.8260 | 1.8197 | 0.7686 | 0.7727 | 0.6359 | 1.8197 | 0.8833 | 0.6546 | 0.4740 | 1.3428 | 0.7802 | 0.9638 | 0.9588 |
| 359 | P54727 | UV excision repair protein RAD23 homolog B (HR23B) (hHR23B) (XP-C repair-complementing complex 58 kDa protein) (p58) | RAD23B | 7 | 10 | 0.6607 | 0.2884 | 0.8241 | 0.8155 | 0.8954 | 0.6126 | 2.2491 | 0.6893 | 0.8551 | 0.9628 | 3.3729 | 0.1823 | 0.7943 | 0.1431 | 1.7701 | 0.5531 | 0.8091 | 0.3103 | 1.6144 | 0.4179 | 1.5560 | 0.0405 | 3.0761 | 0.2540 | 0.9908 | 0.7202 | 1.3932 | 0.7537 |
| 360 | P63000 | Ras-related C3 botulinum toxin substrate 1 (Cell migration-inducing gene 5 protein) (Ras-like protein TC25) (p21-Rac1) | RAC1 TC25 MIG5 | 3 | 6 | 1.0186 | 0.9747 | 0.6138 | 0.4347 | 0.8790 | 0.5430 | 0.4656 | 0.3032 | 0.8790 | 0.7015 | 0.6607 | 0.4863 | 0.5970 | 0.1623 | 0.7943 | 0.6703 | 0.9550 | 0.8397 | 0.7379 | 0.5882 | 0.8395 | 0.4834 | 0.5916 | 0.4120 | 0.9376 | 0.7828 | 0.8630 | 0.7881 |
| 361 | Q92930 | Ras-related protein Rab-8B | RAB8B | 10 | 7 | 0.7047 | 0.3122 | 0.7112 | 0.5505 | 0.8091 | 0.3919 | 0.9290 | 0.9003 | 1.1588 | 0.9071 | 1.7061 | 0.3995 | 1.0765 | 0.8635 | 1.3677 | 0.5655 | 0.9120 | 0.5291 | 0.8954 | 0.8436 | 0.4613 | 0.2329 | 1.0864 | 0.8498 | 0.8630 | 0.7612 | 1.2589 | 0.6496 |
| 362 | P61006 | Ras-related protein Rab-8A (Oncogene c-mel) | RAB8A MEL RAB8 | 6 | 8 | 0.8472 | 0.1748 | 0.9462 | 0.8669 | 0.9290 | 0.8270 | 0.7727 | 0.0136 | 0.9036 | 0.4014 | 0.8790 | 0.3039 | 0.9462 | 0.9112 | 1.0765 | 0.2713 | 0.6138 | 0.1219 | 0.9204 | 0.1410 | 1.0864 | 0.8160 | 0.9550 | 0.6827 | 0.9120 | 0.9095 | 1.0765 | 0.3539 |
| 363 | P51149 | Ras-related protein Rab-7a | RAB7A RAB7 | 6 | 10 | 1.6444 | 0.9427 | 0.9376 | 0.4189 | 3.7670 | 0.3123 | 1.3552 | 0.9894 | 3.8726 | 0.3251 | 1.4191 | 0.4392 | 1.9231 | 0.9168 | 0.9727 | 0.2573 | 2.2909 | 0.8549 | 1.4191 | 0.9760 | 4.7863 | 0.2568 | 1.4723 | 0.9660 | 4.2855 | 0.2958 | 0.9376 | 0.6044 |
| 364 | P20340 | Ras-related protein Rab-6A (Rab-6) | RAB6A RAB6 | 6 | 5 | 0.6918 | 0.2454 | 1.1066 | 0.8530 | 0.9290 | 0.6104 | 0.9036 | 0.9539 | 0.6546 | 0.1389 | 1.1272 | 0.7348 | 0.6730 | 0.1443 | 0.7656 | 0.7601 | 0.6427 | 0.1464 | 1.1482 | 0.6441 | 1.0965 | 0.6992 | 1.1482 | 0.8006 | 0.9290 | 0.9533 | 1.0471 | 0.7931 |
| 365 | P51148 | Ras-related protein Rab-5C (L1880) (RAB5L) | RAB5C RABL | 12 | 8 | 1.1803 | 0.5101 | 1.2823 | 0.9121 | 1.0765 | 0.9979 | 1.5704 | 0.3291 | 1.5276 | 0.2625 | 1.8197 | 0.1953 | 1.1695 | 0.5542 | 1.6144 | 0.4318 | 1.0568 | 0.6059 | 1.1066 | 0.7237 | 1.3183 | 0.1225 | 1.2246 | 0.8705 | 0.6855 | 0.4241 | 1.5136 | 0.4868 |
| 366 | P61020 | Ras-related protein Rab-5B | RAB5B | 7 | 4 | 0.4875 | 0.0491 | 0.6081 | 0.3693 | 0.8710 | 0.5929 | 0.4571 | 0.2405 | 0.8710 | 0.8883 | 0.8091 | 0.6535 | 0.7656 | 0.5600 | 0.7244 | 0.5089 | 0.8318 | 0.8465 | 0.8017 | 0.6348 | 1.3183 | 0.1988 | 0.6368 | 0.4038 | 1.1912 | 0.3477 | 0.5861 | 0.3476 |
| 367 | P61019 | Ras-related protein Rab-2A | RAB2A RAB2 | 8 | 8 | 3.8019 | 0.0998 | 0.9120 | 0.7501 | 1.9953 | 0.4196 | 1.0471 | 0.6023 | 2.4889 | 0.1872 | 0.5916 | 0.3221 | 3.1915 | 0.0379 | 0.9120 | 0.9051 | 1.2706 | 0.7908 | 0.7311 | 0.6514 | 2.1086 | 0.3777 | 0.4613 | 0.1194 | 2.6792 | 0.0819 | 1.0000 | 0.9606 |
| 368 | Q9H0U4 | Ras-related protein Rab-1B | RAB1B | 3 | 10 | 0.8710 | 0.7964 | 0.5200 | 0.5467 | 1.1482 | 0.7734 | 0.5012 | 0.4938 | 0.9638 | 0.9606 | 0.2630 | 0.4182 | 1.0280 | 0.9337 | 0.3281 | 0.4455 | 1.3428 | 0.5824 | 0.7311 | 0.7573 | 0.4365 | 0.2831 | 0.7586 | 0.9039 | 0.6138 | 0.4362 | 1.0186 | 0.9725 |
| 369 | P62820 | Ras-related protein Rab-1A (YPT1-related protein) | RAB1A RAB1 | 8 | 13 | 0.9908 | 0.6773 | 0.8472 | 0.5987 | 0.9204 | 0.6120 | 1.0093 | 0.2514 | 0.8551 | 0.3840 | 1.2474 | 0.0922 | 1.1066 | 0.8508 | 1.2706 | 0.0896 | 0.8872 | 0.4646 | 1.5560 | 0.0522 | 0.8872 | 0.2442 | 0.9376 | 0.3625 | 0.7798 | 0.3043 | 1.1695 | 0.1112 |
| 370 | P61106 | Ras-related protein Rab-14 | RAB14 | 11 | 9 | 0.7798 | 0.6558 | 1.7865 | 0.0503 | 0.7870 | 0.6649 | 1.5704 | 0.2600 | 0.5916 | 0.4127 | 1.5704 | 0.2671 | 0.7447 | 0.6012 | 1.0186 | 0.8716 | 0.7178 | 0.5571 | 1.5417 | 0.3686 | 0.5445 | 0.3696 | 1.8365 | 0.2071 | 0.8551 | 0.7766 | 1.2474 | 0.8480 |
| 371 | Q15907 | Ras-related protein Rab-11B (GTP-binding protein YPT3) | RAB11B YPT3 | 10 | 9 | 0.8710 | 0.5994 | 1.0864 | 0.5354 | 1.1482 | 0.5819 | 0.9290 | 0.4863 | 0.7870 | 0.3095 | 1.0093 | 0.9720 | 1.0186 | 0.9274 | 1.1169 | 0.5482 | 0.7798 | 0.3398 | 1.1272 | 0.4619 | 1.2823 | 0.3749 | 1.0471 | 0.8213 | 0.9727 | 0.8774 | 1.0965 | 0.6887 |
| 372 | P61026 | Ras-related protein Rab-10 | RAB10 | 12 | 9 | 1.0471 | 0.8626 | 3.6983 | 0.3865 | 1.0093 | 0.9636 | 2.7290 | 0.7288 | 1.0093 | 0.8023 | 1.8197 | 0.8932 | 1.0093 | 0.9424 | 2.4210 | 0.4633 | 1.0186 | 0.9092 | 1.9055 | 0.8953 | 0.9550 | 0.4544 | 2.6062 | 0.5787 | 0.9817 | 0.8667 | 2.3335 | 0.4567 |
| 373 | Q15274 | Nicotinate-nucleotide pyrophosphorylase [carboxylating] (EC 2.4.2.19) (Quinolinate phosphoribosyltransferase [decarboxylating]) (QAPRTase) (QPRTase) | QPRT | 8 | 19 | 0.9204 | 0.6592 | 1.4454 | 0.4819 | 0.8472 | 0.2893 | 1.0765 | 0.7772 | 1.0186 | 0.9751 | 1.5136 | 0.3630 | 1.1912 | 0.5574 | 2.3335 | 0.1093 | 1.2474 | 0.9650 | 1.5417 | 0.3796 | 1.7061 | 0.0842 | 1.6144 | 0.6616 | 1.7219 | 0.0985 | 1.6293 | 0.5859 |
| 374 | P09417 | Dihydropteridine reductase (EC 1.5.1.34) (HDHPR) (Quinoid dihydropteridine reductase) (Short chain dehydrogenase/reductase family 33C member 1) | QDPR DHPR SDR33C1 | 9 | 6 | 1.7701 | 0.2369 | 0.8017 | 0.6856 | 2.5823 | 0.2021 | 1.2589 | 0.6526 | 2.7290 | 0.1379 | 0.5297 | 0.3538 | 1.6749 | 0.2699 | 0.5861 | 0.4080 | 2.2080 | 0.2872 | 0.6026 | 0.4242 | 2.5351 | 0.1506 | 0.6792 | 0.5076 | 3.1623 | 0.1610 | 0.3837 | 0.2496 |
| 375 | P47897 | Glutamine--tRNA ligase (EC 6.1.1.18) (Glutaminyl-tRNA synthetase) (GlnRS) | QARS | 14 | 25 | 1.4191 | 0.7332 | 0.4406 | 0.0045 | 1.4322 | 0.4102 | 0.9120 | 0.1211 | 1.9231 | 0.1123 | 0.5248 | 0.0063 | 0.9817 | 0.9183 | 0.7379 | 0.1074 | 1.1695 | 0.8061 | 0.6310 | 0.0065 | 0.3467 | 0.0505 | 0.8091 | 0.0893 | 0.9204 | 0.6530 | 0.1803 | 0.0005 |
| 376 | P06737 | Glycogen phosphorylase, liver form (EC 2.4.1.1) | PYGL | 27 | 101 | 0.9204 | 0.3955 | 1.6293 | 0.0919 | 0.7943 | 0.3274 | 1.4997 | 0.7356 | 0.8091 | 0.7532 | 1.3932 | 0.8178 | 0.7870 | 0.1507 | 0.3981 | 0.0036 | 0.4656 | 0.0069 | 1.8030 | 0.0006 | 0.2780 | 0.0003 | 1.6904 | 0.0286 | 0.2679 | 0.0188 | 0.6855 | 0.0134 |
| 377 | P11216 | Glycogen phosphorylase, brain form (EC 2.4.1.1) | PYGB | 16 | 35 | 1.2942 | 0.2227 | 1.2134 | 0.7786 | 0.8241 | 0.5033 | 1.2589 | 0.7173 | 1.6144 | 0.0927 | 1.6904 | 0.2290 | 1.3932 | 0.2581 | 1.9055 | 0.0985 | 1.3183 | 0.2192 | 2.0512 | 0.1137 | 0.9290 | 0.4399 | 2.1878 | 0.1190 | 1.4060 | 0.1267 | 0.8318 | 0.1109 |
| 378 | Q9ULZ3 | Apoptosis-associated speck-like protein containing a CARD (hASC) (Caspase recruitment domain-containing protein 5) (PYD and CARD domain-containing protein) (Target of methylation-induced silencing 1) | PYCARD ASC CARD5 TMS1 | 104 | 3 | 1.0000 | 0.2907 | 1.3428 | 0.2992 | 1.1695 | 0.4976 | 0.7447 | 0.7325 | 1.0375 | 0.5004 | 1.3305 | 0.4968 | 1.2474 | 0.2610 | 0.2582 | 0.2222 | 1.0864 | 0.5990 | 1.2134 | 0.6339 | 0.1159 | 0.0000 | 0.3311 | 0.1485 | 0.2188 | 0.0000 | 1.5849 | 0.3559 |
| 379 | Q9NR77 | Peroxisomal membrane protein 2 (22 kDa peroxisomal membrane protein) | PXMP2 PMP22 | 31 | 9 | 0.6668 | 0.2011 | 1.4191 | 0.1568 | 0.9727 | 0.9963 | 0.9376 | 0.6486 | 0.6427 | 0.1851 | 0.7379 | 0.3805 | 1.1588 | 0.6696 | 1.0965 | 0.3114 | 1.3552 | 0.2198 | 1.0000 | 0.5725 | 0.6668 | 0.0973 | 0.7447 | 0.2648 | 0.7870 | 0.4910 | 0.8166 | 0.6561 |
| 380 | Q00577 | Transcriptional activator protein Pur-alpha (Purine-rich single-stranded DNA-binding protein alpha) | PURA PUR1 | 4 | 6 | 0.8318 | 0.5777 | 0.8241 | 0.7853 | 1.3183 | 0.4075 | 0.9204 | 0.9814 | 1.1912 | 0.5658 | 0.5395 | 0.4278 | 1.4322 | 0.3110 | 1.0000 | 0.6816 | 1.1588 | 0.6161 | 1.1066 | 0.7919 | 2.4210 | 0.0821 | 1.1066 | 0.7964 | 2.0512 | 0.1172 | 0.7727 | 0.2956 |
| 381 | Q9UHX1 | Poly(U)-binding-splicing factor PUF60 (60 kDa poly(U)-binding-splicing factor) (FUSE-binding protein-interacting repressor) (FBP-interacting repressor) (Ro-binding protein 1) (RoBP1) (Siah-binding protein 1) (Siah-BP1) | PUF60 FIR ROBPI SIAHBP1 | 6 | 6 | 1.2706 | 0.3954 | 0.9204 | 0.9874 | 0.6194 | 0.3192 | 0.9550 | 0.7185 | 0.8472 | 0.9408 | 0.7112 | 0.8860 | 0.9462 | 0.9527 | 1.7701 | 0.0944 | 1.4588 | 0.3017 | 1.3932 | 0.2001 | 0.5495 | 0.1304 | 1.8707 | 0.0547 | 1.3677 | 0.6558 | 1.2474 | 0.6793 |
| 382 | Q6NZI2 | Polymerase I and transcript release factor (Cavin-1) | PTRF FKSG13 | 4 | 10 | 0.6546 | 0.7663 | 3.1333 | 0.1417 | 1.3552 | 0.6566 | 5.7016 | 0.0134 | 1.6144 | 0.5091 | 2.5823 | 0.1004 | 1.3183 | 0.5678 | 2.2699 | 0.2027 | 0.7870 | 0.8641 | 2.0701 | 0.3816 | 1.1272 | 0.7049 | 3.2211 | 0.1022 | 0.5598 | 0.5183 | 2.8840 | 0.1148 |
| 383 | P29350 | Tyrosine-protein phosphatase non-receptor type 6 (EC 3.1.3.48) (Hematopoietic cell protein-tyrosine phosphatase) (Protein-tyrosine phosphatase 1C) (PTP-1C) (Protein-tyrosine phosphatase SHP-1) (SH-PTP1) | PTPN6 HCP PTP1C | 3 | 4 | 0.9462 | 0.8821 | 0.8954 | 0.5596 | 0.9204 | 0.9586 | 1.0186 | 0.8217 | 1.1588 | 0.4441 | 1.1482 | 0.9232 | 1.1695 | 0.2815 | 1.4997 | 0.1458 | 1.0864 | 0.6193 | 1.1588 | 0.4700 | 1.1482 | 0.3169 | 1.1482 | 0.4393 | 1.2023 | 0.2224 | 1.2589 | 0.3130 |
| 384 | Q9P035 | Very-long-chain (3R)-3-hydroxyacyl-CoA dehydratase 3 (EC 4.2.1.134) (3-hydroxyacyl-CoA dehydratase 3) (HACD3) (Butyrate-induced protein 1) (B-ind1) (hB-ind1) (Protein-tyrosine phosphatase-like A domain-containing protein 1) | HACD3 BIND1 PTPLAD1 | 8 | 8 | 1.0666 | 0.7840 | 1.8707 | 0.0394 | 1.3552 | 0.3433 | 1.5417 | 0.1835 | 1.1482 | 0.3663 | 1.0666 | 0.9424 | 1.1803 | 0.4088 | 1.0965 | 0.7149 | 1.3183 | 0.3431 | 1.2246 | 0.3492 | 1.0765 | 0.4179 | 1.1272 | 0.7058 | 1.1376 | 0.4211 | 1.1695 | 0.6973 |
| 385 | P20962 | Parathymosin | PTMS | 3 | 3 | 1.4060 | 0.1792 | 0.6730 | 0.7058 | 0.8710 | 0.8035 | 0.5808 | 0.4543 | 1.0093 | 0.9898 | 0.5248 | 0.5747 | 1.1272 | 0.4885 | 0.9638 | 0.7722 | 0.8630 | 0.7198 | 0.7178 | 0.5785 | 1.6444 | 0.1056 | 0.9908 | 0.7790 | 1.5417 | 0.1291 | 0.1368 | 0.1905 |
| 386 | Q8N8N7 | Prostaglandin reductase 2 (PRG-2) (EC 1.3.1.48) (15-oxoprostaglandin 13-reductase) (Zinc-binding alcohol dehydrogenase domain-containing protein 1) | PTGR2 ZADH1 | 7 | 4 | 1.1169 | 0.1424 | 1.1066 | 0.7779 | 0.8630 | 0.8441 | 0.8872 | 0.2564 | 0.8954 | 0.9903 | 1.0471 | 0.9371 | 1.0965 | 0.4371 | 0.7379 | 0.3555 | 1.2023 | 0.1157 | 1.1272 | 0.4549 | 1.0280 | 0.5840 | 0.9204 | 0.2580 | 1.0765 | 0.2146 | 1.0568 | 0.8513 |
| 387 | Q14914 | Prostaglandin reductase 1 (PRG-1) (EC 1.3.1.-) (15-oxoprostaglandin 13-reductase) (EC 1.3.1.48) (NADP-dependent leukotriene B4 12-hydroxydehydrogenase) (EC 1.3.1.74) | PTGR1 LTB4DH | 7 | 36 | 0.9290 | 0.5856 | 0.4786 | 0.0029 | 0.5248 | 0.3415 | 1.2134 | 0.8134 | 0.7112 | 0.7101 | 1.1066 | 0.8526 | 0.6982 | 0.6377 | 0.8017 | 0.1775 | 0.8241 | 0.6670 | 0.1629 | 0.0000 | 0.0745 | 0.1077 | 0.6668 | 0.1669 | 0.4207 | 0.2925 | 0.3945 | 0.0002 |
| 388 | P26599 | Polypyrimidine tract-binding protein 1 (PTB) (57 kDa RNA-binding protein PPTB-1) (Heterogeneous nuclear ribonucleoprotein I) (hnRNP I) | PTBP1 PTB | 3 | 15 | 1.0965 | 0.8948 | 2.2080 | 0.2479 | 0.7112 | 0.4702 | 2.7290 | 0.3369 | 0.9462 | 0.8027 | 2.4434 | 0.3728 | 1.1803 | 0.6255 | 1.2589 | 0.8523 | 1.4723 | 0.5368 | 3.4356 | 0.1467 | 0.3192 | 0.3337 | 1.8365 | 0.8237 | 0.4325 | 0.5340 | 1.9588 | 0.6187 |
| 389 | Q9UL46 | Proteasome activator complex subunit 2 (11S regulator complex subunit beta) (REG-beta) (Activator of multicatalytic protease subunit 2) (Proteasome activator 28 subunit beta) (PA28b) (PA28beta) | PSME2 | 38 | 21 | 0.8166 | 0.3084 | 1.0375 | 0.4269 | 0.0258 | 0.0000 | 0.8241 | 0.8923 | 0.9036 | 0.8308 | 2.6062 | 0.0736 | 1.0186 | 0.7938 | 0.6194 | 0.5411 | 0.7656 | 0.1333 | 2.5823 | 0.0104 | 0.2911 | 0.0000 | 1.3428 | 0.9318 | 0.5808 | 0.0001 | 3.2211 | 0.0012 |
| 390 | Q06323 | Proteasome activator complex subunit 1 (11S regulator complex subunit alpha) (REG-alpha) (Activator of multicatalytic protease subunit 1) (Interferon gamma up-regulated I-5111 protein) (IGUP I-5111) (Proteasome activator 28 subunit alpha) (PA28a) (PA28alpha) | PSME1 IFI5111 | 17 | 19 | 1.7219 | 0.6908 | 0.7727 | 0.3849 | 1.9953 | 0.2730 | 0.5200 | 0.1355 | 1.0186 | 0.7963 | 1.6144 | 0.0029 | 1.8365 | 0.3431 | 0.5105 | 0.1443 | 1.6596 | 0.2669 | 1.3552 | 0.0361 | 2.6546 | 0.0084 | 0.8395 | 0.3297 | 1.9055 | 0.3589 | 1.6444 | 0.0186 |
| 391 | P48556 | 26S proteasome non-ATPase regulatory subunit 8 (26S proteasome regulatory subunit RPN12) (26S proteasome regulatory subunit S14) (p31) | PSMD8 | 26 | 4 | 0.4446 | 0.2713 | 1.7701 | 0.5698 | 1.1066 | 0.9736 | 2.0701 | 0.5473 | 1.1066 | 0.2105 | 1.8365 | 0.3923 | 0.6081 | 0.3993 | 1.9953 | 0.2153 | 1.6444 | 0.1255 | 2.7040 | 0.1838 | 1.6293 | 0.0126 | 2.1878 | 0.2032 | 2.1878 | 0.0148 | 2.2080 | 0.4519 |
| 392 | P51665 | 26S proteasome non-ATPase regulatory subunit 7 (26S proteasome regulatory subunit RPN8) (26S proteasome regulatory subunit S12) (Mov34 protein homolog) (Proteasome subunit p40) | PSMD7 MOV34L | 26 | 5 | 0.5970 | 0.0200 | 1.3305 | 0.4439 | 0.6918 | 0.1570 | 0.8954 | 0.3562 | 0.7727 | 0.4542 | 1.3552 | 0.3996 | 0.4406 | 0.0249 | 1.1588 | 0.9537 | 0.7586 | 0.7996 | 1.0765 | 0.7826 | 1.0965 | 0.8392 | 0.9817 | 0.9505 | 0.9908 | 0.8650 | 1.3183 | 0.3246 |
| 393 | Q16401 | 26S proteasome non-ATPase regulatory subunit 5 (26S protease subunit S5 basic) (26S proteasome subunit S5B) | PSMD5 KIAA0072 | 4 | 6 | 0.7870 | 0.3690 | 2.9648 | 0.1523 | 1.0186 | 0.9923 | 1.4191 | 0.9729 | 0.9638 | 0.8174 | 1.6904 | 0.7426 | 1.1169 | 0.6013 | 0.8395 | 0.8608 | 0.9727 | 0.9287 | 1.5996 | 0.8272 | 0.8166 | 0.2209 | 0.6855 | 0.4285 | 0.9376 | 0.7512 | 1.4859 | 0.9163 |
| 394 | O43242 | 26S proteasome non-ATPase regulatory subunit 3 (26S proteasome regulatory subunit RPN3) (26S proteasome regulatory subunit S3) (Proteasome subunit p58) | PSMD3 | 4 | 7 | 0.3873 | 0.3224 | 0.6427 | 0.1845 | 0.6730 | 0.6347 | 0.5012 | 0.0866 | 1.1169 | 0.9931 | 1.3305 | 0.4447 | 0.7311 | 0.8116 | 1.2023 | 0.5449 | 0.4966 | 0.5575 | 1.2359 | 0.6460 | 0.7047 | 0.7247 | 0.8395 | 0.5822 | 0.5346 | 0.4732 | 1.2023 | 0.7066 |
| 395 | Q13200 | 26S proteasome non-ATPase regulatory subunit 2 (26S proteasome regulatory subunit RPN1) (26S proteasome regulatory subunit S2) (26S proteasome subunit p97) (Protein 55.11) (Tumor necrosis factor type 1 receptor-associated protein 2) | PSMD2 TRAP2 | 10 | 14 | 0.8318 | 0.5868 | 0.5546 | 0.1358 | 0.6310 | 0.5516 | 0.9204 | 0.8830 | 1.0093 | 0.9341 | 0.6310 | 0.4446 | 0.9120 | 0.5717 | 0.6982 | 0.5224 | 1.1912 | 0.8046 | 0.9727 | 0.6184 | 0.8241 | 0.3399 | 0.6730 | 0.4423 | 0.8872 | 0.9376 | 0.5248 | 0.0843 |
| 396 | O00487 | 26S proteasome non-ATPase regulatory subunit 14 (EC 3.4.19.-) (26S proteasome regulatory subunit RPN11) (26S proteasome-associated PAD1 homolog 1) | PSMD14 POH1 | 5 | 9 | 0.9462 | 0.8570 | 5.3951 | 0.0293 | 1.0280 | 0.6745 | 3.7670 | 0.3771 | 0.9204 | 0.6732 | 4.7424 | 0.0457 | 0.9120 | 0.5024 | 2.3988 | 0.4005 | 1.0280 | 0.5818 | 4.7424 | 0.2264 | 1.0375 | 0.7056 | 3.5975 | 0.3681 | 1.0864 | 0.6937 | 2.5586 | 0.3149 |
| 397 | Q9UNM6 | 26S proteasome non-ATPase regulatory subunit 13 (26S proteasome regulatory subunit RPN9) (26S proteasome regulatory subunit S11) (26S proteasome regulatory subunit p40.5) | PSMD13 | 10 | 9 | 0.9638 | 0.6460 | 0.7943 | 0.7015 | 1.1272 | 0.3290 | 1.6749 | 0.4468 | 1.1066 | 0.4112 | 1.7378 | 0.4945 | 0.9817 | 0.9567 | 1.4723 | 0.3376 | 0.9550 | 0.5474 | 1.5276 | 0.4710 | 0.9817 | 0.8297 | 0.7047 | 0.4214 | 1.0471 | 0.2934 | 1.1066 | 0.8001 |
| 398 | O00232 | 26S proteasome non-ATPase regulatory subunit 12 (26S proteasome regulatory subunit RPN5) (26S proteasome regulatory subunit p55) | PSMD12 | 7 | 6 | 1.6444 | 0.4088 | 0.3499 | 0.0138 | 3.2810 | 0.0320 | 0.5346 | 0.4984 | 4.0926 | 0.0336 | 1.0186 | 0.6608 | 4.4875 | 0.0208 | 1.0965 | 0.7314 | 3.1623 | 0.0322 | 1.0965 | 0.8270 | 2.1878 | 0.1047 | 0.8091 | 0.7221 | 2.6303 | 0.1480 | 1.1169 | 0.3870 |
| 399 | O00231 | 26S proteasome non-ATPase regulatory subunit 11 (26S proteasome regulatory subunit RPN6) (26S proteasome regulatory subunit S9) (26S proteasome regulatory subunit p44.5) | PSMD11 | 5 | 6 | 1.6596 | 0.2217 | 1.0280 | 0.9679 | 1.1695 | 0.5241 | 0.5861 | 0.1620 | 0.8017 | 0.7475 | 1.4322 | 0.2054 | 1.0471 | 0.6944 | 1.0093 | 0.8440 | 2.0512 | 0.0636 | 1.1376 | 0.6306 | 1.8365 | 0.1164 | 1.3305 | 0.1891 | 1.2246 | 0.5266 | 1.4859 | 0.0823 |
| 400 | Q99460 | 26S proteasome non-ATPase regulatory subunit 1 (26S proteasome regulatory subunit RPN2) (26S proteasome regulatory subunit S1) (26S proteasome subunit p112) | PSMD1 | 9 | 9 | 0.6026 | 0.2235 | 0.8790 | 0.2730 | 0.5649 | 0.1994 | 0.8395 | 0.2659 | 0.8166 | 0.6650 | 0.9638 | 0.6011 | 0.6252 | 0.2268 | 0.9120 | 0.5269 | 0.6194 | 0.0966 | 0.9908 | 0.8761 | 0.8017 | 0.5954 | 0.9204 | 0.5857 | 0.7379 | 0.3780 | 0.9204 | 0.5995 |
| 401 | P62333 | 26S protease regulatory subunit 10B (26S proteasome AAA-ATPase subunit RPT4) (Proteasome 26S subunit ATPase 6) (Proteasome subunit p42) | PSMC6 SUG2 | 7 | 11 | 0.5152 | 0.3627 | 0.4325 | 0.2008 | 0.9036 | 0.5011 | 1.1912 | 0.5966 | 0.8551 | 0.4891 | 1.4723 | 0.2462 | 0.6138 | 0.3232 | 1.0666 | 0.8631 | 1.2359 | 0.3346 | 1.1169 | 0.4834 | 0.8166 | 0.9655 | 1.4997 | 0.4387 | 0.8630 | 0.5935 | 0.9036 | 0.7771 |
| 402 | P62195 | 26S protease regulatory subunit 8 (26S proteasome AAA-ATPase subunit RPT6) (Proteasome 26S subunit ATPase 5) (Proteasome subunit p45) (Thyroid hormone receptor-interacting protein 1) (TRIP1) (p45/SUG) | PSMC5 SUG1 | 7 | 7 | 0.9462 | 0.5537 | 1.1272 | 0.8065 | 1.0186 | 0.8807 | 0.7798 | 0.6011 | 0.9120 | 0.4779 | 2.2699 | 0.1807 | 0.8318 | 0.2653 | 1.5276 | 0.4951 | 1.0280 | 0.6687 | 2.2699 | 0.4081 | 1.0186 | 0.8261 | 2.7542 | 0.0933 | 0.9817 | 0.9895 | 1.9231 | 0.7033 |
| 403 | P43686 | 26S protease regulatory subunit 6B (26S proteasome AAA-ATPase subunit RPT3) (MB67-interacting protein) (MIP224) (Proteasome 26S subunit ATPase 4) (Tat-binding protein 7) (TBP-7) | PSMC4 MIP224 TBP7 | 10 | 11 | 0.7112 | 0.2341 | 1.8365 | 0.0829 | 1.0666 | 0.8341 | 1.9588 | 0.0537 | 0.8318 | 0.3364 | 2.4210 | 0.0138 | 1.2246 | 0.9460 | 2.0893 | 0.0305 | 0.4966 | 0.0921 | 1.6749 | 0.0491 | 0.9638 | 0.8396 | 1.0000 | 0.0533 | 0.7379 | 0.1907 | 0.5808 | 0.2543 |
| 404 | P17980 | 26S protease regulatory subunit 6A (26S proteasome AAA-ATPase subunit RPT5) (Proteasome 26S subunit ATPase 3) (Proteasome subunit P50) (Tat-binding protein 1) (TBP-1) | PSMC3 TBP1 | 8 | 16 | 0.7311 | 0.5607 | 0.9036 | 0.9437 | 1.1803 | 0.9597 | 1.1912 | 0.1867 | 0.9036 | 0.8390 | 1.9770 | 0.0049 | 0.8710 | 0.6272 | 1.6293 | 0.1724 | 1.3677 | 0.7290 | 1.2359 | 0.7879 | 0.8166 | 0.5973 | 1.4454 | 0.3650 | 1.8707 | 0.1606 | 1.2706 | 0.1626 |
| 405 | P35998 | 26S protease regulatory subunit 7 (26S proteasome AAA-ATPase subunit RPT1) (Proteasome 26S subunit ATPase 2) (Protein MSS1) | PSMC2 MSS1 | 11 | 10 | 1.7539 | 0.4809 | 1.0000 | 0.9542 | 1.3932 | 0.8787 | 1.1066 | 0.4510 | 2.5823 | 0.0930 | 1.0666 | 0.6975 | 0.9290 | 0.6960 | 1.0765 | 0.7003 | 2.4434 | 0.1425 | 0.9817 | 0.9030 | 1.7219 | 0.2726 | 0.9908 | 0.9462 | 2.2699 | 0.3589 | 1.0186 | 0.8527 |
| 406 | P62191 | 26S protease regulatory subunit 4 (P26s4) (26S proteasome AAA-ATPase subunit RPT2) (Proteasome 26S subunit ATPase 1) | PSMC1 | 9 | 8 | 1.0280 | 0.9577 | 1.1482 | 0.2448 | 0.9376 | 0.9613 | 1.0965 | 0.2020 | 1.8197 | 0.0334 | 1.0965 | 0.7782 | 1.3305 | 0.3545 | 1.1588 | 0.1773 | 1.2589 | 0.6913 | 1.0666 | 0.4623 | 1.1588 | 0.7614 | 1.1912 | 0.1236 | 1.4191 | 0.3117 | 1.1912 | 0.1171 |
| 407 | P28065 | Proteasome subunit beta type-9 (EC 3.4.25.1) (Low molecular mass protein 2) (Macropain chain 7) (Multicatalytic endopeptidase complex chain 7) (Proteasome chain 7) (Proteasome subunit beta-1i) (Really interesting new gene 12 protein) | PSMB9 LMP2 PSMB6i RING12 | 8 | 7 | 0.9638 | 0.4787 | 2.1086 | 0.0505 | 1.0568 | 0.9232 | 0.3945 | 0.1103 | 1.3183 | 0.4944 | 2.4210 | 0.0616 | 0.6026 | 0.3430 | 1.1066 | 0.9814 | 0.4446 | 0.0342 | 1.7378 | 0.2464 | 1.6904 | 0.3200 | 0.9462 | 0.6241 | 1.1912 | 0.9577 | 2.5586 | 0.0483 |
| 408 | P28062 | Proteasome subunit beta type-8 (EC 3.4.25.1) (Low molecular mass protein 7) (Macropain subunit C13) (Multicatalytic endopeptidase complex subunit C13) (Proteasome component C13) (Proteasome subunit beta-5i) (Really interesting new gene 10 protein) | PSMB8 LMP7 PSMB5i RING10 Y2 | 7 | 8 | 2.3768 | 0.0936 | 1.0765 | 0.6117 | 2.3768 | 0.0234 | 0.6855 | 0.5034 | 2.8840 | 0.0136 | 1.4997 | 0.0735 | 2.5119 | 0.0346 | 0.6427 | 0.2781 | 2.6546 | 0.0565 | 1.2706 | 0.4197 | 2.0512 | 0.1297 | 0.2780 | 0.0678 | 1.6444 | 0.1933 | 1.4859 | 0.0872 |
| 409 | Q99436 | Proteasome subunit beta type-7 (EC 3.4.25.1) (Macropain chain Z) (Multicatalytic endopeptidase complex chain Z) (Proteasome subunit Z) | PSMB7 Z | 6 | 7 | 1.0186 | 0.9377 | 1.0864 | 0.8753 | 1.9770 | 0.2075 | 1.0568 | 0.9170 | 2.8840 | 0.0965 | 1.2823 | 0.5914 | 2.0893 | 0.1637 | 0.8954 | 0.7806 | 0.6730 | 0.8501 | 1.0765 | 0.8437 | 2.0893 | 0.1836 | 1.1695 | 0.7734 | 2.4434 | 0.0931 | 1.0375 | 0.9439 |
| 410 | P28074 | Proteasome subunit beta type-5 (EC 3.4.25.1) (Macropain epsilon chain) (Multicatalytic endopeptidase complex epsilon chain) (Proteasome chain 6) (Proteasome epsilon chain) (Proteasome subunit MB1) (Proteasome subunit X) | PSMB5 LMPX MB1 X | 8 | 6 | 1.0765 | 0.9885 | 0.7311 | 0.2571 | 2.1478 | 0.2015 | 0.6194 | 0.0434 | 1.3804 | 0.6125 | 0.3698 | 0.0427 | 1.4322 | 0.7277 | 0.7244 | 0.1564 | 2.0512 | 0.2367 | 0.5702 | 0.0321 | 1.9231 | 0.0500 | 0.6252 | 0.0665 | 1.8365 | 0.2991 | 0.5649 | 0.0200 |
| 411 | P28070 | Proteasome subunit beta type-4 (EC 3.4.25.1) (26 kDa prosomal protein) (HsBPROS26) (PROS-26) (Macropain beta chain) (Multicatalytic endopeptidase complex beta chain) (Proteasome beta chain) (Proteasome chain 3) (HsN3) | PSMB4 PROS26 | 7 | 15 | 1.0965 | 0.9540 | 0.8318 | 0.8666 | 0.8954 | 0.3806 | 1.2942 | 0.3711 | 1.0280 | 0.7140 | 0.7311 | 0.5914 | 1.1482 | 0.9032 | 1.4322 | 0.1354 | 0.9727 | 0.9143 | 1.5136 | 0.5088 | 0.9204 | 0.2358 | 1.3305 | 0.4358 | 0.9036 | 0.6126 | 1.1376 | 0.4213 |
| 412 | P49720 | Proteasome subunit beta type-3 (EC 3.4.25.1) (Proteasome chain 13) (Proteasome component C10-II) (Proteasome theta chain) | PSMB3 | 5 | 7 | 2.1281 | 0.3166 | 0.9908 | 0.9338 | 1.0965 | 0.6138 | 0.9290 | 0.5740 | 1.2589 | 0.9331 | 1.8535 | 0.2268 | 1.7701 | 0.5235 | 1.2023 | 0.9804 | 0.9036 | 0.6391 | 1.4454 | 0.2673 | 0.4571 | 0.2209 | 1.4723 | 0.5161 | 1.2359 | 0.8113 | 1.4723 | 0.2568 |
| 413 | P49721 | Proteasome subunit beta type-2 (EC 3.4.25.1) (Macropain subunit C7-I) (Multicatalytic endopeptidase complex subunit C7-I) (Proteasome component C7-I) | PSMB2 | 16 | 7 | 1.0186 | 0.9018 | 1.0093 | 0.7265 | 0.9817 | 0.5462 | 0.9727 | 0.5841 | 0.9817 | 0.8925 | 1.5417 | 0.6831 | 0.9908 | 0.8488 | 1.2023 | 0.9957 | 0.9638 | 0.8005 | 1.0864 | 0.8021 | 0.9638 | 0.3740 | 0.8790 | 0.3675 | 0.9638 | 0.3302 | 1.2134 | 0.8246 |
| 414 | P40306 | Proteasome subunit beta type-10 (EC 3.4.25.1) (Low molecular mass protein 10) (Macropain subunit MECl-1) (Multicatalytic endopeptidase complex subunit MECl-1) (Proteasome MECl-1) (Proteasome subunit beta-2i) | PSMB10 LMP10 MECL1 | 7 | 3 | 0.8318 | 0.7615 | 1.1169 | 0.9381 | 1.0568 | 0.7691 | 0.9908 | 0.9756 | 1.1066 | 0.4626 | 1.9055 | 0.4040 | 0.7586 | 0.5684 | 1.0093 | 0.8455 | 1.2023 | 0.4968 | 0.8241 | 0.5379 | 0.8630 | 0.9397 | 0.8166 | 0.6796 | 1.3428 | 0.1883 | 1.4723 | 0.8004 |
| 415 | P20618 | Proteasome subunit beta type-1 (EC 3.4.25.1) (Macropain subunit C5) (Multicatalytic endopeptidase complex subunit C5) (Proteasome component C5) (Proteasome gamma chain) | PSMB1 PSC5 | 8 | 19 | 0.6026 | 0.7628 | 1.1272 | 0.8031 | 1.0471 | 0.9197 | 0.9727 | 0.6462 | 0.9817 | 0.8874 | 2.0893 | 0.3413 | 1.1066 | 0.9492 | 0.7311 | 0.6485 | 1.1482 | 0.8828 | 1.8197 | 0.5745 | 1.3804 | 0.7548 | 1.7061 | 0.9714 | 1.3677 | 0.6414 | 2.1878 | 0.2341 |
| 416 | O14818 | Proteasome subunit alpha type-7 (EC 3.4.25.1) (Proteasome subunit RC6-1) (Proteasome subunit XAPC7) | PSMA7 HSPC | 4 | 11 | 0.4487 | 0.5378 | 0.6982 | 0.9851 | 1.1912 | 0.8309 | 1.4723 | 0.2546 | 0.6194 | 0.7113 | 2.9648 | 0.0447 | 0.1977 | 0.5016 | 1.1588 | 0.5997 | 1.1482 | 0.9285 | 2.0512 | 0.2470 | 2.3335 | 0.3776 | 1.6596 | 0.3529 | 1.8030 | 0.3411 | 1.5560 | 0.2734 |
| 417 | P60900 | Proteasome subunit alpha type-6 (EC 3.4.25.1) (27 kDa prosomal protein) (PROS-27) (p27K) (Macropain iota chain) (Multicatalytic endopeptidase complex iota chain) (Proteasome iota chain) | PSMA6 PROS27 | 17 | 7 | 0.8318 | 0.9590 | 0.9638 | 0.7950 | 0.7244 | 0.5457 | 0.9550 | 0.8718 | 0.5754 | 0.5557 | 1.3677 | 0.4782 | 0.5970 | 0.3245 | 0.9462 | 0.5233 | 0.6730 | 0.1177 | 1.3804 | 0.4996 | 0.9550 | 0.5731 | 1.4723 | 0.5612 | 0.8166 | 0.3572 | 1.3552 | 0.4601 |
| 418 | P28066 | Proteasome subunit alpha type-5 (EC 3.4.25.1) (Macropain zeta chain) (Multicatalytic endopeptidase complex zeta chain) (Proteasome zeta chain) | PSMA5 | 11 | 11 | 1.0568 | 0.4240 | 1.9231 | 0.6241 | 1.0186 | 0.6052 | 1.8030 | 0.6313 | 1.0280 | 0.5169 | 2.9107 | 0.2316 | 1.0375 | 0.5074 | 1.0093 | 0.6111 | 1.0666 | 0.3343 | 2.4660 | 0.2340 | 1.0000 | 0.7687 | 2.0701 | 0.3312 | 1.0765 | 0.5547 | 1.8707 | 0.2002 |
| 419 | P25789 | Proteasome subunit alpha type-4 (EC 3.4.25.1) (Macropain subunit C9) (Multicatalytic endopeptidase complex subunit C9) (Proteasome component C9) (Proteasome subunit L) | PSMA4 HC9 PSC9 | 11 | 16 | 0.7244 | 0.2628 | 0.9036 | 0.9808 | 0.8790 | 0.3656 | 1.3677 | 0.8192 | 1.0765 | 0.6137 | 1.1169 | 0.9619 | 1.1482 | 0.9487 | 0.9204 | 0.5181 | 1.1376 | 0.9957 | 1.7378 | 0.3727 | 1.1169 | 0.7729 | 0.7798 | 0.8885 | 0.7798 | 0.4546 | 1.7219 | 0.2017 |
| 420 | P25788 | Proteasome subunit alpha type-3 (EC 3.4.25.1) (Macropain subunit C8) (Multicatalytic endopeptidase complex subunit C8) (Proteasome component C8) | PSMA3 HC8 PSC8 | 11 | 8 | 1.1588 | 0.1524 | 1.2823 | 0.6055 | 1.1272 | 0.7495 | 1.2023 | 0.6479 | 1.1912 | 0.8745 | 2.3550 | 0.0753 | 1.1482 | 0.5462 | 1.6904 | 0.3277 | 1.0471 | 0.7993 | 2.7040 | 0.0415 | 1.0093 | 0.9865 | 1.4588 | 0.7929 | 1.1169 | 0.6005 | 2.3335 | 0.0447 |
| 421 | P25787 | Proteasome subunit alpha type-2 (EC 3.4.25.1) (Macropain subunit C3) (Multicatalytic endopeptidase complex subunit C3) (Proteasome component C3) | PSMA2 HC3 PSC3 | 16 | 11 | 0.8472 | 0.9322 | 3.4674 | 0.0629 | 0.7656 | 0.4497 | 3.2211 | 0.1679 | 1.0093 | 0.4845 | 3.6983 | 0.1270 | 0.8241 | 0.6404 | 2.9107 | 0.3608 | 1.1695 | 0.3712 | 4.4463 | 0.0347 | 1.0568 | 0.4714 | 3.7670 | 0.2434 | 1.3677 | 0.2764 | 3.2509 | 0.0961 |
| 422 | P25786 | Proteasome subunit alpha type-1 (EC 3.4.25.1) (30 kDa prosomal protein) (PROS-30) (Macropain subunit C2) (Multicatalytic endopeptidase complex subunit C2) (Proteasome component C2) (Proteasome nu chain) | PSMA1 HC2 NU PROS30 PSC2 | 10 | 15 | 0.4446 | 0.1930 | 1.1482 | 0.6455 | 0.6918 | 0.3481 | 1.5704 | 0.2841 | 0.7311 | 0.4906 | 1.9953 | 0.0260 | 0.8166 | 0.5351 | 1.3062 | 0.3644 | 0.5970 | 0.6266 | 1.5849 | 0.2732 | 0.8710 | 0.5862 | 2.1281 | 0.0442 | 1.0093 | 0.9467 | 1.5704 | 0.1506 |
| 423 | Q9Y617 | Phosphoserine aminotransferase (EC 2.6.1.52) (Phosphohydroxythreonine aminotransferase) (PSAT) | PSAT1 PSA | 14 | 22 | 1.6144 | 0.1658 | 0.9290 | 0.8239 | 1.6749 | 0.2535 | 1.3428 | 0.1741 | 2.4434 | 0.0152 | 0.4656 | 0.0189 | 3.0200 | 0.0117 | 0.8241 | 0.6686 | 1.8535 | 0.1087 | 1.0864 | 0.6583 | 2.2080 | 0.1095 | 0.7727 | 0.8401 | 1.6144 | 0.1353 | 0.2679 | 0.0051 |
| 424 | P07602 | Prosaposin (Proactivator polypeptide) [Cleaved into: Saposin-A (Protein A); Saposin-B-Val; Saposin-B (Cerebroside sulfate activator) (CSAct) (Dispersin) (Sphingolipid activator protein 1) (SAP-1) (Sulfatide/GM1 activator); Saposin-C (A1 activator) (Co-beta-glucosidase) (Glucosylceramidase activator) (Sphingolipid activator protein 2) (SAP-2); Saposin-D (Component C) (Protein C)] | PSAP GLBA SAP1 | 14 | 7 | 1.2474 | 0.2827 | 0.3837 | 0.4740 | 1.0765 | 0.4542 | 1.0093 | 0.9530 | 0.9462 | 0.5971 | 1.5560 | 0.6623 | 0.9204 | 0.9402 | 1.2706 | 0.6637 | 0.8017 | 0.7599 | 1.0375 | 0.8128 | 0.9638 | 0.8842 | 1.4191 | 0.7673 | 0.7311 | 0.9022 | 1.0965 | 0.7612 |
| 425 | Q96M27 | Protein PRRC1 (Proline-rich and coiled-coil-containing protein 1) | PRRC1 | 23 | 4 | 1.0666 | 0.3787 | 1.1803 | 0.2432 | 0.9727 | 0.3753 | 0.6546 | 0.7837 | 0.6252 | 0.0032 | 1.9055 | 0.0782 | 0.9727 | 0.6337 | 0.6427 | 0.7194 | 0.6792 | 0.0281 | 1.2942 | 0.9707 | 0.1459 | 0.0000 | 1.8030 | 0.1483 | 0.3436 | 0.0001 | 1.0568 | 0.3774 |
| 426 | Q14558 | Phosphoribosyl pyrophosphate synthase-associated protein 1 (PRPP synthase-associated protein 1) (39 kDa phosphoribosypyrophosphate synthase-associated protein) (PAP39) | PRPSAP1 | 10 | 10 | 0.6194 | 0.3049 | 1.6444 | 0.4475 | 0.5012 | 0.4256 | 1.2589 | 0.8132 | 0.3597 | 0.2367 | 1.1066 | 0.9390 | 0.6546 | 0.6934 | 1.2474 | 0.8910 | 0.3436 | 0.0965 | 0.7244 | 0.5289 | 1.4723 | 0.3288 | 1.5704 | 0.5135 | 0.8954 | 0.9605 | 0.5970 | 0.4907 |
| 427 | P60891 | Ribose-phosphate pyrophosphokinase 1 (EC 2.7.6.1) (PPRibP) (Phosphoribosyl pyrophosphate synthase I) (PRS-I) | PRPS1 | 3 | 7 | 1.1066 | 0.7907 | 0.4325 | 0.1379 | 0.9817 | 0.9213 | 0.3631 | 0.0762 | 0.8790 | 0.2886 | 1.0280 | 0.5634 | 0.9817 | 0.6260 | 1.1376 | 0.4567 | 0.9817 | 0.8938 | 0.5346 | 0.3103 | 1.2134 | 0.2904 | 0.6792 | 0.6470 | 1.0864 | 0.9071 | 0.5702 | 0.2712 |
| 428 | O94903 | Proline synthase co-transcribed bacterial homolog protein | PROSC | 5 | 6 | 0.6668 | 0.7420 | 0.9638 | 0.7386 | 0.5598 | 0.4235 | 0.7656 | 0.5294 | 1.4859 | 0.4514 | 0.3162 | 0.0599 | 1.4997 | 0.2390 | 0.7586 | 0.3778 | 1.0965 | 0.9222 | 0.6252 | 0.2328 | 0.3664 | 0.1390 | 1.0375 | 0.9640 | 0.8318 | 0.5520 | 0.5200 | 0.5490 |
| 429 | Q9UF12 | Probable proline dehydrogenase 2 (EC 1.5.5.2) (Kidney and liver proline oxidase 1) (HsPOX1) (Probable proline oxidase 2) | PRODH2 | 4 | 4 | 0.7047 | 0.2402 | 1.0375 | 0.4246 | 0.8395 | 0.6340 | 1.2823 | 0.4577 | 1.0000 | 0.7117 | 1.4191 | 0.6376 | 0.9550 | 0.9502 | 1.2023 | 0.9458 | 0.7047 | 0.3273 | 0.7447 | 0.6193 | 0.7178 | 0.2152 | 1.3428 | 0.4054 | 0.7178 | 0.2236 | 1.4859 | 0.4651 |
| 430 | P78527 | DNA-dependent protein kinase catalytic subunit (DNA-PK catalytic subunit) (DNA-PKcs) (EC 2.7.11.1) (DNPK1) (p460) | PRKDC HYRC HYRC1 | 4 | 15 | 1.3932 | 0.5797 | 0.8017 | 0.5147 | 0.7178 | 0.3895 | 1.1912 | 0.4330 | 0.9817 | 0.9160 | 0.8790 | 0.7014 | 0.9290 | 0.8340 | 1.1169 | 0.3498 | 0.7943 | 0.5075 | 0.3733 | 0.1652 | 0.8241 | 0.4921 | 1.0864 | 0.7650 | 0.7244 | 0.7085 | 1.7061 | 0.0656 |
| 431 | P14314 | Glucosidase 2 subunit beta (80K-H protein) (Glucosidase II subunit beta) (Protein kinase C substrate 60.1 kDa protein heavy chain) (PKCSH) | PRKCSH G19P1 | 5 | 10 | 1.2023 | 0.6208 | 0.2399 | 0.0000 | 0.8091 | 0.3781 | 0.5754 | 0.0459 | 0.8790 | 0.8508 | 0.4831 | 0.0024 | 1.1695 | 0.6790 | 0.2089 | 0.0001 | 1.5849 | 0.0857 | 0.4018 | 0.0006 | 0.5248 | 0.1358 | 0.6546 | 0.0206 | 0.5346 | 0.2501 | 0.5808 | 0.0193 |
| 432 | P13861 | cAMP-dependent protein kinase type II-alpha regulatory subunit | PRKAR2A PKR2 PRKAR2 | 13 | 16 | 0.6546 | 0.3412 | 1.2706 | 0.7886 | 0.5546 | 0.3029 | 0.3467 | 0.1269 | 1.2023 | 0.3189 | 0.8954 | 0.5545 | 1.0093 | 0.7959 | 0.9204 | 0.9361 | 1.2706 | 0.4879 | 0.9550 | 0.6067 | 1.6749 | 0.0122 | 0.8630 | 0.7936 | 1.4454 | 0.0200 | 0.7178 | 0.1518 |
| 433 | P10644 | cAMP-dependent protein kinase type I-alpha regulatory subunit (Tissue-specific extinguisher 1) (TSE1) [Cleaved into: cAMP-dependent protein kinase type I-alpha regulatory subunit, N-terminally processed] | PRKAR1A PKR1 PRKAR1 TSE1 | 16 | 9 | 0.3597 | 0.0038 | 1.0280 | 0.7308 | 0.8091 | 0.5530 | 1.0864 | 0.9581 | 0.5754 | 0.0371 | 1.9231 | 0.2309 | 0.4875 | 0.0264 | 1.4060 | 0.7037 | 0.5808 | 0.0077 | 2.0701 | 0.3522 | 0.6194 | 0.0065 | 1.7539 | 0.5943 | 0.5754 | 0.0152 | 1.8535 | 0.3876 |
| 434 | P17612 | cAMP-dependent protein kinase catalytic subunit alpha (PKA C-alpha) (EC 2.7.11.11) | PRKACA PKACA | 16 | 4 | 0.5297 | 0.1525 | 0.5808 | 0.4448 | 0.6982 | 0.0297 | 0.4406 | 0.1915 | 0.7798 | 0.3820 | 1.0093 | 0.7951 | 0.7379 | 0.2066 | 0.6855 | 0.2022 | 0.6081 | 0.0560 | 0.7516 | 0.3681 | 0.6855 | 0.0705 | 0.5012 | 0.2908 | 0.5649 | 0.0378 | 0.7244 | 0.5055 |
| 435 | Q13131 | 5'-AMP-activated protein kinase catalytic subunit alpha-1 (AMPK subunit alpha-1) (EC 2.7.11.1) (Acetyl-CoA carboxylase kinase) (ACACA kinase) (EC 2.7.11.27) (Hydroxymethylglutaryl-CoA reductase kinase) (HMGCR kinase) (EC 2.7.11.31) (Tau-protein kinase PRKAA1) (EC 2.7.11.26) | PRKAA1 AMPK1 | 9 | 4 | 1.0000 | 0.7776 | 0.8790 | 0.2599 | 1.0375 | 0.7927 | 0.8790 | 0.1426 | 1.0375 | 0.6624 | 0.9550 | 0.2346 | 0.9638 | 0.4479 | 0.8551 | 0.1686 | 0.9817 | 0.8963 | 0.8872 | 0.1535 | 1.0471 | 0.6884 | 0.9550 | 0.3973 | 1.0375 | 0.8414 | 0.8710 | 0.1135 |
| 436 | P48147 | Prolyl endopeptidase (PE) (EC 3.4.21.26) (Post-proline cleaving enzyme) | PREP PEP | 3 | 4 | 0.8166 | 0.4258 | 0.4093 | 0.2293 | 0.7870 | 0.4592 | 0.4656 | 0.8118 | 0.7178 | 0.3063 | 1.6904 | 0.1096 | 0.8166 | 0.5501 | 0.7379 | 0.7421 | 0.8790 | 0.6499 | 1.2823 | 0.3055 | 0.8318 | 0.4885 | 2.0512 | 0.0267 | 0.8790 | 0.6225 | 1.6596 | 0.1753 |
| 437 | P30041 | Peroxiredoxin-6 (EC 1.11.1.15) (1-Cys peroxiredoxin) (1-Cys PRX) (24 kDa protein) (Acidic calcium-independent phospholipase A2) (aiPLA2) (EC 3.1.1.-) (Antioxidant protein 2) (Liver 2D page spot 40) (Non-selenium glutathione peroxidase) (NSGPx) (EC 1.11.1.9) (Red blood cells page spot 12) | PRDX6 AOP2 KIAA0106 | 6 | 47 | 0.6486 | 0.0659 | 1.6144 | 0.2682 | 0.7244 | 0.1079 | 1.8197 | 0.0692 | 0.8017 | 0.2552 | 1.2823 | 0.9561 | 0.6982 | 0.1415 | 1.5560 | 0.9779 | 0.7447 | 0.1312 | 1.2246 | 0.6157 | 0.4966 | 0.0146 | 1.3305 | 0.8295 | 0.6427 | 0.0355 | 0.3698 | 0.0008 |
| 438 | P30044 | Peroxiredoxin-5, mitochondrial (EC 1.11.1.15) (Alu corepressor 1) (Antioxidant enzyme B166) (AOEB166) (Liver tissue 2D-page spot 71B) (PLP) (Peroxiredoxin V) (Prx-V) (Peroxisomal antioxidant enzyme) (TPx type VI) (Thioredoxin peroxidase PMP20) (Thioredoxin reductase) | PRDX5 ACR1 SBBI10 | 5 | 22 | 0.6252 | 0.3794 | 1.4997 | 0.3000 | 0.7112 | 0.4809 | 1.9588 | 0.0335 | 1.6749 | 0.2631 | 2.2491 | 0.0070 | 0.7311 | 0.4220 | 1.3183 | 0.0413 | 1.1376 | 0.8269 | 1.2589 | 0.3630 | 1.0093 | 0.9548 | 3.1046 | 0.0007 | 1.5276 | 0.3375 | 0.8091 | 0.5082 |
| 439 | Q13162 | Peroxiredoxin-4 (EC 1.11.1.15) (Antioxidant enzyme AOE372) (AOE37-2) (Peroxiredoxin IV) (Prx-IV) (Thioredoxin peroxidase AO372) (Thioredoxin-dependent peroxide reductase A0372) | PRDX4 | 52 | 17 | 1.0765 | 0.7320 | 0.5649 | 0.3302 | 1.0965 | 0.7884 | 0.7311 | 0.2404 | 0.8872 | 0.7262 | 0.6138 | 0.1522 | 0.8166 | 0.9571 | 0.7516 | 0.2425 | 0.9290 | 0.5796 | 0.7798 | 0.2628 | 0.1107 | 0.0000 | 0.7379 | 0.1970 | 0.2148 | 0.0000 | 0.9120 | 0.5795 |
| 440 | P30048 | Thioredoxin-dependent peroxide reductase, mitochondrial (EC 1.11.1.15) (Antioxidant protein 1) (AOP-1) (HBC189) (Peroxiredoxin III) (Prx-III) (Peroxiredoxin-3) (Protein MER5 homolog) | PRDX3 AOP1 | 26 | 15 | 0.9638 | 0.5977 | 0.6607 | 0.0467 | 1.2474 | 0.5694 | 0.7379 | 0.1124 | 1.2942 | 0.3850 | 0.4018 | 0.0140 | 1.2706 | 0.1664 | 0.5970 | 0.0732 | 1.3677 | 0.3927 | 0.6081 | 0.0733 | 0.2421 | 0.0190 | 0.6982 | 0.1407 | 0.3105 | 0.0341 | 0.5248 | 0.0225 |
| 441 | P32119 | Peroxiredoxin-2 (EC 1.11.1.15) (Natural killer cell-enhancing factor B) (NKEF-B) (PRP) (Thiol-specific antioxidant protein) (TSA) (Thioredoxin peroxidase 1) (Thioredoxin-dependent peroxide reductase 1) | PRDX2 NKEFB TDPX1 | 18 | 25 | 1.0765 | 0.4081 | 0.7943 | 0.3425 | 0.7943 | 0.3351 | 0.3733 | 0.0332 | 0.9727 | 0.2230 | 0.9908 | 0.7980 | 1.8535 | 0.2516 | 1.0965 | 0.6122 | 1.3305 | 0.7264 | 0.8395 | 0.4449 | 0.9376 | 0.4999 | 0.6486 | 0.4564 | 0.6982 | 0.4319 | 0.5808 | 0.2554 |
| 442 | Q06830 | Peroxiredoxin-1 (EC 1.11.1.15) (Natural killer cell-enhancing factor A) (NKEF-A) (Proliferation-associated gene protein) (PAG) (Thioredoxin peroxidase 2) (Thioredoxin-dependent peroxide reductase 2) | PRDX1 PAGA PAGB TDPX2 | 20 | 33 | 0.6427 | 0.1790 | 0.5152 | 0.0867 | 0.5297 | 0.0339 | 0.6918 | 0.1460 | 0.4365 | 0.0118 | 0.9817 | 0.3915 | 1.0471 | 0.6893 | 0.5861 | 0.1782 | 0.5970 | 0.1543 | 0.7586 | 0.2899 | 0.3565 | 0.0103 | 0.8017 | 0.7707 | 0.4529 | 0.0398 | 0.6855 | 0.9209 |
| 443 | Q15257 | Serine/threonine-protein phosphatase 2A activator (EC 5.2.1.8) (PP2A, subunit B', PR53 isoform) (Phosphotyrosyl phosphatase activator) (PTPA) (Serine/threonine-protein phosphatase 2A regulatory subunit 4) (Serine/threonine-protein phosphatase 2A regulatory subunit B') | PPP2R4 PTPA | 26 | 4 | 0.4130 | 0.0017 | 0.9908 | 0.9928 | 0.4487 | 0.0037 | 1.0666 | 0.6398 | 0.9817 | 0.9356 | 1.1803 | 0.4332 | 0.4920 | 0.0128 | 1.1066 | 0.3928 | 0.4613 | 0.0169 | 1.0093 | 0.9914 | 0.2270 | 0.0008 | 1.0965 | 0.4527 | 0.5012 | 0.0075 | 1.0471 | 0.5572 |
| 444 | P30153 | Serine/threonine-protein phosphatase 2A 65 kDa regulatory subunit A alpha isoform (Medium tumor antigen-associated 61 kDa protein) (PP2A subunit A isoform PR65-alpha) (PP2A subunit A isoform R1-alpha) | PPP2R1A | 34 | 18 | 0.4699 | 0.0157 | 0.3404 | 0.0666 | 0.4246 | 0.0480 | 0.6730 | 0.2269 | 0.6081 | 0.2758 | 0.9204 | 0.5215 | 0.7311 | 0.3842 | 0.8630 | 0.3767 | 0.3631 | 0.0059 | 0.7244 | 0.2725 | 0.8395 | 0.2886 | 0.6607 | 0.1512 | 0.4831 | 0.0419 | 0.8091 | 0.4897 |
| 445 | Q15435 | Protein phosphatase 1 regulatory subunit 7 (Protein phosphatase 1 regulatory subunit 22) | PPP1R7 SDS22 | 4 | 9 | 1.0093 | 0.3624 | 1.1695 | 0.6201 | 0.5754 | 0.2432 | 0.8166 | 0.4474 | 1.0568 | 0.8808 | 1.1482 | 0.6158 | 0.3837 | 0.5723 | 0.8395 | 0.7787 | 1.0375 | 0.7171 | 0.9462 | 0.7892 | 0.4966 | 0.1199 | 1.0093 | 0.9739 | 0.1202 | 0.3070 | 1.0186 | 0.9253 |
| 446 | P62136 | Serine/threonine-protein phosphatase PP1-alpha catalytic subunit (PP-1A) (EC 3.1.3.16) | PPP1CA PPP1A | 21 | 10 | 0.8872 | 0.1809 | 1.6144 | 0.7105 | 0.9120 | 0.2595 | 1.6749 | 0.5655 | 1.0000 | 0.9710 | 2.3121 | 0.2277 | 0.9204 | 0.2718 | 1.3062 | 0.8127 | 0.9462 | 0.3994 | 2.9376 | 0.0527 | 0.9638 | 0.2827 | 1.1066 | 0.9096 | 0.9817 | 0.4119 | 1.9588 | 0.2980 |
| 447 | O75688 | Protein phosphatase 1B (EC 3.1.3.16) (Protein phosphatase 2C isoform beta) (PP2C-beta) | PPM1B PP2CB | 6 | 6 | 1.0568 | 0.5487 | 0.8551 | 0.7409 | 0.9908 | 0.4832 | 0.9376 | 0.9125 | 1.2134 | 0.4567 | 0.9550 | 0.9467 | 1.0280 | 0.5700 | 1.0280 | 0.9188 | 1.0280 | 0.9743 | 1.0864 | 0.8269 | 1.1376 | 0.4373 | 0.6194 | 0.4013 | 1.1376 | 0.2096 | 0.8318 | 0.7069 |
| 448 | O60437 | Periplakin (190 kDa paraneoplastic pemphigus antigen) (195 kDa cornified envelope precursor protein) | PPL KIAA0568 | 8 | 15 | 0.9462 | 0.9434 | 0.9376 | 0.4196 | 1.0965 | 0.7042 | 0.9638 | 0.7768 | 1.1272 | 0.5974 | 0.9817 | 0.6044 | 1.1376 | 0.4738 | 0.9550 | 0.6045 | 1.0375 | 0.8084 | 0.9290 | 0.3311 | 1.0765 | 0.6352 | 0.9908 | 0.8551 | 1.0568 | 0.5491 | 0.9290 | 0.3404 |
| 449 | P30405 | Peptidyl-prolyl cis-trans isomerase F, mitochondrial (PPIase F) (EC 5.2.1.8) (Cyclophilin D) (CyP-D) (CypD) (Cyclophilin F) (Mitochondrial cyclophilin) (CyP-M) (Rotamase F) | PPIF CYP3 | 4 | 7 | 0.5495 | 0.1522 | 0.7656 | 0.6140 | 1.0471 | 0.8539 | 0.5105 | 0.0898 | 0.9550 | 0.9045 | 0.8091 | 0.4454 | 0.8790 | 0.6867 | 1.1912 | 0.3666 | 0.6730 | 0.2764 | 1.2246 | 0.6552 | 0.6982 | 0.3160 | 1.1912 | 0.6715 | 1.3305 | 0.3894 | 1.4859 | 0.2125 |
| 450 | P23284 | Peptidyl-prolyl cis-trans isomerase B (PPIase B) (EC 5.2.1.8) (CYP-S1) (Cyclophilin B) (Rotamase B) (S-cyclophilin) (SCYLP) | PPIB CYPB | 11 | 22 | 1.1803 | 0.4890 | 0.7311 | 0.1882 | 1.6444 | 0.0178 | 1.0186 | 0.5075 | 1.2706 | 0.3930 | 1.0864 | 0.7843 | 1.2359 | 0.4929 | 0.4325 | 0.0090 | 0.6607 | 0.9730 | 0.9462 | 0.6404 | 0.2312 | 0.0204 | 0.7379 | 0.1543 | 0.9376 | 0.7953 | 0.8872 | 0.2446 |
| 451 | P62937 | Peptidyl-prolyl cis-trans isomerase A (PPIase A) (EC 5.2.1.8) (Cyclophilin A) (Cyclosporin A-binding protein) (Rotamase A) [Cleaved into: Peptidyl-prolyl cis-trans isomerase A, N-terminally processed] | PPIA CYPA | 8 | 33 | 0.4055 | 0.0090 | 1.1803 | 0.4870 | 0.3698 | 0.0085 | 1.2942 | 0.4731 | 1.1066 | 0.6225 | 2.2909 | 0.3489 | 0.4699 | 0.0253 | 1.8030 | 0.8878 | 0.5346 | 0.1713 | 1.9055 | 0.5833 | 0.4613 | 0.0081 | 2.0893 | 0.6009 | 0.5495 | 0.0235 | 1.1272 | 0.6202 |
| 452 | Q9HAB8 | Phosphopantothenate--cysteine ligase (EC 6.3.2.5) (Phosphopantothenoylcysteine synthetase) (PPC synthetase) | PPCS COAB | 23 | 4 | 0.4169 | 0.0681 | 1.0471 | 0.7463 | 0.9120 | 0.9153 | 0.7112 | 0.4684 | 0.8318 | 0.3230 | 0.4786 | 0.2758 | 1.4997 | 0.1140 | 0.6368 | 0.5361 | 0.9817 | 0.7163 | 1.5136 | 0.0890 | 1.4060 | 0.0755 | 0.8017 | 0.6287 | 0.7656 | 0.1558 | 1.2942 | 0.3470 |
| 453 | Q9H2U2 | Inorganic pyrophosphatase 2, mitochondrial (EC 3.6.1.1) (Pyrophosphatase SID6-306) (Pyrophosphate phospho-hydrolase 2) (PPase 2) | PPA2 HSPC124 | 42 | 10 | 0.9036 | 0.8611 | 1.3677 | 0.9741 | 1.5849 | 0.6857 | 1.9231 | 0.5383 | 2.3121 | 0.1734 | 1.2246 | 0.6854 | 2.0324 | 0.6163 | 1.5417 | 0.7594 | 2.5823 | 0.2263 | 1.7539 | 0.3962 | 2.9376 | 0.0449 | 1.2023 | 0.9251 | 0.7870 | 0.8922 | 0.8318 | 0.7119 |
| 454 | Q15181 | Inorganic pyrophosphatase (EC 3.6.1.1) (Pyrophosphate phospho-hydrolase) (PPase) | PPA1 IOPPP PP | 4 | 11 | 1.2706 | 0.5149 | 0.2168 | 0.4876 | 1.1803 | 0.6164 | 1.3305 | 0.4409 | 1.3062 | 0.4833 | 2.7290 | 0.0692 | 1.1912 | 0.6088 | 0.8091 | 0.8150 | 1.1272 | 0.6930 | 1.9770 | 0.1901 | 0.9204 | 0.8120 | 2.2080 | 0.1740 | 0.8954 | 0.7833 | 3.0479 | 0.0579 |
| 455 | Q15063 | Periostin (PN) (Osteoblast-specific factor 2) (OSF-2) | POSTN OSF2 | 9 | 4 | 1.9770 | 0.7305 | 0.1888 | 0.0845 | 2.4660 | 0.9375 | 0.2168 | 0.1993 | 2.1281 | 0.9644 | 0.6026 | 0.4839 | 4.1305 | 0.1592 | 0.7311 | 0.9540 | 3.0479 | 0.5454 | 0.1355 | 0.0810 | 0.8710 | 0.1620 | 0.7447 | 0.3827 | 0.5495 | 0.1651 | 0.0360 | 0.0315 |
| 456 | P16435 | NADPH--cytochrome P450 reductase (CPR) (P450R) (EC 1.6.2.4) | POR CYPOR | 9 | 39 | 1.3428 | 0.7435 | 1.9770 | 0.2038 | 2.1677 | 0.6690 | 2.0324 | 0.2624 | 1.7865 | 0.6160 | 2.5823 | 0.0258 | 2.2491 | 0.4953 | 1.1912 | 0.9021 | 1.2706 | 0.8365 | 1.5276 | 0.7925 | 2.5119 | 0.1922 | 2.0701 | 0.1675 | 2.3988 | 0.2576 | 2.3550 | 0.0818 |
| 457 | Q15166 | Serum paraoxonase/lactonase 3 (EC 3.1.1.2) (EC 3.1.1.81) (EC 3.1.8.1) | PON3 | 5 | 17 | 2.8576 | 0.0606 | 0.9290 | 0.9384 | 1.6444 | 0.3765 | 1.3062 | 0.0938 | 2.7040 | 0.1211 | 0.4966 | 0.0321 | 0.6486 | 0.2817 | 0.4831 | 0.0945 | 1.3428 | 0.8280 | 0.2704 | 0.0033 | 0.6252 | 0.2972 | 0.8241 | 0.2312 | 0.5808 | 0.1850 | 0.1271 | 0.0007 |
| 458 | Q15165 | Serum paraoxonase/arylesterase 2 (PON 2) (EC 3.1.1.2) (EC 3.1.1.81) (Aromatic esterase 2) (A-esterase 2) (Serum aryldialkylphosphatase 2) | PON2 | 43 | 12 | 2.3550 | 0.1879 | 1.5996 | 0.3373 | 1.9231 | 0.0430 | 1.8880 | 0.1810 | 1.5136 | 0.9677 | 2.4660 | 0.1313 | 3.4356 | 0.0001 | 0.6982 | 0.9620 | 1.3428 | 0.7343 | 0.8790 | 0.9732 | 1.9770 | 0.2295 | 1.8880 | 0.2613 | 1.4859 | 0.9123 | 1.5996 | 0.2239 |
| 459 | P27169 | Serum paraoxonase/arylesterase 1 (PON 1) (EC 3.1.1.2) (EC 3.1.1.81) (EC 3.1.8.1) (Aromatic esterase 1) (A-esterase 1) (K-45) (Serum aryldialkylphosphatase 1) | PON1 PON | 18 | 21 | 0.9290 | 0.9711 | 0.9120 | 0.9259 | 0.7112 | 0.3440 | 0.5546 | 0.3000 | 1.0864 | 0.4113 | 0.6668 | 0.0755 | 1.0375 | 0.8759 | 1.4322 | 0.1562 | 1.1376 | 0.1990 | 0.4055 | 0.0209 | 0.0679 | 0.0000 | 1.3428 | 0.2023 | 0.2228 | 0.0015 | 0.2249 | 0.0471 |
| 460 | Q9NVS9 | Pyridoxine-5'-phosphate oxidase (EC 1.4.3.5) (Pyridoxamine-phosphate oxidase) | PNPO | 9 | 11 | 0.9290 | 0.9692 | 1.3552 | 0.3108 | 0.8954 | 0.7618 | 1.7219 | 0.3108 | 1.0000 | 0.8340 | 2.0324 | 0.4515 | 0.9376 | 0.9297 | 1.2942 | 0.5133 | 0.8954 | 0.8308 | 1.6749 | 0.6984 | 0.7311 | 0.1448 | 2.6792 | 0.0101 | 0.8954 | 0.5213 | 0.5297 | 0.1223 |
| 461 | P00491 | Purine nucleoside phosphorylase (PNP) (EC 2.4.2.1) (Inosine phosphorylase) (Inosine-guanosine phosphorylase) | PNP NP | 21 | 17 | 1.2942 | 0.1807 | 1.4997 | 0.1327 | 0.8630 | 0.6446 | 1.2942 | 0.3481 | 1.3932 | 0.1867 | 1.4588 | 0.7157 | 1.3428 | 0.2766 | 2.1281 | 0.1013 | 0.6138 | 0.0074 | 1.8535 | 0.1378 | 0.1528 | 0.1654 | 1.5276 | 0.1038 | 0.2938 | 0.0861 | 0.3342 | 0.1760 |
| 462 | Q15126 | Phosphomevalonate kinase (PMKase) (hPMK) (EC 2.7.4.2) | PMVK PMKI | 11 | 5 | 0.5297 | 0.1616 | 5.4450 | 0.1176 | 1.0000 | 0.8246 | 6.5464 | 0.0367 | 0.5754 | 0.4300 | 6.3096 | 0.0513 | 1.0568 | 0.8265 | 7.7983 | 0.0073 | 0.7178 | 0.6200 | 3.0200 | 0.1299 | 0.6081 | 0.4365 | 5.9704 | 0.1436 | 0.4093 | 0.0536 | 3.2211 | 0.4574 |
| 463 | O75439 | Mitochondrial-processing peptidase subunit beta (EC 3.4.24.64) (Beta-MPP) (P-52) | PMPCB MPPB | 13 | 10 | 1.3062 | 0.4131 | 1.8880 | 0.5402 | 1.8365 | 0.3085 | 1.8197 | 0.4094 | 1.8365 | 0.3841 | 1.1482 | 0.9779 | 1.1272 | 0.8649 | 1.4997 | 0.4732 | 1.5417 | 0.6223 | 1.0375 | 0.7534 | 0.2606 | 0.2545 | 2.0512 | 0.2139 | 0.3192 | 0.3307 | 1.9588 | 0.2970 |
| 464 | Q10713 | Mitochondrial-processing peptidase subunit alpha (EC 3.4.24.64) (Alpha-MPP) (P-55) | PMPCA INPP5E KIAA0123 MPPA | 6 | 7 | 1.0186 | 0.8010 | 1.4322 | 0.8810 | 1.0375 | 0.9630 | 0.9120 | 0.9623 | 1.0864 | 0.5145 | 2.9107 | 0.9944 | 1.0864 | 0.6890 | 1.9409 | 0.2875 | 0.9908 | 0.6376 | 1.3552 | 0.7839 | 0.9462 | 0.8264 | 1.4454 | 0.8141 | 0.9204 | 0.7098 | 4.0551 | 0.1766 |
| 465 | O15031 | Plexin-B2 (MM1) | PLXNB2 KIAA0315 | 6 | 3 | 1.6904 | 0.2320 | 0.7943 | 0.7570 | 1.1169 | 0.9523 | 1.0965 | 0.7018 | 1.5276 | 0.1720 | 0.6918 | 0.5088 | 1.4723 | 0.2289 | 0.7943 | 0.6733 | 1.8030 | 0.1184 | 0.9290 | 0.8921 | 0.6486 | 0.8486 | 0.7244 | 0.4812 | 0.9817 | 0.7706 | 1.1169 | 0.7473 |
| 466 | P13797 | Plastin-3 (T-plastin) | PLS3 | 7 | 19 | 0.6486 | 0.5541 | 0.7178 | 0.4818 | 0.3436 | 0.1254 | 0.9908 | 0.7248 | 0.4055 | 0.4218 | 0.8630 | 0.9859 | 0.8318 | 0.5898 | 1.0093 | 0.9521 | 0.7943 | 0.8129 | 1.1803 | 0.8430 | 0.6546 | 0.4347 | 1.4588 | 0.2312 | 0.3981 | 0.6599 | 0.7112 | 0.5400 |
| 467 | Q14651 | Plastin-1 (Intestine-specific plastin) (I-plastin) | PLS1 | 3 | 11 | 0.6668 | 0.9859 | 0.5702 | 0.7520 | 1.1272 | 0.8566 | 1.5276 | 0.1200 | 0.1629 | 0.0591 | 0.4285 | 0.2690 | 1.0568 | 0.8948 | 1.1588 | 0.5890 | 0.9290 | 0.9072 | 0.5649 | 0.5636 | 1.5704 | 0.5737 | 1.9953 | 0.0731 | 1.1169 | 0.9182 | 0.3837 | 0.6416 |
| 468 | Q96Q06 | Perilipin-4 (Adipocyte protein S3-12) | PLIN4 KIAA1881 | 27 | 8 | 0.8551 | 0.6565 | 1.4454 | 0.5302 | 0.7047 | 0.4462 | 0.5916 | 0.3092 | 1.1695 | 0.5962 | 3.5318 | 0.2170 | 1.2474 | 0.9617 | 5.8076 | 0.0390 | 1.3804 | 0.8038 | 4.4875 | 0.1774 | 1.6293 | 0.3799 | 2.4889 | 0.6578 | 0.6310 | 0.3045 | 3.4674 | 0.2939 |
| 469 | O60664 | Perilipin-3 (47 kDa mannose 6-phosphate receptor-binding protein) (47 kDa MPR-binding protein) (Cargo selection protein TIP47) (Mannose-6-phosphate receptor-binding protein 1) (Placental protein 17) (PP17) | PLIN3 M6PRBP1 TIP47 | 8 | 12 | 2.1478 | 0.2382 | 2.7040 | 0.3657 | 1.6444 | 0.3293 | 1.0186 | 0.8623 | 1.6293 | 0.3392 | 4.4463 | 0.1370 | 1.3932 | 0.4334 | 2.8576 | 0.3718 | 2.0324 | 0.2545 | 3.1623 | 0.3329 | 1.3062 | 0.4886 | 3.9084 | 0.1916 | 1.2474 | 0.5731 | 3.5975 | 0.1200 |
| 470 | P00747 | Plasminogen (EC 3.4.21.7) [Cleaved into: Plasmin heavy chain A; Activation peptide; Angiostatin; Plasmin heavy chain A, short form; Plasmin light chain B] | PLG | 8 | 10 | 1.7701 | 0.2313 | 0.5445 | 0.0968 | 2.6303 | 0.0820 | 0.6730 | 0.1707 | 3.2509 | 0.0441 | 0.5248 | 0.0738 | 3.1915 | 0.0015 | 0.5649 | 0.0570 | 0.2754 | 0.3166 | 0.9036 | 0.6227 | 3.4995 | 0.0107 | 0.9638 | 0.6878 | 2.9107 | 0.1037 | 1.1272 | 0.8547 |
| 471 | Q15149 | Plectin (PCN) (PLTN) (Hemidesmosomal protein 1) (HD1) (Plectin-1) | PLEC PLEC1 | 6 | 122 | 1.0864 | 0.9811 | 0.9908 | 0.5609 | 1.8535 | 0.2357 | 0.9908 | 0.7108 | 1.2474 | 0.8184 | 1.5276 | 0.0185 | 0.9120 | 0.7781 | 1.0093 | 0.7361 | 1.3804 | 0.3470 | 1.5136 | 0.0221 | 2.1878 | 0.0131 | 1.2942 | 0.6900 | 2.1878 | 0.0535 | 1.5849 | 0.0160 |
| 472 | Q8IV08 | Phospholipase D3 (PLD 3) (EC 3.1.4.4) (Choline phosphatase 3) (HindIII K4L homolog) (Hu-K4) (Phosphatidylcholine-hydrolyzing phospholipase D3) | PLD3 | 10 | 3 | 1.2134 | 0.4865 | 1.1588 | 0.2085 | 1.7378 | 0.3487 | 0.6668 | 0.6771 | 1.3677 | 0.3470 | 1.6904 | 0.1467 | 0.5346 | 0.0737 | 0.4831 | 0.2244 | 0.4920 | 0.0180 | 0.7178 | 0.7330 | 0.5200 | 0.0315 | 0.8017 | 0.8852 | 1.1803 | 0.4934 | 1.1912 | 0.4834 |
| 473 | Q99959 | Plakophilin-2 | PKP2 | 116 | 5 | 0.7178 | 0.0457 | 0.2965 | 0.0630 | 1.1695 | 0.4224 | 1.0000 | 0.9130 | 1.4454 | 0.0195 | 0.6486 | 0.6085 | 0.9908 | 0.9564 | 0.8241 | 0.9503 | 1.2474 | 0.1906 | 0.9817 | 0.9038 | 1.0375 | 0.3684 | 0.7447 | 0.5161 | 1.1695 | 0.2477 | 0.5105 | 0.3666 |
| 474 | P14618 | Pyruvate kinase PKM (EC 2.7.1.40) (Cytosolic thyroid hormone-binding protein) (CTHBP) (Opa-interacting protein 3) (OIP-3) (Pyruvate kinase 2/3) (Pyruvate kinase muscle isozyme) (Thyroid hormone-binding protein 1) (THBP1) (Tumor M2-PK) (p58) | PKM OIP3 PK2 PK3 PKM2 | 5 | 25 | 0.4529 | 0.6873 | 2.3550 | 0.1749 | 0.6730 | 0.7565 | 2.3121 | 0.1918 | 0.6194 | 0.9820 | 4.0551 | 0.0008 | 0.7943 | 0.8770 | 1.9588 | 0.7717 | 1.0666 | 0.8896 | 3.6308 | 0.0133 | 2.4660 | 0.0181 | 1.7061 | 0.6251 | 2.2080 | 0.0494 | 4.6559 | 0.0000 |
| 475 | P30613 | Pyruvate kinase PKLR (EC 2.7.1.40) (Pyruvate kinase 1) (Pyruvate kinase isozymes L/R) (R-type/L-type pyruvate kinase) (Red cell/liver pyruvate kinase) | PKLR PK1 PKL | 5 | 43 | 0.9638 | 0.8500 | 1.1272 | 0.6311 | 0.9036 | 0.7515 | 1.7219 | 0.3018 | 0.7727 | 0.6360 | 1.6293 | 0.6690 | 0.8091 | 0.4128 | 4.2462 | 0.0001 | 0.8395 | 0.4946 | 2.3550 | 0.2517 | 0.7727 | 0.3445 | 4.2855 | 0.0011 | 0.8318 | 0.3447 | 1.6144 | 0.8911 |
| 476 | Q9P0Z9 | Peroxisomal sarcosine oxidase (PSO) (EC 1.5.3.1) (EC 1.5.3.7) (L-pipecolate oxidase) (L-pipecolic acid oxidase) | PIPOX LPIPOX PSO | 25 | 21 | 0.7516 | 0.6716 | 0.8954 | 0.6760 | 2.7542 | 0.0630 | 1.3183 | 0.0656 | 1.8365 | 0.5495 | 1.0765 | 0.2871 | 1.9770 | 0.4376 | 1.5849 | 0.0030 | 1.8197 | 0.2651 | 1.0186 | 0.6275 | 4.6132 | 0.0000 | 0.7447 | 0.8521 | 3.0761 | 0.0022 | 1.1376 | 0.3544 |
| 477 | P48426 | Phosphatidylinositol 5-phosphate 4-kinase type-2 alpha (EC 2.7.1.149) (1-phosphatidylinositol 5-phosphate 4-kinase 2-alpha) (Diphosphoinositide kinase 2-alpha) (PIP5KIII) (Phosphatidylinositol 5-phosphate 4-kinase type II alpha) (PI(5)P 4-kinase type II alpha) (PIP4KII-alpha) (PtdIns(4)P-5-kinase B isoform) (PtdIns(4)P-5-kinase C isoform) (PtdIns(5)P-4-kinase isoform 2-alpha) | PIP4K2A PIP5K2 PIP5K2A | 40 | 4 | 1.2474 | 0.6693 | 1.1482 | 0.5808 | 0.9817 | 0.9316 | 1.1803 | 0.9609 | 2.0512 | 0.0171 | 1.8535 | 0.9914 | 0.8710 | 0.3400 | 0.5495 | 0.1884 | 0.6792 | 0.0139 | 3.8726 | 0.1426 | 0.0895 | 0.0000 | 1.4454 | 0.7128 | 0.2831 | 0.0003 | 2.6546 | 0.3885 |
| 478 | Q6ZUJ8 | Phosphoinositide 3-kinase adapter protein 1 (B-cell adapter for phosphoinositide 3-kinase) (B-cell phosphoinositide 3-kinase adapter protein 1) | PIK3AP1 BCAP | 19 | 4 | 1.5704 | 0.7317 | 0.8630 | 0.6888 | 1.8535 | 0.0598 | 1.1588 | 0.6982 | 1.3183 | 0.6996 | 0.4613 | 0.1901 | 1.2246 | 0.7446 | 0.6194 | 0.4285 | 2.6062 | 0.0022 | 1.2474 | 0.2958 | 0.6546 | 0.0072 | 0.6855 | 0.5038 | 2.0324 | 0.1279 | 0.2911 | 0.2392 |
| 479 | Q13492 | Phosphatidylinositol-binding clathrin assembly protein (Clathrin assembly lymphoid myeloid leukemia protein) | PICALM CALM | 3 | 4 | 0.2858 | 0.3909 | 1.7539 | 0.3544 | 1.6293 | 0.2643 | 1.7539 | 0.6344 | 1.5560 | 0.2880 | 3.0479 | 0.0523 | 1.2474 | 0.3799 | 2.2699 | 0.3460 | 0.3404 | 0.4842 | 2.7542 | 0.2551 | 1.7865 | 0.2344 | 1.1066 | 0.9749 | 2.4660 | 0.1833 | 1.5276 | 0.6353 |
| 480 | Q5SRE7 | Phytanoyl-CoA dioxygenase domain-containing protein 1 (EC 1.-.-.-) | PHYHD1 | 5 | 5 | 0.7178 | 0.8066 | 1.0093 | 0.9528 | 0.5248 | 0.5163 | 0.4487 | 0.1009 | 0.1905 | 0.0120 | 1.0666 | 0.8006 | 0.5495 | 0.3410 | 0.5649 | 0.1840 | 0.5346 | 0.1771 | 1.3183 | 0.3997 | 0.5248 | 0.1454 | 0.7311 | 0.3888 | 0.7447 | 0.3680 | 1.2023 | 0.5460 |
| 481 | O14832 | Phytanoyl-CoA dioxygenase, peroxisomal (EC 1.14.11.18) (Phytanic acid oxidase) (Phytanoyl-CoA alpha-hydroxylase) (PhyH) | PHYH PAHX | 7 | 5 | 1.4588 | 0.3933 | 0.3467 | 0.4142 | 1.2134 | 0.5874 | 0.4571 | 0.1124 | 1.6904 | 0.3097 | 0.4130 | 0.0300 | 1.4997 | 0.3719 | 0.9908 | 0.9040 | 1.5136 | 0.3673 | 1.1695 | 0.6170 | 1.5996 | 0.3332 | 1.5560 | 0.0764 | 1.7061 | 0.3057 | 1.0965 | 0.5759 |
| 482 | Q93100 | Phosphorylase b kinase regulatory subunit beta (Phosphorylase kinase subunit beta) | PHKB | 4 | 7 | 2.9107 | 0.3362 | 0.6310 | 0.1488 | 2.1086 | 0.5148 | 1.0568 | 0.8152 | 0.8017 | 0.9230 | 1.0375 | 0.8009 | 2.2699 | 0.5107 | 1.0093 | 0.9831 | 2.0701 | 0.5306 | 0.8872 | 0.7855 | 0.8472 | 0.8261 | 0.6607 | 0.2042 | 0.9376 | 0.9253 | 0.5297 | 0.1107 |
| 483 | O43175 | D-3-phosphoglycerate dehydrogenase (3-PGDH) (EC 1.1.1.95) | PHGDH PGDH3 | 7 | 34 | 0.7870 | 0.5445 | 1.9770 | 0.4016 | 1.7865 | 0.2645 | 1.9588 | 0.1067 | 1.0471 | 0.9527 | 1.7865 | 0.2608 | 0.5346 | 0.2130 | 2.3988 | 0.1778 | 0.9376 | 0.6690 | 1.0965 | 0.8061 | 0.3733 | 0.1334 | 1.8365 | 0.4716 | 0.9376 | 0.9167 | 0.6607 | 0.0212 |
| 484 | Q99623 | Prohibitin-2 (B-cell receptor-associated protein BAP37) (D-prohibitin) (Repressor of estrogen receptor activity) | PHB2 BAP REA | 8 | 27 | 1.1695 | 0.7950 | 0.9638 | 0.2322 | 0.7379 | 0.9290 | 0.9638 | 0.4034 | 0.5808 | 0.3231 | 1.0186 | 0.6473 | 1.1376 | 0.9800 | 1.0280 | 0.7527 | 1.0000 | 0.9247 | 1.0000 | 0.6616 | 0.5297 | 0.3150 | 0.9908 | 0.8190 | 0.7244 | 0.5525 | 0.9817 | 0.8997 |
| 485 | P35232 | Prohibitin | PHB | 32 | 33 | 1.1803 | 0.7799 | 0.8472 | 0.7229 | 1.5136 | 0.7515 | 0.8710 | 0.7453 | 1.1912 | 0.9750 | 1.4322 | 0.0811 | 1.2246 | 0.9334 | 1.5849 | 0.0178 | 1.5136 | 0.4735 | 1.4060 | 0.2652 | 0.2992 | 0.0004 | 1.5136 | 0.0417 | 0.5808 | 0.0051 | 1.1912 | 0.0566 |
| 486 | O15173 | Membrane-associated progesterone receptor component 2 (Progesterone membrane-binding protein) (Steroid receptor protein DG6) | PGRMC2 DG6 PMBP | 30 | 7 | 0.9550 | 0.7251 | 0.5754 | 0.7174 | 0.8872 | 0.6530 | 1.1376 | 0.2236 | 0.9376 | 0.6679 | 0.9290 | 0.8397 | 1.4454 | 0.0579 | 0.6546 | 0.9183 | 1.2589 | 0.1419 | 0.3698 | 0.5405 | 0.3802 | 0.0004 | 1.0471 | 0.7000 | 0.9462 | 0.5648 | 0.4571 | 0.2908 |
| 487 | O00264 | Membrane-associated progesterone receptor component 1 (mPR) | PGRMC1 HPR6.6 PGRMC | 36 | 12 | 1.4997 | 0.1504 | 0.5248 | 0.0331 | 1.0568 | 0.9988 | 1.0375 | 0.8954 | 1.2706 | 0.4500 | 0.5495 | 0.2378 | 1.1066 | 0.2989 | 0.6982 | 0.1392 | 1.2706 | 0.3611 | 0.6982 | 0.0607 | 0.4831 | 0.0104 | 1.2823 | 0.3523 | 0.6427 | 0.5976 | 0.5702 | 0.0515 |
| 488 | O95394 | Phosphoacetylglucosamine mutase (PAGM) (EC 5.4.2.3) (Acetylglucosamine phosphomutase) (N-acetylglucosamine-phosphate mutase) (Phosphoglucomutase-3) (PGM 3) | PGM3 AGM1 | 6 | 4 | 1.3552 | 0.5981 | 1.0666 | 0.5728 | 0.6792 | 0.7837 | 1.0765 | 0.8161 | 1.4997 | 0.3869 | 1.2134 | 0.4214 | 0.9036 | 0.9106 | 1.2134 | 0.1610 | 0.9817 | 0.9100 | 1.1376 | 0.3830 | 0.5649 | 0.4115 | 1.1066 | 0.4286 | 0.6486 | 0.5102 | 1.0186 | 0.6173 |
| 489 | Q96G03 | Phosphoglucomutase-2 (PGM 2) (EC 5.4.2.2) (Glucose phosphomutase 2) (Phosphodeoxyribomutase) (Phosphopentomutase) (EC 5.4.2.7) | PGM2 MSTP006 | 14 | 12 | 1.1912 | 0.9337 | 1.0765 | 0.6246 | 1.4060 | 0.9629 | 0.8472 | 0.3621 | 0.6081 | 0.1083 | 0.8318 | 0.5668 | 1.0568 | 0.8101 | 1.8707 | 0.6321 | 1.2359 | 0.6695 | 0.7656 | 0.3688 | 0.1213 | 0.0197 | 1.2474 | 0.4259 | 0.5152 | 0.1446 | 0.3873 | 0.1591 |
| 490 | P36871 | Phosphoglucomutase-1 (PGM 1) (EC 5.4.2.2) (Glucose phosphomutase 1) | PGM1 | 8 | 90 | 1.4060 | 0.8712 | 1.2706 | 0.8931 | 1.5560 | 0.7176 | 1.7219 | 0.8131 | 0.8710 | 0.8821 | 0.9290 | 0.0272 | 2.1677 | 0.1402 | 1.5276 | 0.8638 | 0.4325 | 0.3078 | 1.4723 | 0.6548 | 1.3183 | 0.8173 | 2.2491 | 0.0241 | 2.3988 | 0.1980 | 0.2858 | 0.0000 |
| 491 | Q96PD5 | N-acetylmuramoyl-L-alanine amidase (EC 3.5.1.28) (Peptidoglycan recognition protein 2) (Peptidoglycan recognition protein long) (PGRP-L) | PGLYRP2 PGLYRPL PGRPL UNQ3103/PRO10102 | 10 | 4 | 1.0000 | 0.9087 | 0.9908 | 0.8738 | 1.1066 | 0.5670 | 1.0765 | 0.8570 | 1.2134 | 0.2236 | 1.0765 | 0.6580 | 0.7727 | 0.1863 | 1.0375 | 0.8102 | 1.0568 | 0.4736 | 0.8241 | 0.7059 | 0.7727 | 0.2840 | 0.8872 | 0.9642 | 0.6668 | 0.2090 | 1.1272 | 0.6290 |
| 492 | O95336 | 6-phosphogluconolactonase (6PGL) (EC 3.1.1.31) | PGLS | 83 | 14 | 1.0765 | 0.1541 | 0.9376 | 0.5786 | 1.1272 | 0.0024 | 1.3062 | 0.6729 | 0.8472 | 0.0056 | 1.2706 | 0.3259 | 1.5560 | 0.8380 | 0.7727 | 0.7856 | 1.2474 | 0.1156 | 1.1803 | 0.9709 | 0.0973 | 0.0000 | 1.5849 | 0.2126 | 0.1330 | 0.0000 | 0.5754 | 0.8274 |
| 493 | P00558 | Phosphoglycerate kinase 1 (EC 2.7.2.3) (Cell migration-inducing gene 10 protein) (Primer recognition protein 2) (PRP 2) | PGK1 PGKA MIG10 OK/SW-cl.110 | 3 | 48 |  |  | 0.3837 | 0.0076 |  |  | 1.0666 | 0.8683 |  |  | 1.1695 | 0.7127 |  |  | 0.8710 | 0.1675 |  |  | 1.0186 | 0.9598 |  |  | 1.1482 | 0.7622 |  |  | 0.8790 | 0.4944 |
| 494 | P52209 | 6-phosphogluconate dehydrogenase, decarboxylating (EC 1.1.1.44) | PGD PGDH | 18 | 20 | 1.8197 | 0.2226 | 0.8017 | 0.6761 | 1.3932 | 0.6565 | 0.4613 | 0.0623 | 2.4210 | 0.0452 | 1.4997 | 0.0690 | 2.4889 | 0.0340 | 1.3552 | 0.0516 | 1.8030 | 0.2903 | 1.4588 | 0.0595 | 1.1376 | 0.7904 | 1.6293 | 0.0408 | 1.0000 | 0.4047 | 1.0568 | 0.8855 |
| 495 | Q96HS1 | Serine/threonine-protein phosphatase PGAM5, mitochondrial (EC 3.1.3.16) (Bcl-XL-binding protein v68) (Phosphoglycerate mutase family member 5) | PGAM5 | 52 | 4 | 0.4406 | 0.0002 | 1.3552 | 0.8146 | 0.9204 | 0.7804 | 0.8166 | 0.6494 | 0.9638 | 0.8465 | 0.9727 | 0.8310 | 1.1588 | 0.1658 | 1.0093 | 0.8250 | 0.8472 | 0.2432 | 1.0765 | 0.8197 | 0.8954 | 0.2814 | 1.7219 | 0.5004 | 0.5152 | 0.0005 | 0.4285 | 0.5036 |
| 496 | P18669 | Phosphoglycerate mutase 1 (EC 3.1.3.13) (EC 5.4.2.11) (EC 5.4.2.4) (BPG-dependent PGAM 1) (Phosphoglycerate mutase isozyme B) (PGAM-B) | PGAM1 PGAMA CDABP0006 | 25 | 26 | 0.5808 | 0.3848 | 0.6310 | 0.7060 | 2.7797 | 0.0692 | 0.7943 | 0.8355 | 2.5586 | 0.0763 | 1.6293 | 0.1282 | 2.1677 | 0.1326 | 0.6194 | 0.5852 | 1.0864 | 0.9456 | 1.4588 | 0.2280 | 0.9638 | 0.9774 | 1.7701 | 0.1606 | 1.8880 | 0.2246 | 1.4191 | 0.3361 |
| 497 | P07737 | Profilin-1 (Epididymis tissue protein Li 184a) (Profilin I) | PFN1 | 3 | 22 | 1.5996 | 0.4902 | 1.6596 | 0.4689 | 1.1169 | 0.3304 | 1.0375 | 0.6697 | 0.9817 | 0.6859 | 2.6546 | 0.1310 | 1.1912 | 0.6585 | 1.6144 | 0.7421 | 1.6144 | 0.1430 | 2.5823 | 0.0975 | 1.2823 | 0.3636 | 3.0200 | 0.0170 | 1.0666 | 0.8194 | 2.3768 | 0.0402 |
| 498 | P17858 | ATP-dependent 6-phosphofructokinase, liver type (ATP-PFK) (PFK-L) (EC 2.7.1.11) (6-phosphofructokinase type B) (Phosphofructo-1-kinase isozyme B) (PFK-B) (Phosphohexokinase) | PFKL | 28 | 29 | 0.4875 | 0.0614 | 0.6310 | 0.0807 | 1.2134 | 0.7822 | 0.9550 | 0.8963 | 1.2023 | 0.7278 | 0.7311 | 0.0988 | 1.0666 | 0.6591 | 0.6730 | 0.1838 | 0.8166 | 0.1897 | 1.1376 | 0.5816 | 0.6427 | 0.0074 | 0.8318 | 0.1593 | 0.4699 | 0.0207 | 1.0965 | 0.9433 |
| 499 | P12955 | Xaa-Pro dipeptidase (X-Pro dipeptidase) (EC 3.4.13.9) (Imidodipeptidase) (Peptidase D) (Proline dipeptidase) (Prolidase) | PEPD PRD | 34 | 13 | 0.7798 | 0.2773 | 0.7943 | 0.6824 | 1.4191 | 0.8983 | 1.1376 | 0.3577 | 1.8030 | 0.2157 | 1.3932 | 0.3843 | 1.4997 | 0.2665 | 1.3552 | 0.6549 | 1.0666 | 0.8921 | 1.9409 | 0.0337 | 1.7539 | 0.1284 | 1.3804 | 0.4248 | 1.6904 | 0.2425 | 1.6749 | 0.0477 |
| 500 | Q9BY49 | Peroxisomal trans-2-enoyl-CoA reductase (TERP) (EC 1.3.1.38) (2,4-dienoyl-CoA reductase-related protein) (DCR-RP) (HPDHase) (Short chain dehydrogenase/reductase family 29C member 1) (pVI-ARL) | PECR SDR29C1 PRO1004 | 26 | 17 | 1.0000 | 0.7857 | 1.2359 | 0.3276 | 0.8017 | 0.6688 | 0.9550 | 0.8598 | 0.7047 | 0.2578 | 1.6293 | 0.0820 | 0.6855 | 0.2583 | 2.3335 | 0.0125 | 0.7379 | 0.2549 | 0.8318 | 0.6734 | 0.5598 | 0.0141 | 0.5346 | 0.0964 | 0.9204 | 0.9302 | 1.1482 | 0.4640 |
| 501 | P30086 | Phosphatidylethanolamine-binding protein 1 (PEBP-1) (HCNPpp) (Neuropolypeptide h3) (Prostatic-binding protein) (Raf kinase inhibitor protein) (RKIP) [Cleaved into: Hippocampal cholinergic neurostimulating peptide (HCNP)] | PEBP1 PBP PEBP | 13 | 59 | 2.1677 | 0.1823 | 0.8872 | 0.1157 | 1.7539 | 0.7252 | 1.2246 | 0.9171 | 2.7290 | 0.0564 | 1.3062 | 0.6131 | 2.5823 | 0.0447 | 0.5297 | 0.0041 | 1.0864 | 0.8809 | 0.9376 | 0.0880 | 1.2589 | 0.3589 | 2.0512 | 0.0958 | 4.2855 | 0.0002 | 0.2780 | 0.0027 |
| 502 | Q5T2W1 | Na(+)/H(+) exchange regulatory cofactor NHE-RF3 (NHERF-3) (CFTR-associated protein of 70 kDa) (Na(+)/H(+) exchanger regulatory factor 3) (Na/Pi cotransporter C-terminal-associated protein 1) (NaPi-Cap1) (PDZ domain-containing protein 1) (Sodium-hydrogen exchanger regulatory factor 3) | PDZK1 CAP70 NHERF3 PDZD1 | 24 | 8 | 1.1376 | 0.3115 | 1.2359 | 0.6082 | 0.9462 | 0.1526 | 1.1695 | 0.3505 | 1.7219 | 0.0927 | 1.6904 | 0.0964 | 1.3932 | 0.5937 | 1.0375 | 0.8322 | 2.2284 | 0.0203 | 1.4454 | 0.2232 | 0.2559 | 0.0000 | 1.0471 | 0.5584 | 0.5248 | 0.0004 | 1.2359 | 0.3080 |
| 503 | Q96GD0 | Pyridoxal phosphate phosphatase (PLP phosphatase) (EC 3.1.3.3) (EC 3.1.3.74) (Chronophin) | PDXP CIN PLP PLPP | 70 | 4 | 1.0471 | 0.5465 | 1.0093 | 0.5568 | 1.0765 | 0.8382 | 0.4325 | 0.7397 | 1.3183 | 0.0989 | 1.4060 | 0.8535 | 0.9817 | 0.3263 | 0.5916 | 0.4860 | 1.0000 | 0.9452 | 2.3121 | 0.0935 | 0.0912 | 0.0000 | 1.8030 | 0.1728 | 0.1542 | 0.0005 | 0.5495 | 0.6385 |
| 504 | O00764 | Pyridoxal kinase (EC 2.7.1.35) (Pyridoxine kinase) | PDXK C21orf124 C21orf97 PKH PNK PRED79 | 4 | 7 | 1.3932 | 0.5992 | 0.7870 | 0.7496 | 2.1086 | 0.1383 | 0.8472 | 0.7502 | 1.7219 | 0.1978 | 0.5297 | 0.2757 | 0.6792 | 0.5159 | 1.0568 | 0.9726 | 1.7219 | 0.1702 | 0.8954 | 0.7733 | 1.0471 | 0.6684 | 1.0093 | 0.8360 | 1.5560 | 0.3152 | 1.1376 | 0.7801 |
| 505 | Q6P996 | Pyridoxal-dependent decarboxylase domain-containing protein 1 (EC 4.1.1.-) | PDXDC1 KIAA0251 | 4 | 4 | 1.0375 | 0.7700 | 2.7290 | 0.2136 | 0.7586 | 0.3524 | 2.2699 | 0.4507 | 0.9727 | 0.8695 | 2.1677 | 0.2637 | 0.9908 | 0.4784 | 4.2073 | 0.0138 | 0.9908 | 0.4980 | 2.9923 | 0.1355 | 0.3404 | 0.0966 | 2.3335 | 0.1400 | 0.9908 | 0.6309 | 2.8840 | 0.1475 |
| 506 | Q96HC4 | PDZ and LIM domain protein 5 (Enigma homolog) (Enigma-like PDZ and LIM domains protein) | PDLIM5 ENH L9 | 4 | 10 | 0.9462 | 0.6910 | 0.9908 | 0.9547 | 0.7870 | 0.7326 | 1.0568 | 0.5996 | 0.8630 | 0.9726 | 1.1376 | 0.2462 | 0.9462 | 0.7839 | 0.9908 | 0.7310 | 0.8318 | 0.9313 | 1.0864 | 0.3747 | 0.8395 | 0.9687 | 1.0000 | 0.8969 | 0.8241 | 0.9817 | 1.0186 | 0.8048 |
| 507 | Q96JY6 | PDZ and LIM domain protein 2 (PDZ-LIM protein mystique) | PDLIM2 PP6345 | 4 | 5 | 0.4699 | 0.2657 | 1.2942 | 0.9496 | 0.7311 | 0.2934 | 1.3804 | 0.6544 | 0.4446 | 0.4454 | 1.1588 | 0.9548 | 0.8872 | 0.6303 | 1.6596 | 0.1087 | 0.5495 | 0.1433 | 1.0093 | 0.4082 | 1.2023 | 0.4805 | 1.5704 | 0.3881 | 0.7379 | 0.5219 | 1.0765 | 0.9787 |
| 508 | O00151 | PDZ and LIM domain protein 1 (C-terminal LIM domain protein 1) (Elfin) (LIM domain protein CLP-36) | PDLIM1 CLIM1 CLP36 | 6 | 14 | 1.1272 | 0.8185 | 1.3305 | 0.3451 | 1.0471 | 0.8102 | 1.8880 | 0.3184 | 1.4060 | 0.5464 | 2.0137 | 0.2760 | 1.1066 | 0.7375 | 1.4723 | 0.3556 | 1.2359 | 0.5800 | 0.9462 | 0.4573 | 1.3305 | 0.8450 | 1.7865 | 0.2964 | 0.3597 | 0.1161 | 2.0701 | 0.2758 |
| 509 | Q15084 | Protein disulfide-isomerase A6 (EC 5.3.4.1) (Endoplasmic reticulum protein 5) (ER protein 5) (ERp5) (Protein disulfide isomerase P5) (Thioredoxin domain-containing protein 7) | PDIA6 ERP5 P5 TXNDC7 | 5 | 29 | 1.1588 | 0.5090 | 0.9727 | 0.8676 | 1.3428 | 0.1985 | 0.9376 | 0.7290 | 0.6546 | 0.9183 | 0.9376 | 0.9576 | 1.3552 | 0.5945 | 0.7586 | 0.2426 | 1.1482 | 0.6454 | 1.0280 | 0.8213 | 0.1905 | 0.1770 | 1.1695 | 0.1964 | 0.8710 | 0.7777 | 0.7586 | 0.9859 |
| 510 | Q14554 | Protein disulfide-isomerase A5 (EC 5.3.4.1) (Protein disulfide isomerase-related protein) | PDIA5 PDIR | 16 | 11 | 1.2359 | 0.3715 | 1.1912 | 0.4776 | 1.5704 | 0.6797 | 1.0375 | 0.5113 | 2.0137 | 0.2742 | 1.0186 | 0.8360 | 1.5276 | 0.7665 | 0.5297 | 0.0011 | 1.2246 | 0.7593 | 0.4920 | 0.0381 | 1.4997 | 0.3208 | 1.0186 | 0.7707 | 1.3062 | 0.8403 | 0.4406 | 0.0289 |
| 511 | P13667 | Protein disulfide-isomerase A4 (EC 5.3.4.1) (Endoplasmic reticulum resident protein 70) (ER protein 70) (ERp70) (Endoplasmic reticulum resident protein 72) (ER protein 72) (ERp-72) (ERp72) | PDIA4 ERP70 ERP72 | 39 | 50 | 0.7311 | 0.3380 | 0.7727 | 0.3051 | 1.4454 | 0.1967 | 1.1482 | 0.3481 | 0.9376 | 0.7437 | 1.5996 | 0.5657 | 1.7378 | 0.0040 | 0.6194 | 0.0046 | 1.3677 | 0.2046 | 1.8030 | 0.0363 | 1.4997 | 0.0273 | 1.4454 | 0.4004 | 0.6081 | 0.2530 | 1.1169 | 0.1206 |
| 512 | P30101 | Protein disulfide-isomerase A3 (EC 5.3.4.1) (58 kDa glucose-regulated protein) (58 kDa microsomal protein) (p58) (Disulfide isomerase ER-60) (Endoplasmic reticulum resident protein 57) (ER protein 57) (ERp57) (Endoplasmic reticulum resident protein 60) (ER protein 60) (ERp60) | PDIA3 ERP57 ERP60 GRP58 | 12 | 46 | 0.7516 | 0.0540 | 0.8395 | 0.1086 | 0.8166 | 0.2039 | 0.9036 | 0.1222 | 0.6607 | 0.0618 | 1.3428 | 0.5029 | 0.5495 | 0.0111 | 0.5546 | 0.0013 | 1.3183 | 0.7762 | 1.3428 | 0.6761 | 0.2421 | 0.0005 | 1.3932 | 0.3880 | 0.3076 | 0.0041 | 1.0280 | 0.7952 |
| 513 | P11177 | Pyruvate dehydrogenase E1 component subunit beta, mitochondrial (PDHE1-B) (EC 1.2.4.1) | PDHB PHE1B | 55 | 8 | 0.5916 | 0.0534 | 0.8395 | 0.8668 | 0.9817 | 0.8416 | 1.1588 | 0.6762 | 0.6310 | 0.0121 | 1.9231 | 0.1738 | 1.4588 | 0.0128 | 1.6596 | 0.1708 | 0.7586 | 0.1579 | 1.2359 | 0.6004 | 1.0186 | 0.7740 | 1.6144 | 0.1705 | 0.5970 | 0.0008 | 1.5849 | 0.2170 |
| 514 | P08559 | Pyruvate dehydrogenase E1 component subunit alpha, somatic form, mitochondrial (EC 1.2.4.1) (PDHE1-A type I) | PDHA1 PHE1A | 57 | 7 | 0.5754 | 0.0197 | 0.4742 | 0.4113 | 1.1588 | 0.8493 | 0.5702 | 0.4648 | 0.6486 | 0.2899 | 0.6486 | 0.4492 | 1.5704 | 0.0180 | 0.9550 | 0.6778 | 1.0864 | 0.6901 | 0.7943 | 0.2250 | 1.6293 | 0.2116 | 1.0568 | 0.7774 | 0.6194 | 0.1010 | 0.9036 | 0.7286 |
| 515 | Q8WUM4 | Programmed cell death 6-interacting protein (PDCD6-interacting protein) (ALG-2-interacting protein 1) (ALG-2-interacting protein X) (Hp95) | PDCD6IP AIP1 ALIX KIAA1375 | 6 | 17 | 1.4060 | 0.1682 | 2.2284 | 0.0891 | 1.0471 | 0.6562 | 1.2942 | 0.1631 | 1.3552 | 0.1513 | 2.9923 | 0.0056 | 1.1803 | 0.5713 | 2.9923 | 0.0406 | 1.5136 | 0.0915 | 1.4322 | 0.2046 | 0.9727 | 0.9928 | 1.8707 | 0.2382 | 1.4322 | 0.0754 | 2.5119 | 0.0163 |
| 516 | Q99447 | Ethanolamine-phosphate cytidylyltransferase (EC 2.7.7.14) (CTP:phosphoethanolamine cytidylyltransferase) (Phosphorylethanolamine transferase) | PCYT2 | 9 | 6 | 1.4322 | 0.1797 | 1.2246 | 0.5233 | 0.5861 | 0.5489 | 1.9055 | 0.7359 | 1.3305 | 0.4478 | 1.0666 | 0.7997 | 0.8551 | 0.5414 | 1.6444 | 0.9880 | 1.5560 | 0.2850 | 1.0965 | 0.7491 | 1.8197 | 0.4613 | 2.0893 | 0.4262 | 1.6904 | 0.2156 | 0.5970 | 0.6993 |
| 517 | Q9UHG3 | Prenylcysteine oxidase 1 (EC 1.8.3.5) (Prenylcysteine lyase) | PCYOX1 KIAA0908 PCL1 UNQ597/PRO1183 | 21 | 15 | 1.7539 | 0.2065 | 0.9290 | 0.5303 | 1.5704 | 0.4206 | 1.5704 | 0.1112 | 1.7701 | 0.1780 | 1.4454 | 0.1146 | 2.2699 | 0.0592 | 0.3873 | 0.2703 | 1.5276 | 0.1022 | 1.1803 | 0.5383 | 1.5276 | 0.0401 | 1.0375 | 0.5374 | 0.8551 | 0.8876 | 0.6668 | 0.2145 |
| 518 | Q9UKL6 | Phosphatidylcholine transfer protein (PC-TP) (START domain-containing protein 2) (StARD2) (StAR-related lipid transfer protein 2) | PCTP STARD2 | 11 | 8 | 1.8197 | 0.3231 | 0.6607 | 0.7687 | 1.8365 | 0.2657 | 1.0000 | 0.9536 | 2.2491 | 0.0570 | 1.6444 | 0.5081 | 1.6904 | 0.5598 | 0.8790 | 0.5224 | 1.6749 | 0.3310 | 0.6026 | 0.8333 | 0.5702 | 0.2027 | 0.7112 | 0.7970 | 1.4322 | 0.4992 | 0.4529 | 0.7118 |
| 519 | P22061 | Protein-L-isoaspartate(D-aspartate) O-methyltransferase (PIMT) (EC 2.1.1.77) (L-isoaspartyl protein carboxyl methyltransferase) (Protein L-isoaspartyl/D-aspartyl methyltransferase) (Protein-beta-aspartate methyltransferase) | PCMT1 | 13 | 6 | 1.9409 | 0.0309 | 2.4660 | 0.9988 | 1.6444 | 0.0248 | 1.8365 | 0.8978 | 1.3305 | 0.3672 | 2.5351 | 0.8835 | 1.6444 | 0.0922 | 2.3768 | 0.9961 | 1.6293 | 0.0783 | 1.5849 | 0.6951 | 0.9376 | 0.9942 | 2.0701 | 0.7338 | 0.8710 | 0.7828 | 1.0666 | 0.5465 |
| 520 | Q16822 | Phosphoenolpyruvate carboxykinase [GTP], mitochondrial (PEPCK-M) (EC 4.1.1.32) | PCK2 PEPCK2 | 4 | 78 | 1.8707 | 0.4748 | 0.9036 | 0.1896 | 1.0471 | 0.8606 | 1.1588 | 0.8343 | 0.6730 | 0.6461 | 0.5248 | 0.0003 | 1.0765 | 0.7766 | 0.8091 | 0.0489 | 1.0280 | 0.6110 | 0.3597 | 0.0001 | 1.2023 | 0.9403 | 0.8166 | 0.1853 | 0.6982 | 0.4340 | 0.2466 | 0.0000 |
| 521 | P35558 | Phosphoenolpyruvate carboxykinase, cytosolic [GTP] (PEPCK-C) (EC 4.1.1.32) | PCK1 PEPCK1 | 4 | 10 | 1.6444 | 0.1796 | 1.2474 | 0.8793 | 1.5849 | 0.1993 | 1.9409 | 0.4756 | 1.4322 | 0.2727 | 0.3467 | 0.1286 | 1.4723 | 0.2460 | 0.1959 | 0.1789 | 1.6144 | 0.1844 | 0.4656 | 0.1262 | 1.1912 | 0.5072 | 0.3908 | 0.1706 | 1.3305 | 0.3417 | 0.5861 | 0.1274 |
| 522 | P05166 | Propionyl-CoA carboxylase beta chain, mitochondrial (PCCase subunit beta) (EC 6.4.1.3) (Propanoyl-CoA:carbon dioxide ligase subunit beta) | PCCB | 79 | 28 | 0.9817 | 0.1249 | 0.9550 | 0.8717 | 1.0864 | 0.7554 | 0.8630 | 0.5357 | 0.6855 | 0.0281 | 0.7178 | 0.4759 | 0.8241 | 0.0158 | 1.0568 | 0.2325 | 1.1482 | 0.9188 | 1.0765 | 0.7032 | 0.0466 | 0.0000 | 1.1588 | 0.3047 | 0.3105 | 0.0000 | 0.6486 | 0.0312 |
| 523 | P05165 | Propionyl-CoA carboxylase alpha chain, mitochondrial (PCCase subunit alpha) (EC 6.4.1.3) (Propanoyl-CoA:carbon dioxide ligase subunit alpha) | PCCA | 13 | 25 | 0.7112 | 0.3076 | 0.6026 | 0.1113 | 0.4656 | 0.0276 | 0.5861 | 0.0407 | 0.3162 | 0.0847 | 0.2992 | 0.0009 | 1.9055 | 0.0655 | 0.8630 | 0.1436 | 0.9638 | 0.4959 | 0.7447 | 0.0249 | 0.4875 | 0.0388 | 0.6427 | 0.0047 | 0.6546 | 0.2496 | 0.5970 | 0.0039 |
| 524 | Q15366 | Poly(rC)-binding protein 2 (Alpha-CP2) (Heterogeneous nuclear ribonucleoprotein E2) (hnRNP E2) | PCBP2 | 27 | 19 | 0.8551 | 0.8575 | 1.6144 | 0.2543 | 1.2134 | 0.9132 | 1.3183 | 0.5779 | 0.9204 | 0.8713 | 2.4889 | 0.0554 | 1.4191 | 0.1330 | 0.9908 | 0.6041 | 1.1272 | 0.7314 | 1.2134 | 0.7651 | 0.4920 | 0.0016 | 1.8197 | 0.3388 | 0.7112 | 0.1355 | 1.2942 | 0.1414 |
| 525 | Q15365 | Poly(rC)-binding protein 1 (Alpha-CP1) (Heterogeneous nuclear ribonucleoprotein E1) (hnRNP E1) (Nucleic acid-binding protein SUB2.3) | PCBP1 | 23 | 24 | 0.9036 | 0.4042 | 0.6368 | 0.7997 | 0.8551 | 0.6263 | 1.6596 | 0.4891 | 0.5346 | 0.0117 | 1.8535 | 0.3876 | 1.0186 | 0.8577 | 2.2080 | 0.1627 | 0.7516 | 0.0804 | 1.7061 | 0.5790 | 0.4285 | 0.0001 | 1.7219 | 0.3363 | 0.7112 | 0.0265 | 1.5704 | 0.2663 |
| 526 | P61457 | Pterin-4-alpha-carbinolamine dehydratase (PHS) (EC 4.2.1.96) (4-alpha-hydroxy-tetrahydropterin dehydratase) (Dimerization cofactor of hepatocyte nuclear factor 1-alpha) (DCoH) (Dimerization cofactor of HNF1) (Phenylalanine hydroxylase-stimulating protein) (Pterin carbinolamine dehydratase) (PCD) | PCBD1 DCOH PCBD | 22 | 12 | 0.8630 | 0.5322 | 1.3305 | 0.4127 | 1.5704 | 0.2276 | 1.9055 | 0.0546 | 1.8030 | 0.1916 | 0.4055 | 0.7628 | 2.0701 | 0.1539 | 1.1376 | 0.8316 | 1.2246 | 0.7733 | 1.0965 | 0.3060 | 2.2284 | 0.0585 | 2.3768 | 0.0135 | 1.0186 | 0.8838 | 0.2582 | 0.3422 |
| 527 | P11498 | Pyruvate carboxylase, mitochondrial (EC 6.4.1.1) (Pyruvic carboxylase) (PCB) | PC | 23 | 76 | 0.9638 | 0.9475 | 0.6194 | 0.0067 | 1.3552 | 0.7070 | 0.8017 | 0.0957 | 1.5417 | 0.6403 | 0.3342 | 0.0000 | 1.5276 | 0.9663 | 0.5105 | 0.0002 | 1.1695 | 0.6251 | 0.5058 | 0.0000 | 1.8880 | 0.1608 | 0.3631 | 0.0000 | 1.2706 | 0.5973 | 0.4055 | 0.0000 |
| 528 | P30039 | Phenazine biosynthesis-like domain-containing protein (EC 5.1.-.-) (MAWD-binding protein) (MAWDBP) (Unknown protein 32 from 2D-page of liver tissue) | PBLD MAWBP | 11 | 29 | 1.5417 | 0.6943 | 1.4588 | 0.0360 | 1.3552 | 0.7389 | 1.7219 | 0.0288 | 1.7701 | 0.2202 | 0.6194 | 0.2889 | 1.8365 | 0.1987 | 1.4454 | 0.1465 | 1.1169 | 0.9725 | 1.2474 | 0.1295 | 0.1600 | 0.0321 | 1.1803 | 0.3507 | 0.2655 | 0.3253 | 0.2399 | 0.0199 |
| 529 | Q9NVD7 | Alpha-parvin (Actopaxin) (CH-ILKBP) (Calponin-like integrin-linked kinase-binding protein) (Matrix-remodeling-associated protein 2) | PARVA MXRA2 | 101 | 4 | 1.0471 | 0.5500 | 0.8551 | 0.9126 | 0.5152 | 0.0000 | 1.9770 | 0.2513 | 0.3802 | 0.0000 | 1.4060 | 0.5940 | 1.6144 | 0.0000 | 2.2909 | 0.2365 | 1.0186 | 0.1939 | 2.7040 | 0.2065 | 0.2729 | 0.0000 | 0.7379 | 0.6092 | 0.3597 | 0.0000 | 0.8091 | 0.8595 |
| 530 | Q8IXQ6 | Poly [ADP-ribose] polymerase 9 (PARP-9) (EC 2.4.2.30) (ADP-ribosyltransferase diphtheria toxin-like 9) (ARTD9) (B aggressive lymphoma protein) | PARP9 BAL BAL1 | 33 | 3 | 1.2942 | 0.7201 | 0.4169 | 0.2365 | 0.8017 | 0.2261 | 0.1486 | 0.0755 | 1.5136 | 0.0879 | 1.3804 | 0.6010 | 1.0186 | 0.8747 | 0.5546 | 0.2396 | 1.2359 | 0.8475 | 0.6194 | 0.3365 | 0.1318 | 0.0000 | 0.1019 | 0.0711 | 0.4406 | 0.0002 | 0.6855 | 0.5426 |
| 531 | P09874 | Poly [ADP-ribose] polymerase 1 (PARP-1) (EC 2.4.2.30) (ADP-ribosyltransferase diphtheria toxin-like 1) (ARTD1) (NAD(+) ADP-ribosyltransferase 1) (ADPRT 1) (Poly[ADP-ribose] synthase 1) | PARP1 ADPRT PPOL | 3 | 6 | 1.1169 | 0.5534 | 2.6792 | 0.3745 | 1.2942 | 0.2226 | 1.5136 | 0.8355 | 1.1169 | 0.8681 | 3.3419 | 0.2096 | 1.1803 | 0.5325 | 0.5105 | 0.6593 | 1.0375 | 0.8926 | 4.0179 | 0.0934 | 1.1803 | 0.2617 | 1.7061 | 0.8973 | 1.0000 | 0.6368 | 6.2517 | 0.0255 |
| 532 | Q99497 | Protein deglycase DJ-1 (DJ-1) (EC 3.1.2.-) (EC 3.5.1.-) (Oncogene DJ1) (Parkinson disease protein 7) | PARK7 | 3 | 23 | 1.6144 | 0.3020 | 1.1912 | 0.4228 | 0.9550 | 0.9952 | 2.3335 | 0.0645 | 1.1803 | 0.6332 | 2.1281 | 0.6720 | 1.1588 | 0.6225 | 2.7797 | 0.0692 | 1.1169 | 0.7683 | 1.4997 | 0.5000 | 1.1588 | 0.7155 | 2.6546 | 0.0385 | 1.2474 | 0.4918 | 0.8395 | 0.8701 |
| 533 | O95340 | Bifunctional 3'-phosphoadenosine 5'-phosphosulfate synthase 2 (PAPS synthase 2) (PAPSS 2) (Sulfurylase kinase 2) (SK 2) (SK2) [Includes: Sulfate adenylyltransferase (EC 2.7.7.4) (ATP-sulfurylase) (Sulfate adenylate transferase) (SAT); Adenylyl-sulfate kinase (EC 2.7.1.25) (3'-phosphoadenosine-5'-phosphosulfate synthase) (APS kinase) (Adenosine-5'-phosphosulfate 3'-phosphotransferase) (Adenylylsulfate 3'-phosphotransferase)] | PAPSS2 ATPSK2 | 9 | 13 | 1.6904 | 0.0829 | 0.3251 | 0.1020 | 2.5351 | 0.0020 | 0.9290 | 0.9522 | 1.9953 | 0.0154 | 0.9036 | 0.9386 | 1.7378 | 0.0529 | 0.8551 | 0.4571 | 2.0701 | 0.0147 | 0.8872 | 0.8320 | 3.1915 | 0.0007 | 0.8710 | 0.7231 | 2.7797 | 0.0065 | 1.2589 | 0.9166 |
| 534 | A6NDB9 | Paralemmin-3 | PALM3 | 26 | 4 | 1.2823 | 0.9327 | 0.8318 | 0.3757 | 0.9462 | 0.8994 | 1.1169 | 0.4712 | 1.6444 | 0.0613 | 0.7798 | 0.2338 | 1.4859 | 0.1877 | 1.0186 | 0.8782 | 1.3062 | 0.1827 | 0.9638 | 0.8960 | 0.4207 | 0.0056 | 0.9908 | 0.9675 | 0.5012 | 0.0290 | 1.0568 | 0.6595 |
| 535 | Q13177 | Serine/threonine-protein kinase PAK 2 (EC 2.7.11.1) (Gamma-PAK) (PAK65) (S6/H4 kinase) (p21-activated kinase 2) (PAK-2) (p58) [Cleaved into: PAK-2p27 (p27); PAK-2p34 (p34) (C-t-PAK2)] | PAK2 | 8 | 4 | 0.8954 | 0.2128 | 0.8091 | 0.7357 | 0.9376 | 0.6102 | 1.8880 | 0.4198 | 0.7516 | 0.2039 | 0.6982 | 0.7307 | 0.7656 | 0.5766 | 2.2909 | 0.1820 | 0.5916 | 0.0234 | 2.4434 | 0.3402 | 0.3499 | 0.0074 | 1.4454 | 0.5384 | 0.7311 | 0.1832 | 1.9770 | 0.2898 |
| 536 | P22234 | Multifunctional protein ADE2 [Includes: Phosphoribosylaminoimidazole-succinocarboxamide synthase (EC 6.3.2.6) (SAICAR synthetase); Phosphoribosylaminoimidazole carboxylase (EC 4.1.1.21) (AIR carboxylase) (AIRC)] | PAICS ADE2 AIRC PAIS | 4 | 10 | 2.0701 | 0.1006 | 0.9204 | 0.7263 | 0.9290 | 0.9480 | 0.8166 | 0.4584 | 1.3552 | 0.2794 | 0.9204 | 0.9144 | 0.6138 | 0.8438 | 1.6293 | 0.1592 | 1.2359 | 0.4661 | 1.2706 | 0.6236 | 0.2070 | 0.2107 | 0.6546 | 0.8648 | 1.1803 | 0.5941 | 1.0280 | 0.8381 |
| 537 | P00439 | Phenylalanine-4-hydroxylase (PAH) (EC 1.14.16.1) (Phe-4-monooxygenase) | PAH | 5 | 14 | 0.9550 | 0.8308 | 1.3804 | 0.8837 | 0.9204 | 0.9878 | 1.9770 | 0.0250 | 1.1066 | 0.4350 | 0.8630 | 0.1647 | 0.8017 | 0.9273 | 1.4588 | 0.9589 | 1.1695 | 0.9002 | 1.1803 | 0.5419 | 2.9376 | 0.0582 | 1.3305 | 0.8413 | 2.0893 | 0.1193 | 0.9376 | 0.2891 |
| 538 | P43034 | Platelet-activating factor acetylhydrolase IB subunit alpha (Lissencephaly-1 protein) (LIS-1) (PAF acetylhydrolase 45 kDa subunit) (PAF-AH 45 kDa subunit) (PAF-AH alpha) (PAFAH alpha) | PAFAH1B1 LIS1 MDCR MDS PAFAHA | 10 | 6 | 1.1272 | 0.9948 | 0.7798 | 0.6132 | 1.0568 | 0.5988 | 0.8395 | 0.4560 | 1.4322 | 0.7958 | 0.3532 | 0.1068 | 1.4859 | 0.7300 | 0.8630 | 0.6564 | 1.5136 | 0.2732 | 0.4018 | 0.2055 | 0.8551 | 0.6655 | 0.6546 | 0.5786 | 0.7047 | 0.9911 | 1.0471 | 0.9668 |
| 539 | Q9UKS6 | Protein kinase C and casein kinase substrate in neurons protein 3 (SH3 domain-containing protein 6511) | PACSIN3 | 25 | 5 | 1.9055 | 0.1085 | 0.7943 | 0.7368 | 1.4588 | 0.3454 | 0.7943 | 0.7971 | 1.7378 | 0.1534 | 0.6792 | 0.4816 | 1.4723 | 0.7811 | 0.6855 | 0.7528 | 1.6444 | 0.1603 | 0.4169 | 0.5410 | 0.7727 | 0.6057 | 0.8872 | 0.9131 | 0.9376 | 0.3489 | 0.3802 | 0.0609 |
| 540 | Q13310 | Polyadenylate-binding protein 4 (PABP-4) (Poly(A)-binding protein 4) (Activated-platelet protein 1) (APP-1) (Inducible poly(A)-binding protein) (iPABP) | PABPC4 APP1 PABP4 | 4 | 13 | 0.7311 | 0.3108 | 1.5417 | 0.3403 | 0.5200 | 0.0812 | 1.5276 | 0.4498 | 0.5970 | 0.1367 | 1.3804 | 0.3022 | 0.6081 | 0.1190 | 1.4859 | 0.4988 | 0.6427 | 0.2941 | 1.5704 | 0.4038 | 0.9817 | 0.8243 | 1.7539 | 0.2640 | 0.6918 | 0.9167 | 1.1482 | 0.9348 |
| 541 | P11940 | Polyadenylate-binding protein 1 (PABP-1) (Poly(A)-binding protein 1) | PABPC1 PAB1 PABP1 PABPC2 | 4 | 20 | 0.9638 | 0.9577 | 1.2246 | 0.8509 | 0.8395 | 0.7151 | 1.5560 | 0.5750 | 0.9376 | 0.9119 | 2.0324 | 0.1774 | 0.9376 | 0.9041 | 0.8318 | 0.7774 | 1.2359 | 0.6472 | 1.1803 | 0.6091 | 0.5495 | 0.3350 | 2.1677 | 0.1991 | 0.6368 | 0.4271 | 1.1482 | 0.6157 |
| 542 | Q9UQ80 | Proliferation-associated protein 2G4 (Cell cycle protein p38-2G4 homolog) (hG4-1) (ErbB3-binding protein 1) | PA2G4 EBP1 | 17 | 10 | 1.0093 | 0.5211 | 1.1588 | 0.3687 | 1.2589 | 0.9353 | 0.6138 | 0.5420 | 0.4169 | 0.0288 | 1.0375 | 0.4654 | 1.1066 | 0.8722 | 0.7244 | 0.5147 | 1.2246 | 0.7272 | 0.9204 | 0.3890 | 0.9120 | 0.5228 | 1.1588 | 0.3574 | 1.0864 | 0.6279 | 1.2706 | 0.3396 |
| 543 | P07237 | Protein disulfide-isomerase (PDI) (EC 5.3.4.1) (Cellular thyroid hormone-binding protein) (Prolyl 4-hydroxylase subunit beta) (p55) | P4HB ERBA2L PDI PDIA1 PO4DB | 25 | 92 | 0.8166 | 0.6849 | 0.4207 | 0.0032 | 1.4723 | 0.3150 | 0.5808 | 0.0083 | 1.4723 | 0.5775 | 0.6982 | 0.0168 | 1.3677 | 0.4068 | 0.4325 | 0.0005 | 0.7244 | 0.2872 | 0.9290 | 0.5922 | 2.6546 | 0.0027 | 1.5704 | 0.1370 | 1.6444 | 0.2102 | 0.8395 | 0.7579 |
| 544 | Q96FW1 | Ubiquitin thioesterase OTUB1 (EC 3.4.19.12) (Deubiquitinating enzyme OTUB1) (OTU domain-containing ubiquitin aldehyde-binding protein 1) (Otubain-1) (hOTU1) (Ubiquitin-specific-processing protease OTUB1) | OTUB1 OTB1 OTU1 HSPC263 | 7 | 4 | 1.0965 | 0.4323 | 1.7378 | 0.3679 | 1.3305 | 0.6076 | 1.1588 | 0.8375 | 1.2359 | 0.2167 | 1.7219 | 0.7418 | 1.2023 | 0.8998 | 1.6444 | 0.7094 | 1.3804 | 0.8749 | 1.6293 | 0.6413 | 2.6062 | 0.0482 | 1.1169 | 0.7978 | 1.6749 | 0.4786 | 1.6144 | 0.6057 |
| 545 | P00480 | Ornithine carbamoyltransferase, mitochondrial (EC 2.1.3.3) (Ornithine transcarbamylase) (OTCase) | OTC | 117 | 50 | 0.3565 | 0.0000 | 0.9204 | 0.5656 | 1.1803 | 0.6360 | 1.0765 | 0.3542 | 0.8790 | 0.1624 | 0.1706 | 0.0001 | 2.0512 | 0.0033 | 1.0965 | 0.4278 | 0.8241 | 0.0266 | 1.1588 | 0.8501 | 1.3428 | 0.0171 | 0.8954 | 0.4678 | 0.4920 | 0.0060 | 1.4060 | 0.1237 |
| 546 | Q92882 | Osteoclast-stimulating factor 1 | OSTF1 | 4 | 3 | 2.0137 | 0.2183 | 1.3552 | 0.4527 | 2.1281 | 0.1339 | 1.1588 | 0.7626 | 2.4210 | 0.1909 | 0.9817 | 0.9312 | 1.6293 | 0.5412 | 0.9204 | 0.6958 | 2.3550 | 0.1686 | 0.9036 | 0.6345 | 3.1915 | 0.0418 | 0.8710 | 0.6078 | 3.1333 | 0.0450 | 1.0568 | 0.9744 |
| 547 | P22059 | Oxysterol-binding protein 1 | OSBP OSBP1 | 57 | 6 | 1.2134 | 0.6231 | 0.7178 | 0.6728 | 1.4588 | 0.4090 | 0.5297 | 0.1140 | 0.4365 | 0.0006 | 1.0280 | 0.5365 | 1.6144 | 0.3159 | 0.8166 | 0.4492 | 1.7061 | 0.1826 | 0.8872 | 0.7358 | 0.2168 | 0.0002 | 0.6855 | 0.6005 | 0.5916 | 0.0007 | 1.0471 | 0.5261 |
| 548 | P02763 | Alpha-1-acid glycoprotein 1 (AGP 1) (Orosomucoid-1) (OMD 1) | ORM1 AGP1 | 3 | 7 | 0.7516 | 0.9796 | 0.4529 | 0.0090 | 0.8551 | 0.8289 | 0.5105 | 0.0374 | 0.8872 | 0.5714 | 0.7311 | 0.1544 | 0.1556 | 0.2679 | 0.4130 | 0.0150 | 0.6310 | 0.7862 | 0.7727 | 0.2360 | 1.2823 | 0.2450 | 0.9908 | 0.5077 | 1.5996 | 0.2009 | 0.3404 | 0.0725 |
| 549 | O14841 | 5-oxoprolinase (EC 3.5.2.9) (5-oxo-L-prolinase) (5-OPase) (Pyroglutamase) | OPLAH | 4 | 19 | 0.8318 | 0.8276 | 2.0893 | 0.0241 | 0.6855 | 0.2638 | 1.6749 | 0.1781 | 1.7865 | 0.3718 | 0.9204 | 0.6328 | 0.9290 | 0.9043 | 1.2823 | 0.6526 | 1.2823 | 0.8202 | 0.5702 | 0.7940 | 1.3305 | 0.7600 | 1.1482 | 0.0504 | 1.2023 | 0.5809 | 0.5861 | 0.2053 |
| 550 | O60313 | Dynamin-like 120 kDa protein, mitochondrial (EC 3.6.5.5) (Optic atrophy protein 1) [Cleaved into: Dynamin-like 120 kDa protein, form S1] | OPA1 KIAA0567 | 6 | 4 | 0.3162 | 0.0459 | 1.2589 | 0.4794 | 0.8241 | 0.7241 | 1.0568 | 0.8122 | 0.4055 | 0.0766 | 0.8395 | 0.5172 | 0.3698 | 0.0967 | 1.0471 | 0.9236 | 0.1888 | 0.0447 | 1.1376 | 0.4664 | 0.1905 | 0.0556 | 1.2359 | 0.2876 | 0.2312 | 0.0292 | 1.0000 | 0.7049 |
| 551 | Q9NTK5 | Obg-like ATPase 1 (DNA damage-regulated overexpressed in cancer 45) (DOC45) (GTP-binding protein 9) | OLA1 GTPBP9 PRO2455 PTD004 | 10 | 7 | 1.0965 | 0.4826 | 1.3183 | 0.8190 | 1.1066 | 0.3235 | 2.4434 | 0.4227 | 0.6252 | 0.3745 | 3.9084 | 0.0674 | 0.7379 | 0.5114 | 3.4041 | 0.1200 | 0.8630 | 0.6784 | 3.7325 | 0.0571 | 0.2333 | 0.0504 | 2.3335 | 0.0716 | 0.8872 | 0.9862 | 3.9811 | 0.0031 |
| 552 | P20774 | Mimecan (Osteoglycin) (Osteoinductive factor) (OIF) | OGN OIF SLRR3A | 7 | 9 | 1.1695 | 0.2646 | 0.4093 | 0.0331 | 1.0186 | 0.8085 | 1.4723 | 0.0771 | 1.2134 | 0.1910 | 0.6546 | 0.1799 | 0.9290 | 0.7862 | 0.8318 | 0.5713 | 1.1695 | 0.3046 | 0.3698 | 0.1873 | 0.9727 | 0.9391 | 0.1472 | 0.0152 | 1.2474 | 0.2972 | 0.3162 | 0.1205 |
| 553 | Q9ULD0 | 2-oxoglutarate dehydrogenase-like, mitochondrial (EC 1.2.4.-) (2-oxoglutarate dehydrogenase complex component E1-like) (OGDC-E1-like) (Alpha-ketoglutarate dehydrogenase-like) | OGDHL KIAA1290 | 3 | 16 | 5.7544 | 0.3109 | 1.0280 | 0.9846 | 2.5823 | 0.3673 | 1.2359 | 0.6396 | 5.1051 | 0.3043 | 0.6855 | 0.5003 | 6.9823 | 0.2795 | 0.8790 | 0.5976 | 3.4995 | 0.3517 | 0.7447 | 0.6605 | 4.9659 | 0.3151 | 1.3932 | 0.3476 | 4.6132 | 0.2995 | 0.8241 | 0.7216 |
| 554 | Q02218 | 2-oxoglutarate dehydrogenase, mitochondrial (EC 1.2.4.2) (2-oxoglutarate dehydrogenase complex component E1) (OGDC-E1) (Alpha-ketoglutarate dehydrogenase) | OGDH | 12 | 19 | 0.2399 | 0.1301 | 1.2134 | 0.4766 | 4.6989 | 0.0006 | 1.5276 | 0.1381 | 0.3342 | 0.0504 | 0.8166 | 0.5483 | 1.5849 | 0.9941 | 1.4723 | 0.0704 | 1.7378 | 0.3629 | 1.5136 | 0.5813 | 0.2911 | 0.0702 | 1.9055 | 0.1364 | 0.1754 | 0.0670 | 1.0765 | 0.3178 |
| 555 | Q9NX40 | OCIA domain-containing protein 1 (Ovarian carcinoma immunoreactive antigen) | OCIAD1 OCIA | 10 | 6 | 1.6144 | 0.1976 | 1.0093 | 0.6951 | 1.2474 | 0.7395 | 0.9376 | 0.6706 | 0.9550 | 0.3332 | 1.3183 | 0.2712 | 1.4588 | 0.1604 | 1.4997 | 0.1008 | 1.3062 | 0.6393 | 1.1695 | 0.4067 | 0.3404 | 0.0273 | 1.1695 | 0.6822 | 0.5808 | 0.2361 | 1.4191 | 0.1896 |
| 556 | P04181 | Ornithine aminotransferase, mitochondrial (EC 2.6.1.13) (Ornithine delta-aminotransferase) (Ornithine--oxo-acid aminotransferase) [Cleaved into: Ornithine aminotransferase, hepatic form; Ornithine aminotransferase, renal form] | OAT | 14 | 11 | 1.0280 | 0.8537 | 1.4997 | 0.0641 | 0.9462 | 0.7074 | 2.0893 | 0.0044 | 0.7727 | 0.1823 | 0.2188 | 0.0140 | 1.1482 | 0.3543 | 1.9953 | 0.0013 | 0.7516 | 0.2428 | 1.4191 | 0.0199 | 0.4571 | 0.0368 | 1.6444 | 0.0246 | 0.4920 | 0.0454 | 0.1528 | 0.0051 |
| 557 | Q14980 | Nuclear mitotic apparatus protein 1 (NuMA protein) (Nuclear matrix protein-22) (NMP-22) (SP-H antigen) | NUMA1 NMP22 NUMA | 6 | 6 | 1.4723 | 0.3764 | 0.9638 | 0.8400 | 1.1912 | 0.5842 | 1.0375 | 0.6260 | 0.7870 | 0.6780 | 0.9638 | 0.8409 | 1.4588 | 0.2107 | 1.1482 | 0.2057 | 1.5996 | 0.3052 | 0.9120 | 0.7421 | 1.0375 | 0.9161 | 0.9638 | 0.8727 | 1.2359 | 0.7996 | 1.0093 | 0.8927 |
| 558 | Q9UKK9 | ADP-sugar pyrophosphatase (EC 3.6.1.13) (8-oxo-dGDP phosphatase) (EC 3.6.1.58) (Nucleoside diphosphate-linked moiety X motif 5) (Nudix motif 5) (YSA1H) | NUDT5 HSPC115 | 10 | 9 | 2.6792 | 0.0028 | 1.0666 | 0.9560 | 3.4356 | 0.0003 | 1.1695 | 0.9835 | 1.9588 | 0.1288 | 1.3804 | 0.3542 | 1.4322 | 0.6725 | 1.7219 | 0.0596 | 0.7943 | 0.2289 | 0.9462 | 0.9014 | 0.7586 | 0.1886 | 1.9055 | 0.1080 | 2.4889 | 0.0161 | 0.5754 | 0.5246 |
| 559 | Q9Y266 | Nuclear migration protein nudC (Nuclear distribution protein C homolog) | NUDC | 9 | 4 | 0.5649 | 0.2853 | 0.1472 | 0.1741 | 0.7516 | 0.3138 | 0.1355 | 0.1598 | 0.9908 | 0.7235 | 0.3105 | 0.2667 | 0.8091 | 0.4982 | 0.3837 | 0.2223 | 1.2823 | 0.6016 | 0.2805 | 0.2964 | 0.7379 | 0.2200 | 1.0186 | 0.9603 | 1.7061 | 0.1516 | 0.6730 | 0.0943 |
| 560 | Q9H1E3 | Nuclear ubiquitous casein and cyclin-dependent kinase substrate 1 (P1) | NUCKS1 NUCKS JC7 | 8 | 5 | 1.3305 | 0.4893 | 0.5754 | 0.8278 | 1.0965 | 0.8622 | 0.5598 | 0.2689 | 1.6444 | 0.1197 | 1.2246 | 0.5703 | 0.9908 | 0.9279 | 1.0965 | 0.9761 | 1.1803 | 0.7590 | 1.3677 | 0.8394 | 0.8472 | 0.4830 | 1.0666 | 0.9930 | 0.8872 | 0.8245 | 1.2246 | 0.9916 |
| 561 | Q02818 | Nucleobindin-1 (CALNUC) | NUCB1 NUC | 4 | 7 | 0.8790 | 0.7884 | 0.5970 | 0.4646 | 0.9036 | 0.7479 | 0.7047 | 0.7414 | 0.9036 | 0.7910 | 0.8395 | 0.4177 | 1.0375 | 0.7367 | 0.5346 | 0.3114 | 0.9290 | 0.9302 | 1.0186 | 0.9817 | 1.0186 | 0.6899 | 0.9462 | 0.9426 | 1.1169 | 0.6737 | 1.5560 | 0.0711 |
| 562 | P21589 | 5'-nucleotidase (5'-NT) (EC 3.1.3.5) (Ecto-5'-nucleotidase) (CD antigen CD73) | NT5E NT5 NTE | 4 | 4 | 1.1376 | 0.4110 | 0.9120 | 0.5998 | 1.0765 | 0.4039 | 1.0375 | 0.9694 | 1.1482 | 0.4406 | 1.0666 | 0.9732 | 1.0186 | 0.4472 | 1.0765 | 0.9016 | 1.1912 | 0.3630 | 1.0093 | 0.9840 | 1.2134 | 0.3807 | 1.1066 | 0.9818 | 1.1588 | 0.4009 | 1.0186 | 0.9194 |
| 563 | Q9UNZ2 | NSFL1 cofactor p47 (UBX domain-containing protein 2C) (p97 cofactor p47) | NSFL1C UBXN2C | 8 | 8 | 1.1803 | 0.1809 | 0.8630 | 0.9008 | 1.0765 | 0.6114 | 0.7244 | 0.3163 | 0.8790 | 0.8949 | 1.8707 | 0.0515 | 0.6427 | 0.3017 | 0.6792 | 0.9375 | 0.8472 | 0.6758 | 0.4786 | 0.6421 | 0.8241 | 0.6457 | 1.5849 | 0.2452 | 1.0375 | 0.8494 | 1.5996 | 0.1082 |
| 564 | P46459 | Vesicle-fusing ATPase (EC 3.6.4.6) (N-ethylmaleimide-sensitive fusion protein) (NEM-sensitive fusion protein) (Vesicular-fusion protein NSF) | NSF | 5 | 5 | 1.0666 | 0.6992 | 0.5012 | 0.0673 | 1.0765 | 0.8592 | 0.8166 | 0.6557 | 1.1695 | 0.4155 | 1.1066 | 0.9641 | 1.0965 | 0.6147 | 1.2246 | 0.6268 | 0.8710 | 0.5695 | 1.3804 | 0.2525 | 1.0864 | 0.9740 | 0.9120 | 0.6888 | 0.9817 | 0.8329 | 1.1169 | 0.7979 |
| 565 | P16083 | Ribosyldihydronicotinamide dehydrogenase [quinone] (EC 1.10.5.1) (NRH dehydrogenase [quinone] 2) (NRH:quinone oxidoreductase 2) (Quinone reductase 2) (QR2) | NQO2 NMOR2 | 7 | 4 | 1.0965 | 0.4381 | 1.9770 | 0.5145 | 1.1376 | 0.1231 | 2.2284 | 0.1290 | 1.1482 | 0.7181 | 0.6427 | 0.1023 | 0.9817 | 0.8108 | 1.6444 | 0.7627 | 0.9727 | 0.7950 | 1.8880 | 0.1964 | 1.0568 | 0.6569 | 0.4487 | 0.1099 | 1.1169 | 0.4803 | 0.4920 | 0.1231 |
| 566 | P06748 | Nucleophosmin (NPM) (Nucleolar phosphoprotein B23) (Nucleolar protein NO38) (Numatrin) | NPM1 NPM | 4 | 15 | 0.5702 | 0.1310 | 1.1588 | 0.7909 | 1.2823 | 0.9761 | 1.0864 | 0.7865 | 1.0093 | 0.4341 | 1.2023 | 0.9321 | 0.8166 | 0.4722 | 1.0093 | 0.4168 | 1.2134 | 0.4773 | 1.4997 | 0.9229 | 1.0864 | 0.7433 | 1.5276 | 0.8287 | 1.4997 | 0.6187 | 1.3305 | 0.9452 |
| 567 | P55786 | Puromycin-sensitive aminopeptidase (PSA) (EC 3.4.11.14) (Cytosol alanyl aminopeptidase) (AAP-S) | NPEPPS PSA | 5 | 14 | 0.7244 | 0.1123 | 0.9908 | 0.9218 | 0.3802 | 0.0305 | 1.2942 | 0.2958 | 0.7311 | 0.1742 | 1.3183 | 0.1519 | 0.6792 | 0.1188 | 0.9036 | 0.9374 | 1.2706 | 0.7675 | 1.0000 | 0.6748 | 0.2489 | 0.0055 | 0.8395 | 0.8954 | 0.1393 | 0.0051 | 1.1482 | 0.7269 |
| 568 | P61916 | Epididymal secretory protein E1 (Human epididymis-specific protein 1) (He1) (Niemann-Pick disease type C2 protein) | NPC2 HE1 | 15 | 4 | 1.1588 | 0.7352 | 0.1528 | 0.0390 | 1.1912 | 0.9025 | 0.2992 | 0.0666 | 1.5136 | 0.4550 | 0.6607 | 0.1906 | 0.9376 | 0.9092 | 0.3251 | 0.0661 | 0.9036 | 0.6163 | 1.3062 | 0.5000 | 2.0137 | 0.1284 | 0.7586 | 0.3969 | 1.0000 | 0.7645 | 1.1482 | 0.9787 |
| 569 | Q9Y2X3 | Nucleolar protein 58 (Nucleolar protein 5) | NOP58 NOL5 NOP5 HSPC120 | 7 | 3 | 1.0375 | 0.9693 | 0.4130 | 0.2812 | 1.0186 | 0.7353 | 0.8166 | 0.4898 | 1.0093 | 0.8104 | 0.6918 | 0.3308 | 1.0186 | 0.9577 | 0.6730 | 0.4423 | 1.0186 | 0.7503 | 0.6730 | 0.3269 | 0.9638 | 0.6333 | 0.7447 | 0.2013 | 1.0965 | 0.3296 | 0.6081 | 0.1513 |
| 570 | Q15233 | Non-POU domain-containing octamer-binding protein (NonO protein) (54 kDa nuclear RNA- and DNA-binding protein) (55 kDa nuclear protein) (DNA-binding p52/p100 complex, 52 kDa subunit) (NMT55) (p54(nrb)) (p54nrb) | NONO NRB54 | 3 | 16 | 0.2128 | 0.1092 | 0.5649 | 0.1463 | 0.2291 | 0.0792 | 0.4571 | 0.0672 | 0.5248 | 0.5161 | 0.8091 | 0.4503 | 0.5861 | 0.2174 | 0.3767 | 0.0575 | 0.2582 | 0.1583 | 0.9204 | 0.6118 | 0.7379 | 0.8104 | 1.0375 | 0.7214 | 0.9204 | 0.9534 | 1.1272 | 0.5119 |
| 571 | Q5JPE7 | Nodal modulator 2 (pM5 protein 2) | NOMO2 | 3 | 11 | 2.4660 | 0.0657 | 1.0765 | 0.5392 | 1.0568 | 0.8747 | 1.4322 | 0.2591 | 1.8197 | 0.2105 | 1.7865 | 0.2105 | 1.2359 | 0.9844 | 1.2246 | 0.4800 | 1.1695 | 0.9563 | 1.9770 | 0.1016 | 1.1169 | 0.9539 | 1.3062 | 0.5460 | 0.5012 | 0.8641 | 1.2134 | 0.8552 |
| 572 | Q13423 | NAD(P) transhydrogenase, mitochondrial (EC 1.6.1.2) (Nicotinamide nucleotide transhydrogenase) (Pyridine nucleotide transhydrogenase) | NNT | 13 | 55 | 0.9376 | 0.4629 | 0.5495 | 0.0131 | 1.5560 | 0.2553 | 0.5445 | 0.0161 | 1.1588 | 0.6540 | 0.3767 | 0.0000 | 1.1588 | 0.6532 | 1.5417 | 0.0046 | 0.6918 | 0.2284 | 1.1482 | 0.9015 | 2.6303 | 0.0270 | 1.4588 | 0.1297 | 1.5560 | 0.8680 | 1.3552 | 0.0269 |
| 573 | P40261 | Nicotinamide N-methyltransferase (EC 2.1.1.1) | NNMT | 6 | 11 | 1.1482 | 0.1224 | 0.3802 | 0.0074 | 1.0280 | 0.6947 | 0.6081 | 0.2047 | 0.9550 | 0.9522 | 0.2559 | 0.0015 | 1.0965 | 0.3249 | 0.0673 | 0.0014 | 1.1066 | 0.1542 | 0.8790 | 0.4678 | 0.9204 | 0.5221 | 0.1722 | 0.0933 | 1.0864 | 0.3582 | 0.2992 | 0.0029 |
| 574 | P30419 | Glycylpeptide N-tetradecanoyltransferase 1 (EC 2.3.1.97) (Myristoyl-CoA:protein N-myristoyltransferase 1) (NMT 1) (Type I N-myristoyltransferase) (Peptide N-myristoyltransferase 1) | NMT1 NMT | 59 | 8 | 0.7656 | 0.0096 | 1.1803 | 0.3412 | 1.0186 | 0.3788 | 1.0666 | 0.3630 | 1.2246 | 0.6838 | 1.2134 | 0.2699 | 1.4588 | 0.1216 | 1.3183 | 0.2113 | 0.7943 | 0.0157 | 1.0568 | 0.7345 | 0.3698 | 0.0000 | 1.4997 | 0.0913 | 0.7798 | 0.0068 | 1.1803 | 0.5262 |
| 575 | Q9NQR4 | Omega-amidase NIT2 (EC 3.5.1.3) (Nitrilase homolog 2) | NIT2 CUA002 | 16 | 21 | 0.5395 | 0.4975 | 0.6138 | 0.2537 | 0.5105 | 0.5050 | 1.0765 | 0.7365 | 0.2014 | 0.7546 | 1.1482 | 0.3874 | 4.0926 | 0.1518 | 2.1086 | 0.0062 | 0.1445 | 0.9410 | 1.5136 | 0.2144 | 1.1695 | 0.3948 | 2.3768 | 0.0006 | 0.3342 | 0.5877 | 0.4742 | 0.0557 |
| 576 | Q86X76 | Nitrilase homolog 1 (EC 3.5.-.-) | NIT1 | 3 | 7 | 2.4210 | 0.2639 | 0.5058 | 0.4147 | 1.6444 | 0.4198 | 1.4997 | 0.0631 | 1.8535 | 0.3558 | 0.5395 | 0.7438 | 1.4191 | 0.5283 | 0.9036 | 0.8816 | 1.9055 | 0.3424 | 1.3428 | 0.2852 | 1.3804 | 0.5494 | 0.7047 | 0.9937 | 1.4060 | 0.5354 | 0.3499 | 0.3183 |
| 577 | Q9UFN0 | Protein NipSnap homolog 3A (NipSnap3A) (Protein NipSnap homolog 4) (NipSnap4) (Target for Salmonella secreted protein C) (TassC) | NIPSNAP3A NIPSNAP4 HSPC299 | 24 | 5 | 0.8017 | 0.0966 | 2.8314 | 0.2265 | 1.1066 | 0.9185 | 2.1281 | 0.0943 | 0.9638 | 0.7616 | 2.9376 | 0.0895 | 1.0280 | 0.5593 | 2.1281 | 0.0844 | 0.6310 | 0.0217 | 4.4055 | 0.0105 | 0.2333 | 0.0000 | 2.6792 | 0.0719 | 0.4613 | 0.0004 | 3.0200 | 0.0300 |
| 578 | Q9BPW8 | Protein NipSnap homolog 1 (NipSnap1) | NIPSNAP1 | 6 | 15 | 0.8954 | 0.1241 | 1.0280 | 0.9451 | 1.0186 | 0.3229 | 1.1803 | 0.9874 | 0.8091 | 0.0822 | 0.7798 | 0.2937 | 0.7870 | 0.1486 | 0.6855 | 0.0627 | 0.7870 | 0.0486 | 0.8395 | 0.1198 | 0.5395 | 0.0016 | 1.0471 | 0.9541 | 0.5754 | 0.0093 | 0.6427 | 0.0702 |
| 579 | Q9UMX5 | Neudesin (Cell immortalization-related protein 2) (Neuron-derived neurotrophic factor) (Protein GIG47) (Secreted protein of unknown function) (SPUF protein) | NENF CIR2 SPUF | 6 | 3 | 2.1878 | 0.2783 | 1.2023 | 0.3019 | 2.4889 | 0.2196 | 1.2134 | 0.2935 | 4.3652 | 0.0429 | 1.1695 | 0.3032 | 3.3419 | 0.1082 | 1.1482 | 0.3858 | 1.9409 | 0.3362 | 0.9204 | 0.2833 | 1.5417 | 0.2147 | 1.2589 | 0.2489 | 1.9953 | 0.4609 | 1.0471 | 0.6860 |
| 580 | Q8TD19 | Serine/threonine-protein kinase Nek9 (EC 2.7.11.1) (Nercc1 kinase) (Never in mitosis A-related kinase 9) (NimA-related protein kinase 9) (NimA-related kinase 8) (Nek8) | NEK9 KIAA1995 NEK8 NERCC | 17 | 3 | 0.8318 | 0.0983 | 1.8365 | 0.1743 | 0.6252 | 0.0657 | 1.4060 | 0.4897 | 0.6546 | 0.0688 | 1.7701 | 0.1556 | 0.8017 | 0.1225 | 1.4191 | 0.4698 | 0.9638 | 0.9444 | 1.5560 | 0.2831 | 0.4656 | 0.0016 | 1.3677 | 0.5093 | 0.4831 | 0.0105 | 1.3183 | 0.6065 |
| 581 | P19404 | NADH dehydrogenase [ubiquinone] flavoprotein 2, mitochondrial (EC 1.6.5.3) (EC 1.6.99.3) (NADH-ubiquinone oxidoreductase 24 kDa subunit) | NDUFV2 | 3 | 6 | 1.1169 | 0.7548 | 0.9727 | 0.6806 | 0.9817 | 0.9405 | 1.1695 | 0.4955 | 0.8710 | 0.7325 | 1.0093 | 0.8470 | 1.1695 | 0.6787 | 1.0471 | 0.5957 | 1.1588 | 0.6933 | 1.1066 | 0.6427 | 0.9908 | 0.9808 | 1.0000 | 0.9746 | 0.8954 | 0.7166 | 1.1066 | 0.7946 |
| 582 | P49821 | NADH dehydrogenase [ubiquinone] flavoprotein 1, mitochondrial (EC 1.6.5.3) (EC 1.6.99.3) (Complex I-51kD) (CI-51kD) (NADH dehydrogenase flavoprotein 1) (NADH-ubiquinone oxidoreductase 51 kDa subunit) | NDUFV1 UQOR1 | 3 | 13 | 1.5276 | 0.1658 | 0.9817 | 0.4739 | 1.6749 | 0.3821 | 1.4588 | 0.0686 | 1.0965 | 0.5296 | 0.4699 | 0.1908 | 1.9055 | 0.2041 | 0.8166 | 0.6915 | 1.1066 | 0.7882 | 0.9462 | 0.8725 | 0.9817 | 0.3477 | 0.6792 | 0.1633 | 1.1912 | 0.6107 | 1.4322 | 0.2217 |
| 583 | O00217 | NADH dehydrogenase [ubiquinone] iron-sulfur protein 8, mitochondrial (EC 1.6.5.3) (EC 1.6.99.3) (Complex I-23kD) (CI-23kD) (NADH-ubiquinone oxidoreductase 23 kDa subunit) (TYKY subunit) | NDUFS8 | 6 | 3 | 1.9055 | 0.3638 | 1.2706 | 0.7974 | 1.1376 | 0.9683 | 1.4191 | 0.5231 | 1.2706 | 0.5343 | 0.7870 | 0.8786 | 2.0137 | 0.2970 | 1.0864 | 0.8890 | 1.7539 | 0.1520 | 1.2942 | 0.6816 | 0.8318 | 0.6001 | 1.0000 | 0.8446 | 1.3552 | 0.8486 | 1.6749 | 0.3561 |
| 584 | O75251 | NADH dehydrogenase [ubiquinone] iron-sulfur protein 7, mitochondrial (EC 1.6.5.3) (EC 1.6.99.3) (Complex I-20kD) (CI-20kD) (NADH-ubiquinone oxidoreductase 20 kDa subunit) (PSST subunit) | NDUFS7 | 13 | 3 | 1.0093 | 0.7680 | 0.9908 | 0.9763 | 0.9120 | 0.4038 | 3.1623 | 0.4026 | 0.8551 | 0.5138 | 0.8954 | 0.8639 | 1.4322 | 0.0635 | 1.6904 | 0.6251 | 1.1803 | 0.2576 | 1.0568 | 0.9858 | 1.0765 | 0.5779 | 0.6081 | 0.7520 | 0.9120 | 0.9524 | 1.8880 | 0.5007 |
| 585 | O75489 | NADH dehydrogenase [ubiquinone] iron-sulfur protein 3, mitochondrial (EC 1.6.5.3) (EC 1.6.99.3) (Complex I-30kD) (CI-30kD) (NADH-ubiquinone oxidoreductase 30 kDa subunit) | NDUFS3 | 3 | 10 | 1.0093 | 0.9236 | 1.5136 | 0.2029 | 1.0093 | 0.6986 | 1.2134 | 0.2386 | 1.0000 | 0.9570 | 0.8017 | 0.9894 | 1.2134 | 0.4317 | 0.9817 | 0.7399 | 0.9908 | 0.9260 | 0.9120 | 0.8068 | 0.9817 | 0.9977 | 0.7112 | 0.4751 | 1.1272 | 0.4393 | 0.8241 | 0.8195 |
| 586 | O75306 | NADH dehydrogenase [ubiquinone] iron-sulfur protein 2, mitochondrial (EC 1.6.5.3) (EC 1.6.99.3) (Complex I-49kD) (CI-49kD) (NADH-ubiquinone oxidoreductase 49 kDa subunit) | NDUFS2 | 5 | 6 | 2.1878 | 0.1628 | 2.9107 | 0.0402 | 1.7378 | 0.5331 | 2.8314 | 0.0821 | 1.4723 | 0.4171 | 1.9588 | 0.1511 | 3.0479 | 0.1682 | 1.4588 | 0.9786 | 1.2942 | 0.3629 | 1.4588 | 0.6517 | 1.0666 | 0.9360 | 1.1169 | 0.9166 | 3.3113 | 0.2548 | 2.3768 | 0.0964 |
| 587 | P28331 | NADH-ubiquinone oxidoreductase 75 kDa subunit, mitochondrial (EC 1.6.5.3) (EC 1.6.99.3) (Complex I-75kD) (CI-75kD) | NDUFS1 | 10 | 20 | 2.3335 | 0.1302 | 1.1803 | 0.9150 | 1.4723 | 0.9548 | 2.4889 | 0.1024 | 1.2474 | 0.7582 | 1.2823 | 0.9745 | 1.6596 | 0.3919 | 1.1376 | 0.8591 | 2.0512 | 0.1409 | 1.5276 | 0.4992 | 1.3062 | 0.8039 | 1.0471 | 0.7910 | 2.6062 | 0.0131 | 1.3932 | 0.2759 |
| 588 | Q9Y6M9 | NADH dehydrogenase [ubiquinone] 1 beta subcomplex subunit 9 (Complex I-B22) (CI-B22) (LYR motif-containing protein 3) (NADH-ubiquinone oxidoreductase B22 subunit) | NDUFB9 LYRM3 UQOR22 | 13 | 4 | 1.4454 | 0.3422 | 1.0568 | 0.8672 | 0.9376 | 0.7417 | 1.5704 | 0.3434 | 0.7447 | 0.4873 | 1.0000 | 0.9249 | 0.7112 | 0.5950 | 0.4246 | 0.5634 | 0.6081 | 0.3482 | 0.8395 | 0.7953 | 1.1803 | 0.8772 | 0.6918 | 0.5959 | 1.2823 | 0.7579 | 0.9376 | 0.5929 |
| 589 | O14561 | Acyl carrier protein, mitochondrial (ACP) (CI-SDAP) (NADH-ubiquinone oxidoreductase 9.6 kDa subunit) | NDUFAB1 | 21 | 7 | 1.7539 | 0.1207 | 1.2589 | 0.3401 | 2.0324 | 0.0417 | 1.4454 | 0.5519 | 1.1169 | 0.8307 | 1.1066 | 0.8808 | 2.6792 | 0.0048 | 1.0186 | 0.9865 | 1.3428 | 0.3725 | 1.3932 | 0.4393 | 1.6293 | 0.3894 | 1.1272 | 0.5524 | 2.3121 | 0.1317 | 0.5297 | 0.4823 |
| 590 | Q16795 | NADH dehydrogenase [ubiquinone] 1 alpha subcomplex subunit 9, mitochondrial (Complex I-39kD) (CI-39kD) (NADH-ubiquinone oxidoreductase 39 kDa subunit) | NDUFA9 NDUFS2L | 6 | 7 | 1.7378 | 0.7981 | 0.7178 | 0.8835 | 1.1695 | 0.5627 | 1.4191 | 0.1577 | 0.3342 | 0.0082 | 0.9204 | 0.9924 | 1.3428 | 0.8162 | 1.0471 | 0.8649 | 1.0568 | 0.6865 | 0.9290 | 0.9165 | 1.0568 | 0.9640 | 0.6194 | 0.3766 | 1.5136 | 0.9065 | 1.7219 | 0.2612 |
| 591 | Q16718 | NADH dehydrogenase [ubiquinone] 1 alpha subcomplex subunit 5 (Complex I subunit B13) (Complex I-13kD-B) (CI-13kD-B) (NADH-ubiquinone oxidoreductase 13 kDa-B subunit) | NDUFA5 | 7 | 4 | 1.0568 | 0.8125 | 1.2134 | 0.1710 | 0.8790 | 0.6884 | 1.1912 | 0.1275 | 0.5495 | 0.6245 | 1.0375 | 0.6814 | 1.3804 | 0.4066 | 1.0186 | 0.9337 | 1.0471 | 0.4912 | 1.0375 | 0.7695 | 0.4446 | 0.2206 | 1.1169 | 0.4284 | 0.6310 | 0.3578 | 1.2474 | 0.4157 |
| 592 | O00483 | Cytochrome c oxidase subunit NDUFA4 (Complex I-MLRQ) (CI-MLRQ) (NADH-ubiquinone oxidoreductase MLRQ subunit) | NDUFA4 | 8 | 4 | 1.1588 | 0.8071 | 0.6730 | 0.0866 | 0.9036 | 0.9727 | 1.4723 | 0.6758 | 0.9550 | 0.8195 | 0.4831 | 0.0478 | 1.1803 | 0.6705 | 0.9462 | 0.3145 | 0.5649 | 0.3100 | 1.5996 | 0.6275 | 0.4699 | 0.1259 | 1.2246 | 0.2511 | 0.8630 | 0.7689 | 2.1086 | 0.3131 |
| 593 | O43678 | NADH dehydrogenase [ubiquinone] 1 alpha subcomplex subunit 2 (Complex I-B8) (CI-B8) (NADH-ubiquinone oxidoreductase B8 subunit) | NDUFA2 | 7 | 3 | 0.8790 | 0.6224 | 1.4454 | 0.4854 | 0.8954 | 0.2116 | 0.9727 | 0.9449 | 0.9462 | 0.8999 | 0.5754 | 0.4304 | 1.0280 | 0.7323 | 0.5546 | 0.4621 | 0.8551 | 0.5592 | 1.1912 | 0.7204 | 1.0093 | 0.8608 | 0.2535 | 0.3332 | 0.8954 | 0.7288 | 0.8954 | 0.8745 |
| 594 | Q9P0J0 | NADH dehydrogenase [ubiquinone] 1 alpha subcomplex subunit 13 (Cell death regulatory protein GRIM-19) (Complex I-B16.6) (CI-B16.6) (Gene associated with retinoic and interferon-induced mortality 19 protein) (GRIM-19) (Gene associated with retinoic and IFN-induced mortality 19 protein) (NADH-ubiquinone oxidoreductase B16.6 subunit) | NDUFA13 GRIM19 CDA016 CGI-39 | 6 | 3 | 0.4966 | 0.1532 | 1.4723 | 0.3255 | 0.8318 | 0.7091 | 1.6293 | 0.2890 | 0.5058 | 0.0593 | 1.2942 | 0.8399 | 1.0765 | 0.8149 | 0.3373 | 0.3647 | 0.5649 | 0.1077 | 0.3631 | 0.2740 | 0.4875 | 0.2212 | 0.8551 | 0.9344 | 0.6026 | 0.2854 | 1.3062 | 0.8007 |
| 595 | O95299 | NADH dehydrogenase [ubiquinone] 1 alpha subcomplex subunit 10, mitochondrial (Complex I-42kD) (CI-42kD) (NADH-ubiquinone oxidoreductase 42 kDa subunit) | NDUFA10 | 5 | 6 | 1.4191 | 0.3205 | 1.2706 | 0.6725 | 1.7701 | 0.1640 | 1.0280 | 0.5415 | 1.4454 | 0.3599 | 1.2474 | 0.7051 | 1.7378 | 0.1322 | 0.8318 | 0.1891 | 1.5849 | 0.2147 | 0.9638 | 0.5834 | 1.5996 | 0.2002 | 0.8954 | 0.2827 | 1.7539 | 0.1603 | 1.3183 | 0.4816 |
| 596 | Q9UN36 | Protein NDRG2 (N-myc downstream-regulated gene 2 protein) (Protein Syld709613) | NDRG2 KIAA1248 SYLD | 5 | 15 | 1.3677 | 0.9869 | 0.5970 | 0.3064 | 1.3428 | 0.8810 | 0.6081 | 0.3184 | 0.9120 | 0.3848 | 0.5546 | 0.0254 | 1.6293 | 0.4968 | 0.5970 | 0.1022 | 1.1066 | 0.6018 | 1.0280 | 0.7506 | 1.1376 | 0.6269 | 0.5012 | 0.1439 | 1.7219 | 0.6537 | 0.5598 | 0.1960 |
| 597 | Q92597 | Protein NDRG1 (Differentiation-related gene 1 protein) (DRG-1) (N-myc downstream-regulated gene 1 protein) (Nickel-specific induction protein Cap43) (Reducing agents and tunicamycin-responsive protein) (RTP) (Rit42) | NDRG1 CAP43 DRG1 RTP | 9 | 3 | 0.7656 | 0.7944 | 0.6138 | 0.6646 | 0.6368 | 0.4915 | 0.8318 | 0.0335 | 1.1588 | 0.1930 | 1.1588 | 0.4260 | 1.2823 | 0.2663 | 0.5495 | 0.0082 | 0.6982 | 0.6767 | 1.4723 | 0.2062 | 0.7870 | 0.9953 | 0.8790 | 0.4630 | 0.8395 | 0.8923 | 0.9462 | 0.4848 |
| 598 | Q969V3 | Nicalin (Nicastrin-like protein) | NCLN | 16 | 6 | 0.4571 | 0.0070 | 0.9204 | 0.8068 | 0.4406 | 0.0348 | 0.9204 | 0.7947 | 0.8790 | 0.1379 | 0.7178 | 0.8465 | 0.4571 | 0.0392 | 1.0000 | 0.5912 | 0.9204 | 0.2573 | 0.9908 | 0.8088 | 0.3664 | 0.0053 | 0.9290 | 0.9929 | 0.1803 | 0.0017 | 0.8872 | 0.7410 |
| 599 | P19338 | Nucleolin (Protein C23) | NCL | 4 | 21 | 8.7096 | 0.0025 | 1.4723 | 0.1251 | 0.2291 | 0.0041 | 1.4997 | 0.2946 | 1.4588 | 0.0033 | 2.7542 | 0.0010 | 0.3020 | 0.0099 | 1.8707 | 0.0062 | 11.3763 | 0.0027 | 2.4434 | 0.0027 | 2.6546 | 0.1196 | 2.2699 | 0.0026 | 6.1944 | 0.0028 | 1.8535 | 0.0803 |
| 600 | Q9UHE5 | N-acetyltransferase 8 (EC 2.3.1.-) (Acetyltransferase 2) (ATase2) (Camello-like protein 1) (Cysteinyl-conjugate N-acetyltransferase) (CCNAT) (EC 2.3.1.80) | NAT8 CML1 GLA TSC501 | 6 | 3 | 1.2023 | 0.2233 | 1.6444 | 0.3059 | 0.9908 | 0.9922 | 1.2359 | 0.5873 | 1.0765 | 0.5261 | 1.2474 | 0.6491 | 1.0471 | 0.9431 | 0.9727 | 0.2874 | 1.0471 | 0.5446 | 0.8395 | 0.6258 | 1.1482 | 0.3790 | 1.2359 | 0.9066 | 1.0864 | 0.8300 | 0.4406 | 0.1944 |
| 601 | O43776 | Asparagine--tRNA ligase, cytoplasmic (EC 6.1.1.22) (Asparaginyl-tRNA synthetase) (AsnRS) | NARS | 19 | 7 | 2.2284 | 0.1349 | 0.8954 | 0.5707 | 1.5996 | 0.3087 | 0.8954 | 0.7930 | 1.8535 | 0.1334 | 1.1272 | 0.6549 | 1.6293 | 0.1336 | 1.2942 | 0.1988 | 2.5119 | 0.1110 | 1.3552 | 0.1815 | 4.0926 | 0.0302 | 1.2134 | 0.7403 | 1.6293 | 0.2215 | 1.8880 | 0.0569 |
| 602 | Q6XQN6 | Nicotinate phosphoribosyltransferase (NAPRTase) (EC 6.3.4.21) (FHA-HIT-interacting protein) (Nicotinate phosphoribosyltransferase domain-containing protein 1) | NAPRT FHIP NAPRT1 | 3 | 34 | 1.6596 | 0.4327 | 0.1585 | 0.0002 | 0.8954 | 0.8997 | 1.6904 | 0.0924 | 1.3932 | 0.5184 | 0.8551 | 0.2102 | 1.4454 | 0.5100 | 0.6982 | 0.1016 | 1.5136 | 0.4674 | 0.8017 | 0.0213 | 1.3305 | 0.6033 | 0.8091 | 0.4799 | 1.4060 | 0.5290 | 0.5702 | 0.0331 |
| 603 | P54920 | Alpha-soluble NSF attachment protein (SNAP-alpha) (N-ethylmaleimide-sensitive factor attachment protein alpha) | NAPA SNAPA | 5 | 9 | 1.2359 | 0.4857 | 0.8872 | 0.7724 | 0.8017 | 0.6575 | 0.9550 | 0.9254 | 1.4859 | 0.3046 | 1.5136 | 0.0630 | 1.0864 | 0.6764 | 1.3804 | 0.1033 | 1.3428 | 0.4142 | 1.0375 | 0.9130 | 1.9953 | 0.0441 | 1.0000 | 0.9297 | 1.9588 | 0.0540 | 0.9908 | 0.9050 |
| 604 | Q99733 | Nucleosome assembly protein 1-like 4 (Nucleosome assembly protein 2) (NAP-2) | NAP1L4 NAP2 | 33 | 8 | 0.7798 | 0.3017 | 1.0471 | 0.5785 | 0.8790 | 0.4372 | 0.9817 | 0.8644 | 0.9036 | 0.6978 | 0.9638 | 0.8415 | 0.6138 | 0.0771 | 1.0471 | 0.5301 | 1.3932 | 0.7399 | 0.9908 | 0.8972 | 0.1271 | 0.0023 | 1.0280 | 0.6420 | 0.3767 | 0.0289 | 1.0864 | 0.5173 |
| 605 | Q9NR45 | Sialic acid synthase (N-acetylneuraminate synthase) (EC 2.5.1.56) (N-acetylneuraminate-9-phosphate synthase) (EC 2.5.1.57) (N-acetylneuraminic acid phosphate synthase) (N-acetylneuraminic acid synthase) | NANS SAS | 14 | 6 | 0.8318 | 0.8204 | 0.5297 | 0.2285 | 1.2823 | 0.8586 | 0.6607 | 0.1396 | 1.4588 | 0.1969 | 0.7112 | 0.5364 | 1.1272 | 0.4325 | 0.8241 | 0.8588 | 1.2706 | 0.4992 | 0.4325 | 0.0457 | 1.7219 | 0.0854 | 0.9908 | 0.8271 | 1.2023 | 0.4106 | 0.6368 | 0.0718 |
| 606 | P43490 | Nicotinamide phosphoribosyltransferase (NAmPRTase) (Nampt) (EC 2.4.2.12) (Pre-B-cell colony-enhancing factor 1) (Pre-B cell-enhancing factor) (Visfatin) | NAMPT PBEF PBEF1 | 7 | 13 | 1.4723 | 0.2796 | 0.3981 | 0.0007 | 1.3804 | 0.3268 | 0.6252 | 0.0316 | 0.5297 | 0.7397 | 0.7516 | 0.4006 | 1.3428 | 0.3069 | 0.2168 | 0.0000 | 1.3552 | 0.3309 | 0.4406 | 0.0139 | 1.2359 | 0.6758 | 0.5970 | 0.1100 | 1.7701 | 0.1233 | 0.4130 | 0.0095 |
| 607 | P54802 | Alpha-N-acetylglucosaminidase (EC 3.2.1.50) (N-acetyl-alpha-glucosaminidase) (NAG) [Cleaved into: Alpha-N-acetylglucosaminidase 82 kDa form; Alpha-N-acetylglucosaminidase 77 kDa form] | NAGLU UFHSD1 | 5 | 4 | 1.2942 | 0.2263 | 0.4742 | 0.5338 | 0.9462 | 0.7453 | 0.2965 | 0.5046 | 1.3305 | 0.3284 | 0.7178 | 0.2578 | 0.7112 | 0.4273 | 0.3251 | 0.3406 | 0.5105 | 0.4516 | 0.6427 | 0.0940 | 1.1169 | 0.7054 | 0.6730 | 0.2349 | 0.8872 | 0.9535 | 0.3404 | 0.3290 |
| 608 | Q9UJ70 | N-acetyl-D-glucosamine kinase (N-acetylglucosamine kinase) (EC 2.7.1.59) (GlcNAc kinase) | NAGK | 12 | 3 | 0.4487 | 0.0024 | 0.4786 | 0.2383 | 0.8472 | 0.3107 | 1.2134 | 0.8814 | 0.4246 | 0.0021 | 1.2359 | 0.4384 | 1.5849 | 0.0071 | 0.3076 | 0.2646 | 0.4018 | 0.0006 | 1.5849 | 0.7381 | 0.5152 | 0.0055 | 0.6138 | 0.6134 | 0.5105 | 0.0008 | 1.4454 | 0.6198 |
| 609 | Q4G0N4 | NAD kinase 2, mitochondrial (EC 2.7.1.23) (Mitochondrial NAD kinase) (NAD kinase domain-containing protein 1, mitochondrial) | NADK2 C5orf33 MNADK NADKD1 | 4 | 23 | 1.4859 | 0.7224 | 0.8166 | 0.6586 | 0.3767 | 0.4844 | 1.0186 | 0.9791 | 1.6144 | 0.2314 | 0.7943 | 0.6326 | 0.9550 | 0.9614 | 0.7379 | 0.5555 | 1.6749 | 0.4958 | 0.8318 | 0.7084 | 2.0512 | 0.3255 | 0.7727 | 0.6347 | 2.2699 | 0.4065 | 0.7656 | 0.5938 |
| 610 | E9PAV3 | Nascent polypeptide-associated complex subunit alpha, muscle-specific form (Alpha-NAC, muscle-specific form) (skNAC) | NACA | 28 | 4 | 1.8030 | 0.3248 | 1.2134 | 0.6367 | 1.3183 | 0.5083 | 1.1912 | 0.6623 | 1.2134 | 0.6139 | 1.8197 | 0.3364 | 1.3552 | 0.9625 | 0.9727 | 0.5278 | 1.2823 | 0.4595 | 1.3932 | 0.4232 | 0.3133 | 0.0161 | 1.0186 | 0.9135 | 0.9290 | 0.4479 | 1.3932 | 0.4464 |
| 611 | O00159 | Unconventional myosin-Ic (Myosin I beta) (MMI-beta) (MMIb) | MYO1C | 22 | 18 | 0.9908 | 0.7485 | 1.0280 | 0.7232 | 1.0093 | 0.7300 | 1.0965 | 0.5602 | 0.8954 | 0.2966 | 1.0093 | 0.7034 | 0.8166 | 0.4116 | 1.0864 | 0.9562 | 0.8395 | 0.1933 | 0.9817 | 0.7987 | 0.8551 | 0.3049 | 0.9908 | 0.6180 | 0.7516 | 0.1988 | 1.0280 | 0.6072 |
| 612 | O43795 | Unconventional myosin-Ib (MYH-1c) (Myosin I alpha) (MMI-alpha) (MMIa) | MYO1B | 5 | 14 | 3.8019 | 0.3139 | 1.4997 | 0.2409 | 2.6303 | 0.4618 | 1.5136 | 0.2418 | 3.5318 | 0.2648 | 1.7219 | 0.0452 | 3.4356 | 0.3725 | 0.9120 | 0.9407 | 2.5351 | 0.4961 | 1.1376 | 0.3917 | 5.7544 | 0.1524 | 1.5136 | 0.2066 | 1.5417 | 0.7977 | 0.9376 | 0.7652 |
| 613 | Q92614 | Unconventional myosin-XVIIIa (Molecule associated with JAK3 N-terminus) (MAJN) (Myosin containing a PDZ domain) | MYO18A KIAA0216 MYSPDZ | 16 | 7 | 1.2134 | 0.6387 | 1.1272 | 0.3889 | 1.1482 | 0.8699 | 1.1695 | 0.2606 | 1.4191 | 0.4951 | 1.1272 | 0.1011 | 0.8954 | 0.4680 | 1.0186 | 0.8685 | 1.5996 | 0.5187 | 0.9376 | 0.9557 | 1.3552 | 0.4867 | 1.0186 | 0.7457 | 1.6144 | 0.2962 | 1.1482 | 0.5245 |
| 614 | Q15746 | Myosin light chain kinase, smooth muscle (MLCK) (smMLCK) (EC 2.7.11.18) (Kinase-related protein) (KRP) (Telokin) [Cleaved into: Myosin light chain kinase, smooth muscle, deglutamylated form] | MYLK MLCK MLCK1 MYLK1 | 27 | 10 | 2.0324 | 0.0006 | 0.5395 | 0.2613 | 1.1066 | 0.9912 | 1.2134 | 0.1428 | 1.5276 | 0.1402 | 0.8472 | 0.9850 | 1.6904 | 0.0172 | 0.9120 | 0.5856 | 2.2909 | 0.0000 | 0.7311 | 0.5178 | 0.7379 | 0.6522 | 0.5916 | 0.0749 | 0.7870 | 0.5493 | 0.7047 | 0.6073 |
| 615 | P24844 | Myosin regulatory light polypeptide 9 (20 kDa myosin light chain) (LC20) (MLC-2C) (Myosin RLC) (Myosin regulatory light chain 2, smooth muscle isoform) (Myosin regulatory light chain 9) (Myosin regulatory light chain MRLC1) | MYL9 MLC2 MRLC1 MYRL2 | 8 | 9 | 2.8576 | 0.0771 | 0.8790 | 0.7607 | 2.5351 | 0.1012 | 1.9770 | 0.1514 | 1.4454 | 0.4896 | 0.9290 | 0.7785 | 2.5351 | 0.0353 | 1.0568 | 0.9423 | 2.0701 | 0.1472 | 1.0666 | 0.6811 | 2.2491 | 0.1705 | 0.7870 | 0.5190 | 2.0324 | 0.1520 | 0.8630 | 0.5270 |
| 616 | P19105 | Myosin regulatory light chain 12A (Epididymis secretory protein Li 24) (HEL-S-24) (MLC-2B) (Myosin RLC) (Myosin regulatory light chain 2, nonsarcomeric) (Myosin regulatory light chain MRLC3) | MYL12A MLCB MRLC3 RLC | 25 | 14 |  |  | 2.3988 | 0.1482 |  |  | 2.6546 | 0.1227 |  |  | 1.8707 | 0.3820 |  |  | 1.2134 | 0.5272 |  |  | 2.2284 | 0.2089 |  |  | 1.9953 | 0.2930 |  |  | 1.6596 | 0.4059 |
| 617 | P35579 | Myosin-9 (Cellular myosin heavy chain, type A) (Myosin heavy chain 9) (Myosin heavy chain, non-muscle IIa) (Non-muscle myosin heavy chain A) (NMMHC-A) (Non-muscle myosin heavy chain IIa) (NMMHC II-a) (NMMHC-IIA) | MYH9 | 24 | 151 |  |  | 0.7047 | 0.0115 |  |  | 1.5417 | 0.0165 |  |  | 1.2589 | 0.3219 |  |  | 0.6310 | 0.0048 |  |  | 1.1169 | 0.6363 |  |  | 0.7516 | 0.0109 |  |  | 1.5996 | 0.0000 |
| 618 | Q7Z406 | Myosin-14 (Myosin heavy chain 14) (Myosin heavy chain, non-muscle IIc) (Non-muscle myosin heavy chain IIc) (NMHC II-C) | MYH14 KIAA2034 FP17425 | 16 | 42 | 1.1066 | 0.9601 | 0.6918 | 0.4027 | 2.8576 | 0.0648 | 0.8318 | 0.7848 | 1.7219 | 0.3487 | 0.9550 | 0.6087 | 1.1803 | 0.5054 | 0.9290 | 0.4078 | 2.2080 | 0.4130 | 0.8318 | 0.8605 | 3.5318 | 0.0944 | 1.4060 | 0.0190 | 1.6749 | 0.2791 | 1.7378 | 0.0008 |
| 619 | P35749 | Myosin-11 (Myosin heavy chain 11) (Myosin heavy chain, smooth muscle isoform) (SMMHC) | MYH11 KIAA0866 | 167 | 34 | 0.4656 | 0.0000 | 0.1432 | 0.0587 | 1.4588 | 0.2083 | 2.0137 | 0.0340 | 0.8017 | 0.3948 | 0.2051 | 0.0746 | 0.9550 | 0.2707 | 2.0137 | 0.0128 | 1.1588 | 0.9028 | 0.4742 | 0.0101 | 2.2699 | 0.0000 | 0.2729 | 0.0716 | 1.4997 | 0.2737 | 0.2355 | 0.0003 |
| 620 | P35580 | Myosin-10 (Cellular myosin heavy chain, type B) (Myosin heavy chain 10) (Myosin heavy chain, non-muscle IIb) (Non-muscle myosin heavy chain B) (NMMHC-B) (Non-muscle myosin heavy chain IIb) (NMMHC II-b) (NMMHC-IIB) | MYH10 | 34 | 48 | 0.6427 | 0.7142 | 1.2589 | 0.3446 | 1.5849 | 0.0020 | 2.9648 | 0.0000 | 0.6668 | 0.6690 | 0.8790 | 0.4502 | 0.8710 | 0.8667 | 1.1272 | 0.3682 | 0.9204 | 0.6594 | 1.0000 | 0.7654 | 0.8710 | 0.8262 | 0.7379 | 0.1455 | 1.1272 | 0.4869 | 1.3062 | 0.0159 |
| 621 | Q14764 | Major vault protein (MVP) (Lung resistance-related protein) | MVP LRP | 32 | 15 | 0.9120 | 0.9845 | 1.0568 | 0.9305 | 1.1482 | 0.4743 | 0.8472 | 0.9342 | 1.2474 | 0.5645 | 1.3677 | 0.8091 | 0.8710 | 0.7237 | 1.2246 | 0.6693 | 0.6310 | 0.5184 | 2.0137 | 0.1405 | 0.5598 | 0.3771 | 1.4723 | 0.3260 | 0.6427 | 0.5218 | 3.4041 | 0.0029 |
| 622 | Q03426 | Mevalonate kinase (MK) (EC 2.7.1.36) | MVK | 55 | 5 | 0.3908 | 0.0034 | 0.5495 | 0.2738 | 2.2080 | 0.0291 | 2.7290 | 0.4892 | 0.7656 | 0.0098 | 2.8576 | 0.4110 | 1.0000 | 0.1189 | 5.2000 | 0.0688 | 2.0137 | 0.0104 | 3.0479 | 0.3752 | 1.2942 | 0.7091 | 3.5975 | 0.1666 | 1.3677 | 0.7578 | 1.3062 | 0.9175 |
| 623 | P53602 | Diphosphomevalonate decarboxylase (EC 4.1.1.33) (Mevalonate (diphospho)decarboxylase) (MDDase) (Mevalonate pyrophosphate decarboxylase) | MVD MPD | 16 | 10 | 3.3113 | 0.0042 | 1.0280 | 0.9853 | 1.8707 | 0.2831 | 0.8630 | 0.9110 | 1.0000 | 0.7427 | 1.5276 | 0.4369 | 2.4434 | 0.1778 | 1.4191 | 0.8159 | 1.4859 | 0.4313 | 2.2080 | 0.0389 | 3.9446 | 0.0016 | 1.8707 | 0.3138 | 2.4434 | 0.0309 | 0.9908 | 0.9677 |
| 624 | P22033 | Methylmalonyl-CoA mutase, mitochondrial (MCM) (EC 5.4.99.2) (Methylmalonyl-CoA isomerase) | MUT | 6 | 35 | 5.8614 | 0.2703 | 0.4571 | 0.0260 | 4.4463 | 0.3119 | 1.1272 | 0.7861 | 5.5976 | 0.3024 | 0.9120 | 0.5367 | 2.8314 | 0.4733 | 0.9376 | 0.9141 | 3.7670 | 0.3005 | 0.6607 | 0.1208 | 0.6918 | 0.5492 | 0.9817 | 0.7830 | 4.0179 | 0.3260 | 0.2188 | 0.0003 |
| 625 | P55157 | Microsomal triglyceride transfer protein large subunit | MTTP MTP | 6 | 52 | 1.0000 | 0.9737 | 1.7219 | 0.2176 | 1.0666 | 0.6974 | 2.0893 | 0.1282 | 1.1482 | 0.3018 | 1.0864 | 0.4343 | 0.9376 | 0.5469 | 0.8318 | 0.1368 | 0.8551 | 0.7408 | 1.1066 | 0.1973 | 0.3908 | 0.0799 | 0.8872 | 0.0789 | 1.2942 | 0.6293 | 0.5445 | 0.0036 |
| 626 | P11586 | C-1-tetrahydrofolate synthase, cytoplasmic (C1-THF synthase) [Cleaved into: C-1-tetrahydrofolate synthase, cytoplasmic, N-terminally processed] [Includes: Methylenetetrahydrofolate dehydrogenase (EC 1.5.1.5); Methenyltetrahydrofolate cyclohydrolase (EC 3.5.4.9); Formyltetrahydrofolate synthetase (EC 6.3.4.3)] | MTHFD1 MTHFC MTHFD | 35 | 109 | 1.3804 | 0.4314 | 1.5560 | 0.2012 | 1.3677 | 0.4284 | 2.6792 | 0.0003 | 0.8872 | 0.1499 | 2.3550 | 0.0013 | 1.3305 | 0.2918 | 1.5136 | 0.1584 | 1.1482 | 0.4508 | 2.0324 | 0.0373 | 0.2032 | 0.0000 | 2.6546 | 0.0000 | 0.5546 | 0.0001 | 0.7379 | 0.1995 |
| 627 | Q86UE4 | Protein LYRIC (3D3/LYRIC) (Astrocyte elevated gene-1 protein) (AEG-1) (Lysine-rich CEACAM1 co-isolated protein) (Metadherin) (Metastasis adhesion protein) | MTDH AEG1 LYRIC | 69 | 9 | 1.1803 | 0.9709 | 1.7378 | 0.2173 | 1.4859 | 0.3153 | 0.8472 | 0.5074 | 0.9462 | 0.5919 | 2.1478 | 0.0917 | 1.4588 | 0.2165 | 2.1281 | 0.1469 | 2.0512 | 0.0068 | 2.7290 | 0.0707 | 0.3733 | 0.0000 | 2.7797 | 0.1014 | 0.8166 | 0.0076 | 1.9588 | 0.4480 |
| 628 | P00403 | Cytochrome c oxidase subunit 2 (Cytochrome c oxidase polypeptide II) | MT-CO2 COII COXII MTCO2 | 113 | 8 | 1.7865 | 0.0083 | 2.1478 | 0.3196 | 1.8880 | 0.0007 | 2.4434 | 0.2657 | 1.9588 | 0.0000 | 0.8318 | 0.7779 | 2.3768 | 0.0000 | 2.6792 | 0.3569 | 1.2942 | 0.5651 | 1.2823 | 0.5626 | 0.1191 | 0.0000 | 2.7040 | 0.2661 | 0.4831 | 0.0000 | 2.3335 | 0.1549 |
| 629 | Q9Y6C9 | Mitochondrial carrier homolog 2 (Met-induced mitochondrial protein) | MTCH2 MIMP HSPC032 | 7 | 13 | 1.7378 | 0.1888 | 1.5704 | 0.3821 | 1.4322 | 0.1837 | 1.2706 | 0.8749 | 2.0701 | 0.1206 | 0.6730 | 0.3173 | 1.4997 | 0.2119 | 1.1066 | 0.9967 | 0.6918 | 0.6984 | 1.0280 | 0.6690 | 1.5560 | 0.2070 | 0.7586 | 0.7811 | 2.0137 | 0.0842 | 1.1695 | 0.4054 |
| 630 | Q9UJ68 | Mitochondrial peptide methionine sulfoxide reductase (EC 1.8.4.11) (Peptide-methionine (S)-S-oxide reductase) (Peptide Met(O) reductase) (Protein-methionine-S-oxide reductase) (PMSR) | MSRA | 10 | 5 | 2.5119 | 0.0805 | 1.3062 | 0.9806 | 1.8535 | 0.0694 | 1.5560 | 0.5346 | 1.7378 | 0.1760 | 1.2589 | 0.8200 | 1.9409 | 0.3427 | 1.0864 | 0.8800 | 1.4859 | 0.9639 | 1.3932 | 0.5689 | 1.5849 | 0.4372 | 1.4723 | 0.7689 | 1.4859 | 0.5174 | 0.4699 | 0.3723 |
| 631 | P26038 | Moesin (Membrane-organizing extension spike protein) | MSN | 14 | 35 | 1.5560 | 0.6074 | 0.8395 | 0.6356 | 1.1169 | 0.9508 | 0.6668 | 0.2217 | 0.5297 | 0.4605 | 1.3932 | 0.0969 | 1.0666 | 0.9301 | 1.0864 | 0.6845 | 1.4997 | 0.6171 | 1.3932 | 0.0269 | 0.4285 | 0.1969 | 0.9204 | 0.8214 | 1.1912 | 0.8492 | 1.7061 | 0.0034 |
| 632 | Q9BV20 | Methylthioribose-1-phosphate isomerase (M1Pi) (MTR-1-P isomerase) (EC 5.3.1.23) (Mediator of RhoA-dependent invasion) (S-methyl-5-thioribose-1-phosphate isomerase) (Translation initiation factor eIF-2B subunit alpha/beta/delta-like protein) | MRI1 MRDI UNQ6390/PRO21135 | 3 | 4 | 0.4875 | 0.4942 | 0.3467 | 0.4042 | 0.7586 | 0.3547 | 0.4920 | 0.0427 | 0.6855 | 0.3979 | 0.7870 | 0.5659 | 0.7798 | 0.6801 | 0.4831 | 0.1681 | 0.5861 | 0.1290 | 0.2754 | 0.3290 | 0.2489 | 0.0747 | 0.7447 | 0.6388 | 0.5105 | 0.3368 | 0.7112 | 0.4757 |
| 633 | P25325 | 3-mercaptopyruvate sulfurtransferase (MST) (EC 2.8.1.2) | MPST TST2 | 41 | 32 | 0.6792 | 0.2355 | 1.0765 | 0.9381 | 1.4322 | 0.0509 | 1.4322 | 0.9896 | 0.9204 | 0.6475 | 0.7727 | 0.0640 | 1.2706 | 0.2559 | 0.8710 | 0.1743 | 0.7870 | 0.3430 | 0.6668 | 0.0780 | 2.4210 | 0.0000 | 1.5417 | 0.3438 | 1.5276 | 0.0044 | 0.3532 | 0.0047 |
| 634 | P05164 | Myeloperoxidase (MPO) (EC 1.11.2.2) [Cleaved into: Myeloperoxidase; 89 kDa myeloperoxidase; 84 kDa myeloperoxidase; Myeloperoxidase light chain; Myeloperoxidase heavy chain] | MPO | 5 | 9 | 1.3677 | 0.3641 | 0.4966 | 0.1293 | 0.9462 | 0.8354 | 0.3837 | 0.0017 | 1.0666 | 0.9245 | 0.3945 | 0.0092 | 0.8091 | 0.5020 | 0.4169 | 0.0502 | 1.1376 | 0.7340 | 0.4055 | 0.0198 | 1.0093 | 0.8635 | 0.2377 | 0.0016 | 0.8630 | 0.5784 | 0.5495 | 0.0654 |
| 635 | Q969Z3 | Mitochondrial amidoxime reducing component 2 (mARC2) (EC 1.-.-.-) (Molybdenum cofactor sulfurase C-terminal domain-containing protein 2) (MOSC domain-containing protein 2) (Moco sulfurase C-terminal domain-containing protein 2) | MARC2 MOSC2 | 28 | 15 | 0.9376 | 0.1795 | 1.0000 | 0.9649 | 0.8790 | 0.4872 | 1.0666 | 0.9622 | 0.7447 | 0.0725 | 0.9638 | 0.4965 | 0.9638 | 0.7210 | 0.5058 | 0.0416 | 0.6368 | 0.0164 | 1.1066 | 0.3582 | 0.1820 | 0.0000 | 1.3062 | 0.8401 | 0.6427 | 0.0060 | 0.1629 | 0.0044 |
| 636 | Q5VT66 | Mitochondrial amidoxime-reducing component 1 (mARC1) (EC 1.-.-.-) (Molybdenum cofactor sulfurase C-terminal domain-containing protein 1) (MOSC domain-containing protein 1) (Moco sulfurase C-terminal domain-containing protein 1) | MARC1 MOSC1 | 9 | 6 | 0.3802 | 0.0482 | 0.2228 | 0.4835 | 0.4406 | 0.0198 | 0.5546 | 0.2892 | 0.3048 | 0.0085 | 0.8872 | 0.7934 | 0.2291 | 0.0066 | 1.2023 | 0.4372 | 0.6486 | 0.0405 | 0.6668 | 0.4878 | 1.9953 | 0.0048 | 1.2134 | 0.5642 | 0.9290 | 0.5705 | 0.3631 | 0.5691 |
| 637 | Q13724 | Mannosyl-oligosaccharide glucosidase (EC 3.2.1.106) (Processing A-glucosidase I) | MOGS GCS1 | 12 | 17 | 1.2706 | 0.4618 | 0.7586 | 0.3814 | 0.6982 | 0.1953 | 0.8710 | 0.6657 | 0.5546 | 0.1250 | 0.5200 | 0.2091 | 1.3062 | 0.6935 | 0.6486 | 0.3459 | 1.0666 | 0.7357 | 1.1695 | 0.9589 | 0.2128 | 0.0028 | 0.8954 | 0.4269 | 0.3908 | 0.0164 | 0.5970 | 0.1323 |
| 638 | Q96EY8 | Cob(I)yrinic acid a,c-diamide adenosyltransferase, mitochondrial (EC 2.5.1.17) (Cob(I)alamin adenosyltransferase) (Methylmalonic aciduria type B protein) | MMAB | 5 | 7 | 1.2823 | 0.4777 | 0.9036 | 0.7704 | 1.2589 | 0.4739 | 0.5754 | 0.2355 | 1.3183 | 0.8224 | 0.6792 | 0.2256 | 1.3062 | 0.5271 | 1.1482 | 0.2248 | 0.8551 | 0.5788 | 0.6546 | 0.5500 | 0.8091 | 0.5028 | 1.2706 | 0.2563 | 1.0186 | 0.9067 | 0.3873 | 0.1238 |
| 639 | O95822 | Malonyl-CoA decarboxylase, mitochondrial (MCD) (EC 4.1.1.9) | MLYCD | 11 | 5 | 1.0471 | 0.8241 | 1.2359 | 0.4481 | 1.0186 | 0.6666 | 1.6444 | 0.3850 | 1.0568 | 0.9154 | 0.7798 | 0.6361 | 0.8551 | 0.8151 | 1.2589 | 0.4637 | 1.4191 | 0.7148 | 1.6444 | 0.4133 | 0.4446 | 0.0104 | 0.9376 | 0.7265 | 1.0186 | 0.9149 | 1.3552 | 0.4669 |
| 640 | P55196 | Afadin (ALL1-fused gene from chromosome 6 protein) (Protein AF-6) | MLLT4 AF6 | 4 | 4 | 1.0093 | 0.9748 | 1.0280 | 0.8603 | 0.7798 | 0.1068 | 0.9204 | 0.7845 | 0.9908 | 0.6207 | 1.0568 | 0.7844 | 1.0000 | 0.6097 | 0.8872 | 0.6810 | 0.8395 | 0.5507 | 1.0965 | 0.6756 | 0.2630 | 0.0024 | 0.7516 | 0.3241 | 0.8166 | 0.4376 | 0.9638 | 0.8958 |
| 641 | Q14165 | Malectin | MLEC KIAA0152 | 8 | 3 | 1.2359 | 0.6225 | 1.2823 | 0.3665 | 0.8630 | 0.1228 | 0.9638 | 0.9301 | 0.5058 | 0.2039 | 0.8166 | 0.3950 | 1.0765 | 0.6386 | 0.7379 | 0.4217 | 1.4322 | 0.8306 | 1.0375 | 0.9896 | 1.1588 | 0.5341 | 1.0965 | 0.8365 | 1.5996 | 0.2405 | 0.8630 | 0.5989 |
| 642 | Q14240 | Eukaryotic initiation factor 4A-II (eIF-4A-II) (eIF4A-II) (EC 3.6.4.13) (ATP-dependent RNA helicase eIF4A-2) [Cleaved into: Eukaryotic initiation factor 4A-II, N-terminally processed] | EIF4A2 DDX2B EIF4F | 5 | 14 | 1.0568 | 0.9127 | 0.9908 | 0.9792 | 1.2023 | 0.1550 | 1.1376 | 0.7346 | 1.0666 | 0.8295 | 0.9727 | 0.8709 | 1.0375 | 0.7717 | 1.1588 | 0.2588 | 1.2823 | 0.1766 | 1.0471 | 0.7005 | 0.8710 | 0.5661 | 0.9036 | 0.7785 | 0.9204 | 0.5034 | 1.0568 | 0.5952 |
| 643 | P14174 | Macrophage migration inhibitory factor (MIF) (EC 5.3.2.1) (Glycosylation-inhibiting factor) (GIF) (L-dopachrome isomerase) (L-dopachrome tautomerase) (EC 5.3.3.12) (Phenylpyruvate tautomerase) | MIF GLIF MMIF | 5 | 8 | 1.0093 | 0.9833 | 1.1169 | 0.7856 | 0.9204 | 0.4301 | 1.8535 | 0.1830 | 1.0375 | 0.9447 | 1.1169 | 0.4470 | 0.9638 | 0.4041 | 1.0765 | 0.6704 | 1.0280 | 0.9737 | 0.7244 | 0.7920 | 1.0471 | 0.8988 | 2.1878 | 0.1464 | 0.9204 | 0.2804 | 1.0186 | 0.1622 |
| 644 | Q5JRA6 | Melanoma inhibitory activity protein 3 (C219-reactive peptide) (D320) (Transport and Golgi organization protein 1) | MIA3 KIAA0268 TANGO TANGO1 UNQ6077/PRO20088 | 21 | 6 | 0.4406 | 0.1468 | 1.2134 | 0.6232 | 0.8954 | 0.7115 | 1.0864 | 0.9678 | 0.4529 | 0.4857 | 0.8710 | 0.5929 | 0.7311 | 0.4059 | 1.0666 | 0.9276 | 0.8630 | 0.9522 | 1.2359 | 0.3980 | 1.2359 | 0.5053 | 1.1588 | 0.4769 | 0.6546 | 0.9427 | 0.9462 | 0.5415 |
| 645 | P10620 | Microsomal glutathione S-transferase 1 (Microsomal GST-1) (EC 2.5.1.18) (Microsomal GST-I) | MGST1 GST12 MGST | 8 | 10 | 1.0000 | 0.3101 | 0.5861 | 0.7110 | 1.4723 | 0.1185 | 0.9036 | 0.8485 | 1.8535 | 0.1124 | 0.4130 | 0.1569 | 0.9290 | 0.3561 | 0.8710 | 0.9072 | 0.2312 | 0.3455 | 0.6855 | 0.5670 | 0.9036 | 0.3961 | 0.3664 | 0.2673 | 0.5058 | 0.7095 | 0.1803 | 0.0303 |
| 646 | P55083 | Microfibril-associated glycoprotein 4 | MFAP4 | 3 | 8 | 0.8872 | 0.8992 | 1.1272 | 0.4799 | 0.9376 | 0.7746 | 5.8614 | 0.0444 | 0.8790 | 0.5531 | 0.3837 | 0.4591 | 0.7727 | 0.3000 | 0.3698 | 0.4635 | 0.9036 | 0.5684 | 1.0280 | 0.8053 | 0.9120 | 0.9655 | 0.1271 | 0.2185 | 0.9908 | 0.9510 | 0.1202 | 0.1341 |
| 647 | Q6UX53 | Methyltransferase-like protein 7B (EC 2.1.1.-) | METTL7B UNQ594/PRO1180 | 15 | 6 | 0.9204 | 0.6775 | 0.8166 | 0.4501 | 0.6194 | 0.0680 | 0.7516 | 0.9804 | 0.5808 | 0.2234 | 0.6026 | 0.6718 | 0.8710 | 0.8865 | 1.1912 | 0.8286 | 0.8872 | 0.5573 | 0.3373 | 0.0261 | 0.4406 | 0.0108 | 0.7870 | 0.6069 | 0.2679 | 0.0741 | 0.6310 | 0.2802 |
| 648 | Q9H8H3 | Methyltransferase-like protein 7A (EC 2.1.1.-) (Protein AAM-B) | METTL7A PRO0066 UNQ1902/PRO4348 | 4 | 10 | 0.4487 | 0.2891 | 0.8472 | 0.2495 | 4.7424 | 0.1538 | 1.0000 | 0.9423 | 0.1038 | 0.0954 | 0.5297 | 0.0213 | 1.1695 | 0.7469 | 0.7943 | 0.1600 | 2.5586 | 0.2449 | 0.7047 | 0.2553 | 0.4246 | 0.2732 | 0.7656 | 0.2160 | 0.3311 | 0.2183 | 0.4130 | 0.0466 |
| 649 | P23368 | NAD-dependent malic enzyme, mitochondrial (NAD-ME) (EC 1.1.1.38) (Malic enzyme 2) | ME2 | 10 | 4 | 1.6749 | 0.1784 | 1.0471 | 0.5204 | 0.6194 | 0.4353 | 0.7727 | 0.6361 | 1.1588 | 0.6427 | 1.0000 | 0.6531 | 1.9055 | 0.0935 | 0.7870 | 0.8599 | 1.9770 | 0.1627 | 1.1482 | 0.3852 | 1.1169 | 0.9432 | 0.9638 | 0.5315 | 1.0000 | 0.5724 | 1.1169 | 0.4711 |
| 650 | P40926 | Malate dehydrogenase, mitochondrial (EC 1.1.1.37) | MDH2 | 9 | 38 | 1.4322 | 0.8166 | 0.5445 | 0.0073 | 0.6368 | 0.1117 | 0.8318 | 0.0541 | 0.7379 | 0.2714 | 0.7047 | 0.0365 | 1.0093 | 0.4056 | 0.7516 | 0.1435 | 0.9120 | 0.8496 | 1.0280 | 0.3690 | 0.2965 | 0.0797 | 1.1482 | 0.5070 | 0.3565 | 0.3247 | 0.7656 | 0.1691 |
| 651 | P40925 | Malate dehydrogenase, cytoplasmic (EC 1.1.1.37) (Cytosolic malate dehydrogenase) (Diiodophenylpyruvate reductase) (EC 1.1.1.96) | MDH1 MDHA | 6 | 33 | 2.4434 | 0.2133 | 0.9908 | 0.5480 | 1.5560 | 0.4606 | 1.5849 | 0.6035 | 0.6081 | 0.7197 | 2.3121 | 0.1035 | 2.3768 | 0.0477 | 1.5849 | 0.9328 | 1.1695 | 0.5491 | 1.9953 | 0.5995 | 2.7290 | 0.0598 | 1.5276 | 0.9868 | 2.9923 | 0.0244 | 1.3932 | 0.9351 |
| 652 | Q96PE7 | Methylmalonyl-CoA epimerase, mitochondrial (EC 5.1.99.1) (DL-methylmalonyl-CoA racemase) | MCEE | 51 | 5 | 0.9908 | 0.3649 | 1.1169 | 0.9735 | 0.6918 | 0.1297 | 1.0864 | 0.9728 | 0.5861 | 0.0034 | 0.9727 | 0.6321 | 1.3677 | 0.1893 | 1.0186 | 0.7763 | 0.7727 | 0.2930 | 1.0864 | 0.9903 | 0.2443 | 0.0002 | 0.9638 | 0.8099 | 0.2466 | 0.0002 | 0.6855 | 0.4317 |
| 653 | Q9HCC0 | Methylcrotonoyl-CoA carboxylase beta chain, mitochondrial (MCCase subunit beta) (EC 6.4.1.4) (3-methylcrotonyl-CoA carboxylase 2) (3-methylcrotonyl-CoA carboxylase non-biotin-containing subunit) (3-methylcrotonyl-CoA:carbon dioxide ligase subunit beta) | MCCC2 MCCB | 34 | 29 | 0.7244 | 0.7707 | 0.6194 | 0.3368 | 0.4656 | 0.1893 | 0.8395 | 0.6999 | 0.8318 | 0.8866 | 0.5754 | 0.3080 | 1.3552 | 0.3655 | 0.8241 | 0.8309 | 1.4723 | 0.4720 | 0.5702 | 0.1717 | 1.0280 | 0.8052 | 0.3837 | 0.0236 | 0.5058 | 0.2803 | 0.6546 | 0.1191 |
| 654 | Q96RQ3 | Methylcrotonoyl-CoA carboxylase subunit alpha, mitochondrial (MCCase subunit alpha) (EC 6.4.1.4) (3-methylcrotonyl-CoA carboxylase 1) (3-methylcrotonyl-CoA carboxylase biotin-containing subunit) (3-methylcrotonyl-CoA:carbon dioxide ligase subunit alpha) | MCCC1 MCCA | 3 | 14 | 1.0568 | 0.6956 | 1.0375 | 0.6436 | 1.2134 | 0.6735 | 1.0471 | 0.7827 | 0.8241 | 0.7582 | 1.0375 | 0.4235 | 1.7219 | 0.3312 | 1.0765 | 0.1358 | 0.9817 | 0.7770 | 1.0186 | 0.5881 | 0.4169 | 0.4154 | 1.0186 | 0.4826 | 0.5702 | 0.4350 | 0.9638 | 0.9857 |
| 655 | P11226 | Mannose-binding protein C (MBP-C) (Collectin-1) (MBP1) (Mannan-binding protein) (Mannose-binding lectin) | MBL2 COLEC1 MBL | 24 | 4 | 0.8241 | 0.9625 | 0.4169 | 0.0683 | 0.7943 | 0.3591 | 2.0701 | 0.4168 | 0.7656 | 0.7721 | 0.7586 | 0.2914 | 1.3183 | 0.2909 | 1.0280 | 0.4393 | 1.2942 | 0.2558 | 0.8872 | 0.4070 | 0.3048 | 0.0019 | 1.3677 | 0.9220 | 0.4529 | 0.1720 | 0.6792 | 0.1082 |
| 656 | Q7Z434 | Mitochondrial antiviral-signaling protein (MAVS) (CARD adapter inducing interferon beta) (Cardif) (Interferon beta promoter stimulator protein 1) (IPS-1) (Putative NF-kappa-B-activating protein 031N) (Virus-induced-signaling adapter) (VISA) | MAVS IPS1 KIAA1271 VISA | 14 | 4 | 1.5849 | 0.2723 | 2.0512 | 0.3609 | 1.3932 | 0.2679 | 1.3552 | 0.5710 | 0.8954 | 0.8712 | 0.3251 | 0.1973 | 2.1677 | 0.0011 | 1.6444 | 0.3075 | 1.6444 | 0.0806 | 2.2080 | 0.2806 | 0.2109 | 0.0005 | 1.0666 | 0.7749 | 0.8318 | 0.9433 | 1.5136 | 0.4536 |
| 657 | P43243 | Matrin-3 | MATR3 KIAA0723 | 3 | 11 | 2.7797 | 0.0423 | 1.0375 | 0.5133 | 1.0000 | 0.9096 | 1.1912 | 0.4906 | 1.1588 | 0.5333 | 1.3183 | 0.1523 | 1.7865 | 0.2237 | 0.9908 | 0.8539 | 0.6855 | 0.4484 | 1.2134 | 0.3119 | 0.7244 | 0.6210 | 1.1169 | 0.5204 | 0.5808 | 0.8117 | 1.2474 | 0.2529 |
| 658 | Q9NZL9 | Methionine adenosyltransferase 2 subunit beta (Methionine adenosyltransferase II beta) (MAT II beta) (Putative dTDP-4-keto-6-deoxy-D-glucose 4-reductase) | MAT2B TGR MSTP045 Nbla02999 UNQ2435/PRO4995 | 3 | 6 | 1.3932 | 0.5630 | 0.8872 | 0.4136 | 1.1912 | 0.4501 | 1.5704 | 0.2527 | 1.5276 | 0.5315 | 1.4997 | 0.2899 | 0.9638 | 0.9562 | 1.0864 | 0.8174 | 1.1803 | 0.7463 | 1.2474 | 0.6144 | 1.4723 | 0.4489 | 1.7701 | 0.1456 | 1.2023 | 0.6227 | 1.0186 | 0.8983 |
| 659 | P31153 | S-adenosylmethionine synthase isoform type-2 (AdoMet synthase 2) (EC 2.5.1.6) (Methionine adenosyltransferase 2) (MAT 2) (Methionine adenosyltransferase II) (MAT-II) | MAT2A AMS2 MATA2 | 12 | 4 | 0.8091 | 0.0898 | 0.9120 | 0.7107 | 1.0765 | 0.5990 | 1.0093 | 0.7718 | 1.0186 | 0.9503 | 0.9120 | 0.6209 | 0.8790 | 0.2744 | 0.7727 | 0.3973 | 1.0000 | 0.8456 | 1.0471 | 0.7125 | 1.0093 | 0.8370 | 0.8472 | 0.5881 | 0.9120 | 0.3393 | 0.9550 | 0.9712 |
| 660 | Q00266 | S-adenosylmethionine synthase isoform type-1 (AdoMet synthase 1) (EC 2.5.1.6) (Methionine adenosyltransferase 1) (MAT 1) (Methionine adenosyltransferase I/III) (MAT-I/III) | MAT1A AMS1 MATA1 | 4 | 22 | 1.4723 | 0.5063 | 0.7447 | 0.6197 | 1.1912 | 0.8847 | 1.1376 | 0.2531 | 1.7539 | 0.3268 | 0.2312 | 0.0046 | 1.2706 | 0.8624 | 0.3499 | 0.0186 | 1.3677 | 0.5732 | 0.4169 | 0.0385 | 0.4875 | 0.7979 | 0.6546 | 0.0557 | 1.1482 | 0.5842 | 0.1445 | 0.0130 |
| 661 | P29966 | Myristoylated alanine-rich C-kinase substrate (MARCKS) (Protein kinase C substrate, 80 kDa protein, light chain) (80K-L protein) (PKCSL) | MARCKS MACS PRKCSL | 5 | 4 | 1.0666 | 0.7219 | 1.0000 | 0.4151 | 0.7798 | 0.4892 | 0.6855 | 0.3666 | 0.9817 | 0.9868 | 1.8707 | 0.0402 | 0.9290 | 0.7561 | 0.4169 | 0.3041 | 0.7379 | 0.4203 | 1.8365 | 0.0234 | 1.0375 | 0.7974 | 0.9727 | 0.5253 | 1.0864 | 0.7069 | 1.7378 | 0.0626 |
| 662 | P27816 | Microtubule-associated protein 4 (MAP-4) | MAP4 | 26 | 8 | 0.9550 | 0.2798 | 1.9055 | 0.1050 | 0.6730 | 0.3129 | 2.9376 | 0.0614 | 0.5754 | 0.0659 | 3.5318 | 0.0108 | 1.0864 | 0.6747 | 2.9376 | 0.0935 | 0.6026 | 0.0370 | 3.2211 | 0.0263 | 0.1393 | 0.0005 | 4.1305 | 0.0091 | 0.4529 | 0.0084 | 4.9204 | 0.0002 |
| 663 | P27338 | Amine oxidase [flavin-containing] B (EC 1.4.3.4) (Monoamine oxidase type B) (MAO-B) | MAOB | 6 | 34 | 0.5152 | 0.4989 | 1.4454 | 0.5686 | 1.8197 | 0.4457 | 2.0512 | 0.0399 | 0.5058 | 0.1994 | 1.2134 | 0.9191 | 1.2823 | 0.4647 | 0.7727 | 0.5618 | 0.7586 | 0.6459 | 1.4191 | 0.4981 | 4.4463 | 0.0283 | 1.4060 | 0.4684 | 2.1281 | 0.6465 | 0.9036 | 0.7789 |
| 664 | P21397 | Amine oxidase [flavin-containing] A (EC 1.4.3.4) (Monoamine oxidase type A) (MAO-A) | MAOA | 7 | 26 | 2.1878 | 0.2213 | 0.7586 | 0.3888 | 4.6559 | 0.0191 | 0.9638 | 0.9844 | 3.3419 | 0.1847 | 0.6368 | 0.0815 | 1.9953 | 0.0695 | 0.7311 | 0.3897 | 1.9055 | 0.5660 | 0.6918 | 0.0894 | 5.2966 | 0.0126 | 0.8954 | 0.9009 | 4.5709 | 0.0113 | 0.2780 | 0.0016 |
| 665 | Q9UPN3 | Microtubule-actin cross-linking factor 1, isoforms 1/2/3/5 (620 kDa actin-binding protein) (ABP620) (Actin cross-linking family protein 7) (Macrophin-1) (Trabeculin-alpha) | MACF1 ABP620 ACF7 KIAA0465 KIAA1251 | 36 | 10 | 0.4875 | 0.1297 | 0.9727 | 0.9951 | 1.3428 | 0.7928 | 1.0471 | 0.8632 | 1.1066 | 0.6471 | 0.8395 | 0.0535 | 1.8365 | 0.1033 | 0.8872 | 0.4443 | 2.3335 | 0.0015 | 0.9036 | 0.6837 | 0.2729 | 0.0007 | 0.9462 | 0.6385 | 0.3664 | 0.0028 | 0.9908 | 0.9499 |
| 666 | P61626 | Lysozyme C (EC 3.2.1.17) (1,4-beta-N-acetylmuramidase C) | LYZ LZM | 25 | 4 | 0.8472 | 0.9717 | 0.3162 | 0.0053 | 0.7047 | 0.1471 | 0.5152 | 0.2093 | 0.5754 | 0.0496 | 0.4742 | 0.0214 | 0.7379 | 0.2361 | 0.4966 | 0.0225 | 0.9908 | 0.8381 | 0.3873 | 0.0215 | 0.1213 | 0.0002 | 0.2606 | 0.0162 | 0.3767 | 0.0035 | 0.8472 | 0.6872 |
| 667 | Q5VWZ2 | Lysophospholipase-like protein 1 (EC 3.1.2.-) | LYPLAL1 | 6 | 3 | 2.3768 | 0.1465 | 1.1169 | 0.6869 | 2.4660 | 0.1191 | 1.1169 | 0.8582 | 1.3677 | 0.4715 | 0.7112 | 0.3387 | 1.7219 | 0.4282 | 0.9550 | 0.8999 | 2.2909 | 0.0973 | 0.9817 | 0.7682 | 1.6904 | 0.2621 | 1.0471 | 0.8651 | 3.4356 | 0.0015 | 1.0765 | 0.8973 |
| 668 | O75608 | Acyl-protein thioesterase 1 (APT-1) (hAPT1) (EC 3.1.2.-) (Lysophospholipase 1) (Lysophospholipase I) (LPL-I) (LysoPLA I) | LYPLA1 APT1 LPL1 | 5 | 5 | 0.1754 | 0.0076 | 0.5808 | 0.2910 | 0.4365 | 0.0260 | 1.1912 | 0.9793 | 0.3597 | 0.0224 | 1.0000 | 0.8227 | 0.0809 | 0.0018 | 1.0765 | 0.5845 | 0.2168 | 0.0050 | 1.2359 | 0.7815 | 1.1803 | 0.4711 | 1.5276 | 0.4042 | 0.5861 | 0.2398 | 0.6486 | 0.5372 |
| 669 | P51884 | Lumican (Keratan sulfate proteoglycan lumican) (KSPG lumican) | LUM LDC SLRR2D | 5 | 20 | 0.7798 | 0.7978 | 0.3767 | 0.0377 | 0.9908 | 0.8865 | 1.7865 | 0.0019 | 0.7586 | 0.4786 | 0.6546 | 0.2434 | 0.9908 | 0.8251 | 1.0471 | 0.6254 | 1.5996 | 0.2000 | 0.4786 | 0.3480 | 1.1912 | 0.4542 | 0.0982 | 0.0028 | 0.7311 | 0.4661 | 0.9727 | 0.9014 |
| 670 | P02788 | Lactotransferrin (Lactoferrin) (EC 3.4.21.-) (Growth-inhibiting protein 12) (Talalactoferrin) [Cleaved into: Lactoferricin-H (Lfcin-H); Kaliocin-1; Lactoferroxin-A; Lactoferroxin-B; Lactoferroxin-C] | LTF GIG12 LF | 7 | 8 | 1.3804 | 0.5225 | 0.2208 | 0.0389 | 1.4060 | 0.9077 | 0.4831 | 0.0643 | 3.0479 | 0.3112 | 0.3404 | 0.0399 | 1.7061 | 0.3777 | 0.4699 | 0.1187 | 1.5417 | 0.6285 | 0.6368 | 0.0493 | 0.5808 | 0.3816 | 0.5702 | 0.0118 | 1.4454 | 0.7518 | 0.3192 | 0.0813 |
| 671 | P09960 | Leukotriene A-4 hydrolase (LTA-4 hydrolase) (EC 3.3.2.6) (Leukotriene A(4) hydrolase) | LTA4H LTA4 | 17 | 9 | 0.2729 | 0.0021 | 0.9727 | 0.8332 | 2.2909 | 0.0002 | 1.0471 | 0.5798 | 0.5105 | 0.0432 | 1.2589 | 0.8377 | 0.6026 | 0.1455 | 0.7798 | 0.5044 | 1.1803 | 0.4064 | 1.2246 | 0.7591 | 0.4018 | 0.0013 | 0.9727 | 0.9360 | 0.1614 | 0.0002 | 2.2491 | 0.2220 |
| 672 | P48449 | Lanosterol synthase (EC 5.4.99.7) (2,3-epoxysqualene--lanosterol cyclase) (Oxidosqualene--lanosterol cyclase) (OSC) (hOSC) | LSS OSC | 13 | 18 | 0.4966 | 0.0273 | 1.1803 | 0.4514 | 0.5702 | 0.0010 | 1.2589 | 0.3492 | 0.5702 | 0.0039 | 0.3020 | 0.0001 | 0.2333 | 0.0001 | 1.7539 | 0.0276 | 0.3873 | 0.0003 | 1.7378 | 0.0625 | 1.3305 | 0.3591 | 1.6444 | 0.0335 | 0.3767 | 0.0026 | 0.4325 | 0.0103 |
| 673 | Q96AG4 | Leucine-rich repeat-containing protein 59 (Ribosome-binding protein p34) (p34) | LRRC59 PRO1855 | 10 | 5 | 0.4786 | 0.0569 | 1.9409 | 0.5855 | 0.4699 | 0.0245 | 2.9107 | 0.2218 | 0.6026 | 0.2276 | 3.5645 | 0.0206 | 0.9638 | 0.7639 | 1.8365 | 0.6196 | 0.9204 | 0.6058 | 3.6983 | 0.1917 | 1.0471 | 0.9632 | 3.3729 | 0.1667 | 0.8954 | 0.3070 | 3.5318 | 0.1538 |
| 674 | Q8N1G4 | Leucine-rich repeat-containing protein 47 | LRRC47 KIAA1185 | 16 | 14 | 1.0280 | 0.9413 | 0.9638 | 0.8276 | 1.0568 | 0.9769 | 0.9908 | 0.9203 | 1.3305 | 0.0097 | 1.1272 | 0.2138 | 0.6918 | 0.1279 | 0.8630 | 0.4129 | 1.1695 | 0.1845 | 0.9376 | 0.5453 | 0.1047 | 0.0002 | 1.0765 | 0.3971 | 0.7447 | 0.3015 | 1.0965 | 0.5046 |
| 675 | P42704 | Leucine-rich PPR motif-containing protein, mitochondrial (130 kDa leucine-rich protein) (LRP 130) (GP130) | LRPPRC LRP130 | 6 | 35 | 0.4529 | 0.0145 | 1.1272 | 0.5512 | 0.8630 | 0.2428 | 0.8551 | 0.2565 | 1.0568 | 0.9798 | 0.3908 | 0.0007 | 1.1272 | 0.8166 | 0.9727 | 0.4534 | 0.6138 | 0.1725 | 1.3552 | 0.7185 | 1.2706 | 0.3098 | 1.0965 | 0.9081 | 1.0093 | 0.5536 | 0.9817 | 0.9290 |
| 676 | Q07954 | Prolow-density lipoprotein receptor-related protein 1 (LRP-1) (Alpha-2-macroglobulin receptor) (A2MR) (Apolipoprotein E receptor) (APOER) (CD antigen CD91) [Cleaved into: Low-density lipoprotein receptor-related protein 1 85 kDa subunit (LRP-85); Low-density lipoprotein receptor-related protein 1 515 kDa subunit (LRP-515); Low-density lipoprotein receptor-related protein 1 intracellular domain (LRPICD)] | LRP1 A2MR APR | 12 | 22 | 3.1915 | 0.0205 | 0.9908 | 0.4579 | 1.4191 | 0.6379 | 0.9817 | 0.6507 | 1.9953 | 0.0459 | 0.7943 | 0.9526 | 1.2942 | 0.3809 | 1.0471 | 0.7948 | 2.0512 | 0.0518 | 1.5276 | 0.1236 | 1.4060 | 0.6381 | 0.8630 | 0.7728 | 2.1677 | 0.0815 | 1.4060 | 0.3882 |
| 677 | Q93052 | Lipoma-preferred partner (LIM domain-containing preferred translocation partner in lipoma) | LPP | 30 | 3 | 0.9817 | 0.9402 | 0.5346 | 0.3186 | 0.6138 | 0.0570 | 1.0000 | 0.9910 | 0.8551 | 0.4413 | 1.1272 | 0.4215 | 1.3062 | 0.0995 | 0.5754 | 0.6873 | 0.7656 | 0.0855 | 0.7047 | 0.8111 | 1.0093 | 0.9682 | 0.7586 | 0.3982 | 0.9462 | 0.5668 | 0.7943 | 0.6849 |
| 678 | P36776 | Lon protease homolog, mitochondrial (EC 3.4.21.-) (LONHs) (Lon protease-like protein) (LONP) (Mitochondrial ATP-dependent protease Lon) (Serine protease 15) | LONP1 PRSS15 | 20 | 20 | 0.8091 | 0.4618 | 0.5248 | 0.0693 | 1.4723 | 0.3201 | 0.5598 | 0.1396 | 1.3062 | 0.4008 | 0.9638 | 0.6938 | 1.2134 | 0.7136 | 1.0666 | 0.6321 | 1.1482 | 0.4692 | 0.9120 | 0.1900 | 1.5849 | 0.0769 | 0.9638 | 0.6056 | 1.2023 | 0.5875 | 0.7586 | 0.3386 |
| 679 | P39023 | 60S ribosomal protein L3 (HIV-1 TAR RNA-binding protein B) (TARBP-B) | RPL3 OK/SW-cl.32 | 7 | 17 | 0.4406 | 0.3693 | 0.5012 | 0.2364 | 1.8880 | 0.2177 | 0.4365 | 0.1031 | 1.2134 | 0.9224 | 1.2589 | 0.3829 | 0.8630 | 0.6435 | 0.6368 | 0.2376 | 0.9376 | 0.9010 | 0.8790 | 0.7684 | 0.5598 | 0.6284 | 1.1588 | 0.9540 | 1.2134 | 0.7048 | 1.1695 | 0.5304 |
| 680 | P84243 | Histone H3.3 | H3F3A H3.3A H3F3 PP781; H3F3B H3.3B | 19 | 29 | 0.6310 | 0.3042 | 2.6062 | 0.4323 | 1.1066 | 0.8609 | 4.3652 | 0.3622 | 0.6026 | 0.1051 | 2.8840 | 0.4232 | 0.9638 | 0.8503 | 2.4434 | 0.6235 | 0.7943 | 0.4555 | 4.8306 | 0.3748 | 0.6486 | 0.0663 | 3.8726 | 0.2912 | 0.6138 | 0.0723 | 0.1690 | 0.5207 |
| 681 | Q30154 | HLA class II histocompatibility antigen, DR beta 5 chain (DR beta-5) (DR2-beta-2) (Dw2) (MHC class II antigen DRB5) | HLA-DRB5 | 18 | 3 | 1.0471 | 0.7100 | 1.7061 | 0.2107 | 0.8872 | 0.3350 | 0.9908 | 0.4488 | 1.2589 | 0.7279 | 2.2699 | 0.1583 | 1.6444 | 0.4683 | 1.3183 | 0.3903 | 1.4454 | 0.8798 | 2.2284 | 0.1588 | 2.1281 | 0.0252 | 0.8954 | 0.4981 | 1.0093 | 0.9006 | 1.4588 | 0.2652 |
| 682 | P27144 | Adenylate kinase 4, mitochondrial (AK 4) (EC 2.7.4.10) (EC 2.7.4.6) (Adenylate kinase 3-like) (GTP:AMP phosphotransferase AK4) | AK4 AK3 AK3L1 | 33 | 9 | 1.0280 | 0.5786 | 1.7539 | 0.3344 | 2.6546 | 0.2369 | 1.6596 | 0.7693 | 2.2080 | 0.3133 | 1.3552 | 0.7665 | 1.9409 | 0.5123 | 2.2909 | 0.1628 | 1.6444 | 0.4147 | 2.0512 | 0.3659 | 0.3133 | 0.5116 | 2.2909 | 0.1326 | 0.0370 | 0.1399 | 0.9290 | 0.6370 |
| 683 | Q8WWI1 | LIM domain only protein 7 (LMO-7) (F-box only protein 20) (LOMP) | LMO7 FBX20 FBXO20 KIAA0858 | 3 | 9 | 0.6546 | 0.4632 | 1.1169 | 0.4636 | 0.9550 | 0.7580 | 0.9204 | 0.3744 | 0.2754 | 0.5009 | 0.8551 | 0.5495 | 0.4365 | 0.1560 | 1.0471 | 0.9832 | 0.1202 | 0.1118 | 0.8710 | 0.2473 | 1.8365 | 0.3457 | 1.0375 | 0.6366 | 0.9376 | 0.9204 | 0.9462 | 0.7945 |
| 684 | Q03252 | Lamin-B2 | LMNB2 LMN2 | 14 | 11 | 1.1376 | 0.5293 | 0.7516 | 0.6235 | 0.7798 | 0.2257 | 1.9588 | 0.5651 | 0.9550 | 0.7267 | 0.9550 | 0.5119 | 0.7379 | 0.6776 | 1.2474 | 0.3494 | 0.9290 | 0.9172 | 1.5704 | 0.7009 | 0.3733 | 0.0034 | 1.1169 | 0.0968 | 0.5445 | 0.0950 | 1.3804 | 0.9243 |
| 685 | P20700 | Lamin-B1 | LMNB1 LMN2 LMNB | 8 | 20 | 1.2823 | 0.4221 | 0.8710 | 0.7259 | 1.1588 | 0.6563 | 0.6310 | 0.1993 | 0.3873 | 0.3192 | 1.0765 | 0.6149 | 0.6668 | 0.3577 | 0.2655 | 0.0128 | 0.9908 | 0.7319 | 1.0186 | 0.7039 | 0.6252 | 0.1974 | 0.8472 | 0.4744 | 1.0093 | 0.9635 | 1.5417 | 0.0415 |
| 686 | P02545 | Prelamin-A/C [Cleaved into: Lamin-A/C (70 kDa lamin) (Renal carcinoma antigen NY-REN-32)] | LMNA LMN1 | 8 | 51 | 0.9290 | 0.6164 | 0.8551 | 0.0770 | 1.1695 | 0.3258 | 1.3677 | 0.2934 | 0.9727 | 0.7041 | 1.1912 | 0.8085 | 0.8710 | 0.4184 | 0.3105 | 0.0000 | 1.0965 | 0.6034 | 0.4055 | 0.0004 | 1.1066 | 0.4961 | 0.9036 | 0.1738 | 0.9817 | 0.8238 | 0.5200 | 0.0032 |
| 687 | Q12907 | Vesicular integral-membrane protein VIP36 (Glycoprotein GP36b) (Lectin mannose-binding 2) (Vesicular integral-membrane protein 36) (VIP36) | LMAN2 C5orf8 | 20 | 10 | 0.9908 | 0.7256 | 1.0666 | 0.6691 | 1.5849 | 0.1368 | 0.9036 | 0.6199 | 1.5560 | 0.0425 | 0.9638 | 0.7265 | 0.6310 | 0.6676 | 0.9120 | 0.6598 | 1.9055 | 0.1207 | 0.9817 | 0.6272 | 2.4434 | 0.0005 | 1.0186 | 0.9601 | 1.7865 | 0.0682 | 0.9290 | 0.4574 |
| 688 | P49257 | Protein ERGIC-53 (ER-Golgi intermediate compartment 53 kDa protein) (Gp58) (Intracellular mannose-specific lectin MR60) (Lectin mannose-binding 1) | LMAN1 ERGIC53 F5F8D | 62 | 10 | 0.5012 | 0.0001 | 1.1376 | 0.9649 | 1.3552 | 0.3160 | 1.1169 | 0.4628 | 0.5152 | 0.0018 | 1.0471 | 0.8082 | 0.6546 | 0.0076 | 0.9908 | 0.1075 | 1.1695 | 0.7857 | 1.1272 | 0.6513 | 0.5297 | 0.0008 | 1.2023 | 0.8814 | 0.5754 | 0.0015 | 1.1169 | 0.4993 |
| 689 | Q9H008 | Phospholysine phosphohistidine inorganic pyrophosphate phosphatase (hLHPP) (EC 3.1.3.-) (EC 3.6.1.1) | LHPP | 9 | 9 | 0.7516 | 0.8105 | 1.8707 | 0.5541 | 1.5704 | 0.2330 | 1.4454 | 0.5332 | 1.4859 | 0.1181 | 3.9084 | 0.0746 | 0.9817 | 0.7411 | 1.6144 | 0.6656 | 0.9120 | 0.8345 | 2.7797 | 0.2234 | 1.5136 | 0.2735 | 2.4434 | 0.1480 | 0.9550 | 0.7529 | 1.2706 | 0.7375 |
| 690 | P56470 | Galectin-4 (Gal-4) (Antigen NY-CO-27) (L-36 lactose-binding protein) (L36LBP) (Lactose-binding lectin 4) | LGALS4 | 15 | 10 | 0.7047 | 0.2709 | 2.7040 | 0.0457 | 0.7112 | 0.2391 | 0.7112 | 0.2054 | 0.8017 | 0.5098 | 0.4487 | 0.0490 | 0.7311 | 0.4900 | 0.1854 | 0.0006 | 0.7870 | 0.4815 | 2.7040 | 0.0605 | 0.6310 | 0.0722 | 0.6918 | 0.2282 | 0.5970 | 0.1477 | 2.3335 | 0.1431 |
| 691 | Q08380 | Galectin-3-binding protein (Basement membrane autoantigen p105) (Lectin galactoside-binding soluble 3-binding protein) (Mac-2-binding protein) (MAC2BP) (Mac-2 BP) (Tumor-associated antigen 90K) | LGALS3BP M2BP | 9 | 6 | 1.7378 | 0.5588 | 1.1695 | 0.9641 | 1.2474 | 0.6567 | 0.9638 | 0.9849 | 2.0893 | 0.2434 | 2.9923 | 0.0018 | 0.6855 | 0.9695 | 0.4130 | 0.1675 | 1.3677 | 0.8250 | 1.1695 | 0.7977 | 0.1282 | 0.0670 | 1.0093 | 0.6494 | 1.0666 | 0.6990 | 1.3552 | 0.8686 |
| 692 | P09382 | Galectin-1 (Gal-1) (14 kDa laminin-binding protein) (HLBP14) (14 kDa lectin) (Beta-galactoside-binding lectin L-14-I) (Galaptin) (HBL) (HPL) (Lactose-binding lectin 1) (Lectin galactoside-binding soluble 1) (Putative MAPK-activating protein PM12) (S-Lac lectin 1) | LGALS1 | 13 | 19 | 0.8954 | 0.9667 | 6.2517 | 0.0236 | 0.4529 | 0.0653 | 7.7268 | 0.0014 | 0.7178 | 0.5914 | 8.3946 | 0.0014 | 3.6983 | 0.0005 | 5.8614 | 0.0160 | 2.4889 | 0.0070 | 6.7920 | 0.0020 | 1.0965 | 0.5090 | 7.2444 | 0.0025 | 0.5012 | 0.1020 | 3.1915 | 0.0707 |
| 693 | Q86WU2 | Probable D-lactate dehydrogenase, mitochondrial (DLD) (Lactate dehydrogenase D) (EC 1.1.2.4) | LDHD | 5 | 13 | 1.1376 | 0.8702 | 1.1169 | 0.4848 | 1.3183 | 0.5262 | 0.6368 | 0.4890 | 1.0666 | 0.9698 | 0.7656 | 0.5569 | 1.9409 | 0.0884 | 0.7244 | 0.9983 | 1.1272 | 0.7205 | 0.3733 | 0.0807 | 1.0280 | 0.9436 | 0.5970 | 0.6510 | 2.0512 | 0.0247 | 0.4529 | 0.3777 |
| 694 | P07195 | L-lactate dehydrogenase B chain (LDH-B) (EC 1.1.1.27) (LDH heart subunit) (LDH-H) (Renal carcinoma antigen NY-REN-46) | LDHB | 22 | 8 | 1.1695 | 0.8455 | 1.2942 | 0.9676 | 1.6749 | 0.6171 | 1.4060 | 0.8696 | 2.4434 | 0.0477 | 1.4859 | 0.9654 | 2.1677 | 0.2098 | 0.7586 | 0.2980 | 2.9648 | 0.0557 | 1.6596 | 0.4332 | 1.3804 | 0.2521 | 0.3192 | 0.0188 | 0.8710 | 0.4023 | 1.7865 | 0.1824 |
| 695 | P00338 | L-lactate dehydrogenase A chain (LDH-A) (EC 1.1.1.27) (Cell proliferation-inducing gene 19 protein) (LDH muscle subunit) (LDH-M) (Renal carcinoma antigen NY-REN-59) | LDHA PIG19 | 16 | 32 | 1.3062 | 0.7812 | 0.1820 | 0.0002 | 1.5136 | 0.7401 | 0.8472 | 0.0818 | 0.7798 | 0.5325 | 0.3251 | 0.0003 | 1.3677 | 0.6167 | 0.4018 | 0.0002 | 1.7219 | 0.2250 | 0.9120 | 0.3832 | 0.1995 | 0.0326 | 0.5346 | 0.0293 | 0.8017 | 0.2744 | 0.5546 | 0.0021 |
| 696 | P13796 | Plastin-2 (L-plastin) (LC64P) (Lymphocyte cytosolic protein 1) (LCP-1) | LCP1 PLS2 | 10 | 31 | 0.3802 | 0.1176 | 1.2942 | 0.7479 | 1.5136 | 0.5000 | 0.9908 | 0.6018 | 0.8472 | 0.8003 | 2.7040 | 0.0014 | 0.9462 | 0.9220 | 0.6310 | 0.0820 | 1.0093 | 0.9118 | 3.1046 | 0.0038 | 2.4210 | 0.0018 | 0.7798 | 0.0989 | 1.6144 | 0.2315 | 3.0479 | 0.0000 |
| 697 | Q14847 | LIM and SH3 domain protein 1 (LASP-1) (Metastatic lymph node gene 50 protein) (MLN 50) | LASP1 MLN50 | 49 | 11 | 0.5754 | 0.0008 | 0.7379 | 0.2549 | 0.4613 | 0.0000 | 1.7701 | 0.3870 | 0.1614 | 0.0001 | 2.1478 | 0.1534 | 1.7219 | 0.0033 | 0.8630 | 0.7179 | 0.3499 | 0.0000 | 1.9588 | 0.1862 | 0.3373 | 0.0000 | 2.1478 | 0.2284 | 0.2938 | 0.0000 | 1.6749 | 0.5382 |
| 698 | Q9P2J5 | Leucine--tRNA ligase, cytoplasmic (EC 6.1.1.4) (Leucyl-tRNA synthetase) (LeuRS) | LARS KIAA1352 | 36 | 3 | 0.7516 | 0.1625 | 1.1169 | 0.3454 | 1.3062 | 0.3121 | 1.2474 | 0.1611 | 0.6792 | 0.5559 | 1.0568 | 0.7195 | 0.8954 | 0.2148 | 1.1169 | 0.4742 | 0.9204 | 0.4930 | 1.1588 | 0.3169 | 4.1687 | 0.0000 | 1.1912 | 0.3222 | 2.8314 | 0.0118 | 1.0965 | 0.7340 |
| 699 | P28838 | Cytosol aminopeptidase (EC 3.4.11.1) (Leucine aminopeptidase 3) (LAP-3) (Leucyl aminopeptidase) (Peptidase S) (Proline aminopeptidase) (EC 3.4.11.5) (Prolyl aminopeptidase) | LAP3 LAPEP PEPS | 11 | 45 | 0.9638 | 0.9182 | 0.7727 | 0.2804 | 3.4041 | 0.0436 | 0.2606 | 0.0002 | 2.9923 | 0.1219 | 2.0324 | 0.0000 | 1.9770 | 0.5093 | 0.3698 | 0.0004 | 0.9204 | 0.6864 | 1.2134 | 0.2677 | 3.3729 | 0.0683 | 0.9376 | 0.5003 | 1.9231 | 0.1852 | 1.1066 | 0.2550 |
| 700 | P13473 | Lysosome-associated membrane glycoprotein 2 (LAMP-2) (Lysosome-associated membrane protein 2) (CD107 antigen-like family member B) (CD antigen CD107b) | LAMP2 | 5 | 5 | 1.3183 | 0.1937 | 1.4191 | 0.5546 | 0.8318 | 0.5234 | 1.3183 | 0.9269 | 0.9638 | 0.9089 | 1.9055 | 0.2167 | 1.0471 | 0.8320 | 1.6293 | 0.2410 | 0.9036 | 0.5715 | 2.1086 | 0.2454 | 1.2706 | 0.1538 | 2.3121 | 0.0565 | 1.0965 | 0.6883 | 2.4210 | 0.0600 |
| 701 | P11279 | Lysosome-associated membrane glycoprotein 1 (LAMP-1) (Lysosome-associated membrane protein 1) (CD107 antigen-like family member A) (CD antigen CD107a) | LAMP1 | 50 | 6 | 0.3076 | 0.0000 | 0.6310 | 0.9101 | 0.6855 | 0.1372 | 1.0864 | 0.6015 | 0.3698 | 0.0003 | 1.0765 | 0.3792 | 0.4699 | 0.0013 | 1.2134 | 0.1292 | 0.5649 | 0.0241 | 1.0000 | 0.4712 | 0.4018 | 0.0001 | 1.6293 | 0.1396 | 0.8318 | 0.0274 | 1.8030 | 0.0553 |
| 702 | P11047 | Laminin subunit gamma-1 (Laminin B2 chain) (Laminin-1 subunit gamma) (Laminin-10 subunit gamma) (Laminin-11 subunit gamma) (Laminin-2 subunit gamma) (Laminin-3 subunit gamma) (Laminin-4 subunit gamma) (Laminin-6 subunit gamma) (Laminin-7 subunit gamma) (Laminin-8 subunit gamma) (Laminin-9 subunit gamma) (S-laminin subunit gamma) (S-LAM gamma) | LAMC1 LAMB2 | 5 | 7 | 1.2246 | 0.7497 | 0.4920 | 0.1609 | 1.7701 | 0.3240 | 0.7311 | 0.2847 | 1.8365 | 0.2299 | 0.9550 | 0.8288 | 1.2589 | 0.4949 | 0.1148 | 0.0450 | 1.1695 | 0.8588 | 0.6026 | 0.2063 | 2.8054 | 0.0889 | 0.4169 | 0.1353 | 2.2491 | 0.1219 | 0.9120 | 0.6072 |
| 703 | P55268 | Laminin subunit beta-2 (Laminin B1s chain) (Laminin-11 subunit beta) (Laminin-14 subunit beta) (Laminin-15 subunit beta) (Laminin-3 subunit beta) (Laminin-4 subunit beta) (Laminin-7 subunit beta) (Laminin-9 subunit beta) (S-laminin subunit beta) (S-LAM beta) | LAMB2 LAMS | 4 | 6 | 1.6444 | 0.6495 | 0.8166 | 0.8473 | 1.0568 | 0.5803 | 1.2589 | 0.6469 | 1.9588 | 0.4306 | 1.1695 | 0.9802 | 1.2706 | 0.9193 | 1.4322 | 0.9208 | 0.5297 | 0.2102 | 1.0864 | 0.9524 | 1.8365 | 0.4471 | 0.8091 | 0.6279 | 2.3768 | 0.2159 | 0.6252 | 0.5937 |
| 704 | O15230 | Laminin subunit alpha-5 (Laminin-10 subunit alpha) (Laminin-11 subunit alpha) (Laminin-15 subunit alpha) | LAMA5 KIAA0533 KIAA1907 | 5 | 6 | 0.8241 | 0.9871 | 1.2589 | 0.9566 | 1.2023 | 0.4075 | 1.8365 | 0.3822 | 1.1376 | 0.5034 | 1.9770 | 0.3818 | 1.1803 | 0.5225 | 1.2023 | 0.8978 | 2.2699 | 0.0207 | 0.9817 | 0.9820 | 0.8872 | 0.9455 | 0.5105 | 0.5583 | 1.1695 | 0.3957 | 1.0568 | 0.8393 |
| 705 | O00515 | Ladinin-1 (Lad-1) (Linear IgA disease antigen) (LADA) | LAD1 LAD | 5 | 4 | 1.4723 | 0.9755 | 1.7701 | 0.2332 | 1.9588 | 0.3805 | 1.6596 | 0.1747 | 0.6982 | 0.2585 | 1.9409 | 0.1654 | 1.3804 | 0.9671 | 1.9588 | 0.0874 | 2.3121 | 0.0322 | 0.5546 | 0.9969 | 0.8872 | 0.4125 | 1.8880 | 0.0944 | 1.4454 | 0.8105 | 2.7290 | 0.0131 |
| 706 | Q53H82 | Beta-lactamase-like protein 2 (EC 3.-.-.-) | LACTB2 CGI-83 | 7 | 5 | 1.3183 | 0.3696 | 0.5970 | 0.3082 | 1.7378 | 0.0787 | 0.2858 | 0.1287 | 1.0765 | 0.8131 | 0.4246 | 0.1114 | 0.6310 | 0.9469 | 0.9462 | 0.8755 | 1.8707 | 0.1451 | 0.8472 | 0.7998 | 0.4875 | 0.2122 | 0.7112 | 0.9595 | 0.8872 | 0.7005 | 0.6918 | 0.4282 |
| 707 | P83111 | Serine beta-lactamase-like protein LACTB, mitochondrial (EC 3.4.-.-) | LACTB MRPL56 UNQ843/PRO1781 | 5 | 13 | 0.8710 | 0.8213 | 0.8551 | 0.2271 | 0.7311 | 0.4797 | 0.8790 | 0.3711 | 2.0137 | 0.0961 | 0.8241 | 0.2621 | 0.7870 | 0.1838 | 0.8954 | 0.4373 | 1.4322 | 0.3901 | 1.0375 | 0.9431 | 1.8030 | 0.1580 | 0.9376 | 0.5781 | 1.6904 | 0.2851 | 0.7943 | 0.2757 |
| 708 | Q9H9P8 | L-2-hydroxyglutarate dehydrogenase, mitochondrial (EC 1.1.99.2) (Duranin) | L2HGDH C14orf160 | 4 | 5 | 1.0375 | 0.6088 | 2.5119 | 0.1011 | 1.0093 | 0.3730 | 1.8535 | 0.1005 | 1.0375 | 0.5065 | 0.6855 | 0.7887 | 1.0666 | 0.7888 | 1.9409 | 0.2020 | 1.0864 | 0.4291 | 0.5105 | 0.9379 | 1.2589 | 0.5856 | 1.8707 | 0.1950 | 1.5276 | 0.3077 | 0.6427 | 0.4820 |
| 709 | Q16719 | Kynureninase (EC 3.7.1.3) (L-kynurenine hydrolase) | KYNU | 7 | 7 | 0.7379 | 0.1661 | 1.1803 | 0.6758 | 0.5702 | 0.0662 | 0.7311 | 0.1316 | 0.6730 | 0.1100 | 1.2023 | 0.5418 | 0.9908 | 0.9141 | 0.9290 | 0.3891 | 0.7870 | 0.1043 | 0.8790 | 0.1309 | 0.6026 | 0.0208 | 1.2942 | 0.7257 | 0.3020 | 0.0334 | 0.6730 | 0.1668 |
| 710 | Q86UP2 | Kinectin (CG-1 antigen) (Kinesin receptor) | KTN1 CG1 KIAA0004 | 6 | 5 | 1.8030 | 0.2518 | 1.0864 | 0.6625 | 0.9817 | 0.7122 | 0.9376 | 0.5653 | 1.2023 | 0.6497 | 1.0000 | 0.8337 | 1.5276 | 0.3781 | 0.8954 | 0.2976 | 1.5849 | 0.3837 | 1.0093 | 0.9773 | 0.8872 | 0.6913 | 0.8395 | 0.1214 | 0.3251 | 0.2802 | 0.9908 | 0.9581 |
| 711 | P05787 | Keratin, type II cytoskeletal 8 (Cytokeratin-8) (CK-8) (Keratin-8) (K8) (Type-II keratin Kb8) | KRT8 CYK8 | 8 | 130 | 0.5152 | 0.4335 | 0.5248 | 0.0152 | 0.4487 | 0.1024 | 0.2831 | 0.0003 | 1.0765 | 0.8464 | 1.0568 | 0.3684 | 0.5297 | 0.1238 | 1.4454 | 0.0914 | 0.9550 | 0.8019 | 0.5058 | 0.0425 | 0.5346 | 0.1629 | 1.4060 | 0.0394 | 0.4831 | 0.2453 | 1.4997 | 0.0008 |
| 712 | P08729 | Keratin, type II cytoskeletal 7 (Cytokeratin-7) (CK-7) (Keratin-7) (K7) (Sarcolectin) (Type-II keratin Kb7) | KRT7 SCL | 7 | 27 | 0.9204 | 0.6343 | 0.6918 | 0.5010 | 0.9817 | 0.6828 | 1.0666 | 0.9927 | 1.0568 | 0.9738 | 0.6368 | 0.1905 | 0.9462 | 0.6660 | 1.1695 | 0.7369 | 0.9036 | 0.5624 | 0.9036 | 0.6801 | 1.0186 | 0.9541 | 0.3837 | 0.1547 | 0.9376 | 0.7549 | 2.4210 | 0.0012 |
| 713 | P35908 | Keratin, type II cytoskeletal 2 epidermal (Cytokeratin-2e) (CK-2e) (Epithelial keratin-2e) (Keratin-2 epidermis) (Keratin-2e) (K2e) (Type-II keratin Kb2) | KRT2 KRT2A KRT2E | 147 | 4 | 1.0280 | 0.1934 | 1.0000 | 0.8580 | 0.5012 | 0.0086 | 0.8091 | 0.3458 | 2.8840 | 0.0000 | 0.9376 | 0.6235 | 1.4454 | 0.3701 | 0.8954 | 0.4405 | 1.0666 | 0.1689 | 0.8241 | 0.3952 | 2.0324 | 0.0042 | 1.0568 | 0.9780 | 1.5849 | 0.6472 | 0.7943 | 0.2664 |
| 714 | P08727 | Keratin, type I cytoskeletal 19 (Cytokeratin-19) (CK-19) (Keratin-19) (K19) | KRT19 | 26 | 16 | 1.2134 | 0.7451 | 0.3192 | 0.0112 | 0.8091 | 0.8641 | 0.9204 | 0.5501 | 0.5395 | 0.3366 | 0.1247 | 0.0014 | 1.1272 | 0.4399 | 0.3908 | 0.0040 | 2.3121 | 0.0428 | 0.3499 | 0.0030 | 3.0200 | 0.0028 | 0.7379 | 0.0406 | 1.8707 | 0.0966 | 1.1272 | 0.9022 |
| 715 | P05783 | Keratin, type I cytoskeletal 18 (Cell proliferation-inducing gene 46 protein) (Cytokeratin-18) (CK-18) (Keratin-18) (K18) | KRT18 CYK18 PIG46 | 5 | 147 | 0.1941 | 0.0706 | 1.0666 | 0.3584 | 0.1459 | 0.0294 | 0.3221 | 0.0002 | 0.3133 | 0.0847 | 1.7701 | 0.1432 | 0.1318 | 0.0284 | 2.4434 | 0.0791 | 0.1009 | 0.0221 | 0.8551 | 0.0624 | 0.2421 | 0.0664 | 1.8030 | 0.0620 | 0.3048 | 0.0896 | 2.6792 | 0.0000 |
| 716 | P13645 | Keratin, type I cytoskeletal 10 (Cytokeratin-10) (CK-10) (Keratin-10) (K10) | KRT10 KPP | 24 | 5 | 0.2489 | 0.0051 | 1.3932 | 0.5319 | 1.8030 | 0.0149 | 0.8017 | 0.6703 | 0.2512 | 0.0347 | 1.3428 | 0.5658 | 1.0666 | 0.5252 | 1.1588 | 0.7443 | 2.2491 | 0.0036 | 0.8872 | 0.8048 | 2.5351 | 0.0005 | 1.1066 | 0.8191 | 1.5996 | 0.0942 | 0.8872 | 0.8166 |
| 717 | P04264 | Keratin, type II cytoskeletal 1 (67 kDa cytokeratin) (Cytokeratin-1) (CK-1) (Hair alpha protein) (Keratin-1) (K1) (Type-II keratin Kb1) | KRT1 KRTA | 155 | 9 | 1.4322 | 0.9648 | 2.1878 | 0.1374 | 0.6138 | 0.0276 | 1.7865 | 0.3882 | 3.5975 | 0.0000 | 2.8576 | 0.0299 | 1.8535 | 0.1860 | 2.5586 | 0.1462 | 1.3428 | 0.7352 | 1.4588 | 0.5833 | 2.6792 | 0.0005 | 1.4723 | 0.6737 | 1.8197 | 0.0976 | 0.9462 | 0.8170 |
| 718 | Q14974 | Importin subunit beta-1 (Importin-90) (Karyopherin subunit beta-1) (Nuclear factor p97) (Pore targeting complex 97 kDa subunit) (PTAC97) | KPNB1 NTF97 | 9 | 12 | 0.0887 | 0.0014 | 0.8472 | 0.9335 | 0.0637 | 0.0015 | 0.9908 | 0.7559 | 0.0964 | 0.0028 | 1.2474 | 0.2605 | 0.1047 | 0.0001 | 1.5560 | 0.3418 | 0.1738 | 0.0000 | 1.2706 | 0.7992 | 0.0780 | 0.0000 | 0.5970 | 0.0627 | 0.0608 | 0.0015 | 1.1482 | 0.5239 |
| 719 | P52294 | Importin subunit alpha-5 (Karyopherin subunit alpha-1) (Nucleoprotein interactor 1) (NPI-1) (RAG cohort protein 2) (SRP1-beta) [Cleaved into: Importin subunit alpha-5, N-terminally processed] | KPNA1 RCH2 | 13 | 4 | 0.0871 | 0.0000 | 1.1376 | 0.5839 | 0.1148 | 0.0032 | 0.8790 | 0.5172 | 0.2466 | 0.0450 | 0.8166 | 0.6469 | 0.1213 | 0.0000 | 0.8872 | 0.3772 | 0.0920 | 0.0075 | 0.8318 | 0.2975 | 0.1786 | 0.0000 | 1.1376 | 0.6276 | 0.2911 | 0.0012 | 0.7727 | 0.5260 |
| 720 | O15229 | Kynurenine 3-monooxygenase (EC 1.14.13.9) (Kynurenine 3-hydroxylase) | KMO | 14 | 9 | 0.4285 | 0.4285 | 1.0666 | 0.2345 | 1.0471 | 0.7460 | 1.9409 | 0.3050 | 0.8472 | 0.4872 | 1.5417 | 0.1248 | 1.1695 | 0.6512 | 2.2491 | 0.0669 | 1.3677 | 0.3341 | 0.6546 | 0.5431 | 1.8535 | 0.0562 | 2.1086 | 0.3029 | 1.7378 | 0.2543 | 1.0864 | 0.9848 |
| 721 | P33176 | Kinesin-1 heavy chain (Conventional kinesin heavy chain) (Ubiquitous kinesin heavy chain) (UKHC) | KIF5B KNS KNS1 | 3 | 4 | 0.5346 | 0.2874 | 0.9290 | 0.4581 | 0.5861 | 0.2647 | 0.4130 | 0.0418 | 0.9376 | 0.8696 | 1.3552 | 0.2802 | 0.6081 | 0.3542 | 0.8318 | 0.6525 | 0.6855 | 0.4344 | 0.7798 | 0.2887 | 0.8017 | 0.6261 | 1.0765 | 0.9812 | 0.6138 | 0.3444 | 1.3677 | 0.3787 |
| 722 | O94874 | E3 UFM1-protein ligase 1 (EC 6.3.2.-) (Novel LZAP-binding protein) (Regulator of C53/LZAP and DDRGK1) | UFL1 KIAA0776 NLBP RCAD | 6 | 8 | 1.8365 | 0.1105 | 1.0093 | 0.7951 | 1.5996 | 0.0888 | 0.9550 | 0.6934 | 0.6918 | 0.7822 | 0.4529 | 0.1856 | 1.6749 | 0.3278 | 0.5546 | 0.2685 | 1.8197 | 0.0728 | 1.0280 | 0.6736 | 1.2359 | 0.5048 | 0.6310 | 0.3961 | 1.4723 | 0.3941 | 1.2246 | 0.1693 |
| 723 | O75153 | Clustered mitochondria protein homolog | CLUH KIAA0664 | 7 | 4 | 2.3121 | 0.2137 | 0.8954 | 0.3410 | 0.6486 | 0.4535 | 1.0765 | 0.7004 | 2.3335 | 0.2240 | 1.0093 | 0.9693 | 2.2699 | 0.2323 | 0.8472 | 0.2951 | 2.8314 | 0.1975 | 0.9290 | 0.7483 | 3.7325 | 0.1771 | 0.8710 | 0.3014 | 2.8054 | 0.2010 | 1.0666 | 0.6774 |
| 724 | A3KMH1 | von Willebrand factor A domain-containing protein 8 | VWA8 KIAA0564 | 13 | 10 | 0.9550 | 0.6777 | 0.7727 | 0.9379 | 1.0000 | 0.4747 | 0.7798 | 0.7545 | 0.9638 | 0.9131 | 0.8166 | 0.6411 | 0.9817 | 0.9481 | 0.7656 | 0.4462 | 0.9550 | 0.8056 | 1.0864 | 0.6337 | 0.8954 | 0.4057 | 1.5560 | 0.3166 | 0.9727 | 0.9115 | 0.9290 | 0.5615 |
| 725 | Q92945 | Far upstream element-binding protein 2 (FUSE-binding protein 2) (KH type-splicing regulatory protein) (KSRP) (p75) | KHSRP FUBP2 | 5 | 11 | 1.1695 | 0.3314 | 0.8091 | 0.7085 | 1.0471 | 0.7632 | 0.8710 | 0.7534 | 1.0568 | 0.7088 | 1.4454 | 0.3970 | 1.2706 | 0.1743 | 0.7656 | 0.7992 | 1.0375 | 0.7544 | 0.8395 | 0.7823 | 0.9817 | 0.9396 | 1.4060 | 0.0918 | 1.2474 | 0.1971 | 0.9290 | 0.4602 |
| 726 | P50053 | Ketohexokinase (EC 2.7.1.3) (Hepatic fructokinase) | KHK | 4 | 23 | 1.0666 | 0.4311 | 0.9120 | 0.8533 | 1.2246 | 0.3414 | 1.1803 | 0.9989 | 0.9204 | 0.6593 | 0.9290 | 0.4039 | 1.1066 | 0.5768 | 0.9462 | 0.8515 | 1.1169 | 0.3722 | 0.5861 | 0.1304 | 0.9204 | 0.4964 | 1.4588 | 0.0469 | 1.0666 | 0.4036 | 0.1977 | 0.0074 |
| 727 | Q15046 | Lysine--tRNA ligase (EC 6.1.1.6) (Lysyl-tRNA synthetase) (LysRS) | KARS KIAA0070 | 24 | 5 | 1.3183 | 0.6483 | 0.6252 | 0.1735 | 1.1482 | 0.6741 | 1.1169 | 0.5050 | 1.2474 | 0.2720 | 1.0186 | 0.6057 | 1.0471 | 0.8215 | 1.0965 | 0.4045 | 1.3552 | 0.5165 | 1.3183 | 0.4443 | 0.1570 | 0.1474 | 0.5297 | 0.1731 | 0.5012 | 0.2159 | 0.6138 | 0.2948 |
| 728 | Q63ZY3 | KN motif and ankyrin repeat domain-containing protein 2 (Ankyrin repeat domain-containing protein 25) (Matrix-remodeling-associated protein 3) (SRC-1-interacting protein) (SIP) (SRC-interacting protein) (SRC1-interacting protein) | KANK2 ANKRD25 KIAA1518 MXRA3 SIP | 18 | 6 | 0.9290 | 0.8939 | 1.4060 | 0.1286 | 0.8790 | 0.8130 | 1.1376 | 0.3532 | 0.7727 | 0.6446 | 1.3428 | 0.2271 | 0.7516 | 0.6136 | 1.0864 | 0.4847 | 0.9727 | 0.9658 | 1.0666 | 0.4852 | 0.5200 | 0.3461 | 0.9908 | 0.9584 | 0.7943 | 0.6704 | 1.1588 | 0.3341 |
| 729 | P14923 | Junction plakoglobin (Catenin gamma) (Desmoplakin III) (Desmoplakin-3) | JUP CTNNG DP3 | 7 | 11 | 1.0375 | 0.6861 | 1.7539 | 0.0222 | 1.5136 | 0.1470 | 0.8710 | 0.8487 | 1.2246 | 0.4024 | 0.7047 | 0.5093 | 1.7061 | 0.0754 | 1.1695 | 0.5751 | 1.6444 | 0.0514 | 1.8707 | 0.1240 | 1.3932 | 0.2347 | 1.2023 | 0.3854 | 1.3804 | 0.2550 | 0.9290 | 0.5159 |
| 730 | P26440 | Isovaleryl-CoA dehydrogenase, mitochondrial (IVD) (EC 1.3.8.4) | IVD | 5 | 28 | 0.9550 | 0.9001 | 0.5200 | 0.5261 | 0.8166 | 0.4244 | 0.5754 | 0.3036 | 0.9908 | 0.9945 | 0.3981 | 0.0029 | 0.6607 | 0.1373 | 0.7516 | 0.0136 | 1.0568 | 0.7898 | 0.7047 | 0.1295 | 0.7244 | 0.2509 | 0.7311 | 0.1664 | 0.9120 | 0.7438 | 0.3873 | 0.0080 |
| 731 | Q14571 | Inositol 1,4,5-trisphosphate receptor type 2 (IP3 receptor isoform 2) (IP3R 2) (InsP3R2) (Type 2 inositol 1,4,5-trisphosphate receptor) (Type 2 InsP3 receptor) | ITPR2 | 12 | 7 | 0.9638 | 0.4727 | 0.5445 | 0.0776 | 1.0765 | 0.7531 | 0.8395 | 0.2309 | 0.8551 | 0.2837 | 0.4699 | 0.0637 | 1.0568 | 0.5906 | 0.7379 | 0.1208 | 0.8241 | 0.2266 | 0.8318 | 0.3000 | 0.9290 | 0.9984 | 0.8472 | 0.2669 | 0.6918 | 0.3112 | 0.6855 | 0.1020 |
| 732 | Q14624 | Inter-alpha-trypsin inhibitor heavy chain H4 (ITI heavy chain H4) (ITI-HC4) (Inter-alpha-inhibitor heavy chain 4) (Inter-alpha-trypsin inhibitor family heavy chain-related protein) (IHRP) (Plasma kallikrein sensitive glycoprotein 120) (Gp120) (PK-120) [Cleaved into: 70 kDa inter-alpha-trypsin inhibitor heavy chain H4; 35 kDa inter-alpha-trypsin inhibitor heavy chain H4] | ITIH4 IHRP ITIHL1 PK120 PRO1851 | 30 | 16 | 0.9817 | 0.0920 | 0.4365 | 0.1454 | 0.6668 | 0.0130 | 1.0765 | 0.5819 | 0.4831 | 0.0013 | 0.6138 | 0.0062 | 1.1588 | 0.4283 | 0.6730 | 0.0027 | 1.1695 | 0.4416 | 1.2246 | 0.9000 | 0.2559 | 0.0000 | 0.5495 | 0.0827 | 0.3981 | 0.0002 | 0.6855 | 0.0693 |
| 733 | P19823 | Inter-alpha-trypsin inhibitor heavy chain H2 (ITI heavy chain H2) (ITI-HC2) (Inter-alpha-inhibitor heavy chain 2) (Inter-alpha-trypsin inhibitor complex component II) (Serum-derived hyaluronan-associated protein) (SHAP) | ITIH2 IGHEP2 | 5 | 7 | 0.8166 | 0.5152 | 0.4831 | 0.1279 | 0.5916 | 0.0756 | 1.0186 | 0.9970 | 0.4831 | 0.1082 | 0.6486 | 0.2696 | 0.2992 | 0.0165 | 0.6368 | 0.3528 | 0.8017 | 0.4197 | 0.9376 | 0.8892 | 0.5546 | 0.1506 | 0.7311 | 0.5198 | 0.8017 | 0.5195 | 0.6427 | 0.3555 |
| 734 | P19827 | Inter-alpha-trypsin inhibitor heavy chain H1 (ITI heavy chain H1) (ITI-HC1) (Inter-alpha-inhibitor heavy chain 1) (Inter-alpha-trypsin inhibitor complex component III) (Serum-derived hyaluronan-associated protein) (SHAP) | ITIH1 IGHEP1 | 13 | 10 | 0.8241 | 0.9776 | 0.7311 | 0.3766 | 0.8872 | 0.5141 | 0.9550 | 0.9613 | 1.1482 | 0.3071 | 0.8710 | 0.5767 | 0.9290 | 0.9385 | 0.8318 | 0.5698 | 0.7311 | 0.0706 | 1.0568 | 0.6683 | 0.9727 | 0.7168 | 0.9550 | 0.7939 | 0.8630 | 0.2898 | 0.7379 | 0.2816 |
| 735 | P05556 | Integrin beta-1 (Fibronectin receptor subunit beta) (Glycoprotein IIa) (GPIIA) (VLA-4 subunit beta) (CD antigen CD29) | ITGB1 FNRB MDF2 MSK12 | 7 | 15 | 0.7870 | 0.9984 | 0.8954 | 0.4358 | 1.1376 | 0.2269 | 1.9953 | 0.1126 | 1.3932 | 0.1589 | 0.9290 | 0.9754 | 0.5297 | 0.1600 | 0.7516 | 0.6685 | 1.0186 | 0.3160 | 1.6904 | 0.2613 | 1.1695 | 0.6034 | 0.9376 | 0.6034 | 1.5996 | 0.0969 | 0.9727 | 0.8867 |
| 736 | P56199 | Integrin alpha-1 (CD49 antigen-like family member A) (Laminin and collagen receptor) (VLA-1) (CD antigen CD49a) | ITGA1 | 11 | 14 | 0.7586 | 0.2391 | 0.7943 | 0.4824 | 1.2589 | 0.4320 | 2.2284 | 0.1562 | 1.2134 | 0.4033 | 1.4060 | 0.9795 | 0.8472 | 0.2296 | 2.2080 | 0.1284 | 0.6486 | 0.0650 | 1.0864 | 0.5370 | 0.2754 | 0.0850 | 1.6293 | 0.7978 | 0.8091 | 0.2121 | 1.3552 | 0.9269 |
| 737 | Q96AB3 | Isochorismatase domain-containing protein 2, mitochondrial | ISOC2 | 11 | 9 | 0.5808 | 0.0363 | 1.6596 | 0.1240 | 1.0093 | 0.6859 | 2.2080 | 0.4007 | 0.8472 | 0.3974 | 1.0186 | 0.1397 | 0.7870 | 0.4515 | 0.7311 | 0.2765 | 0.7943 | 0.1847 | 2.0512 | 0.0919 | 0.8091 | 0.6042 | 0.9204 | 0.2460 | 0.7311 | 0.2080 | 0.6486 | 0.1176 |
| 738 | Q96CN7 | Isochorismatase domain-containing protein 1 | ISOC1 CGI-111 | 8 | 9 | 0.6792 | 0.6547 | 1.9588 | 0.6368 | 1.4588 | 0.3722 | 1.6444 | 0.3719 | 0.8091 | 0.3144 | 1.4859 | 0.8022 | 1.1376 | 0.7264 | 3.6644 | 0.0153 | 1.1272 | 0.9818 | 2.0324 | 0.2946 | 1.4060 | 0.7871 | 2.3335 | 0.3196 | 0.7870 | 0.4962 | 2.6546 | 0.2695 |
| 739 | Q13576 | Ras GTPase-activating-like protein IQGAP2 | IQGAP2 | 12 | 78 | 1.2134 | 0.7930 | 1.4191 | 0.2708 | 1.9055 | 0.4299 | 1.7539 | 0.0291 | 1.3932 | 0.5556 | 2.0324 | 0.0001 | 1.2706 | 0.3782 | 1.2474 | 0.6253 | 1.6904 | 0.3417 | 1.5136 | 0.2946 | 0.2188 | 0.0281 | 1.3183 | 0.4483 | 0.3945 | 0.1665 | 0.3565 | 0.0000 |
| 740 | P46940 | Ras GTPase-activating-like protein IQGAP1 (p195) | IQGAP1 KIAA0051 | 13 | 32 | 0.9817 | 0.9544 | 0.7112 | 0.5617 | 0.7727 | 0.9361 | 1.4859 | 0.3723 | 2.3988 | 0.0715 | 1.5996 | 0.3812 | 1.4454 | 0.4650 | 0.6607 | 0.2518 | 1.8707 | 0.2268 | 1.7378 | 0.1447 | 0.7656 | 0.4541 | 0.8872 | 0.3810 | 1.5560 | 0.4532 | 1.3677 | 0.3862 |
| 741 | O00410 | Importin-5 (Imp5) (Importin subunit beta-3) (Karyopherin beta-3) (Ran-binding protein 5) (RanBP5) | IPO5 KPNB3 RANBP5 | 71 | 3 | 1.4859 | 0.2408 | 1.0375 | 0.9283 | 1.2023 | 0.8568 | 1.1695 | 0.4925 | 1.4060 | 0.5235 | 0.9817 | 0.7555 | 0.7447 | 0.0083 | 1.3428 | 0.3057 | 1.0375 | 0.2587 | 1.0280 | 0.5929 | 0.1585 | 0.0000 | 1.1376 | 0.5242 | 0.3436 | 0.0000 | 1.2023 | 0.5390 |
| 742 | Q27J81 | Inverted formin-2 (HBEBP2-binding protein C) | INF2 C14orf151 C14orf173 | 30 | 7 | 0.5970 | 0.3931 | 1.3552 | 0.4556 | 1.6596 | 0.1493 | 1.7865 | 0.1034 | 1.7539 | 0.4112 | 1.5996 | 0.1846 | 0.9290 | 0.6336 | 1.1272 | 0.6952 | 1.6596 | 0.3828 | 0.8551 | 0.8407 | 2.8314 | 0.0022 | 1.7865 | 0.2053 | 2.0701 | 0.0398 | 1.6749 | 0.3292 |
| 743 | P12268 | Inosine-5'-monophosphate dehydrogenase 2 (IMP dehydrogenase 2) (IMPD 2) (IMPDH 2) (EC 1.1.1.205) (IMPDH-II) | IMPDH2 IMPD2 | 6 | 6 | 1.5417 | 0.6583 | 0.4207 | 0.4613 | 0.5346 | 0.8511 | 0.5970 | 0.3508 | 1.4997 | 0.6918 | 0.3251 | 0.0724 | 1.2474 | 0.7271 | 0.7379 | 0.7291 | 0.5546 | 0.8167 | 1.7865 | 0.0530 | 1.7865 | 0.4195 | 1.2823 | 0.5310 | 0.9462 | 0.8842 | 0.5445 | 0.3134 |
| 744 | A1L0T0 | Acetolactate synthase-like protein (EC 2.2.1.-) (IlvB-like protein) | ILVBL AHAS | 5 | 12 | 0.9204 | 0.9129 | 2.0701 | 0.1059 | 0.6792 | 0.2399 | 1.7701 | 0.2969 | 1.1695 | 0.3605 | 1.0765 | 0.8849 | 0.7311 | 0.2080 | 0.9036 | 0.9059 | 0.8790 | 0.7373 | 1.1066 | 0.8380 | 1.0568 | 0.4714 | 1.7539 | 0.3231 | 1.0093 | 0.6481 | 1.7219 | 0.3168 |
| 745 | Q13418 | Integrin-linked protein kinase (EC 2.7.11.1) (59 kDa serine/threonine-protein kinase) (ILK-1) (ILK-2) (p59ILK) | ILK ILK1 ILK2 | 5 | 7 | 0.8790 | 0.5326 | 0.3873 | 0.0435 | 0.9462 | 0.6822 | 1.0666 | 0.8820 | 0.8166 | 0.2641 | 0.6607 | 0.1273 | 0.9462 | 0.6927 | 0.6855 | 0.3197 | 0.8630 | 0.3067 | 0.6792 | 0.0366 | 0.9550 | 0.4123 | 0.6138 | 0.1005 | 1.1169 | 0.6614 | 0.6252 | 0.0537 |
| 746 | Q12906 | Interleukin enhancer-binding factor 3 (Double-stranded RNA-binding protein 76) (DRBP76) (M-phase phosphoprotein 4) (MPP4) (Nuclear factor associated with dsRNA) (NFAR) (Nuclear factor of activated T-cells 90 kDa) (NF-AT-90) (Translational control protein 80) (TCP80) | ILF3 DRBF MPHOSPH4 NF90 | 7 | 7 | 1.1912 | 0.8830 | 1.0000 | 0.7420 | 1.4859 | 0.4297 | 0.9120 | 0.3920 | 1.7701 | 0.5697 | 0.8954 | 0.4004 | 2.3335 | 0.0388 | 1.0093 | 0.7935 | 1.3804 | 0.8612 | 0.9036 | 0.4663 | 1.2474 | 0.6702 | 0.9290 | 0.5783 | 1.4997 | 0.9125 | 1.0280 | 0.8438 |
| 747 | Q12905 | Interleukin enhancer-binding factor 2 (Nuclear factor of activated T-cells 45 kDa) | ILF2 NF45 PRO3063 | 7 | 6 | 0.7586 | 0.1242 | 0.8954 | 0.5916 | 1.2706 | 0.3143 | 1.2134 | 0.6051 | 0.7516 | 0.2264 | 1.7701 | 0.1628 | 0.6982 | 0.3555 | 1.3552 | 0.6452 | 0.8954 | 0.5742 | 1.5849 | 0.3765 | 1.0568 | 0.5355 | 1.5560 | 0.5712 | 0.8790 | 0.5643 | 1.4859 | 0.2117 |
| 748 | Q969P0 | Immunoglobulin superfamily member 8 (IgSF8) (CD81 partner 3) (Glu-Trp-Ile EWI motif-containing protein 2) (EWI-2) (Keratinocytes-associated transmembrane protein 4) (KCT-4) (LIR-D1) (Prostaglandin regulatory-like protein) (PGRL) (CD antigen CD316) | IGSF8 CD81P3 EWI2 KCT4 | 10 | 6 | 0.4169 | 0.2017 | 0.3945 | 0.2700 | 1.2246 | 0.9594 | 1.0093 | 0.4954 | 0.7798 | 0.7227 | 0.6081 | 0.5073 | 0.9036 | 0.6635 | 0.5395 | 0.2226 | 1.1376 | 0.9187 | 0.1259 | 0.0824 | 2.0324 | 0.1001 | 0.7244 | 0.2486 | 1.1066 | 0.7959 | 0.5808 | 0.7016 |
| 749 | P01834 | Ig kappa chain C region | IGKC | 6 | 34 | 1.1482 | 0.8917 | 1.5560 | 0.5156 | 1.2359 | 0.4980 | 0.7727 | 0.6108 | 1.0765 | 0.6161 | 0.2443 | 0.6522 | 1.2134 | 0.5558 | 1.0864 | 0.9946 | 1.1272 | 0.4690 | 1.4060 | 0.6558 | 1.5996 | 0.2011 | 0.8017 | 0.7415 | 1.2134 | 0.7334 | 1.9588 | 0.3658 |
| 750 | P01857 | Ig gamma-1 chain C region | IGHG1 | 4 | 47 | 1.2942 | 0.4671 | 0.8790 | 0.9605 | 0.9120 | 0.5740 | 0.4699 | 0.1314 | 1.1588 | 0.5773 | 0.2512 | 0.0092 | 1.1066 | 0.5030 | 1.0375 | 0.6722 | 1.2359 | 0.2070 | 0.8551 | 0.4519 | 0.8241 | 0.6664 | 0.4875 | 0.0512 | 1.0666 | 0.5383 | 2.4210 | 0.0239 |
| 751 | P01871 | Ig mu chain C region | IGHM | 19 | 10 | 1.1066 | 0.4627 | 0.3281 | 0.0765 | 1.1588 | 0.3730 | 0.7112 | 0.8152 | 1.1588 | 0.4058 | 0.5970 | 0.0735 | 1.2359 | 0.3776 | 0.5546 | 0.1538 | 1.0375 | 0.9889 | 0.4325 | 0.0080 | 1.0093 | 0.7729 | 0.6855 | 0.6852 | 1.1066 | 0.5081 | 0.6982 | 0.2537 |
| 752 | P01861 | Ig gamma-4 chain C region | IGHG4 | 56 | 22 | 0.2443 | 0.0143 | 0.0453 | 0.1116 | 0.6730 | 0.2209 | 0.5297 | 0.3359 | 0.9204 | 0.6389 | 0.4207 | 0.2970 | 0.7047 | 0.1664 | 0.4613 | 0.4097 | 0.0787 | 0.0088 | 0.2249 | 0.2132 | 0.9817 | 0.8137 | 0.1486 | 0.1716 | 1.0666 | 0.5689 | 0.0625 | 0.1380 |
| 753 | P01860 | Ig gamma-3 chain C region (HDC) (Heavy chain disease protein) | IGHG3 | 9 | 24 | 0.1614 | 0.0101 | 1.3677 | 0.4092 | 0.9462 | 0.5897 | 0.0625 | 0.0099 | 1.0000 | 0.2441 | 1.2706 | 0.8672 | 0.4613 | 0.0963 | 0.2655 | 0.0143 | 0.1803 | 0.0037 | 0.5916 | 0.1553 | 0.8710 | 0.7630 | 0.2858 | 0.0132 | 0.6792 | 0.2553 | 0.9817 | 0.8525 |
| 754 | P01859 | Ig gamma-2 chain C region | IGHG2 | 22 | 39 | 0.5058 | 0.3395 | 0.3133 | 0.4760 | 0.2489 | 0.1801 | 0.1738 | 0.3256 | 0.4325 | 0.2949 | 0.3048 | 0.3779 | 0.8395 | 0.8305 | 1.0186 | 0.9571 | 0.2270 | 0.1686 | 0.6730 | 0.8534 | 0.3020 | 0.2028 | 0.4831 | 0.4870 | 0.4487 | 0.2935 | 1.0093 | 0.7947 |
| 755 | P01877 | Ig alpha-2 chain C region | IGHA2 | 29 | 16 | 0.8551 | 0.6005 | 1.1066 | 0.9118 | 1.2474 | 0.8770 | 0.8395 | 0.5661 | 0.3499 | 0.2967 | 0.2489 | 0.1584 | 1.1912 | 0.8009 | 0.9727 | 0.3878 | 0.6252 | 0.4217 | 1.2246 | 0.8244 | 0.4130 | 0.3830 | 0.7586 | 0.4263 | 0.4920 | 0.3381 | 1.6596 | 0.5970 |
| 756 | P01876 | Ig alpha-1 chain C region | IGHA1 | 47 | 16 |  |  | 0.3373 | 0.0765 |  |  | 0.6138 | 0.2094 |  |  | 0.1180 | 0.0078 |  |  | 0.5702 | 0.1217 |  |  | 0.4742 | 0.1139 |  |  | 0.2729 | 0.0170 |  |  | 1.0471 | 0.6264 |
| 757 | O14879 | Interferon-induced protein with tetratricopeptide repeats 3 (IFIT-3) (CIG49) (ISG-60) (Interferon-induced 60 kDa protein) (IFI-60K) (Interferon-induced protein with tetratricopeptide repeats 4) (IFIT-4) (Retinoic acid-induced gene G protein) (P60) (RIG-G) | IFIT3 CIG-49 IFI60 IFIT4 ISG60 | 43 | 3 |  |  | 1.5276 | 0.6787 |  |  | 0.6855 | 0.3409 |  |  | 3.3419 | 0.1225 |  |  | 0.7379 | 0.6807 |  |  | 2.6792 | 0.2150 |  |  | 0.8954 | 0.7590 |  |  | 0.4055 | 0.4631 |
| 758 | Q13907 | Isopentenyl-diphosphate Delta-isomerase 1 (EC 5.3.3.2) (Isopentenyl pyrophosphate isomerase 1) (IPP isomerase 1) (IPPI1) | IDI1 | 17 | 4 | 0.3048 | 0.1341 | 1.1066 | 0.7348 | 0.6855 | 0.3905 | 1.1169 | 0.5089 | 0.7586 | 0.6364 | 1.1169 | 0.4857 | 0.4246 | 0.1613 | 1.0965 | 0.5252 | 0.0752 | 0.0520 | 1.4997 | 0.2243 | 1.1376 | 0.7376 | 1.1066 | 0.7151 | 0.3873 | 0.1952 | 0.9204 | 0.7591 |
| 759 | P50213 | Isocitrate dehydrogenase [NAD] subunit alpha, mitochondrial (EC 1.1.1.41) (Isocitric dehydrogenase subunit alpha) (NAD(+)-specific ICDH subunit alpha) | IDH3A | 3 | 4 | 1.0568 | 0.9921 | 0.9550 | 0.5989 | 1.1588 | 0.9566 | 1.0568 | 0.6867 | 0.2884 | 0.2840 | 1.0765 | 0.2614 | 0.7727 | 0.7203 | 1.0186 | 0.9860 | 1.0000 | 0.7004 | 1.0568 | 0.6011 | 0.8472 | 0.4904 | 0.9817 | 0.6040 | 0.7870 | 0.4963 | 1.0568 | 0.6948 |
| 760 | P48735 | Isocitrate dehydrogenase [NADP], mitochondrial (IDH) (EC 1.1.1.42) (ICD-M) (IDP) (NADP(+)-specific ICDH) (Oxalosuccinate decarboxylase) | IDH2 | 4 | 36 | 0.9204 | 0.8024 | 0.4786 | 0.0003 | 1.3932 | 0.6797 | 0.3499 | 0.0000 | 2.7290 | 0.0677 | 0.3076 | 0.0000 | 2.1281 | 0.1625 | 1.0375 | 0.8617 | 2.5823 | 0.0225 | 1.1912 | 0.2208 | 0.6855 | 0.6966 | 0.7311 | 0.0373 | 1.4859 | 0.1267 | 1.0000 | 0.7814 |
| 761 | O75874 | Isocitrate dehydrogenase [NADP] cytoplasmic (IDH) (EC 1.1.1.42) (Cytosolic NADP-isocitrate dehydrogenase) (IDP) (NADP(+)-specific ICDH) (Oxalosuccinate decarboxylase) | IDH1 PICD | 3 | 80 | 0.6792 | 0.7450 | 0.6138 | 0.0313 | 0.4207 | 0.1473 | 0.9036 | 0.3212 | 0.5702 | 0.1811 | 0.6855 | 0.0583 | 1.4859 | 0.1590 | 1.3804 | 0.3392 | 1.0666 | 0.5276 | 0.9290 | 0.2294 | 0.9550 | 0.5442 | 1.7219 | 0.0776 | 1.2823 | 0.3309 | 0.2938 | 0.0010 |
| 762 | P05362 | Intercellular adhesion molecule 1 (ICAM-1) (Major group rhinovirus receptor) (CD antigen CD54) | ICAM1 | 43 | 4 | 0.4920 | 0.0004 | 0.4656 | 0.0986 | 0.8790 | 0.1502 | 1.0375 | 0.3124 | 0.3436 | 0.0000 | 0.5598 | 0.1263 | 1.6904 | 0.0003 | 0.9204 | 0.3279 | 0.2938 | 0.0000 | 0.8017 | 0.1858 | 0.2443 | 0.0000 | 0.1459 | 0.0722 | 0.3373 | 0.0000 | 0.3767 | 0.0152 |
| 763 | Q5T440 | Putative transferase CAF17, mitochondrial (EC 2.1.-.-) (Iron-sulfur cluster assembly factor homolog) | IBA57 C1orf69 | 76 | 5 | 0.5970 | 0.0001 | 0.8710 | 0.5541 | 0.9036 | 0.1110 | 0.7112 | 0.1538 | 0.9638 | 0.7445 | 0.9550 | 0.3696 | 0.7798 | 0.0088 | 1.1482 | 0.5786 | 0.6310 | 0.0001 | 0.7178 | 0.2535 | 0.1159 | 0.0000 | 0.7870 | 0.1963 | 0.3597 | 0.0000 | 0.9462 | 0.7646 |
| 764 | Q9NSE4 | Isoleucine--tRNA ligase, mitochondrial (EC 6.1.1.5) (Isoleucyl-tRNA synthetase) (IleRS) | IARS2 | 4 | 13 | 0.9817 | 0.4589 | 0.8630 | 0.3760 | 1.0471 | 0.7243 | 0.8091 | 0.1846 | 0.8790 | 0.5115 | 0.3698 | 0.0138 | 0.8166 | 0.1694 | 0.5754 | 0.1844 | 0.7311 | 0.2982 | 0.7178 | 0.0927 | 1.1066 | 0.5394 | 0.6368 | 0.1397 | 0.8241 | 0.2622 | 0.5105 | 0.0409 |
| 765 | P41252 | Isoleucine--tRNA ligase, cytoplasmic (EC 6.1.1.5) (Isoleucyl-tRNA synthetase) (IRS) (IleRS) | IARS | 4 | 7 | 0.9290 | 0.7195 | 1.3932 | 0.2363 | 0.9290 | 0.8790 | 0.6607 | 0.4196 | 0.9727 | 0.7894 | 1.3677 | 0.2325 | 1.0568 | 0.6768 | 0.6668 | 0.5830 | 0.9462 | 0.8109 | 0.8630 | 0.8935 | 0.7870 | 0.3194 | 0.7178 | 0.6002 | 1.0471 | 0.9907 | 1.5704 | 0.2267 |
| 766 | Q2TAA2 | Isoamyl acetate-hydrolyzing esterase 1 homolog (EC 3.1.-.-) | IAH1 | 13 | 3 | 1.2359 | 0.7811 | 0.7870 | 0.4444 | 0.9462 | 0.9334 | 0.7586 | 0.3904 | 0.7047 | 0.1539 | 0.9727 | 0.9514 | 0.9817 | 0.5727 | 0.6486 | 0.2088 | 1.4322 | 0.3989 | 0.9376 | 0.8534 | 0.4742 | 0.0711 | 0.9290 | 0.8207 | 0.4446 | 0.0381 | 0.7112 | 0.3019 |
| 767 | Q9Y4L1 | Hypoxia up-regulated protein 1 (150 kDa oxygen-regulated protein) (ORP-150) (170 kDa glucose-regulated protein) (GRP-170) | HYOU1 GRP170 ORP150 | 3 | 34 | 0.9550 | 0.7721 | 1.1272 | 0.6926 | 1.0471 | 0.9067 | 0.8091 | 0.4560 | 0.3048 | 0.3106 | 0.8551 | 0.6557 | 1.0568 | 0.8217 | 1.1272 | 0.9508 | 0.9462 | 0.7434 | 1.7378 | 0.0598 | 1.4723 | 0.1052 | 1.1803 | 0.6972 | 1.3183 | 0.2117 | 1.0186 | 0.8893 |
| 768 | Q7Z6Z7 | E3 ubiquitin-protein ligase HUWE1 (EC 6.3.2.-) (ARF-binding protein 1) (ARF-BP1) (HECT, UBA and WWE domain-containing protein 1) (Homologous to E6AP carboxyl terminus homologous protein 9) (HectH9) (Large structure of UREB1) (LASU1) (Mcl-1 ubiquitin ligase E3) (Mule) (Upstream regulatory element-binding protein 1) (URE-B1) (URE-binding protein 1) | HUWE1 KIAA0312 KIAA1578 UREB1 HSPC272 | 3 | 6 | 0.9727 | 0.8797 | 5.9704 | 0.3902 | 0.9120 | 0.8207 | 0.8710 | 0.5982 | 0.8790 | 0.6381 | 3.4041 | 0.4615 | 1.0000 | 0.9014 | 0.9462 | 0.7177 | 0.8710 | 0.2943 | 1.1169 | 0.5160 | 1.0666 | 0.7696 | 10.7647 | 0.3225 | 0.9204 | 0.8072 | 1.8365 | 0.5308 |
| 769 | Q9BUP3 | Oxidoreductase HTATIP2 (EC 1.1.1.-) (30 kDa HIV-1 TAT-interacting protein) (HIV-1 TAT-interactive protein 2) | HTATIP2 CC3 TIP30 | 37 | 3 | 0.7311 | 0.2276 | 0.8395 | 0.4587 | 1.1912 | 0.3619 | 1.1588 | 0.4186 | 0.7311 | 0.1362 | 1.3062 | 0.1957 | 1.8535 | 0.0074 | 0.9376 | 0.8227 | 0.6546 | 0.0613 | 1.0765 | 0.4462 | 1.5996 | 0.0445 | 1.0186 | 0.9744 | 0.9204 | 0.1817 | 1.1169 | 0.6789 |
| 770 | P98160 | Basement membrane-specific heparan sulfate proteoglycan core protein (HSPG) (Perlecan) (PLC) [Cleaved into: Endorepellin; LG3 peptide] | HSPG2 | 5 | 46 | 0.8091 | 0.5153 | 0.4055 | 0.0014 | 0.5152 | 0.5774 | 1.4723 | 0.0436 | 1.1482 | 0.4498 | 0.7586 | 0.4225 | 0.3873 | 0.7693 | 0.5861 | 0.0111 | 0.8318 | 0.5133 | 0.4613 | 0.0032 | 1.5849 | 0.4328 | 0.4169 | 0.0003 | 0.6792 | 0.5264 | 0.7586 | 0.1729 |
| 771 | P61604 | 10 kDa heat shock protein, mitochondrial (Hsp10) (10 kDa chaperonin) (Chaperonin 10) (CPN10) (Early-pregnancy factor) (EPF) | HSPE1 | 4 | 15 | 1.1376 | 0.2905 | 1.2359 | 0.4540 | 1.1695 | 0.3590 | 1.7865 | 0.1448 | 1.0375 | 0.8876 | 1.3552 | 0.3823 | 1.2023 | 0.2458 | 1.6293 | 0.1677 | 1.3305 | 0.1776 | 1.6596 | 0.2239 | 1.1482 | 0.7089 | 1.8535 | 0.0607 | 0.9817 | 0.6726 | 1.1912 | 0.9534 |
| 772 | P10809 | 60 kDa heat shock protein, mitochondrial (60 kDa chaperonin) (Chaperonin 60) (CPN60) (Heat shock protein 60) (HSP-60) (Hsp60) (HuCHA60) (Mitochondrial matrix protein P1) (P60 lymphocyte protein) | HSPD1 HSP60 | 51 | 131 | 0.5754 | 0.1290 | 0.9290 | 0.4419 | 1.3552 | 0.0058 | 1.1912 | 0.7637 | 0.4656 | 0.0284 | 1.5704 | 0.5046 | 0.4285 | 0.0234 | 1.1912 | 0.3912 | 1.0864 | 0.2048 | 1.0471 | 0.2300 | 0.9817 | 0.3949 | 1.4859 | 0.1651 | 0.9120 | 0.7616 | 1.1376 | 0.6036 |
| 773 | P04792 | Heat shock protein beta-1 (HspB1) (28 kDa heat shock protein) (Estrogen-regulated 24 kDa protein) (Heat shock 27 kDa protein) (HSP 27) (Stress-responsive protein 27) (SRP27) | HSPB1 HSP27 HSP28 | 20 | 24 | 2.1281 | 0.1675 | 2.3335 | 0.3680 | 1.2589 | 0.8855 | 3.1046 | 0.0686 | 1.2589 | 0.9769 | 5.1051 | 0.0008 | 2.9648 | 0.0008 | 3.5318 | 0.0193 | 2.5823 | 0.0064 | 1.6596 | 0.6566 | 0.8630 | 0.3465 | 2.3988 | 0.2619 | 0.9036 | 0.2750 | 2.4434 | 0.1981 |
| 774 | P38646 | Stress-70 protein, mitochondrial (75 kDa glucose-regulated protein) (GRP-75) (Heat shock 70 kDa protein 9) (Mortalin) (MOT) (Peptide-binding protein 74) (PBP74) | HSPA9 GRP75 HSPA9B mt-HSP70 | 178 | 50 | 0.6918 | 0.1670 | 0.4831 | 0.0123 | 0.8790 | 0.6077 | 0.7516 | 0.0049 | 0.8017 | 0.5963 | 1.2359 | 0.5266 | 1.9588 | 0.0001 | 1.2706 | 0.8992 | 1.7865 | 0.0097 | 1.6293 | 0.5376 | 0.6194 | 0.0031 | 1.2706 | 0.5481 | 0.3981 | 0.0000 | 0.5297 | 0.0005 |
| 775 | P11142 | Heat shock cognate 71 kDa protein (Heat shock 70 kDa protein 8) (Lipopolysaccharide-associated protein 1) (LAP-1) (LPS-associated protein 1) | HSPA8 HSC70 HSP73 HSPA10 | 22 | 78 | 1.5849 | 0.7206 | 1.0186 | 0.7731 | 1.8880 | 0.3881 | 0.7178 | 0.1736 | 3.2810 | 0.0119 | 1.7219 | 0.0828 | 1.6444 | 0.3814 | 2.0324 | 0.0127 | 2.3121 | 0.2035 | 1.7865 | 0.0615 | 2.1281 | 0.2380 | 1.5996 | 0.2850 | 1.5560 | 0.7282 | 1.7539 | 0.0287 |
| 776 | P11021 | 78 kDa glucose-regulated protein (GRP-78) (Endoplasmic reticulum lumenal Ca(2+)-binding protein grp78) (Heat shock 70 kDa protein 5) (Immunoglobulin heavy chain-binding protein) (BiP) | HSPA5 GRP78 | 55 | 70 | 1.2246 | 0.9544 | 1.0000 | 0.7853 | 1.0965 | 0.4484 | 0.8091 | 0.3524 | 1.0375 | 0.2820 | 1.2589 | 0.2240 | 1.3428 | 0.1101 | 0.7047 | 0.0778 | 0.9908 | 0.4006 | 1.6749 | 0.0034 | 0.8710 | 0.1056 | 1.4723 | 0.0482 | 0.6427 | 0.0482 | 1.0765 | 0.3353 |
| 777 | P34932 | Heat shock 70 kDa protein 4 (HSP70RY) (Heat shock 70-related protein APG-2) | HSPA4 APG2 | 88 | 8 | 0.9727 | 0.1003 | 0.6026 | 0.1810 | 1.3804 | 0.4813 | 0.7727 | 0.3162 | 2.3988 | 0.0034 | 0.7727 | 0.3798 | 1.8535 | 0.2275 | 1.0965 | 0.7872 | 0.9204 | 0.1028 | 0.8472 | 0.3302 | 1.8880 | 0.0363 | 0.6252 | 0.0107 | 1.6596 | 0.6510 | 0.6138 | 0.3019 |
| 778 | P0DMV9 | Heat shock 70 kDa protein 1B (Heat shock 70 kDa protein 2) (HSP70-2) (HSP70.2) | HSPA1B | 96 | 52 | 0.9290 | 0.0531 | 1.1912 | 0.9463 | 1.4588 | 0.6651 | 1.4322 | 0.4981 | 1.7061 | 0.0294 | 1.1912 | 0.4467 | 3.1915 | 0.0000 | 1.2589 | 0.5929 | 1.1066 | 0.7756 | 1.4588 | 0.5534 | 2.3550 | 0.0001 | 1.4454 | 0.5676 | 1.5704 | 0.3993 | 0.8954 | 0.1372 |
| 779 | P14625 | Endoplasmin (94 kDa glucose-regulated protein) (GRP-94) (Heat shock protein 90 kDa beta member 1) (Tumor rejection antigen 1) (gp96 homolog) | HSP90B1 GRP94 TRA1 | 14 | 83 | 0.8395 | 0.6497 | 0.7943 | 0.4237 | 1.3677 | 0.6247 | 1.1272 | 0.6155 | 1.0093 | 0.8961 | 0.9908 | 0.5341 | 1.2023 | 0.7235 | 0.7516 | 0.0613 | 0.6427 | 0.5912 | 1.7061 | 0.0018 | 1.8880 | 0.0512 | 0.9908 | 0.4931 | 1.7539 | 0.1016 | 0.9908 | 0.7741 |
| 780 | P08238 | Heat shock protein HSP 90-beta (HSP 90) (Heat shock 84 kDa) (HSP 84) (HSP84) | HSP90AB1 HSP90B HSPC2 HSPCB | 57 | 77 | 0.5012 | 0.0016 | 0.5395 | 0.0921 | 1.7539 | 0.5017 | 0.5200 | 0.1263 | 1.6144 | 0.5973 | 1.1376 | 0.9192 | 0.9204 | 0.0293 | 0.9462 | 0.8922 | 1.9770 | 0.2069 | 1.0471 | 0.9616 | 1.0666 | 0.0094 | 0.5297 | 0.0564 | 0.8017 | 0.0284 | 1.0000 | 0.4801 |
| 781 | P07900 | Heat shock protein HSP 90-alpha (Heat shock 86 kDa) (HSP 86) (HSP86) (Lipopolysaccharide-associated protein 2) (LAP-2) (LPS-associated protein 2) (Renal carcinoma antigen NY-REN-38) | HSP90AA1 HSP90A HSPC1 HSPCA | 106 | 76 | 0.7379 | 0.0082 | 0.7727 | 0.9389 | 1.4454 | 0.3843 | 0.7798 | 0.5028 | 0.6982 | 0.2487 | 1.3932 | 0.0712 | 2.5586 | 0.0000 | 0.9290 | 0.9953 | 1.4454 | 0.5938 | 1.3428 | 0.1723 | 1.5560 | 0.2211 | 0.8091 | 0.8756 | 0.5346 | 0.0000 | 1.4997 | 0.1366 |
| 782 | Q6YN16 | Hydroxysteroid dehydrogenase-like protein 2 (EC 1.-.-.-) (Short chain dehydrogenase/reductase family 13C member 1) | HSDL2 C9orf99 SDR13C1 | 91 | 16 | 0.3802 | 0.0425 | 1.9588 | 0.8691 | 1.1066 | 0.6632 | 1.6904 | 0.8911 | 0.8318 | 0.5478 | 2.9923 | 0.0902 | 1.3428 | 0.5129 | 3.5318 | 0.0187 | 0.5012 | 0.1967 | 2.7290 | 0.1489 | 2.1677 | 0.0090 | 2.6303 | 0.2837 | 1.0864 | 0.9354 | 1.7865 | 0.2966 |
| 783 | Q92506 | Estradiol 17-beta-dehydrogenase 8 (EC 1.1.1.62) (17-beta-hydroxysteroid dehydrogenase 8) (17-beta-HSD 8) (3-oxoacyl-[acyl-carrier-protein] reductase) (EC 1.1.1.-) (Protein Ke6) (Ke-6) (Really interesting new gene 2 protein) (Short chain dehydrogenase/reductase family 30C member 1) (Testosterone 17-beta-dehydrogenase 8) (EC 1.1.1.239) | HSD17B8 FABGL HKE6 RING2 SDR30C1 | 89 | 8 | 0.6792 | 0.6325 | 1.5849 | 0.4270 | 1.2474 | 0.7720 | 2.1677 | 0.2322 | 1.2134 | 0.3494 | 2.0324 | 0.4289 | 1.3932 | 0.3901 | 1.9231 | 0.3595 | 1.0186 | 0.5588 | 1.6444 | 0.7394 | 1.9055 | 0.0226 | 1.4997 | 0.7534 | 1.0765 | 0.7473 | 0.7870 | 0.5839 |
| 784 | O14756 | 17-beta-hydroxysteroid dehydrogenase type 6 (17-beta-HSD 6) (17-beta-HSD6) (EC 1.1.1.105) (EC 1.1.1.239) (EC 1.1.1.62) (3-alpha->beta-hydroxysteroid epimerase) (3-alpha->beta-HSE) (Oxidative 3-alpha hydroxysteroid dehydrogenase) (Short chain dehydrogenase/reductase family 9C member 6) | HSD17B6 RODH SDR9C6 | 22 | 26 | 2.1086 | 0.8564 | 0.4055 | 0.0178 | 1.2023 | 0.3399 | 1.3428 | 0.7055 | 2.3121 | 0.3761 | 0.3664 | 0.0025 | 3.1915 | 0.1393 | 0.4055 | 0.0001 | 2.4210 | 0.3238 | 0.5346 | 0.0333 | 1.3552 | 0.9489 | 0.4169 | 0.0066 | 1.7865 | 0.8563 | 0.2208 | 0.0000 |
| 785 | P51659 | Peroxisomal multifunctional enzyme type 2 (MFE-2) (17-beta-hydroxysteroid dehydrogenase 4) (17-beta-HSD 4) (D-bifunctional protein) (DBP) (Multifunctional protein 2) (MPF-2) (Short chain dehydrogenase/reductase family 8C member 1) [Cleaved into: (3R)-hydroxyacyl-CoA dehydrogenase (EC 1.1.1.n12); Enoyl-CoA hydratase 2 (EC 4.2.1.107) (EC 4.2.1.119) (3-alpha,7-alpha,12-alpha-trihydroxy-5-beta-cholest-24-enoyl-CoA hydratase)] | HSD17B4 EDH17B4 SDR8C1 | 10 | 68 | 1.8365 | 0.2285 | 1.7378 | 0.0299 | 0.8551 | 0.7794 | 0.6918 | 0.4922 | 0.9204 | 0.9076 | 2.1478 | 0.0465 | 1.5136 | 0.3573 | 3.8019 | 0.0000 | 1.4454 | 0.5056 | 2.3988 | 0.0003 | 0.2884 | 0.0347 | 1.2134 | 0.7626 | 0.4966 | 0.0873 | 2.3550 | 0.0000 |
| 786 | P37059 | Estradiol 17-beta-dehydrogenase 2 (EC 1.1.1.62) (17-beta-hydroxysteroid dehydrogenase type 2) (17-beta-HSD 2) (20 alpha-hydroxysteroid dehydrogenase) (20-alpha-HSD) (E2DH) (Microsomal 17-beta-hydroxysteroid dehydrogenase) (Short chain dehydrogenase/reductase family 9C member 2) (Testosterone 17-beta-dehydrogenase) (EC 1.1.1.239) | HSD17B2 EDH17B2 SDR9C2 | 21 | 9 | 0.6982 | 0.2762 | 0.8551 | 0.5004 | 0.6138 | 0.2034 | 1.3804 | 0.2671 | 0.5861 | 0.0279 | 0.5916 | 0.0096 | 1.1695 | 0.2500 | 0.7586 | 0.1042 | 0.6546 | 0.0186 | 0.5012 | 0.0617 | 0.1368 | 0.0000 | 0.5058 | 0.0172 | 0.2805 | 0.0001 | 0.5445 | 0.0492 |
| 787 | Q7Z5P4 | 17-beta-hydroxysteroid dehydrogenase 13 (17-beta-HSD 13) (EC 1.1.-.-) (Short chain dehydrogenase/reductase family 16C member 3) (Short-chain dehydrogenase/reductase 9) | HSD17B13 SCDR9 SDR16C3 HMFN0376 UNQ497/PRO1014 | 77 | 25 | 1.5704 | 0.5225 | 0.5916 | 0.3159 | 1.4997 | 0.1759 | 1.9409 | 0.0000 | 3.5975 | 0.0000 | 0.1888 | 0.0001 | 1.6293 | 0.2075 | 0.8166 | 0.7855 | 3.8371 | 0.0000 | 0.5495 | 0.0475 | 2.1281 | 0.0002 | 0.9908 | 0.5299 | 2.3988 | 0.0004 | 0.1127 | 0.0000 |
| 788 | Q53GQ0 | Very-long-chain 3-oxoacyl-CoA reductase (EC 1.1.1.330) (17-beta-hydroxysteroid dehydrogenase 12) (17-beta-HSD 12) (3-ketoacyl-CoA reductase) (KAR) (Estradiol 17-beta-dehydrogenase 12) (EC 1.1.1.62) (Short chain dehydrogenase/reductase family 12C member 1) | HSD17B12 SDR12C1 | 10 | 8 | 1.6144 | 0.1667 | 0.6194 | 0.3445 | 0.9817 | 0.9835 | 0.7656 | 0.6334 | 0.3733 | 0.1247 | 0.8091 | 0.2309 | 2.1086 | 0.0115 | 1.4859 | 0.4426 | 1.7539 | 0.1338 | 1.4997 | 0.1724 | 1.6444 | 0.1493 | 0.6855 | 0.2160 | 1.2023 | 0.3711 | 0.6138 | 0.0351 |
| 789 | Q8NBQ5 | Estradiol 17-beta-dehydrogenase 11 (EC 1.1.1.62) (17-beta-hydroxysteroid dehydrogenase 11) (17-beta-HSD 11) (17bHSD11) (17betaHSD11) (17-beta-hydroxysteroid dehydrogenase XI) (17-beta-HSD XI) (17betaHSDXI) (Cutaneous T-cell lymphoma-associated antigen HD-CL-03) (CTCL-associated antigen HD-CL-03) (Dehydrogenase/reductase SDR family member 8) (Retinal short-chain dehydrogenase/reductase 2) (retSDR2) (Short chain dehydrogenase/reductase family 16C member 2) | HSD17B11 DHRS8 PAN1B SDR16C2 PSEC0029 UNQ207/PRO233 | 19 | 9 | 0.4656 | 0.0071 | 0.5861 | 0.1561 | 1.0375 | 0.7647 | 1.5849 | 0.1012 | 0.5105 | 0.0190 | 0.1786 | 0.0044 | 1.1803 | 0.3541 | 0.3837 | 0.0069 | 0.4285 | 0.0001 | 0.4207 | 0.1083 | 0.0560 | 0.0000 | 0.4406 | 0.0249 | 0.1294 | 0.0000 | 0.4699 | 0.0220 |
| 790 | Q99714 | 3-hydroxyacyl-CoA dehydrogenase type-2 (EC 1.1.1.35) (17-beta-hydroxysteroid dehydrogenase 10) (17-beta-HSD 10) (EC 1.1.1.51) (3-hydroxy-2-methylbutyryl-CoA dehydrogenase) (EC 1.1.1.178) (3-hydroxyacyl-CoA dehydrogenase type II) (Endoplasmic reticulum-associated amyloid beta-peptide-binding protein) (Mitochondrial ribonuclease P protein 2) (Mitochondrial RNase P protein 2) (Short chain dehydrogenase/reductase family 5C member 1) (Short-chain type dehydrogenase/reductase XH98G2) (Type II HADH) | HSD17B10 ERAB HADH2 MRPP2 SCHAD SDR5C1 XH98G2 | 11 | 45 | 1.4997 | 0.8249 | 3.4995 | 0.0687 | 1.0186 | 0.7255 | 3.4356 | 0.1410 | 1.3062 | 0.9462 | 3.5318 | 0.2186 | 1.7865 | 0.3645 | 4.5709 | 0.0259 | 1.7539 | 0.3111 | 2.9107 | 0.4935 | 1.5276 | 0.7096 | 3.4995 | 0.2414 | 0.7112 | 0.6112 | 1.7865 | 0.7445 |
| 791 | P28845 | Corticosteroid 11-beta-dehydrogenase isozyme 1 (EC 1.1.1.146) (11-beta-hydroxysteroid dehydrogenase 1) (11-DH) (11-beta-HSD1) (Short chain dehydrogenase/reductase family 26C member 1) | HSD11B1 HSD11 HSD11L SDR26C1 | 11 | 13 | 1.1803 | 0.8389 | 0.6486 | 0.3960 | 0.4487 | 0.1215 | 1.0280 | 0.9257 | 0.5248 | 0.0263 | 1.1066 | 0.8170 | 0.8710 | 0.3381 | 1.2246 | 0.4625 | 1.1272 | 0.9718 | 0.4920 | 0.1641 | 0.3499 | 0.0103 | 0.3467 | 0.4914 | 0.5861 | 0.0690 | 0.4875 | 0.1420 |
| 792 | P52758 | Ribonuclease UK114 (EC 3.1.-.-) (14.5 kDa translational inhibitor protein) (p14.5) (Heat-responsive protein 12) (UK114 antigen homolog) | HRSP12 PSP | 45 | 34 | 2.5823 | 0.0278 | 5.2481 | 0.1588 | 2.1478 | 0.1193 | 7.1121 | 0.0406 | 2.3335 | 0.0363 | 2.4434 | 0.7429 | 2.7797 | 0.0113 | 5.6494 | 0.0317 | 1.4723 | 0.2294 | 3.9084 | 0.3707 | 0.4786 | 0.0127 | 6.0256 | 0.0087 | 1.0864 | 0.2629 | 1.8365 | 0.9855 |
| 793 | P04196 | Histidine-rich glycoprotein (Histidine-proline-rich glycoprotein) (HPRG) | HRG | 10 | 3 | 0.9817 | 0.8064 | 0.2704 | 0.1201 | 0.7870 | 0.3392 | 0.5152 | 0.1815 | 0.6368 | 0.1129 | 0.4365 | 0.1760 | 0.7516 | 0.5109 | 0.4365 | 0.3651 | 0.5395 | 0.1494 | 0.4487 | 0.1608 | 0.3221 | 0.0017 | 0.8241 | 0.5670 | 0.4920 | 0.0446 | 0.7943 | 0.5588 |
| 794 | P02790 | Hemopexin (Beta-1B-glycoprotein) | HPX | 46 | 10 | 2.8054 | 0.4522 | 0.3837 | 0.0082 | 2.7542 | 0.0768 | 0.5546 | 0.0429 | 3.2509 | 0.2529 | 0.9204 | 0.5448 | 4.2073 | 0.0191 | 0.5970 | 0.0491 | 2.6546 | 0.3841 | 1.0568 | 0.8165 | 0.1738 | 0.0044 | 0.9204 | 0.5381 | 0.4529 | 0.0474 | 0.6427 | 0.1417 |
| 795 | P00739 | Haptoglobin-related protein | HPR | 5 | 28 | 0.3162 | 0.1476 | 0.2754 | 0.0000 | 1.2023 | 0.9379 | 0.2489 | 0.0000 | 0.9376 | 0.7369 | 0.8318 | 0.0480 | 0.6918 | 0.2779 | 0.3499 | 0.0000 | 0.7727 | 0.4638 | 0.6427 | 0.0027 | 0.7112 | 0.2932 | 0.8551 | 0.1586 | 0.7311 | 0.1898 | 0.0673 | 0.0000 |
| 796 | P15428 | 15-hydroxyprostaglandin dehydrogenase [NAD(+)] (15-PGDH) (EC 1.1.1.141) (Prostaglandin dehydrogenase 1) (Short chain dehydrogenase/reductase family 36C member 1) | HPGD PGDH1 SDR36C1 | 10 | 7 | 0.5754 | 0.0864 | 0.1306 | 0.2891 | 1.1169 | 0.2046 | 0.6792 | 0.6582 | 1.2134 | 0.6501 | 0.6310 | 0.5896 | 0.3767 | 0.0042 | 2.3768 | 0.1460 | 0.5702 | 0.0897 | 2.3335 | 0.0346 | 0.1905 | 0.0000 | 0.6026 | 0.6910 | 0.4246 | 0.0188 | 0.9550 | 0.8058 |
| 797 | P32754 | 4-hydroxyphenylpyruvate dioxygenase (EC 1.13.11.27) (4-hydroxyphenylpyruvic acid oxidase) (4HPPD) (HPD) (HPPDase) | HPD PPD | 36 | 56 | 0.4656 | 0.0004 | 0.8318 | 0.7432 | 0.5248 | 0.0013 | 0.9817 | 0.9423 | 0.4831 | 0.0020 | 0.5861 | 0.1175 | 1.6444 | 0.0001 | 1.8880 | 0.0030 | 0.5395 | 0.0005 | 2.1281 | 0.0000 | 0.1542 | 0.0000 | 0.7311 | 0.8795 | 0.2582 | 0.0000 | 0.7586 | 0.8054 |
| 798 | Q5SSJ5 | Heterochromatin protein 1-binding protein 3 (Protein HP1-BP74) | HP1BP3 | 5 | 9 | 1.7701 | 0.2471 | 1.8030 | 0.5283 | 2.1086 | 0.1506 | 3.9811 | 0.0287 | 2.0324 | 0.2626 | 1.7701 | 0.8194 | 3.5975 | 0.0169 | 1.8535 | 0.5593 | 0.6194 | 0.1939 | 2.8840 | 0.1544 | 0.9727 | 0.2670 | 3.5645 | 0.0439 | 3.5975 | 0.0352 | 2.6546 | 0.1586 |
| 799 | Q86XE5 | 4-hydroxy-2-oxoglutarate aldolase, mitochondrial (EC 4.1.3.16) (Dihydrodipicolinate synthase-like) (DHDPS-like protein) (Probable 2-keto-4-hydroxyglutarate aldolase) (Probable KHG-aldolase) (Protein 569272) | HOGA1 C10orf65 DHDPSL | 66 | 6 | 1.4723 | 0.0591 | 1.0965 | 0.4572 | 3.4995 | 0.0000 | 1.5417 | 0.0662 | 2.5119 | 0.0000 | 0.4365 | 0.1430 | 1.1482 | 0.4297 | 0.7244 | 0.9764 | 0.6194 | 0.1254 | 0.1754 | 0.0655 | 0.4613 | 0.0622 | 0.5808 | 0.4687 | 0.6252 | 0.1427 | 0.6982 | 0.4743 |
| 800 | Q00839 | Heterogeneous nuclear ribonucleoprotein U (hnRNP U) (Scaffold attachment factor A) (SAF-A) (p120) (pp120) | HNRNPU HNRPU SAFA U21.1 | 6 | 22 | 1.6144 | 0.6941 | 1.4191 | 0.8718 | 3.3113 | 0.0117 | 1.2942 | 0.8760 | 1.4191 | 0.2172 | 1.9055 | 0.1432 | 0.8630 | 0.8476 | 0.9550 | 0.3736 | 1.7539 | 0.3623 | 1.4997 | 0.6989 | 2.3550 | 0.1450 | 2.1281 | 0.1318 | 1.0280 | 0.9771 | 1.2474 | 0.6284 |
| 801 | O43390 | Heterogeneous nuclear ribonucleoprotein R (hnRNP R) | HNRNPR HNRPR | 4 | 7 | 0.8318 | 0.5982 | 0.7586 | 0.8641 | 0.4699 | 0.1472 | 0.9204 | 0.8259 | 0.9908 | 0.8027 | 0.8241 | 0.7143 | 0.7656 | 0.7105 | 0.8551 | 0.8435 | 0.8630 | 0.5995 | 0.7870 | 0.9667 | 0.0946 | 0.0205 | 0.8241 | 0.9257 | 0.5445 | 0.1745 | 1.1482 | 0.5153 |
| 802 | P52272 | Heterogeneous nuclear ribonucleoprotein M (hnRNP M) | HNRNPM HNRPM NAGR1 | 21 | 19 | 1.4060 | 0.8481 | 0.6918 | 0.2375 | 2.3988 | 0.0468 | 0.5105 | 0.0246 | 1.5560 | 0.3674 | 1.1169 | 0.6224 | 1.4997 | 0.3206 | 0.7244 | 0.0349 | 1.4859 | 0.3954 | 1.4588 | 0.6896 | 3.5975 | 0.0001 | 1.0000 | 0.8579 | 2.1281 | 0.1274 | 1.2474 | 0.7814 |
| 803 | P14866 | Heterogeneous nuclear ribonucleoprotein L (hnRNP L) | HNRNPL HNRPL P/OKcl.14 | 10 | 8 | 0.8551 | 0.8765 | 0.7586 | 0.7164 | 1.2474 | 0.3253 | 1.4322 | 0.3453 | 1.1803 | 0.5002 | 1.0375 | 0.2482 | 1.1376 | 0.3595 | 0.9727 | 0.6862 | 1.2474 | 0.2572 | 2.0893 | 0.1926 | 1.5417 | 0.2013 | 1.2589 | 0.5068 | 1.4322 | 0.2583 | 1.5276 | 0.0283 |
| 804 | P61978 | Heterogeneous nuclear ribonucleoprotein K (hnRNP K) (Transformation up-regulated nuclear protein) (TUNP) | HNRNPK HNRPK | 16 | 21 | 0.6026 | 0.1063 | 0.8790 | 0.9184 | 0.9550 | 0.6821 | 1.2134 | 0.7921 | 0.8395 | 0.9653 | 1.7378 | 0.0067 | 0.7379 | 0.0548 | 1.2134 | 0.6181 | 0.8318 | 0.6174 | 1.6444 | 0.0481 | 1.0864 | 0.4165 | 1.6904 | 0.0998 | 0.6546 | 0.3056 | 1.3677 | 0.1004 |
| 805 | P31942 | Heterogeneous nuclear ribonucleoprotein H3 (hnRNP H3) (Heterogeneous nuclear ribonucleoprotein 2H9) (hnRNP 2H9) | HNRNPH3 HNRPH3 | 10 | 6 | 0.5058 | 0.0262 | 1.6144 | 0.2778 | 0.8710 | 0.3366 | 0.8395 | 0.7812 | 0.7516 | 0.2171 | 1.2246 | 0.6217 | 0.2070 | 0.0022 | 1.0965 | 0.7193 | 0.9638 | 0.6972 | 1.3183 | 0.6442 | 0.7727 | 0.5327 | 1.3183 | 0.4286 | 0.7656 | 0.5209 | 0.7244 | 0.6419 |
| 806 | P55795 | Heterogeneous nuclear ribonucleoprotein H2 (hnRNP H2) (FTP-3) (Heterogeneous nuclear ribonucleoprotein H') (hnRNP H') [Cleaved into: Heterogeneous nuclear ribonucleoprotein H2, N-terminally processed] | HNRNPH2 FTP3 HNRPH2 | 30 | 9 | 1.1482 | 0.7288 | 1.1588 | 0.7037 | 2.0701 | 0.0744 | 0.8630 | 0.4353 | 1.6596 | 0.0403 | 1.2023 | 0.3472 | 1.4997 | 0.3905 | 1.2134 | 0.6068 | 2.0137 | 0.1054 | 1.0568 | 0.9975 | 3.4674 | 0.0001 | 0.9817 | 0.8319 | 1.7378 | 0.5059 | 1.0093 | 0.9014 |
| 807 | P31943 | Heterogeneous nuclear ribonucleoprotein H (hnRNP H) [Cleaved into: Heterogeneous nuclear ribonucleoprotein H, N-terminally processed] | HNRNPH1 HNRPH HNRPH1 | 6 | 14 | 1.0093 | 0.4950 | 1.0568 | 0.9484 | 0.8551 | 0.8981 | 0.8710 | 0.4628 | 0.8790 | 0.3734 | 0.9550 | 0.7820 | 0.5200 | 0.4367 | 1.1169 | 0.6604 | 1.2823 | 0.4336 | 1.1695 | 0.4682 | 1.1272 | 0.6664 | 0.9638 | 0.6956 | 0.5754 | 0.5469 | 1.1588 | 0.4141 |
| 808 | P52597 | Heterogeneous nuclear ribonucleoprotein F (hnRNP F) (Nucleolin-like protein mcs94-1) [Cleaved into: Heterogeneous nuclear ribonucleoprotein F, N-terminally processed] | HNRNPF HNRPF | 11 | 11 | 0.6368 | 0.6620 | 0.9727 | 0.9380 | 1.1912 | 0.8930 | 0.8241 | 0.7707 | 1.1066 | 0.8711 | 1.7061 | 0.3258 | 0.6194 | 0.6302 | 0.4365 | 0.2011 | 1.4997 | 0.7534 | 1.3804 | 0.3116 | 1.8707 | 0.6144 | 1.4723 | 0.5491 | 1.5996 | 0.7719 | 1.3552 | 0.4001 |
| 809 | Q14103 | Heterogeneous nuclear ribonucleoprotein D0 (hnRNP D0) (AU-rich element RNA-binding protein 1) | HNRNPD AUF1 HNRPD | 18 | 6 | 1.1695 | 0.4459 | 1.5136 | 0.2121 | 1.2706 | 0.7266 | 1.3552 | 0.7108 | 1.1376 | 0.3613 | 1.8535 | 0.0679 | 1.1482 | 0.4993 | 1.1588 | 0.7551 | 1.7061 | 0.4090 | 1.6596 | 0.1373 | 1.6904 | 0.3788 | 1.5704 | 0.0740 | 1.9770 | 0.1841 | 1.6596 | 0.1403 |
| 810 | P07910 | Heterogeneous nuclear ribonucleoproteins C1/C2 (hnRNP C1/C2) | HNRNPC HNRPC | 12 | 13 | 0.6194 | 0.1921 | 0.6081 | 0.2436 | 0.5754 | 0.2035 | 0.9120 | 0.7732 | 0.6194 | 0.3235 | 1.2706 | 0.1231 | 0.8241 | 0.4721 | 0.8710 | 0.7279 | 0.8551 | 0.5289 | 1.1272 | 0.2670 | 1.1588 | 0.2103 | 1.2589 | 0.3574 | 0.8017 | 0.4633 | 1.2823 | 0.1837 |
| 811 | Q99729 | Heterogeneous nuclear ribonucleoprotein A/B (hnRNP A/B) (APOBEC1-binding protein 1) (ABBP-1) | HNRNPAB ABBP1 HNRPAB | 9 | 6 | 0.8551 | 0.4226 | 0.3631 | 0.4164 | 1.0965 | 0.7632 | 0.3162 | 0.3933 | 1.0568 | 0.9017 | 0.8166 | 0.8680 | 0.9204 | 0.4385 | 0.6607 | 0.5562 | 1.0186 | 0.8516 | 0.8241 | 0.8006 | 1.0765 | 0.6199 | 0.8630 | 0.9972 | 0.8551 | 0.3665 | 0.8091 | 0.7888 |
| 812 | P51991 | Heterogeneous nuclear ribonucleoprotein A3 (hnRNP A3) | HNRNPA3 HNRPA3 | 10 | 12 | 0.4742 | 0.1157 | 1.9409 | 0.0367 | 0.9290 | 0.5205 | 1.6904 | 0.0912 | 0.5346 | 0.0752 | 1.4997 | 0.0605 | 0.8318 | 0.7316 | 1.4859 | 0.4410 | 0.9120 | 0.8774 | 1.8880 | 0.0383 | 1.6444 | 0.0278 | 1.6749 | 0.0592 | 0.9376 | 0.9621 | 1.0093 | 0.7124 |
| 813 | P22626 | Heterogeneous nuclear ribonucleoproteins A2/B1 (hnRNP A2/B1) | HNRNPA2B1 HNRPA2B1 | 6 | 32 | 1.9409 | 0.2792 | 1.3305 | 0.6325 | 2.0893 | 0.6287 | 2.1478 | 0.3254 | 2.3121 | 0.1938 | 1.9588 | 0.3173 | 1.5704 | 0.8964 | 1.2942 | 0.7435 | 0.9120 | 0.9787 | 2.1677 | 0.0515 | 4.0926 | 0.0603 | 2.2080 | 0.0886 | 0.9817 | 0.8136 | 1.5849 | 0.0409 |
| 814 | P09651 | Heterogeneous nuclear ribonucleoprotein A1 (hnRNP A1) (Helix-destabilizing protein) (Single-strand RNA-binding protein) (hnRNP core protein A1) [Cleaved into: Heterogeneous nuclear ribonucleoprotein A1, N-terminally processed] | HNRNPA1 HNRPA1 | 10 | 17 | 0.8872 | 0.7636 | 2.3121 | 0.1258 | 1.7865 | 0.3393 | 1.5704 | 0.1925 | 1.4322 | 0.4162 | 2.4660 | 0.1889 | 1.0864 | 0.7042 | 2.1878 | 0.1298 | 1.2823 | 0.8599 | 2.6303 | 0.1190 | 2.9107 | 0.0366 | 2.2909 | 0.1080 | 1.3932 | 0.7574 | 2.6062 | 0.1144 |
| 815 | Q13151 | Heterogeneous nuclear ribonucleoprotein A0 (hnRNP A0) | HNRNPA0 HNRPA0 | 31 | 3 | 2.0512 | 0.4285 | 4.5709 | 0.1610 | 2.7797 | 0.0369 | 5.5463 | 0.1676 | 2.1677 | 0.0884 | 7.1121 | 0.1352 | 1.8880 | 0.2756 | 4.2073 | 0.1819 | 2.3121 | 0.3135 | 5.0582 | 0.1678 | 2.5823 | 0.0049 | 5.5976 | 0.1698 | 2.0137 | 0.4323 | 5.8614 | 0.1294 |
| 816 | P50135 | Histamine N-methyltransferase (HMT) (EC 2.1.1.8) | HNMT | 21 | 10 | 0.9817 | 0.7819 | 0.7798 | 0.6246 | 1.4588 | 0.0953 | 1.0093 | 0.6912 | 1.4191 | 0.1287 | 1.4859 | 0.6271 | 1.0864 | 0.5796 | 0.9290 | 0.5321 | 1.2589 | 0.2047 | 0.6982 | 0.4835 | 1.9953 | 0.0094 | 0.7870 | 0.7113 | 1.1803 | 0.2931 | 0.3499 | 0.1879 |
| 817 | Q9H910 | Hematological and neurological expressed 1-like protein (HN1-like protein) | HN1L C16orf34 L11 | 3 | 3 | 0.8954 | 0.7412 | 0.6252 | 0.4322 | 1.1376 | 0.6723 | 0.4875 | 0.2134 | 1.1803 | 0.5959 | 0.3873 | 0.1799 | 1.0186 | 0.9324 | 0.6081 | 0.2659 | 0.7379 | 0.3927 | 0.2655 | 0.0573 | 1.1912 | 0.5708 | 0.3767 | 0.1044 | 0.8630 | 0.6629 | 0.9204 | 0.8356 |
| 818 | P30519 | Heme oxygenase 2 (HO-2) (EC 1.14.99.3) | HMOX2 HO2 | 11 | 3 | 0.6855 | 0.2746 | 0.9290 | 0.5303 | 0.5200 | 0.0246 | 1.0186 | 0.9296 | 0.5248 | 0.0443 | 1.0666 | 0.9701 | 1.1803 | 0.1735 | 1.0471 | 0.6897 | 1.0471 | 0.9917 | 0.8710 | 0.3403 | 0.3597 | 0.0087 | 0.8790 | 0.3394 | 0.2805 | 0.0031 | 0.9036 | 0.4882 |
| 819 | P09601 | Heme oxygenase 1 (HO-1) (EC 1.14.99.3) | HMOX1 HO HO1 | 3 | 5 | 0.7047 | 0.5457 | 1.9588 | 0.3720 | 0.9462 | 0.9298 | 1.1272 | 0.9683 | 1.1169 | 0.8124 | 1.2942 | 0.6177 | 1.0568 | 0.8950 | 1.1695 | 0.8821 | 0.3873 | 0.2520 | 1.3804 | 0.9793 | 1.1588 | 0.7620 | 0.8790 | 0.9887 | 1.4060 | 0.5385 | 1.0568 | 0.9657 |
| 820 | P05204 | Non-histone chromosomal protein HMG-17 (High mobility group nucleosome-binding domain-containing protein 2) | HMGN2 HMG17 | 4 | 11 | 1.0765 | 0.8165 | 1.9770 | 0.2664 | 0.9290 | 0.8822 | 1.3552 | 0.3960 | 1.3305 | 0.3176 | 2.9107 | 0.2407 | 0.8091 | 0.7089 | 1.2474 | 0.5030 | 0.9817 | 0.9026 | 2.7040 | 0.2226 | 0.8241 | 0.7156 | 3.2509 | 0.2261 | 1.0765 | 0.5464 | 4.3652 | 0.1826 |
| 821 | P54868 | Hydroxymethylglutaryl-CoA synthase, mitochondrial (HMG-CoA synthase) (EC 2.3.3.10) (3-hydroxy-3-methylglutaryl coenzyme A synthase) | HMGCS2 | 4 | 107 | 2.1281 | 0.2679 | 1.0666 | 0.0464 | 1.3305 | 0.3406 | 1.5704 | 0.0336 | 2.3768 | 0.0552 | 0.6982 | 0.0029 | 3.3729 | 0.0980 | 0.7870 | 0.0027 | 0.5808 | 0.5764 | 0.8166 | 0.0133 | 1.8707 | 0.1840 | 0.6918 | 0.0330 | 2.1086 | 0.2964 | 0.0535 | 0.0000 |
| 822 | Q01581 | Hydroxymethylglutaryl-CoA synthase, cytoplasmic (HMG-CoA synthase) (EC 2.3.3.10) (3-hydroxy-3-methylglutaryl coenzyme A synthase) | HMGCS1 HMGCS | 14 | 18 | 0.2377 | 0.0151 | 1.5849 | 0.6706 | 0.7870 | 0.7654 | 1.4859 | 0.8768 | 0.8710 | 0.9793 | 2.3550 | 0.4225 | 0.5916 | 0.3218 | 5.0582 | 0.0011 | 1.3183 | 0.2665 | 5.2000 | 0.0000 | 1.6749 | 0.4047 | 1.8365 | 0.2553 | 0.6081 | 0.1190 | 1.9231 | 0.7417 |
| 823 | P35914 | Hydroxymethylglutaryl-CoA lyase, mitochondrial (HL) (HMG-CoA lyase) (EC 4.1.3.4) (3-hydroxy-3-methylglutarate-CoA lyase) | HMGCL | 116 | 21 | 1.3552 | 0.3770 | 2.3550 | 0.6073 | 0.8472 | 0.0002 | 1.8535 | 0.7594 | 0.7870 | 0.0031 | 1.0000 | 0.2050 | 1.1272 | 0.4260 | 3.2509 | 0.1930 | 1.4191 | 0.8361 | 1.4859 | 0.6635 | 0.0470 | 0.0000 | 1.6293 | 0.7338 | 0.0643 | 0.0000 | 0.5152 | 0.3142 |
| 824 | P04222 | HLA class I histocompatibility antigen, Cw-3 alpha chain (MHC class I antigen Cw*3) | HLA-C HLAC | 18 | 11 | 2.7040 | 0.0042 | 1.1482 | 0.8202 | 1.1169 | 0.9777 | 0.8551 | 0.5571 | 7.4473 | 0.0000 | 2.0701 | 0.3625 | 2.3988 | 0.0649 | 1.1695 | 0.8017 | 1.3552 | 0.1675 | 0.5546 | 0.3850 | 2.5351 | 0.0147 | 0.6982 | 0.3999 | 4.0926 | 0.0014 | 0.3981 | 0.2508 |
| 825 | P01889 | HLA class I histocompatibility antigen, B-7 alpha chain (MHC class I antigen B*7) | HLA-B HLAB | 26 | 13 | 1.7539 | 0.6336 | 1.5996 | 0.6900 | 0.8091 | 0.0020 | 0.4246 | 0.9216 | 0.8790 | 0.0058 | 5.8076 | 0.1751 | 1.8365 | 0.5729 | 2.0893 | 0.3994 | 1.4322 | 0.6038 | 2.9923 | 0.2834 | 0.1318 | 0.0000 | 2.0701 | 0.3598 | 0.1722 | 0.0003 | 0.7870 | 0.8234 |
| 826 | P30461 | HLA class I histocompatibility antigen, B-13 alpha chain (MHC class I antigen B*13) | HLA-B HLAB | 14 | 10 | 2.5586 | 0.3217 | 0.2421 | 0.1801 | 3.0479 | 0.3536 | 0.7870 | 0.6665 | 5.8076 | 0.1918 | 6.4269 | 0.1312 | 0.4169 | 0.4427 | 0.2489 | 0.1746 | 1.9409 | 0.4687 | 1.1588 | 0.7542 | 4.4875 | 0.2218 | 1.5704 | 0.4500 | 3.5645 | 0.2480 | 1.5136 | 0.4689 |
| 827 | P01891 | HLA class I histocompatibility antigen, A-68 alpha chain (Aw-68) (HLA class I histocompatibility antigen, A-28 alpha chain) (MHC class I antigen A*68) | HLA-A HLAA | 10 | 11 | 1.6144 | 0.4321 | 0.6792 | 0.5095 | 3.8726 | 0.1777 | 0.2884 | 0.1951 | 0.7656 | 0.6249 | 1.2942 | 0.6146 | 0.9376 | 0.9037 | 0.7112 | 0.5527 | 1.2823 | 0.6340 | 0.6310 | 0.4536 | 6.6069 | 0.1295 | 0.6310 | 0.4576 | 0.8630 | 0.7816 | 1.0666 | 0.8770 |
| 828 | Q2TB90 | Putative hexokinase HKDC1 (EC 2.7.1.1) (Hexokinase domain-containing protein 1) | HKDC1 | 14 | 4 | 0.4571 | 0.5413 | 0.3981 | 0.4353 | 0.2992 | 0.3394 | 0.2489 | 0.0996 | 0.3162 | 0.4444 | 0.2489 | 0.2157 | 0.0331 | 0.1294 | 0.4018 | 0.2994 | 0.8472 | 0.6263 | 1.8197 | 0.2776 | 2.3550 | 0.3032 | 1.6144 | 0.3739 | 4.3652 | 0.1447 | 5.4450 | 0.0026 |
| 829 | P19367 | Hexokinase-1 (EC 2.7.1.1) (Brain form hexokinase) (Hexokinase type I) (HK I) | HK1 | 10 | 4 | 0.7112 | 0.8022 | 1.2359 | 0.5920 | 0.4529 | 0.3811 | 1.2706 | 0.5671 | 0.2188 | 0.1888 | 1.1588 | 0.6902 | 1.6904 | 0.4550 | 1.1482 | 0.7039 | 0.5598 | 0.6953 | 1.5560 | 0.3839 | 0.8091 | 0.7591 | 1.3552 | 0.4813 | 0.3532 | 0.2645 | 1.2823 | 0.5488 |
| 830 | Q16777 | Histone H2A type 2-C (Histone H2A-GL101) (Histone H2A/q) | HIST2H2AC H2AFQ | 6 | 57 | 0.5395 | 0.3730 | 1.5276 | 0.7249 | 1.0471 | 0.8125 | 1.1695 | 0.7913 | 0.1343 | 0.1171 | 2.0137 | 0.5288 | 1.9770 | 0.3475 | 0.8710 | 0.9933 | 0.2884 | 0.3172 | 1.9231 | 0.5058 | 3.3113 | 0.1323 | 1.8535 | 0.5093 | 1.4588 | 0.5072 | 3.0761 | 0.3378 |
| 831 | P62805 | Histone H4 | HIST1H4A H4/A H4FA; HIST1H4B H4/I H4FI; HIST1H4C H4/G H4FG; HIST1H4D H4/B H4FB; HIST1H4E H4/J H4FJ; HIST1H4F H4/C H4FC; HIST1H4H H4/H H4FH; HIST1H4I H4/M H4FM; HIST1H4J H4/E H4FE; HIST1H4K H4/D H4FD; HIST1H4L H4/K H4FK; HIST2H4A H4/N H4F2 H4FN HIST2H4; HIST2H4B H4/O H4FO; HIST4H4 | 5 | 37 | 0.7311 | 0.1165 | 10.5682 | 0.0011 | 0.7727 | 0.5639 | 7.5162 | 0.0067 | 0.6668 | 0.3558 | 5.0119 | 0.0018 | 0.9036 | 0.6258 | 1.7539 | 0.6589 | 1.0280 | 0.8403 | 9.2897 | 0.0002 | 2.1677 | 0.0543 | 5.7016 | 0.0780 | 2.3121 | 0.0112 | 8.4723 | 0.0905 |
| 832 | P68431 | Histone H3.1 (Histone H3/a) (Histone H3/b) (Histone H3/c) (Histone H3/d) (Histone H3/f) (Histone H3/h) (Histone H3/i) (Histone H3/j) (Histone H3/k) (Histone H3/l) | HIST1H3A H3FA; HIST1H3B H3FL; HIST1H3C H3FC; HIST1H3D H3FB; HIST1H3E H3FD; HIST1H3F H3FI; HIST1H3G H3FH; HIST1H3H H3FK; HIST1H3I H3FF; HIST1H3J H3FJ | 64 | 17 | 0.4699 | 0.4711 |  |  | 1.7061 | 0.8069 |  |  | 1.3804 | 0.9985 |  |  | 1.2134 | 0.9969 |  |  | 2.0893 | 0.6528 |  |  | 2.5586 | 0.4688 |  |  | 1.8365 | 0.8089 |  |  |
| 833 | P10412 | Histone H1.4 (Histone H1b) (Histone H1s-4) | HIST1H1E H1F4 | 46 | 22 | 3.4356 | 0.9643 | 4.5290 | 0.1076 | 9.2045 | 0.0135 | 4.2073 | 0.1757 | 1.5417 | 0.6312 | 2.4889 | 0.3807 | 5.4954 | 0.1247 | 1.6293 | 0.9483 | 8.6298 | 0.0285 | 4.4875 | 0.0798 | 7.7268 | 0.0265 | 2.6303 | 0.2079 | 4.4463 | 0.2150 | 4.4463 | 0.0325 |
| 834 | P16401 | Histone H1.5 (Histone H1a) (Histone H1b) (Histone H1s-3) | HIST1H1B H1F5 | 21 | 6 |  |  | 0.3873 | 0.7801 |  |  | 0.5754 | 0.4322 |  |  | 0.8017 | 0.9747 |  |  | 0.4246 | 0.5615 |  |  | 1.0568 | 0.7349 |  |  | 0.4831 | 0.6660 |  |  | 1.8365 | 0.3763 |
| 835 | Q9BX68 | Histidine triad nucleotide-binding protein 2, mitochondrial (HINT-2) (EC 3.-.-.-) (HINT-3) (HIT-17kDa) (PKCI-1-related HIT protein) | HINT2 | 28 | 16 |  |  | 1.3428 | 0.6154 |  |  | 0.8872 | 0.6687 |  |  | 0.8318 | 0.2529 |  |  | 0.6026 | 0.6765 |  |  | 0.9036 | 0.5743 |  |  | 1.7539 | 0.4706 |  |  | 0.5395 | 0.1395 |
| 836 | P49773 | Histidine triad nucleotide-binding protein 1 (EC 3.-.-.-) (Adenosine 5'-monophosphoramidase) (Protein kinase C inhibitor 1) (Protein kinase C-interacting protein 1) (PKCI-1) | HINT1 HINT PKCI1 PRKCNH1 | 14 | 8 | 0.2965 | 0.7148 | 0.6194 | 0.4119 | 1.3428 | 0.6973 | 0.7244 | 0.3690 | 0.9120 | 0.3852 | 0.9727 | 0.8261 | 0.3837 | 0.8068 | 0.8017 | 0.4689 | 1.1066 | 0.4312 | 0.9376 | 0.6966 | 5.4450 | 0.0445 | 1.1482 | 0.7057 | 3.4356 | 0.0914 | 0.2443 | 0.0449 |
| 837 | Q6NVY1 | 3-hydroxyisobutyryl-CoA hydrolase, mitochondrial (EC 3.1.2.4) (3-hydroxyisobutyryl-coenzyme A hydrolase) (HIB-CoA hydrolase) (HIBYL-CoA-H) | HIBCH | 14 | 15 | 1.1482 | 0.8769 | 0.4920 | 0.0679 | 1.1272 | 0.8395 | 0.8954 | 0.3954 | 0.9908 | 0.9381 | 0.6368 | 0.0424 | 0.9376 | 0.6365 | 0.8551 | 0.3763 | 1.2706 | 0.4309 | 1.9953 | 0.0048 | 0.2729 | 0.0259 | 1.4322 | 0.0613 | 0.7047 | 0.1457 | 0.4055 | 0.0134 |
| 838 | P31937 | 3-hydroxyisobutyrate dehydrogenase, mitochondrial (HIBADH) (EC 1.1.1.31) | HIBADH | 9 | 21 | 0.7516 | 0.3056 | 0.9550 | 0.5758 | 0.8091 | 0.5547 | 1.2942 | 0.0842 | 0.9120 | 0.7781 | 0.4656 | 0.0912 | 1.1272 | 0.9493 | 1.1588 | 0.5430 | 0.8318 | 0.4021 | 0.7798 | 0.4478 | 0.3767 | 0.0810 | 0.9036 | 0.7141 | 0.2729 | 0.0495 | 0.4571 | 0.2083 |
| 839 | Q93099 | Homogentisate 1,2-dioxygenase (EC 1.13.11.5) (Homogentisate oxygenase) (Homogentisic acid oxidase) (Homogentisicase) | HGD HGO | 15 | 31 | 1.4454 | 0.1957 | 1.5136 | 0.9575 | 1.1482 | 0.9146 | 2.6792 | 0.0008 | 0.6194 | 0.1382 | 1.1912 | 0.6025 | 0.7586 | 0.1149 | 1.1695 | 0.9528 | 0.8710 | 0.8842 | 1.3552 | 0.9666 | 0.1191 | 0.0009 | 0.8472 | 0.7109 | 0.1977 | 0.0038 | 0.5861 | 0.1151 |
| 840 | P07686 | Beta-hexosaminidase subunit beta (EC 3.2.1.52) (Beta-N-acetylhexosaminidase subunit beta) (Hexosaminidase subunit B) (Cervical cancer proto-oncogene 7 protein) (HCC-7) (N-acetyl-beta-glucosaminidase subunit beta) [Cleaved into: Beta-hexosaminidase subunit beta chain B; Beta-hexosaminidase subunit beta chain A] | HEXB HCC7 | 20 | 10 | 0.7586 | 0.2006 | 0.9204 | 0.6467 | 0.5297 | 0.0085 | 0.9204 | 0.9141 | 0.5808 | 0.0014 | 1.9953 | 0.2316 | 0.9290 | 0.4491 | 0.7586 | 0.9642 | 0.9036 | 0.5976 | 1.5417 | 0.3786 | 0.1820 | 0.0000 | 1.3428 | 0.5233 | 0.3499 | 0.0001 | 2.1677 | 0.1194 |
| 841 | P06865 | Beta-hexosaminidase subunit alpha (EC 3.2.1.52) (Beta-N-acetylhexosaminidase subunit alpha) (Hexosaminidase subunit A) (N-acetyl-beta-glucosaminidase subunit alpha) | HEXA | 40 | 3 | 2.3335 | 0.0013 | 0.8472 | 0.4583 | 1.5136 | 0.0535 | 0.7311 | 0.3016 | 2.2491 | 0.0001 | 0.7870 | 0.4376 | 2.3988 | 0.0002 | 0.7656 | 0.2658 | 1.6749 | 0.0156 | 0.7727 | 0.4014 | 0.2377 | 0.0001 | 0.8472 | 0.4838 | 0.3133 | 0.0116 | 0.9376 | 0.7810 |
| 842 | Q9NRV9 | Heme-binding protein 1 (p22HBP) | HEBP1 HBP | 4 | 10 | 0.8551 | 0.7838 | 0.6668 | 0.9006 | 1.2359 | 0.4152 | 0.9120 | 0.4716 | 0.9817 | 0.7393 | 0.7586 | 0.7186 | 0.8241 | 0.9111 | 1.1272 | 0.6722 | 0.6607 | 0.2619 | 1.6749 | 0.0715 | 1.9409 | 0.0160 | 1.1912 | 0.4835 | 1.2359 | 0.5741 | 1.0864 | 0.9668 |
| 843 | Q00341 | Vigilin (High density lipoprotein-binding protein) (HDL-binding protein) | HDLBP HBP VGL | 5 | 33 | 0.5970 | 0.4450 | 1.2246 | 0.4541 | 0.8472 | 0.3980 | 1.0765 | 0.5466 | 0.7656 | 0.3587 | 1.4191 | 0.2723 | 0.5248 | 0.2853 | 0.7727 | 0.7330 | 0.7379 | 0.4083 | 1.5849 | 0.0873 | 1.7061 | 0.1036 | 1.3183 | 0.1529 | 0.8395 | 0.6650 | 1.2589 | 0.1045 |
| 844 | Q9BSH5 | Haloacid dehalogenase-like hydrolase domain-containing protein 3 | HDHD3 C9orf158 | 12 | 8 | 2.0137 | 0.1592 | 1.1376 | 0.2349 | 2.4434 | 0.0498 | 1.1376 | 0.3812 | 2.7797 | 0.0451 | 1.0375 | 0.5952 | 1.7865 | 0.3337 | 0.9908 | 0.9989 | 2.7797 | 0.0410 | 1.0965 | 0.4741 | 1.9953 | 0.2638 | 0.9727 | 0.8311 | 1.8880 | 0.2041 | 0.9376 | 0.7975 |
| 845 | Q9H0R4 | Haloacid dehalogenase-like hydrolase domain-containing protein 2 | HDHD2 | 41 | 5 | 0.8710 | 0.6789 | 1.0666 | 0.7896 | 1.0765 | 0.5056 | 1.6444 | 0.1656 | 1.0280 | 0.8960 | 1.8707 | 0.1421 | 1.3552 | 0.2379 | 1.3062 | 0.3963 | 1.4454 | 0.3635 | 1.2942 | 0.5563 | 1.0965 | 0.4635 | 0.9036 | 0.8640 | 1.2823 | 0.3689 | 0.6310 | 0.9205 |
| 846 | P51858 | Hepatoma-derived growth factor (HDGF) (High mobility group protein 1-like 2) (HMG-1L2) | HDGF HMG1L2 | 8 | 11 | 1.3428 | 0.2580 | 0.6792 | 0.4985 | 1.0568 | 0.8269 | 0.6792 | 0.2037 | 0.6668 | 0.4746 | 0.9120 | 0.6259 | 0.7516 | 0.7895 | 0.7311 | 0.4007 | 1.1588 | 0.7827 | 0.8395 | 0.3379 | 0.4920 | 0.1262 | 1.1272 | 0.5239 | 0.3192 | 0.1512 | 0.6368 | 0.6500 |
| 847 | Q9UBN7 | Histone deacetylase 6 (HD6) (EC 3.5.1.98) | HDAC6 KIAA0901 JM21 | 3 | 8 | 0.6792 | 0.4254 | 6.7298 | 0.0245 | 1.1272 | 0.7985 | 7.1121 | 0.0040 | 1.1803 | 0.8421 | 3.1915 | 0.0864 | 1.0093 | 0.7376 | 7.9433 | 0.0047 | 0.6982 | 0.5411 | 1.7219 | 0.3996 | 0.7447 | 0.2074 | 5.0119 | 0.0582 | 0.5754 | 0.1688 | 2.6546 | 0.2513 |
| 848 | P69892 | Hemoglobin subunit gamma-2 (Gamma-2-globin) (Hb F Ggamma) (Hemoglobin gamma-2 chain) (Hemoglobin gamma-G chain) | HBG2 | 6 | 24 | 0.5200 | 0.0385 | 1.1272 | 0.4204 | 0.4875 | 0.0493 | 1.2706 | 0.4711 | 0.5916 | 0.1882 | 0.3873 | 0.1996 | 0.2780 | 0.0174 | 1.1912 | 0.4077 | 0.5649 | 0.4019 | 2.2699 | 0.0901 | 0.4246 | 0.0220 | 0.1706 | 0.0158 | 0.3802 | 0.0352 | 0.4246 | 0.6145 |
| 849 | P02042 | Hemoglobin subunit delta (Delta-globin) (Hemoglobin delta chain) | HBD | 8 | 107 | 1.0965 | 0.4737 | 0.7112 | 0.2299 | 0.9638 | 0.8420 | 0.2489 | 0.0051 | 1.0280 | 0.4239 | 0.9204 | 0.4898 | 0.9204 | 0.7040 | 1.1169 | 0.5630 | 0.9727 | 0.9269 | 0.5248 | 0.0600 | 1.1066 | 0.2270 | 0.3020 | 0.0066 | 1.1588 | 0.4176 | 0.1368 | 0.0017 |
| 850 | P68871 | Hemoglobin subunit beta (Beta-globin) (Hemoglobin beta chain) [Cleaved into: LVV-hemorphin-7; Spinorphin] | HBB | 25 | 177 | 0.2312 | 0.2126 | 0.6252 | 0.0283 | 0.6918 | 0.4884 | 0.0413 | 0.0000 | 1.3552 | 0.7163 | 0.3436 | 0.0442 | 0.0149 | 0.0975 | 0.7943 | 0.2313 | 0.4920 | 0.3434 | 0.3251 | 0.0004 | 0.1770 | 0.1960 | 0.0608 | 0.0136 | 0.2168 | 0.1857 | 0.0268 | 0.0070 |
| 851 | P69905 | Hemoglobin subunit alpha (Alpha-globin) (Hemoglobin alpha chain) | HBA1; HBA2 | 137 | 111 | 0.7379 | 0.0233 | 0.5395 | 0.0000 | 0.6855 | 0.0335 | 0.0752 | 0.0015 | 1.9588 | 0.0554 | 0.4207 | 0.0000 | 0.1076 | 0.0001 | 0.7112 | 0.0001 | 0.1614 | 0.0005 | 0.3802 | 0.0000 | 0.2128 | 0.0004 | 0.1380 | 0.0001 | 0.4920 | 0.0049 | 0.0724 | 0.0000 |
| 852 | P12081 | Histidine--tRNA ligase, cytoplasmic (EC 6.1.1.21) (Histidyl-tRNA synthetase) (HisRS) | HARS HRS | 216 | 9 | 0.5495 | 0.0856 | 1.3428 | 0.2918 | 0.4875 | 0.0033 | 1.9055 | 0.2886 | 1.1912 | 0.0951 | 2.0324 | 0.2273 | 0.0540 | 0.0003 | 1.0666 | 0.5687 | 0.0964 | 0.0002 | 1.2134 | 0.8729 | 0.0679 | 0.0191 | 0.8241 | 0.9549 | 0.2831 | 0.0351 | 1.2134 | 0.5013 |
| 853 | Q9NYQ3 | Hydroxyacid oxidase 2 (HAOX2) (EC 1.1.3.15) ((S)-2-hydroxy-acid oxidase, peroxisomal) (Cell growth-inhibiting gene 16 protein) (Long chain alpha-hydroxy acid oxidase) (Long-chain L-2-hydroxy acid oxidase) | HAO2 HAOX2 GIG16 | 145 | 3 | 0.5105 | 0.0090 | 1.3552 | 0.3555 | 0.4406 | 0.0042 | 1.2589 | 0.4558 | 1.1272 | 0.4870 | 1.4454 | 0.2919 | 0.0586 | 0.0000 | 1.1912 | 0.5691 | 0.1294 | 0.0000 | 1.2823 | 0.4346 | 0.1038 | 0.0000 | 1.2942 | 0.4233 | 0.3105 | 0.0021 | 1.0765 | 0.7888 |
| 854 | Q9UJM8 | Hydroxyacid oxidase 1 (HAOX1) (EC 1.1.3.15) (Glycolate oxidase) (GOX) | HAO1 GOX1 HAOX1 | 7 | 52 | 0.9638 | 0.9319 | 1.8365 | 0.3955 | 0.9817 | 0.9316 | 1.9055 | 0.0277 | 0.9727 | 0.9816 | 2.9107 | 0.0065 | 1.0093 | 0.8571 | 4.8753 | 0.0000 | 1.0186 | 0.6834 | 1.2246 | 0.9946 | 1.0765 | 0.2893 | 3.1915 | 0.0032 | 1.1169 | 0.2223 | 1.9409 | 0.2215 |
| 855 | P42357 | Histidine ammonia-lyase (Histidase) (EC 4.3.1.3) | HAL HIS | 4 | 13 | 2.0512 | 0.2837 | 0.2512 | 0.0001 | 1.0186 | 0.9960 | 0.9036 | 0.6807 | 0.8630 | 0.7893 | 0.2051 | 0.0002 | 0.8166 | 0.4903 | 0.2228 | 0.0004 | 2.2491 | 0.1046 | 0.2312 | 0.0000 | 0.1629 | 0.0239 | 0.3221 | 0.0002 | 0.1259 | 0.2055 | 0.3767 | 0.0007 |
| 856 | Q16775 | Hydroxyacylglutathione hydrolase, mitochondrial (EC 3.1.2.6) (Glyoxalase II) (Glx II) | HAGH GLO2 HAGH1 | 46 | 12 | 2.2909 | 0.1366 | 0.9462 | 0.3744 | 1.8880 | 0.0577 | 0.6252 | 0.0746 | 2.5586 | 0.0001 | 0.7047 | 0.0377 | 2.0324 | 0.0196 | 0.5012 | 0.0491 | 2.6062 | 0.0027 | 1.0568 | 0.4463 | 0.2535 | 0.0002 | 1.2706 | 0.9501 | 0.9290 | 0.3011 | 0.3499 | 0.0201 |
| 857 | P55084 | Trifunctional enzyme subunit beta, mitochondrial (TP-beta) [Includes: 3-ketoacyl-CoA thiolase (EC 2.3.1.16) (Acetyl-CoA acyltransferase) (Beta-ketothiolase)] | HADHB MSTP029 | 13 | 41 | 0.9290 | 0.8576 | 1.2706 | 0.7428 | 0.6918 | 0.0898 | 1.3183 | 0.0783 | 0.2754 | 0.0028 | 1.3677 | 0.3465 | 1.6904 | 0.0656 | 1.2023 | 0.6254 | 0.4742 | 0.0327 | 1.5849 | 0.1123 | 0.1738 | 0.0093 | 1.5136 | 0.4204 | 0.1820 | 0.0018 | 0.5702 | 0.0369 |
| 858 | P40939 | Trifunctional enzyme subunit alpha, mitochondrial (78 kDa gastrin-binding protein) (TP-alpha) [Includes: Long-chain enoyl-CoA hydratase (EC 4.2.1.17); Long chain 3-hydroxyacyl-CoA dehydrogenase (EC 1.1.1.211)] | HADHA HADH | 9 | 79 | 0.6081 | 0.0894 | 1.1066 | 0.9319 | 0.9908 | 0.3020 | 1.6904 | 0.9178 | 0.8551 | 0.1238 | 1.8707 | 0.3262 | 0.6918 | 0.1393 | 1.5276 | 0.9757 | 0.6918 | 0.1229 | 2.1086 | 0.0433 | 0.1923 | 0.0008 | 2.2284 | 0.0468 | 0.1330 | 0.0011 | 0.7244 | 0.0143 |
| 859 | Q16836 | Hydroxyacyl-coenzyme A dehydrogenase, mitochondrial (HCDH) (EC 1.1.1.35) (Medium and short-chain L-3-hydroxyacyl-coenzyme A dehydrogenase) (Short-chain 3-hydroxyacyl-CoA dehydrogenase) | HADH HAD HADHSC SCHAD | 38 | 35 | 0.9908 | 0.4836 | 0.7656 | 0.8044 | 1.0965 | 0.4294 | 0.9817 | 0.7878 | 0.7112 | 0.4618 | 0.8395 | 0.6227 | 1.4322 | 0.2103 | 1.4322 | 0.2764 | 1.5849 | 0.0883 | 1.3183 | 0.3370 | 0.5105 | 0.0009 | 1.4859 | 0.1833 | 0.3837 | 0.0004 | 0.3404 | 0.0329 |
| 860 | Q9UJ83 | 2-hydroxyacyl-CoA lyase 1 (EC 4.1.-.-) (2-hydroxyphytanoyl-CoA lyase) (2-HPCL) (Phytanoyl-CoA 2-hydroxylase 2) | HACL1 HPCL HPCL2 PHYH2 HSPC279 | 90 | 3 | 1.1376 | 0.1468 | 0.4786 | 0.2279 | 1.2134 | 0.3381 | 1.7378 | 0.3346 | 0.9550 | 0.5046 | 1.5704 | 0.4475 | 2.1281 | 0.0034 | 0.8790 | 0.8479 | 1.9409 | 0.1919 | 0.7870 | 0.6214 | 0.3767 | 0.0000 | 0.9817 | 0.8097 | 0.2168 | 0.0000 | 0.3698 | 0.1495 |
| 861 | P46952 | 3-hydroxyanthranilate 3,4-dioxygenase (EC 1.13.11.6) (3-hydroxyanthranilate oxygenase) (3-HAO) (3-hydroxyanthranilic acid dioxygenase) (HAD) | HAAO | 36 | 25 | 0.9550 | 0.9251 | 1.4322 | 0.7020 | 1.0471 | 0.6656 | 1.6596 | 0.3481 | 1.2134 | 0.9412 | 1.0965 | 0.8986 | 1.2134 | 0.8359 | 1.1169 | 0.3304 | 1.2823 | 0.7581 | 0.6607 | 0.0343 | 0.2188 | 0.0005 | 1.0864 | 0.9621 | 0.2312 | 0.0143 | 0.2443 | 0.0025 |
| 862 | O95479 | GDH/6PGL endoplasmic bifunctional protein [Includes: Glucose 1-dehydrogenase (EC 1.1.1.47) (Hexose-6-phosphate dehydrogenase); 6-phosphogluconolactonase (6PGL) (EC 3.1.1.31)] | H6PD GDH | 3 | 22 | 0.6138 | 0.8200 | 0.5861 | 0.0909 | 0.3565 | 0.1984 | 0.9550 | 0.4196 | 1.1803 | 0.5445 | 0.7311 | 0.0166 | 0.5495 | 0.2644 | 0.5058 | 0.0046 | 0.9036 | 0.6200 | 0.5445 | 0.0130 | 0.6730 | 0.2229 | 0.6486 | 0.0946 | 0.5916 | 0.2763 | 0.4656 | 0.0007 |
| 863 | Q6NXT2 | Histone H3.3C (Histone H3.5) | H3F3C | 26 | 8 | 0.7656 | 0.5377 | 16.2930 | 0.1242 | 0.8318 | 0.2748 | 3.1623 | 0.4760 | 1.0765 | 0.5904 | 10.0000 | 0.1666 | 1.0186 | 0.3049 | 5.0582 | 0.2580 | 0.8630 | 0.0929 | 6.6681 | 0.2260 | 0.0904 | 0.0000 | 8.7902 | 0.1894 | 0.2911 | 0.0013 | 0.8710 | 0.7187 |
| 864 | P0C0S5 | Histone H2A.Z (H2A/z) | H2AFZ H2AZ | 24 | 14 | 1.1066 | 0.8511 | 1.1272 | 0.8202 | 0.6368 | 0.0156 | 1.0375 | 0.8539 | 0.7178 | 0.0127 | 1.1803 | 0.6459 | 1.3183 | 0.2878 | 0.5808 | 0.2938 | 0.9036 | 0.5834 | 0.9376 | 0.8100 | 0.4831 | 0.0001 | 0.9204 | 0.9379 | 0.5395 | 0.0019 | 0.9290 | 0.7602 |
| 865 | O75367 | Core histone macro-H2A.1 (Histone macroH2A1) (mH2A1) (Histone H2A.y) (H2A/y) (Medulloblastoma antigen MU-MB-50.205) | H2AFY MACROH2A1 | 8 | 47 | 26.3027 | 0.1324 | 2.3988 | 0.1905 | 35.6451 | 0.1120 | 2.3550 | 0.2594 | 32.2107 | 0.1233 | 2.2909 | 0.0611 | 23.7684 | 0.1586 | 1.8365 | 0.4308 | 25.1189 | 0.1598 | 3.2211 | 0.0957 | 22.9087 | 0.2073 | 3.4041 | 0.0240 | 16.2930 | 0.5723 | 3.8726 | 0.0388 |
| 866 | P16104 | Histone H2AX (H2a/x) (Histone H2A.X) | H2AFX H2AX | 11 | 58 | 1.1376 | 0.8320 | 14.7231 | 0.1333 | 1.9055 | 0.2899 | 9.7275 | 0.1668 | 1.4454 | 0.7523 | 5.9156 | 0.2794 | 1.0000 | 0.8471 | 3.1333 | 0.2879 | 2.1086 | 0.2947 | 11.6950 | 0.1453 | 1.9055 | 0.2237 | 13.1826 | 0.1361 | 2.3768 | 0.2420 | 1.4723 | 0.9569 |
| 867 | Q92522 | Histone H1x | H1FX | 43 | 4 | 0.7943 | 0.2880 | 2.4889 | 0.3750 | 1.3677 | 0.3092 | 2.3335 | 0.6216 | 1.0375 | 0.8857 | 2.0701 | 0.6723 | 1.0666 | 0.9421 | 2.5119 | 0.4459 | 1.3804 | 0.5857 | 3.6308 | 0.3280 | 1.6749 | 0.2820 | 3.1333 | 0.3842 | 1.4191 | 0.7485 | 3.2810 | 0.3519 |
| 868 | P07305 | Histone H1.0 (Histone H1') (Histone H1(0)) [Cleaved into: Histone H1.0, N-terminally processed] | H1F0 H1FV | 64 | 7 | 3.8019 | 0.1601 | 2.0701 | 0.0852 | 3.0761 | 0.1840 | 1.1376 | 0.9582 | 2.1677 | 0.2646 | 2.2284 | 0.1998 | 2.1086 | 0.1874 | 1.2474 | 0.8069 | 1.8197 | 0.2177 | 2.2699 | 0.1906 | 2.0137 | 0.2923 | 2.7542 | 0.0363 | 2.1878 | 0.2513 | 0.6026 | 0.4006 |
| 869 | P54840 | Glycogen [starch] synthase, liver (EC 2.4.1.11) | GYS2 | 6 | 10 | 1.9588 | 0.1826 | 0.6194 | 0.0630 | 2.4210 | 0.1382 | 0.2655 | 0.0037 | 1.4191 | 0.4370 | 0.5346 | 0.0151 | 1.5136 | 0.3430 | 0.4055 | 0.0554 | 1.2359 | 0.3777 | 0.5495 | 0.0364 | 2.0701 | 0.1618 | 1.0375 | 0.6746 | 1.7378 | 0.1848 | 0.2399 | 0.0077 |
| 870 | O43708 | Maleylacetoacetate isomerase (MAAI) (EC 5.2.1.2) (GSTZ1-1) (Glutathione S-transferase zeta 1) (EC 2.5.1.18) | GSTZ1 MAAI | 10 | 10 | 1.0093 | 0.9550 | 1.6749 | 0.0451 | 1.2359 | 0.4377 | 0.6194 | 0.3071 | 1.1066 | 0.9180 | 0.9908 | 0.5023 | 0.6730 | 0.8260 | 0.7727 | 0.5808 | 1.6596 | 0.2561 | 1.5560 | 0.0880 | 0.2992 | 0.1333 | 1.9409 | 0.0057 | 0.4831 | 0.2654 | 0.1159 | 0.0567 |
| 871 | P0CG30 | Glutathione S-transferase theta-2B (EC 2.5.1.18) (GST class-theta-2) (Glutathione S-transferase theta-2) | GSTT2B GSTT2 | 11 | 5 | 0.8166 | 0.5562 | 2.3121 | 0.5776 | 0.9290 | 0.3140 | 1.4191 | 0.9534 | 0.5445 | 0.0447 | 1.1376 | 0.8841 | 0.6730 | 0.0351 | 0.6310 | 0.3896 | 0.5346 | 0.0137 | 0.4055 | 0.2168 | 0.1472 | 0.0000 | 1.3804 | 0.7830 | 0.2312 | 0.0001 | 0.0565 | 0.0814 |
| 872 | P30711 | Glutathione S-transferase theta-1 (EC 2.5.1.18) (GST class-theta-1) (Glutathione transferase T1-1) | GSTT1 | 11 | 8 | 1.3932 | 0.2979 | 0.0839 | 0.0582 | 0.5495 | 0.1886 | 0.8017 | 0.4435 | 1.8707 | 0.1591 | 0.1294 | 0.0029 | 1.7061 | 0.1569 | 0.1107 | 0.0158 | 0.8872 | 0.2564 | 0.9550 | 0.4915 | 0.1343 | 0.0016 | 1.1912 | 0.3668 | 0.3873 | 0.0519 | 1.9770 | 0.2372 |
| 873 | P09211 | Glutathione S-transferase P (EC 2.5.1.18) (GST class-pi) (GSTP1-1) | GSTP1 FAEES3 GST3 | 3 | 11 | 0.6982 | 0.4408 | 0.9036 | 0.8639 | 1.0280 | 0.4541 | 0.9376 | 0.8931 | 1.0965 | 0.8235 | 1.4060 | 0.7259 | 2.0893 | 0.1970 | 1.1482 | 0.9451 | 0.1888 | 0.1123 | 1.5417 | 0.4110 | 0.7870 | 0.5900 | 0.5598 | 0.3993 | 0.3105 | 0.1617 | 2.4660 | 0.0205 |
| 874 | P78417 | Glutathione S-transferase omega-1 (GSTO-1) (EC 2.5.1.18) (Glutathione S-transferase omega 1-1) (GSTO 1-1) (Glutathione-dependent dehydroascorbate reductase) (EC 1.8.5.1) (Monomethylarsonic acid reductase) (MMA(V) reductase) (EC 1.20.4.2) (S-(Phenacyl)glutathione reductase) (SPG-R) | GSTO1 GSTTLP28 | 4 | 24 | 2.4210 | 0.1106 | 0.4365 | 0.0110 | 3.3113 | 0.0858 | 0.6982 | 0.0947 | 0.2355 | 0.0989 | 0.5105 | 0.0460 | 2.8840 | 0.0603 | 0.3802 | 0.0837 | 2.0512 | 0.1815 | 0.4055 | 0.0178 | 0.3698 | 0.2584 | 0.7656 | 0.4806 | 1.9953 | 0.2030 | 0.7311 | 0.1741 |
| 875 | P09488 | Glutathione S-transferase Mu 1 (EC 2.5.1.18) (GST HB subunit 4) (GST class-mu 1) (GSTM1-1) (GSTM1a-1a) (GSTM1b-1b) (GTH4) | GSTM1 GST1 | 13 | 24 | 0.1368 | 0.0043 | 0.0288 | 0.0432 | 0.9204 | 0.4557 | 1.0280 | 0.1236 | 0.5012 | 0.2823 | 4.5290 | 0.0003 | 0.9204 | 0.9361 | 0.0268 | 0.0094 | 1.2023 | 0.5634 | 2.2080 | 0.0051 | 1.4060 | 0.0840 | 0.0283 | 0.0001 | 0.5445 | 0.8165 | 1.7219 | 0.0359 |
| 876 | Q9Y2Q3 | Glutathione S-transferase kappa 1 (EC 2.5.1.18) (GST 13-13) (GST class-kappa) (GSTK1-1) (hGSTK1) (Glutathione S-transferase subunit 13) | GSTK1 HDCMD47P | 29 | 16 | 0.6546 | 0.1733 | 0.6026 | 0.8062 | 0.5598 | 0.0126 | 0.6427 | 0.1384 | 0.9462 | 0.7507 | 0.9204 | 0.8887 | 1.4454 | 0.1987 | 0.8551 | 0.7522 | 0.7516 | 0.4333 | 0.9817 | 0.8568 | 0.3981 | 0.0037 | 0.9462 | 0.8963 | 0.4831 | 0.0316 | 1.1272 | 0.3765 |
| 877 | P08263 | Glutathione S-transferase A1 (EC 2.5.1.18) (GST HA subunit 1) (GST class-alpha member 1) (GST-epsilon) (GSTA1-1) (GTH1) [Cleaved into: Glutathione S-transferase A1, N-terminally processed] | GSTA1 | 11 | 111 | 0.0310 | 0.0001 | 1.4322 | 0.4952 | 0.0649 | 0.0000 | 0.9462 | 0.9821 | 0.0429 | 0.0000 | 0.9204 | 0.8919 | 1.7219 | 0.0014 | 0.7798 | 0.6587 | 0.0316 | 0.0002 | 0.8630 | 0.7756 | 0.0614 | 0.0001 | 1.3552 | 0.5439 | 0.0530 | 0.0000 | 1.5704 | 0.4455 |
| 878 | P48637 | Glutathione synthetase (GSH synthetase) (GSH-S) (EC 6.3.2.3) (Glutathione synthase) | GSS | 20 | 17 | 0.7112 | 0.6778 | 0.9036 | 0.6093 | 1.0093 | 0.3363 | 1.0471 | 0.8229 | 1.1066 | 0.6111 | 1.8535 | 0.2336 | 0.9638 | 0.9989 | 1.2023 | 0.1862 | 0.9290 | 0.6868 | 0.9638 | 0.7882 | 0.5916 | 0.0208 | 1.5849 | 0.0333 | 1.2023 | 0.4658 | 1.8707 | 0.0785 |
| 879 | P00390 | Glutathione reductase, mitochondrial (GR) (GRase) (EC 1.8.1.7) | GSR GLUR GRD1 | 107 | 13 | 0.8318 | 0.0302 | 1.3804 | 0.4998 | 1.1272 | 0.4189 | 0.7943 | 0.2001 | 1.1803 | 0.8373 | 2.6303 | 0.4622 | 0.3499 | 0.0004 | 1.3428 | 0.7355 | 1.1169 | 0.6799 | 1.4859 | 0.7000 | 0.0270 | 0.0000 | 1.3305 | 0.8247 | 0.1096 | 0.0000 | 2.2699 | 0.8932 |
| 880 | P15170 | Eukaryotic peptide chain release factor GTP-binding subunit ERF3A (Eukaryotic peptide chain release factor subunit 3a) (eRF3a) (G1 to S phase transition protein 1 homolog) | GSPT1 ERF3A | 13 | 5 | 1.0375 | 0.7347 | 0.9376 | 0.4511 | 0.9817 | 0.8773 | 1.0280 | 0.9621 | 1.0375 | 0.3962 | 1.0000 | 0.8421 | 1.0765 | 0.3751 | 1.0280 | 0.9917 | 1.0000 | 0.8562 | 1.0280 | 0.8343 | 1.0093 | 0.4577 | 0.9727 | 0.6500 | 0.9817 | 0.7521 | 1.0093 | 0.7816 |
| 881 | P06396 | Gelsolin (AGEL) (Actin-depolymerizing factor) (ADF) (Brevin) | GSN | 12 | 14 | 1.1588 | 0.3836 | 0.4487 | 0.0153 | 1.4322 | 0.7384 | 1.2023 | 0.5378 | 1.1482 | 0.8444 | 0.9550 | 0.7881 | 0.9638 | 0.4873 | 1.0471 | 0.9930 | 1.8030 | 0.9962 | 1.0965 | 0.4094 | 1.7701 | 0.5370 | 0.8395 | 0.1855 | 1.3932 | 0.8887 | 1.9953 | 0.1211 |
| 882 | P57764 | Gasdermin-D (Gasdermin domain-containing protein 1) | GSDMD DFNA5L GSDMDC1 FKSG10 | 5 | 7 | 2.8840 | 0.2243 | 1.5704 | 0.4636 | 2.1677 | 0.2939 | 1.8030 | 0.4857 | 2.4434 | 0.2608 | 1.8707 | 0.2064 | 2.2080 | 0.2879 | 2.3121 | 0.2039 | 2.4210 | 0.2621 | 1.5996 | 0.2839 | 2.5119 | 0.2546 | 1.7865 | 0.4132 | 3.6983 | 0.1842 | 2.5586 | 0.0802 |
| 883 | Q9HAV7 | GrpE protein homolog 1, mitochondrial (HMGE) (Mt-GrpE#1) | GRPEL1 GREPEL1 | 14 | 4 | 0.3631 | 0.0234 | 1.5704 | 0.4117 | 1.2359 | 0.4385 | 1.4191 | 0.7562 | 0.9120 | 0.6508 | 1.2023 | 0.9701 | 0.7656 | 0.7952 | 1.8197 | 0.2295 | 1.2474 | 0.7850 | 1.1912 | 0.9564 | 1.6444 | 0.0283 | 1.7219 | 0.4220 | 1.2474 | 0.9409 | 0.9290 | 0.5060 |
| 884 | Q9UBQ7 | Glyoxylate reductase/hydroxypyruvate reductase (EC 1.1.1.79) (EC 1.1.1.81) | GRHPR GLXR MSTP035 | 3 | 49 | 0.9638 | 0.9341 | 1.0280 | 0.8914 | 1.0280 | 0.6439 | 1.2823 | 0.6779 | 1.1912 | 0.3260 | 0.7727 | 0.1052 | 1.2134 | 0.2737 | 0.9817 | 0.3340 | 1.0965 | 0.4663 | 0.8710 | 0.2864 | 0.9727 | 0.8022 | 1.2134 | 0.5075 | 1.1482 | 0.4034 | 0.1432 | 0.0003 |
| 885 | P07203 | Glutathione peroxidase 1 (GPx-1) (GSHPx-1) (EC 1.11.1.9) (Cellular glutathione peroxidase) | GPX1 | 6 | 6 | 1.0093 | 0.5711 | 0.9036 | 0.3971 | 0.9204 | 0.5263 | 0.8017 | 0.3709 | 1.0186 | 0.6572 | 0.9550 | 0.8497 | 0.9908 | 0.3924 | 0.8954 | 0.4245 | 1.0000 | 0.7881 | 0.9120 | 0.4730 | 0.8872 | 0.5919 | 0.8954 | 0.5434 | 0.9204 | 0.6743 | 0.9376 | 0.6648 |
| 886 | P24298 | Alanine aminotransferase 1 (ALT1) (EC 2.6.1.2) (Glutamate pyruvate transaminase 1) (GPT 1) (Glutamic--alanine transaminase 1) (Glutamic--pyruvic transaminase 1) | GPT AAT1 GPT1 | 48 | 31 | 0.9204 | 0.7261 | 1.3804 | 0.6945 | 0.8091 | 0.2957 | 2.4210 | 0.0014 | 0.9120 | 0.7910 | 0.6194 | 0.0717 | 0.6252 | 0.2071 | 1.4997 | 0.1721 | 1.0568 | 0.6573 | 0.8472 | 0.4137 | 0.0437 | 0.0000 | 0.7379 | 0.2490 | 0.1585 | 0.0000 | 1.0765 | 0.9491 |
| 887 | P06744 | Glucose-6-phosphate isomerase (GPI) (EC 5.3.1.9) (Autocrine motility factor) (AMF) (Neuroleukin) (NLK) (Phosphoglucose isomerase) (PGI) (Phosphohexose isomerase) (PHI) (Sperm antigen 36) (SA-36) | GPI | 6 | 43 | 0.8395 | 0.2378 | 0.2312 | 0.0001 | 1.1272 | 0.7649 | 0.4571 | 0.0107 | 1.0666 | 0.9963 | 0.9120 | 0.7153 | 1.1803 | 0.6899 | 1.0375 | 0.8320 | 0.6026 | 0.0690 | 0.9908 | 0.6390 | 1.1588 | 0.7516 | 1.1695 | 0.5262 | 0.9290 | 0.4176 | 1.4060 | 0.0039 |
| 888 | Q9NQX3 | Gephyrin [Includes: Molybdopterin adenylyltransferase (MPT adenylyltransferase) (EC 2.7.7.75) (Domain G); Molybdopterin molybdenumtransferase (MPT Mo-transferase) (EC 2.10.1.1) (Domain E)] | GPHN GPH KIAA1385 | 30 | 11 | 1.0186 | 0.8521 | 1.9409 | 0.1961 | 1.0666 | 0.3759 | 1.9231 | 0.2172 | 1.4588 | 0.0094 | 1.9588 | 0.1858 | 1.2942 | 0.3605 | 1.9055 | 0.2205 | 0.2938 | 0.0001 | 1.6444 | 0.2329 | 0.3076 | 0.0005 | 1.6293 | 0.3484 | 0.6252 | 0.0055 | 1.5417 | 0.2235 |
| 889 | P21695 | Glycerol-3-phosphate dehydrogenase [NAD(+)], cytoplasmic (GPD-C) (GPDH-C) (EC 1.1.1.8) | GPD1 | 40 | 33 | 0.6138 | 0.0318 | 0.4285 | 0.0070 | 0.7311 | 0.0451 | 0.7244 | 0.0569 | 0.6252 | 0.1343 | 0.8318 | 0.0339 | 0.9638 | 0.6292 | 1.1376 | 0.7850 | 0.9036 | 0.4800 | 1.3552 | 0.8078 | 0.5702 | 0.0217 | 1.6749 | 0.2081 | 0.4325 | 0.0760 | 0.7112 | 0.0430 |
| 890 | P00505 | Aspartate aminotransferase, mitochondrial (mAspAT) (EC 2.6.1.1) (EC 2.6.1.7) (Fatty acid-binding protein) (FABP-1) (Glutamate oxaloacetate transaminase 2) (Kynurenine aminotransferase 4) (Kynurenine aminotransferase IV) (Kynurenine--oxoglutarate transaminase 4) (Kynurenine--oxoglutarate transaminase IV) (Plasma membrane-associated fatty acid-binding protein) (FABPpm) (Transaminase A) | GOT2 | 5 | 48 | 0.6730 | 0.7088 | 1.6293 | 0.4400 | 0.5297 | 0.2103 | 1.0765 | 0.0075 | 0.5916 | 0.5897 | 0.4055 | 0.0000 | 1.0568 | 0.7664 | 1.5704 | 0.4487 | 0.4875 | 0.6615 | 0.5702 | 0.0007 | 0.3945 | 0.5000 | 1.3677 | 0.5720 | 1.0765 | 0.9495 | 0.7798 | 0.0006 |
| 891 | P17174 | Aspartate aminotransferase, cytoplasmic (cAspAT) (EC 2.6.1.1) (EC 2.6.1.3) (Cysteine aminotransferase, cytoplasmic) (Cysteine transaminase, cytoplasmic) (cCAT) (Glutamate oxaloacetate transaminase 1) (Transaminase A) | GOT1 | 33 | 46 | 0.5105 | 0.0363 | 0.7178 | 0.7047 | 1.1066 | 0.8956 | 1.8880 | 0.0169 | 1.0280 | 0.9389 | 0.5808 | 0.1165 | 0.6427 | 0.0688 | 0.5546 | 0.1223 | 0.5861 | 0.0234 | 0.9290 | 0.9919 | 0.1202 | 0.0000 | 1.0568 | 0.8348 | 0.2249 | 0.0003 | 0.6730 | 0.5012 |
| 892 | Q9H8Y8 | Golgi reassembly-stacking protein 2 (GRS2) (Golgi phosphoprotein 6) (GOLPH6) (Golgi reassembly-stacking protein of 55 kDa) (GRASP55) (p59) | GORASP2 GOLPH6 | 55 | 3 | 0.7656 | 0.0339 | 1.0186 | 0.9281 | 0.9817 | 0.0682 | 1.0186 | 0.9377 | 0.5445 | 0.0045 | 0.8630 | 0.7026 | 1.2359 | 0.1278 | 0.7516 | 0.4897 | 1.4454 | 0.9457 | 1.0864 | 0.7944 | 0.2992 | 0.0000 | 1.0186 | 0.9220 | 0.2858 | 0.0001 | 1.0864 | 0.7928 |
| 893 | Q96EK6 | Glucosamine 6-phosphate N-acetyltransferase (EC 2.3.1.4) (Phosphoglucosamine acetylase) (Phosphoglucosamine transacetylase) | GNPNAT1 GNA1 | 55 | 3 | 0.7178 | 0.1522 | 0.6546 | 0.1500 | 0.2805 | 0.0000 | 0.8017 | 0.3359 | 0.3221 | 0.0001 | 0.8017 | 0.4590 | 2.0324 | 0.0000 | 0.7943 | 0.3530 | 1.2246 | 0.2529 | 0.7112 | 0.2153 | 0.4325 | 0.0000 | 0.6546 | 0.2089 | 0.6081 | 0.0003 | 0.7047 | 0.2617 |
| 894 | Q14749 | Glycine N-methyltransferase (EC 2.1.1.20) | GNMT | 4 | 15 | 1.5417 | 0.2751 | 0.0824 | 0.0002 | 1.3932 | 0.3875 | 0.8166 | 0.2113 | 1.5560 | 0.3147 | 0.1000 | 0.0011 | 1.4060 | 0.3323 | 0.2051 | 0.0016 | 1.7539 | 0.2301 | 0.8630 | 0.5100 | 1.7219 | 0.2849 | 0.6138 | 0.0822 | 1.4859 | 0.3152 | 0.1148 | 0.0005 |
| 895 | P63244 | Guanine nucleotide-binding protein subunit beta-2-like 1 (Cell proliferation-inducing gene 21 protein) (Guanine nucleotide-binding protein subunit beta-like protein 12.3) (Human lung cancer oncogene 7 protein) (HLC-7) (Receptor for activated C kinase) (Receptor of activated protein kinase C 1) (RACK1) [Cleaved into: Guanine nucleotide-binding protein subunit beta-2-like 1, N-terminally processed] | GNB2L1 HLC7 PIG21 | 6 | 23 | 0.6368 | 0.2230 | 0.6486 | 0.2301 | 0.6026 | 0.2106 | 1.4723 | 0.6279 | 0.6310 | 0.0404 | 1.4060 | 0.7032 | 0.6138 | 0.0486 | 0.8166 | 0.2186 | 0.6368 | 0.1155 | 1.4060 | 0.5129 | 0.4875 | 0.0189 | 0.8551 | 0.5261 | 0.4656 | 0.0786 | 1.3552 | 0.3402 |
| 896 | P62879 | Guanine nucleotide-binding protein G(I)/G(S)/G(T) subunit beta-2 (G protein subunit beta-2) (Transducin beta chain 2) | GNB2 | 17 | 10 | 1.0093 | 0.1343 | 1.1066 | 0.5736 | 0.9376 | 0.5483 | 1.1588 | 0.5223 | 0.5754 | 0.0186 | 1.2823 | 0.2929 | 0.5297 | 0.0028 | 1.1066 | 0.6739 | 0.5495 | 0.0063 | 1.1912 | 0.5437 | 0.0313 | 0.0001 | 0.9462 | 0.6913 | 0.0570 | 0.0000 | 0.9817 | 0.9528 |
| 897 | P62873 | Guanine nucleotide-binding protein G(I)/G(S)/G(T) subunit beta-1 (Transducin beta chain 1) | GNB1 | 23 | 7 | 1.5417 | 0.5403 | 0.7112 | 0.0155 | 1.6749 | 0.6241 | 0.7870 | 0.0885 | 2.3335 | 0.1845 | 0.9908 | 0.6833 | 2.3121 | 0.5285 | 1.0280 | 0.7457 | 1.7701 | 0.8304 | 0.6792 | 0.0711 | 3.1623 | 0.1478 | 0.9036 | 0.5404 | 1.7219 | 0.8140 | 0.8551 | 0.3660 |
| 898 | P63092 | Guanine nucleotide-binding protein G(s) subunit alpha isoforms short (Adenylate cyclase-stimulating G alpha protein) | GNAS GNAS1 GSP | 10 | 7 | 0.5702 | 0.1583 | 1.0965 | 0.7831 | 1.1169 | 0.6897 | 0.9550 | 0.9888 | 1.2942 | 0.4080 | 0.7586 | 0.6940 | 0.9908 | 0.9922 | 0.8472 | 0.5707 | 0.8710 | 0.6657 | 0.7870 | 0.5179 | 0.7244 | 0.3379 | 0.4613 | 0.1836 | 0.6252 | 0.2022 | 1.0568 | 0.9289 |
| 899 | P08754 | Guanine nucleotide-binding protein G(k) subunit alpha (G(i) alpha-3) | GNAI3 | 8 | 4 | 1.2246 | 0.6206 | 1.0765 | 0.8750 | 0.9120 | 0.8760 | 0.9036 | 0.8352 | 1.2474 | 0.6412 | 1.3428 | 0.5587 | 1.1066 | 0.8319 | 1.0280 | 0.8493 | 1.1066 | 0.7725 | 0.8954 | 0.7688 | 1.0000 | 0.9989 | 1.2706 | 0.3227 | 0.8472 | 0.8527 | 1.4997 | 0.1977 |
| 900 | P04899 | Guanine nucleotide-binding protein G(i) subunit alpha-2 (Adenylate cyclase-inhibiting G alpha protein) | GNAI2 GNAI2B | 8 | 16 | 1.6144 | 0.3272 | 1.3305 | 0.3501 | 1.4997 | 0.4084 | 1.2359 | 0.6327 | 1.1376 | 0.9900 | 0.9817 | 0.7607 | 0.2070 | 0.3036 | 0.8630 | 0.8612 | 0.5808 | 0.5368 | 1.2474 | 0.5231 | 1.1169 | 0.9781 | 0.9036 | 0.6802 | 1.3428 | 0.4375 | 1.7061 | 0.0721 |
| 901 | P63096 | Guanine nucleotide-binding protein G(i) subunit alpha-1 (Adenylate cyclase-inhibiting G alpha protein) | GNAI1 | 10 | 5 | 1.3804 | 0.5269 | 1.4191 | 0.4235 | 0.9120 | 0.9961 | 1.5849 | 0.5297 | 1.0864 | 0.7845 | 1.4191 | 0.6138 | 1.0666 | 0.7789 | 0.6194 | 0.8050 | 0.8318 | 0.8002 | 0.9376 | 0.8687 | 1.0000 | 0.9652 | 0.9638 | 0.8650 | 1.4060 | 0.5289 | 0.2249 | 0.4373 |
| 902 | P29992 | Guanine nucleotide-binding protein subunit alpha-11 (G alpha-11) (G-protein subunit alpha-11) (Guanine nucleotide-binding protein G(y) subunit alpha) | GNA11 GA11 | 19 | 3 | 0.6138 | 0.3579 | 0.7379 | 0.4466 | 1.2246 | 0.3685 | 0.5861 | 0.1924 | 0.5808 | 0.0932 | 0.8551 | 0.6789 | 0.4055 | 0.2182 | 0.6310 | 0.3153 | 0.6252 | 0.8184 | 0.8166 | 0.4497 | 1.3677 | 0.2482 | 0.7727 | 0.3820 | 1.0375 | 0.7318 | 0.7112 | 0.3435 |
| 903 | Q96IJ6 | Mannose-1-phosphate guanyltransferase alpha (GDP-mannose pyrophosphorylase A) (GMPP-alpha) (GTP-mannose-1-phosphate guanylyltransferase alpha) | GMPPA | 12 | 6 | 0.9550 | 0.9368 | 0.8872 | 0.7209 | 1.2823 | 0.5896 | 0.6730 | 0.5377 | 0.6368 | 0.4419 | 0.7112 | 0.5546 | 0.8630 | 0.8580 | 0.5649 | 0.5602 | 1.1376 | 0.7728 | 0.7178 | 0.9280 | 0.9908 | 0.9500 | 1.1169 | 0.4573 | 0.8017 | 0.6686 | 1.5417 | 0.1016 |
| 904 | Q8IVS8 | Glycerate kinase (EC 2.7.1.31) (HBeAg-binding protein 4) | GLYCTK HBEBP4 LP5910 | 5 | 13 | 0.4529 | 0.4653 | 1.3677 | 0.3646 | 1.8707 | 0.6146 | 1.5560 | 0.0925 | 1.5276 | 0.8287 | 0.4406 | 0.3097 | 1.4588 | 0.7931 | 1.0186 | 0.2927 | 1.0568 | 0.7873 | 0.3532 | 0.0241 | 1.6444 | 0.7991 | 1.4859 | 0.2532 | 2.1478 | 0.4790 | 0.1820 | 0.0892 |
| 905 | Q969I3 | Glycine N-acyltransferase-like protein 1 (EC 2.3.1.68) (Acyl-CoA:glycine N-acyltransferase-like protein 1) (Glutamine N-acyltransferase) | GLYATL1 GNAT | 4 | 3 | 1.0568 | 0.6757 | 0.8790 | 0.6142 | 0.5152 | 0.0443 | 0.5598 | 0.4191 | 1.3552 | 0.5846 | 0.5861 | 0.2953 | 0.7379 | 0.1794 | 1.0765 | 0.9565 | 0.5649 | 0.1867 | 0.5058 | 0.2159 | 0.5861 | 0.1819 | 0.6026 | 0.2890 | 1.1376 | 0.5646 | 0.4831 | 0.2578 |
| 906 | Q6IB77 | Glycine N-acyltransferase (EC 2.3.1.13) (EC 2.3.1.71) (Acyl-CoA:glycine N-acyltransferase) (AAc) (Aralkyl acyl-CoA N-acyltransferase) (Aralkyl acyl-CoA:amino acid N-acyltransferase) (Benzoyl-coenzyme A:glycine N-acyltransferase) (Glycine N-benzoyltransferase) (HRP-1(CLP)) | GLYAT ACGNAT CAT GAT | 11 | 31 | 1.7865 | 0.2009 | 1.1066 | 0.3802 | 1.4454 | 0.9674 | 1.2942 | 0.8902 | 1.2359 | 0.9972 | 0.6310 | 0.0318 | 1.0568 | 0.7640 | 0.4656 | 0.0012 | 1.3428 | 0.9631 | 0.5297 | 0.0021 | 0.1127 | 0.0185 | 0.6427 | 0.0030 | 0.3981 | 0.1058 | 0.2089 | 0.0000 |
| 907 | P15104 | Glutamine synthetase (GS) (EC 6.3.1.2) (Glutamate decarboxylase) (EC 4.1.1.15) (Glutamate--ammonia ligase) | GLUL GLNS | 6 | 10 | 0.8551 | 0.3803 | 2.4434 | 0.2859 | 0.5649 | 0.4193 | 2.1281 | 0.2116 | 0.9376 | 0.9412 | 1.4997 | 0.4647 | 1.1588 | 0.4252 | 4.6559 | 0.0017 | 1.4588 | 0.2076 | 0.7798 | 0.6165 | 0.2291 | 0.0270 | 1.3552 | 0.8525 | 0.6427 | 0.8485 | 1.1376 | 0.9097 |
| 908 | P00367 | Glutamate dehydrogenase 1, mitochondrial (GDH 1) (EC 1.4.1.3) | GLUD1 GLUD | 35 | 139 | 0.6310 | 0.1314 | 0.6252 | 0.5571 | 1.0666 | 0.3424 | 0.8954 | 0.4377 | 0.8790 | 0.7325 | 0.6546 | 0.1035 | 0.9120 | 0.8691 | 1.6144 | 0.2063 | 0.8472 | 0.6054 | 0.6194 | 0.2030 | 0.0718 | 0.0001 | 0.4875 | 0.0027 | 0.2291 | 0.0002 | 0.1343 | 0.0010 |
| 909 | Q9UI32 | Glutaminase liver isoform, mitochondrial (GLS) (EC 3.5.1.2) (L-glutaminase) (L-glutamine amidohydrolase) | GLS2 GA | 7 | 5 | 0.8318 | 0.7051 | 0.9036 | 0.5283 | 0.9204 | 0.7173 | 1.3428 | 0.8759 | 1.2134 | 0.4557 | 0.2630 | 0.0129 | 0.2582 | 0.0188 | 1.2023 | 0.9865 | 0.2355 | 0.0154 | 1.0864 | 0.7791 | 0.8017 | 0.7839 | 0.5495 | 0.1234 | 0.6486 | 0.4381 | 0.4285 | 0.1356 |
| 910 | Q86SX6 | Glutaredoxin-related protein 5, mitochondrial (Monothiol glutaredoxin-5) | GLRX5 C14orf87 | 145 | 4 | 0.9817 | 0.1263 | 1.0568 | 0.3668 | 0.7447 | 0.0025 | 0.5970 | 0.4698 | 0.5297 | 0.0000 | 1.1376 | 0.6628 | 0.7047 | 0.0002 | 0.6138 | 0.7126 | 0.7516 | 0.0008 | 0.8872 | 0.9760 | 0.0497 | 0.0000 | 1.2134 | 0.6111 | 0.0809 | 0.0000 | 0.5297 | 0.3705 |
| 911 | P35754 | Glutaredoxin-1 (Thioltransferase-1) (TTase-1) | GLRX GRX | 3 | 14 | 0.0515 | 0.0799 | 1.5704 | 0.7119 | 1.4723 | 0.4946 | 1.0666 | 0.9305 | 1.0000 | 0.9869 | 2.0701 | 0.6197 | 0.8872 | 0.8338 | 0.4018 | 0.6232 | 0.8091 | 0.6842 | 1.4723 | 0.6075 | 0.7047 | 0.5330 | 1.4723 | 0.7137 | 0.0113 | 0.0600 | 0.6607 | 0.6712 |
| 912 | Q9HC38 | Glyoxalase domain-containing protein 4 | GLOD4 C17orf25 CGI-150 My027 | 3 | 16 | 14.0605 | 0.5097 | 0.7727 | 0.6755 | 6.0813 | 0.7636 | 0.7178 | 0.2019 | 9.5499 | 0.5546 | 0.8630 | 0.4882 | 11.5878 | 0.5322 | 0.7656 | 0.1381 | 10.2802 | 0.5705 | 1.0000 | 0.6594 | 14.9969 | 0.4940 | 0.9462 | 0.8747 | 16.4437 | 0.4555 | 0.7112 | 0.4113 |
| 913 | Q04760 | Lactoylglutathione lyase (EC 4.4.1.5) (Aldoketomutase) (Glyoxalase I) (Glx I) (Ketone-aldehyde mutase) (Methylglyoxalase) (S-D-lactoylglutathione methylglyoxal lyase) | GLO1 | 18 | 10 | 1.0093 | 0.8927 | 0.2938 | 0.0171 | 0.8872 | 0.9096 | 1.0186 | 0.8440 | 0.7586 | 0.7539 | 0.8017 | 0.2975 | 1.2023 | 0.8098 | 0.7447 | 0.4534 | 0.8551 | 0.9856 | 0.6310 | 0.1693 | 0.5495 | 0.4272 | 1.0375 | 0.8113 | 0.6427 | 0.5013 | 0.1854 | 0.0005 |
| 914 | P23378 | Glycine dehydrogenase (decarboxylating), mitochondrial (EC 1.4.4.2) (Glycine cleavage system P protein) (Glycine decarboxylase) (Glycine dehydrogenase (aminomethyl-transferring)) | GLDC GCSP | 14 | 40 | 0.8318 | 0.3508 | 0.2729 | 0.0000 | 0.8091 | 0.4590 | 0.7727 | 0.0102 | 1.2023 | 0.2585 | 0.7311 | 0.0003 | 0.9550 | 0.9680 | 1.6749 | 0.0002 | 0.8017 | 0.4434 | 1.5136 | 0.0134 | 0.4786 | 0.0389 | 0.2466 | 0.0000 | 0.5012 | 0.0236 | 0.0511 | 0.0000 |
| 915 | P16278 | Beta-galactosidase (EC 3.2.1.23) (Acid beta-galactosidase) (Lactase) (Elastin receptor 1) | GLB1 ELNR1 | 9 | 7 | 1.1912 | 0.7482 | 0.6026 | 0.1006 | 0.9817 | 0.6538 | 0.8472 | 0.2651 | 1.5996 | 0.3785 | 0.9638 | 0.9017 | 1.2134 | 0.7542 | 0.7516 | 0.1524 | 1.1169 | 0.4415 | 0.7379 | 0.3643 | 0.2377 | 0.0024 | 0.8710 | 0.3612 | 0.3192 | 0.0029 | 0.8954 | 0.3340 |
| 916 | Q9NUV9 | GTPase IMAP family member 4 (Immunity-associated nucleotide 1 protein) (IAN-1) (hIAN1) (Immunity-associated protein 4) | GIMAP4 IAN1 IMAP4 MSTP062 | 30 | 3 | 0.4831 | 0.0000 | 0.1445 | 0.0945 | 0.1660 | 0.0000 | 0.2188 | 0.1395 | 0.3698 | 0.0000 | 0.8630 | 0.6813 | 0.5916 | 0.0000 | 0.6668 | 0.3653 | 0.1629 | 0.0000 | 1.1803 | 0.9057 | 0.0773 | 0.0000 | 0.1854 | 0.1677 | 0.2051 | 0.0000 | 1.2589 | 0.9005 |
| 917 | P36269 | Gamma-glutamyltransferase 5 (GGT 5) (EC 2.3.2.2) (Gamma-glutamyl transpeptidase-related enzyme) (GGT-rel) (Gamma-glutamyltransferase-like activity 1) (Gamma-glutamyltranspeptidase 5) (Glutathione hydrolase 5) (EC 3.4.19.13) (Leukotriene-C4 hydrolase) (EC 3.4.19.14) [Cleaved into: Gamma-glutamyltransferase 5 heavy chain; Gamma-glutamyltransferase 5 light chain] | GGT5 GGTLA1 | 4 | 12 | 0.3532 | 0.0675 | 1.7539 | 0.0538 | 0.9376 | 0.8793 | 1.8535 | 0.0385 | 0.7586 | 0.5242 | 2.1478 | 0.0238 | 1.4191 | 0.4195 | 1.8197 | 0.0758 | 0.3981 | 0.2942 | 2.0893 | 0.0977 | 1.5276 | 0.3044 | 1.3428 | 0.1431 | 0.5546 | 0.1708 | 2.1878 | 0.0093 |
| 918 | Q92820 | Gamma-glutamyl hydrolase (EC 3.4.19.9) (Conjugase) (GH) (Gamma-Glu-X carboxypeptidase) | GGH | 3 | 7 | 2.9376 | 0.6281 | 0.5200 | 0.4018 | 2.5586 | 0.7315 | 1.9231 | 0.4217 | 2.1086 | 0.9983 | 0.8017 | 0.7961 | 2.3988 | 0.8606 | 1.7865 | 0.6079 | 2.2699 | 0.7816 | 0.8166 | 0.3929 | 5.3456 | 0.2346 | 1.3932 | 0.7339 | 4.6132 | 0.3012 | 0.3311 | 0.0905 |
| 919 | Q06210 | Glutamine--fructose-6-phosphate aminotransferase [isomerizing] 1 (EC 2.6.1.16) (D-fructose-6-phosphate amidotransferase 1) (Glutamine:fructose-6-phosphate amidotransferase 1) (GFAT 1) (GFAT1) (Hexosephosphate aminotransferase 1) | GFPT1 GFAT GFPT | 14 | 4 | 0.6668 | 0.1831 | 1.9770 | 0.2831 | 1.8880 | 0.0098 | 1.2823 | 0.8181 | 1.0093 | 0.6409 | 1.6596 | 0.3356 | 0.1259 | 0.0015 | 1.0471 | 0.7957 | 1.2942 | 0.2345 | 1.4588 | 0.6567 | 0.9376 | 0.6248 | 1.7061 | 0.0562 | 1.2359 | 0.7856 | 1.2823 | 0.5652 |
| 920 | P50395 | Rab GDP dissociation inhibitor beta (Rab GDI beta) (Guanosine diphosphate dissociation inhibitor 2) (GDI-2) | GDI2 RABGDIB | 6 | 16 | 0.8091 | 0.3489 | 0.6427 | 0.2096 | 0.7112 | 0.2833 | 0.7870 | 0.4472 | 0.7870 | 0.7696 | 1.4588 | 0.7271 | 0.9376 | 0.7947 | 0.7379 | 0.0444 | 0.7656 | 0.6703 | 0.9376 | 0.4369 | 0.9120 | 0.9812 | 0.7586 | 0.3066 | 0.8472 | 0.6676 | 1.0965 | 0.6218 |
| 921 | P31150 | Rab GDP dissociation inhibitor alpha (Rab GDI alpha) (Guanosine diphosphate dissociation inhibitor 1) (GDI-1) (Oligophrenin-2) (Protein XAP-4) | GDI1 GDIL OPHN2 RABGDIA XAP4 | 3 | 24 | 2.7040 | 0.5210 | 0.6427 | 0.1578 | 2.5586 | 0.7528 | 0.6730 | 0.0570 | 5.7016 | 0.1662 | 0.9290 | 0.8016 | 4.9659 | 0.2084 | 0.7112 | 0.1753 | 3.1623 | 0.5399 | 0.8630 | 0.2867 | 5.4450 | 0.1473 | 0.7311 | 0.3251 | 4.4463 | 0.2258 | 0.6138 | 0.1988 |
| 922 | P16260 | Graves disease carrier protein (GDC) (Graves disease autoantigen) (GDA) (Mitochondrial solute carrier protein homolog) (Solute carrier family 25 member 16) | SLC25A16 GDA | 10 | 14 | 1.2474 | 0.3034 | 1.1482 | 0.2613 | 0.8395 | 0.6638 | 0.9120 | 0.6384 | 0.8630 | 0.3724 | 1.2359 | 0.1891 | 1.4060 | 0.2806 | 0.9550 | 0.7844 | 1.2359 | 0.2043 | 1.2023 | 0.1984 | 2.2699 | 0.0640 | 0.9638 | 0.8509 | 1.4723 | 0.2090 | 0.9638 | 0.8127 |
| 923 | Q9Y2T3 | Guanine deaminase (Guanase) (Guanine aminase) (EC 3.5.4.3) (Guanine aminohydrolase) (GAH) (p51-nedasin) | GDA KIAA1258 | 29 | 13 | 0.5916 | 0.0699 | 0.7586 | 0.4641 | 0.9290 | 0.9326 | 0.8630 | 0.8468 | 0.8551 | 0.8223 | 0.7656 | 0.0699 | 0.5754 | 0.2495 | 2.4889 | 0.1387 | 0.7516 | 0.4401 | 1.8880 | 0.3380 | 0.9376 | 0.7687 | 1.8030 | 0.3692 | 0.6310 | 0.3314 | 1.2023 | 0.7020 |
| 924 | P23434 | Glycine cleavage system H protein, mitochondrial (Lipoic acid-containing protein) | GCSH | 12 | 6 | 1.8030 | 0.1539 | 0.4018 | 0.6288 | 1.2246 | 0.7753 | 0.8472 | 0.9273 | 1.2823 | 0.3559 | 0.8395 | 0.8478 | 1.4454 | 0.4686 | 1.5136 | 0.7132 | 1.1066 | 0.7884 | 1.0471 | 0.8952 | 1.7378 | 0.1778 | 1.6904 | 0.7003 | 0.7656 | 0.6527 | 0.9376 | 0.9896 |
| 925 | Q92616 | Translational activator GCN1 (HsGCN1) (GCN1-like protein 1) | GCN1L1 KIAA0219 | 14 | 10 | 0.7586 | 0.0487 | 2.2284 | 0.3942 | 1.4322 | 0.2378 | 2.4434 | 0.2592 | 1.0186 | 0.9725 | 3.1333 | 0.1924 | 1.3552 | 0.4802 | 0.6486 | 0.5798 | 0.5346 | 0.0165 | 3.4041 | 0.1674 | 0.5754 | 0.0055 | 1.4322 | 0.2967 | 0.4920 | 0.0016 | 2.1086 | 0.2667 |
| 926 | P48506 | Glutamate--cysteine ligase catalytic subunit (EC 6.3.2.2) (GCS heavy chain) (Gamma-ECS) (Gamma-glutamylcysteine synthetase) | GCLC GLCL GLCLC | 6 | 16 | 0.8710 | 0.6675 | 0.8241 | 0.5177 | 1.0666 | 0.9045 | 1.7061 | 0.6109 | 0.7586 | 0.8032 | 1.0186 | 0.2491 | 1.1482 | 0.9391 | 0.4613 | 0.1028 | 0.9817 | 0.8825 | 1.0864 | 0.9721 | 0.1472 | 0.1988 | 1.2246 | 0.4076 | 0.2148 | 0.2020 | 0.8710 | 0.2867 |
| 927 | P30047 | GTP cyclohydrolase 1 feedback regulatory protein (GFRP) (GTP cyclohydrolase I feedback regulatory protein) (p35) | GCHFR GFRP | 12 | 9 | 1.1912 | 0.4007 | 1.0965 | 0.7400 | 2.7290 | 0.0585 | 0.7656 | 0.4662 | 1.2246 | 0.9081 | 0.5495 | 0.6066 | 1.7701 | 0.1288 | 0.5152 | 0.4465 | 0.9462 | 0.6245 | 0.4246 | 0.3907 | 1.4060 | 0.3454 | 0.7112 | 0.8554 | 2.4889 | 0.0775 | 0.5248 | 0.3716 |
| 928 | Q92947 | Glutaryl-CoA dehydrogenase, mitochondrial (GCD) (EC 1.3.8.6) | GCDH | 11 | 21 | 1.2359 | 0.6467 | 0.7727 | 0.5824 | 1.5849 | 0.3754 | 1.6904 | 0.2366 | 0.8017 | 0.5264 | 0.7870 | 0.2181 | 0.9908 | 0.4328 | 1.2246 | 0.3890 | 1.3183 | 0.7536 | 1.0280 | 0.7799 | 0.7943 | 0.1523 | 0.9204 | 0.5616 | 1.3552 | 0.9536 | 0.2559 | 0.0201 |
| 929 | P02774 | Vitamin D-binding protein (DBP) (VDB) (Gc protein-derived macrophage activating factor) (Gc-MAF) (GcMAF) (Gc-globulin) (Group-specific component) (Gc) (Vitamin D-binding protein-macrophage activating factor) (DBP-maf) | GC | 11 | 9 | 0.6668 | 0.4773 | 1.4191 | 0.5452 | 0.6026 | 0.4027 | 0.4169 | 0.0917 | 1.2706 | 0.7158 | 1.0471 | 0.8976 | 0.9376 | 0.9507 | 1.1272 | 0.7283 | 0.7379 | 0.6450 | 0.8710 | 0.3675 | 0.3162 | 0.1981 | 1.2023 | 0.9520 | 0.2208 | 0.1266 | 0.7447 | 0.0931 |
| 930 | P32455 | Guanylate-binding protein 1 (EC 3.6.5.-) (GTP-binding protein 1) (GBP-1) (HuGBP-1) (Guanine nucleotide-binding protein 1) (Interferon-induced guanylate-binding protein 1) | GBP1 | 25 | 9 | 1.1695 | 0.7596 | 0.9817 | 0.3420 | 1.2023 | 0.5461 | 0.7586 | 0.7446 | 0.6252 | 0.4527 | 2.5351 | 0.1314 | 1.6144 | 0.1694 | 0.4487 | 0.3193 | 0.9376 | 0.5431 | 1.7865 | 0.1831 | 0.1614 | 0.0035 | 1.1169 | 0.9401 | 0.2489 | 0.0399 | 3.5645 | 0.0780 |
| 931 | Q92538 | Golgi-specific brefeldin A-resistance guanine nucleotide exchange factor 1 (BFA-resistant GEF 1) | GBF1 KIAA0248 | 9 | 7 | 1.5417 | 0.7320 | 0.7943 | 0.7214 | 1.3062 | 0.9086 | 1.1169 | 0.6947 | 3.0479 | 0.2397 | 0.7656 | 0.6544 | 2.0893 | 0.5065 | 0.5495 | 0.3662 | 2.3988 | 0.6072 | 0.5808 | 0.4437 | 5.7544 | 0.1051 | 0.8091 | 0.7484 | 4.4463 | 0.1495 | 1.2823 | 0.5532 |
| 932 | Q04446 | 1,4-alpha-glucan-branching enzyme (EC 2.4.1.18) (Brancher enzyme) (Glycogen-branching enzyme) | GBE1 | 6 | 3 | 0.5861 | 0.4085 | 0.8630 | 0.4597 | 0.5649 | 0.3867 | 0.8318 | 0.4429 | 0.8395 | 0.7440 | 0.8472 | 0.5114 | 0.9638 | 0.9617 | 1.0186 | 0.8186 | 0.6310 | 0.4563 | 0.8954 | 0.8847 | 1.0093 | 0.9770 | 0.8395 | 0.5833 | 0.4966 | 0.3270 | 0.8472 | 0.9691 |
| 933 | P50440 | Glycine amidinotransferase, mitochondrial (EC 2.1.4.1) (L-arginine:glycine amidinotransferase) (Transamidinase) | GATM AGAT | 3 | 58 | 2.0893 | 0.2477 | 0.6138 | 0.0967 | 0.0991 | 0.1296 | 0.3311 | 0.0014 | 0.1225 | 0.1561 | 1.3062 | 0.0826 | 1.5704 | 0.3554 | 0.6486 | 0.0728 | 2.9376 | 0.1781 | 0.2333 | 0.0001 | 5.9704 | 0.1139 | 0.7870 | 0.2493 | 1.7061 | 0.3246 | 0.4571 | 0.0123 |
| 934 | P22102 | Trifunctional purine biosynthetic protein adenosine-3 [Includes: Phosphoribosylamine--glycine ligase (EC 6.3.4.13) (Glycinamide ribonucleotide synthetase) (GARS) (Phosphoribosylglycinamide synthetase); Phosphoribosylformylglycinamidine cyclo-ligase (EC 6.3.3.1) (AIR synthase) (AIRS) (Phosphoribosyl-aminoimidazole synthetase); Phosphoribosylglycinamide formyltransferase (EC 2.1.2.2) (5'-phosphoribosylglycinamide transformylase) (GAR transformylase) (GART)] | GART PGFT PRGS | 21 | 7 | 0.9376 | 0.5102 | 0.6427 | 0.2206 | 1.3552 | 0.5814 | 0.7516 | 0.7019 | 1.3677 | 0.6663 | 1.2359 | 0.6093 | 1.8880 | 0.1515 | 1.4060 | 0.2007 | 0.8790 | 0.0997 | 1.0568 | 0.2375 | 0.2805 | 0.0008 | 0.8017 | 0.7160 | 0.3373 | 0.0046 | 0.9204 | 0.8541 |
| 935 | P41250 | Glycine--tRNA ligase (EC 6.1.1.14) (Diadenosine tetraphosphate synthetase) (AP-4-A synthetase) (Glycyl-tRNA synthetase) (GlyRS) | GARS | 58 | 6 | 0.5916 | 0.0067 | 1.0093 | 0.7140 | 0.3837 | 0.0002 | 0.9036 | 0.5879 | 1.2359 | 0.0615 | 0.9120 | 0.8203 | 0.9550 | 0.6559 | 0.9376 | 0.8859 | 0.4246 | 0.0055 | 0.9204 | 0.9337 | 0.0738 | 0.0000 | 1.0093 | 0.6931 | 0.1977 | 0.0000 | 1.0765 | 0.5225 |
| 936 | P04406 | Glyceraldehyde-3-phosphate dehydrogenase (GAPDH) (EC 1.2.1.12) (Peptidyl-cysteine S-nitrosylase GAPDH) (EC 2.6.99.-) | GAPDH GAPD CDABP0047 OK/SW-cl.12 | 7 | 104 | 0.9908 | 0.7963 | 1.4859 | 0.8317 | 1.0186 | 0.9747 | 2.0512 | 0.5319 | 0.9638 | 0.7308 | 2.5119 | 0.1321 | 1.4060 | 0.1032 | 1.6904 | 0.9889 | 0.7311 | 0.4874 | 0.4920 | 0.0122 | 1.0568 | 0.7825 | 2.3988 | 0.1589 | 1.1482 | 0.7750 | 2.6546 | 0.0248 |
| 937 | Q14697 | Neutral alpha-glucosidase AB (EC 3.2.1.84) (Alpha-glucosidase 2) (Glucosidase II subunit alpha) | GANAB G2AN KIAA0088 | 7 | 50 | 0.6081 | 0.3339 | 1.3183 | 0.8124 | 0.7178 | 0.3476 | 1.5996 | 0.3656 | 0.8710 | 0.4892 | 1.3677 | 0.8796 | 0.8710 | 0.5466 | 1.3428 | 0.9177 | 0.6368 | 0.0898 | 1.4454 | 0.4850 | 1.3428 | 0.6389 | 1.2359 | 0.9264 | 0.7244 | 0.0932 | 0.9908 | 0.3525 |
| 938 | Q14353 | Guanidinoacetate N-methyltransferase (EC 2.1.1.2) | GAMT | 128 | 18 | 0.4406 | 0.0118 | 0.6730 | 0.4259 | 1.5136 | 0.3566 | 1.0965 | 0.3539 | 1.3932 | 0.8024 | 1.4454 | 0.1566 | 2.6062 | 0.0326 | 1.1588 | 0.6696 | 1.0864 | 0.4415 | 0.9036 | 0.9654 | 1.5849 | 0.5327 | 1.0280 | 0.6606 | 1.1588 | 0.2551 | 0.8790 | 0.4445 |
| 939 | P07902 | Galactose-1-phosphate uridylyltransferase (Gal-1-P uridylyltransferase) (EC 2.7.7.12) (UDP-glucose--hexose-1-phosphate uridylyltransferase) | GALT | 62 | 6 | 1.3552 | 0.1730 | 1.9409 | 0.1891 | 1.9055 | 0.0179 | 1.5136 | 0.4347 | 0.9120 | 0.7423 | 3.0761 | 0.0278 | 1.1588 | 0.7954 | 0.7798 | 0.8790 | 1.2823 | 0.2713 | 0.4613 | 0.9970 | 0.9036 | 0.4283 | 2.2080 | 0.1298 | 0.8091 | 0.7010 | 1.9770 | 0.0868 |
| 940 | Q96C23 | Aldose 1-epimerase (EC 5.1.3.3) (Galactose mutarotase) | GALM BLOCK25 | 17 | 10 | 0.9376 | 0.8326 | 0.4742 | 0.2361 | 0.7112 | 0.4234 | 1.3428 | 0.2232 | 1.2474 | 0.4639 | 1.1376 | 0.6489 | 1.3932 | 0.3058 | 0.4613 | 0.3221 | 0.9036 | 0.7353 | 2.4660 | 0.0155 | 0.4786 | 0.5801 | 1.0471 | 0.8563 | 0.3664 | 0.1677 | 0.8241 | 0.7662 |
| 941 | P51570 | Galactokinase (EC 2.7.1.6) (Galactose kinase) | GALK1 GALK | 6 | 30 | 0.5495 | 0.6058 | 0.5152 | 0.0427 | 1.0864 | 0.9419 | 0.7178 | 0.4875 | 1.0965 | 0.9123 | 0.7447 | 0.1965 | 0.4875 | 0.3462 | 1.3804 | 0.3045 | 0.7047 | 0.9619 | 1.9770 | 0.0095 | 0.6310 | 0.4339 | 3.2509 | 0.0000 | 0.5916 | 0.3303 | 0.8472 | 0.6154 |
| 942 | Q14376 | UDP-glucose 4-epimerase (EC 5.1.3.2) (Galactowaldenase) (UDP-N-acetylgalactosamine 4-epimerase) (UDP-GalNAc 4-epimerase) (UDP-N-acetylglucosamine 4-epimerase) (UDP-GlcNAc 4-epimerase) (EC 5.1.3.7) (UDP-galactose 4-epimerase) | GALE | 9 | 16 | 1.3932 | 0.3243 | 0.4325 | 0.0678 | 1.1066 | 0.1747 | 0.6855 | 0.4051 | 1.0666 | 0.1298 | 0.4875 | 0.0742 | 1.0280 | 0.2352 | 0.9550 | 0.6264 | 0.9204 | 0.1100 | 0.5248 | 0.1956 | 0.4446 | 0.0023 | 0.8954 | 0.6959 | 0.5598 | 0.0073 | 0.3251 | 0.0373 |
| 943 | O14976 | Cyclin-G-associated kinase (EC 2.7.11.1) | GAK | 27 | 3 | 0.7586 | 0.3629 | 1.7219 | 0.2826 | 1.1803 | 0.0951 | 2.9923 | 0.2266 | 1.8365 | 0.0045 | 1.9409 | 0.3590 | 0.4055 | 0.2150 | 1.7378 | 0.4010 | 0.9120 | 0.8202 | 1.8880 | 0.3289 | 0.0597 | 0.0002 | 2.1878 | 0.2917 | 0.7178 | 0.1950 | 2.4660 | 0.2744 |
| 944 | P10253 | Lysosomal alpha-glucosidase (EC 3.2.1.20) (Acid maltase) (Aglucosidase alfa) [Cleaved into: 76 kDa lysosomal alpha-glucosidase; 70 kDa lysosomal alpha-glucosidase] | GAA | 10 | 14 | 0.7311 | 0.8134 | 1.5276 | 0.6755 | 0.5970 | 0.2887 | 3.0200 | 0.0246 | 0.5248 | 0.2363 | 1.6749 | 0.3653 | 0.5970 | 0.2171 | 1.5276 | 0.2504 | 0.5861 | 0.5616 | 2.0512 | 0.0946 | 0.1459 | 0.0801 | 2.2284 | 0.1241 | 0.3532 | 0.0923 | 1.3305 | 0.8898 |
| 945 | P11413 | Glucose-6-phosphate 1-dehydrogenase (G6PD) (EC 1.1.1.49) | G6PD | 3 | 3 | 1.5136 | 0.2754 | 1.2023 | 0.5344 | 4.2462 | 0.1054 | 0.4699 | 0.2892 | 1.6144 | 0.5352 | 1.1482 | 0.6930 | 4.7863 | 0.0894 | 0.5445 | 0.3392 | 4.5709 | 0.0977 | 0.7379 | 0.7101 | 6.1376 | 0.0573 | 0.4786 | 0.3797 | 6.7920 | 0.0452 | 0.8472 | 0.7407 |
| 946 | Q13283 | Ras GTPase-activating protein-binding protein 1 (G3BP-1) (EC 3.6.4.12) (EC 3.6.4.13) (ATP-dependent DNA helicase VIII) (hDH VIII) (GAP SH3 domain-binding protein 1) | G3BP1 G3BP | 11 | 4 | 2.6303 | 0.0155 | 0.5297 | 0.1513 | 2.4889 | 0.0312 | 0.6194 | 0.2245 | 1.0186 | 0.7593 | 0.7244 | 0.4100 | 2.3550 | 0.0314 | 0.8395 | 0.5398 | 1.6293 | 0.3167 | 0.7178 | 0.3906 | 0.8241 | 0.5371 | 0.6546 | 0.3172 | 1.2706 | 0.1000 | 0.7943 | 0.5342 |
| 947 | P04066 | Tissue alpha-L-fucosidase (EC 3.2.1.51) (Alpha-L-fucosidase I) (Alpha-L-fucoside fucohydrolase 1) (Alpha-L-fucosidase 1) | FUCA1 Nbla10230 | 4 | 3 | 0.8954 | 0.3249 | 0.7516 | 0.3753 | 0.7516 | 0.0627 | 0.9290 | 0.8087 | 0.9204 | 0.5698 | 0.8710 | 0.8585 | 1.0864 | 0.6574 | 0.8472 | 0.5431 | 0.2704 | 0.0765 | 0.8954 | 0.8529 | 1.6596 | 0.4669 | 0.7586 | 0.3877 | 1.7701 | 0.2512 | 0.8091 | 0.6074 |
| 948 | Q96I24 | Far upstream element-binding protein 3 (FUSE-binding protein 3) | FUBP3 FBP3 | 5 | 3 | 1.9588 | 0.3537 | 1.7701 | 0.2384 | 1.6444 | 0.5735 | 1.8197 | 0.2850 | 2.8840 | 0.1700 | 1.0864 | 0.3007 | 1.4588 | 0.8328 | 1.1803 | 0.8852 | 1.0186 | 0.7675 | 0.7727 | 0.5773 | 3.4041 | 0.1245 | 1.5704 | 0.2145 | 3.7670 | 0.0976 | 1.8030 | 0.2337 |
| 949 | Q96AE4 | Far upstream element-binding protein 1 (FBP) (FUSE-binding protein 1) (DNA helicase V) (hDH V) | FUBP1 | 4 | 7 | 0.9120 | 0.9795 | 0.9727 | 0.6812 | 1.0000 | 0.5676 | 1.0093 | 0.5659 | 0.6081 | 0.0891 | 1.0186 | 0.6943 | 0.9290 | 0.8024 | 0.9376 | 0.7121 | 0.8395 | 0.7198 | 1.0186 | 0.4940 | 0.7379 | 0.7192 | 1.0093 | 0.7321 | 1.0765 | 0.5250 | 1.1695 | 0.2618 |
| 950 | P02792 | Ferritin light chain (Ferritin L subunit) | FTL | 8 | 11 | 0.8790 | 0.8120 | 2.6792 | 0.2075 | 1.8535 | 0.3709 | 5.2000 | 0.0467 | 0.8872 | 0.6301 | 2.1878 | 0.2244 | 1.1272 | 0.9543 | 4.9204 | 0.0499 | 1.2942 | 0.9897 | 0.3076 | 0.5789 | 1.0000 | 0.7191 | 0.2249 | 0.1993 | 1.4060 | 0.7990 | 4.3251 | 0.0371 |
| 951 | P02794 | Ferritin heavy chain (Ferritin H subunit) (EC 1.16.3.1) (Cell proliferation-inducing gene 15 protein) [Cleaved into: Ferritin heavy chain, N-terminally processed] | FTH1 FTH FTHL6 OK/SW-cl.84 PIG15 | 9 | 11 | 0.9376 | 0.6454 | 1.6749 | 0.1104 | 1.0864 | 0.8473 | 2.4210 | 0.0941 | 0.9376 | 0.6698 | 1.3428 | 0.4178 | 0.9204 | 0.9944 | 1.3677 | 0.2529 | 1.0568 | 0.7974 | 0.1770 | 0.0684 | 1.3183 | 0.4005 | 0.0488 | 0.0278 | 1.0280 | 0.8370 | 1.3932 | 0.2023 |
| 952 | O95954 | Formimidoyltransferase-cyclodeaminase (Formiminotransferase-cyclodeaminase) (FTCD) (LCHC1) [Includes: Glutamate formimidoyltransferase (EC 2.1.2.5) (Glutamate formiminotransferase) (Glutamate formyltransferase); Formimidoyltetrahydrofolate cyclodeaminase (EC 4.3.1.4) (Formiminotetrahydrofolate cyclodeaminase)] | FTCD | 14 | 122 | 2.9107 | 0.1875 | 0.6982 | 0.1405 | 0.0711 | 0.0067 | 1.0765 | 0.2181 | 5.0582 | 0.0067 | 0.1343 | 0.0000 | 0.1306 | 0.0094 | 1.1169 | 0.2360 | 1.5849 | 0.4221 | 1.0000 | 0.4669 | 0.7311 | 0.9967 | 1.6904 | 0.0275 | 9.4624 | 0.0140 | 0.2148 | 0.0000 |
| 953 | Q16658 | Fascin (55 kDa actin-bundling protein) (Singed-like protein) (p55) | FSCN1 FAN1 HSN SNL | 10 | 7 | 1.4454 | 0.6150 | 0.9462 | 0.9354 | 0.1514 | 0.0383 | 0.8872 | 0.7616 | 1.3804 | 0.8202 | 0.7943 | 0.3481 | 0.3162 | 0.1112 | 0.8790 | 0.7350 | 0.4699 | 0.4377 | 0.9290 | 0.9023 | 0.5808 | 0.2272 | 0.9036 | 0.7353 | 1.8030 | 0.1942 | 0.9120 | 0.7342 |
| 954 | Q9H479 | Fructosamine-3-kinase (EC 2.7.1.-) | FN3K | 127 | 3 | 0.7379 | 0.1539 | 1.5276 | 0.3641 | 1.1169 | 0.3090 | 1.2474 | 0.5584 | 1.1066 | 0.0837 | 0.9908 | 0.9973 | 0.8318 | 0.9473 | 1.3183 | 0.4897 | 0.5754 | 0.0026 | 0.9817 | 0.9322 | 0.0453 | 0.0000 | 1.0186 | 0.9791 | 0.1738 | 0.0000 | 1.0864 | 0.7883 |
| 955 | P02751 | Fibronectin (FN) (Cold-insoluble globulin) (CIG) [Cleaved into: Anastellin; Ugl-Y1; Ugl-Y2; Ugl-Y3] | FN1 FN | 5 | 29 | 0.9908 | 0.9043 | 0.4246 | 0.1215 | 1.0375 | 0.7713 | 0.7311 | 0.6873 | 0.9817 | 0.9077 | 1.0000 | 0.7486 | 1.0000 | 0.8591 | 1.0568 | 0.8416 | 0.9462 | 0.8824 | 0.6486 | 0.2442 | 0.9204 | 0.7367 | 0.7047 | 0.3648 | 1.1066 | 0.5431 | 1.0666 | 0.7816 |
| 956 | P49326 | Dimethylaniline monooxygenase [N-oxide-forming] 5 (EC 1.14.13.8) (Dimethylaniline oxidase 5) (Hepatic flavin-containing monooxygenase 5) (FMO 5) | FMO5 | 57 | 23 | 0.4699 | 0.0161 | 1.5136 | 0.4324 | 0.8954 | 0.2412 | 1.9953 | 0.0888 | 0.9120 | 0.2638 | 0.7447 | 0.1075 | 0.3342 | 0.0003 | 0.7178 | 0.2383 | 0.7311 | 0.0153 | 0.2489 | 0.0053 | 2.2080 | 0.0000 | 1.2706 | 0.7522 | 1.2706 | 0.1766 | 0.2188 | 0.0001 |
| 957 | P31512 | Dimethylaniline monooxygenase [N-oxide-forming] 4 (EC 1.14.13.8) (Dimethylaniline oxidase 4) (Hepatic flavin-containing monooxygenase 4) (FMO 4) | FMO4 FMO2 | 31 | 7 | 1.4060 | 0.4621 | 1.4859 | 0.4115 | 1.3677 | 0.6936 | 0.7943 | 0.6863 | 2.1478 | 0.6569 | 1.3932 | 0.4078 | 1.0471 | 0.1981 | 1.2942 | 0.6271 | 1.4191 | 0.3186 | 0.6730 | 0.4057 | 4.0551 | 0.1118 | 0.3020 | 0.3925 | 3.0761 | 0.6185 | 0.8954 | 0.7697 |
| 958 | P31513 | Dimethylaniline monooxygenase [N-oxide-forming] 3 (EC 1.14.13.8) (Dimethylaniline oxidase 3) (FMO II) (FMO form 2) (Hepatic flavin-containing monooxygenase 3) (FMO 3) (Trimethylamine monooxygenase) (EC 1.14.13.148) | FMO3 | 26 | 44 | 1.7378 | 0.0280 | 2.8314 | 0.0042 | 1.3183 | 0.9868 | 1.7061 | 0.3053 | 0.4656 | 0.0272 | 0.9120 | 0.1873 | 1.5136 | 0.9855 | 0.1977 | 0.0000 | 1.4997 | 0.9949 | 1.0000 | 0.1707 | 0.1000 | 0.0000 | 1.4454 | 0.6620 | 0.1923 | 0.0000 | 0.9727 | 0.6503 |
| 959 | O75955 | Flotillin-1 | FLOT1 | 5 | 8 | 1.0093 | 0.9370 | 0.5248 | 0.6826 | 0.9638 | 0.8821 | 1.9055 | 0.2541 | 0.9817 | 0.9426 | 1.9588 | 0.2679 | 0.9376 | 0.6747 | 0.8166 | 0.9153 | 0.9376 | 0.7198 | 1.3062 | 0.6011 | 1.0186 | 0.8669 | 1.4723 | 0.5901 | 0.9204 | 0.7109 | 1.8197 | 0.2288 |
| 960 | O75369 | Filamin-B (FLN-B) (ABP-278) (ABP-280 homolog) (Actin-binding-like protein) (Beta-filamin) (Filamin homolog 1) (Fh1) (Filamin-3) (Thyroid autoantigen) (Truncated actin-binding protein) (Truncated ABP) | FLNB FLN1L FLN3 TABP TAP | 49 | 83 | 0.7311 | 0.0260 | 1.3552 | 0.1522 | 1.5136 | 0.0452 | 1.2246 | 0.3098 | 1.4997 | 0.1496 | 1.2023 | 0.2754 | 0.6730 | 0.0440 | 0.7798 | 0.1297 | 0.2489 | 0.0003 | 0.8551 | 0.5813 | 0.8472 | 0.0698 | 1.4060 | 0.1098 | 1.0375 | 0.4416 | 0.9036 | 0.2131 |
| 961 | P21333 | Filamin-A (FLN-A) (Actin-binding protein 280) (ABP-280) (Alpha-filamin) (Endothelial actin-binding protein) (Filamin-1) (Non-muscle filamin) | FLNA FLN FLN1 | 5 | 87 | 0.8091 | 0.3065 | 0.4406 | 0.0402 | 0.8954 | 0.4078 | 2.8314 | 0.0000 | 0.8091 | 0.1828 | 0.6792 | 0.1755 | 0.9817 | 0.8637 | 1.1482 | 0.2964 | 0.9120 | 0.6113 | 0.4875 | 0.0019 | 0.9462 | 0.7590 | 0.2559 | 0.0000 | 0.7798 | 0.1075 | 1.5560 | 0.0025 |
| 962 | Q13045 | Protein flightless-1 homolog | FLII FLIL | 85 | 3 | 1.1376 | 0.8500 | 2.9107 | 0.0336 | 1.1695 | 0.7261 | 2.5823 | 0.0556 | 1.1803 | 0.4759 | 1.7701 | 0.3492 | 0.9462 | 0.0283 | 1.0965 | 0.6891 | 1.2589 | 0.4131 | 2.5351 | 0.0703 | 0.8318 | 0.0015 | 1.7219 | 0.3758 | 0.7244 | 0.0001 | 1.6749 | 0.4699 |
| 963 | Q14318 | Peptidyl-prolyl cis-trans isomerase FKBP8 (PPIase FKBP8) (EC 5.2.1.8) (38 kDa FK506-binding protein) (38 kDa FKBP) (FKBP-38) (hFKBP38) (FK506-binding protein 8) (FKBP-8) (FKBPR38) (Rotamase) | FKBP8 FKBP38 | 83 | 3 | 0.2535 | 0.0000 | 1.2134 | 0.6781 | 2.0512 | 0.0000 | 0.8318 | 0.5002 | 0.7379 | 0.0598 | 0.5970 | 0.5886 | 0.7727 | 0.0035 | 3.6983 | 0.1082 | 1.2134 | 0.2151 | 1.7378 | 0.3400 | 2.1478 | 0.0000 | 1.8197 | 0.3206 | 0.9727 | 0.3865 | 0.9036 | 0.8706 |
| 964 | Q13451 | Peptidyl-prolyl cis-trans isomerase FKBP5 (PPIase FKBP5) (EC 5.2.1.8) (51 kDa FK506-binding protein) (51 kDa FKBP) (FKBP-51) (54 kDa progesterone receptor-associated immunophilin) (Androgen-regulated protein 6) (FF1 antigen) (FK506-binding protein 5) (FKBP-5) (FKBP54) (p54) (HSP90-binding immunophilin) (Rotamase) | FKBP5 AIG6 FKBP51 | 6 | 4 | 1.4588 | 0.1376 | 0.9290 | 0.7074 | 0.8395 | 0.8070 | 1.1588 | 0.5128 | 1.4723 | 0.1100 | 2.0701 | 0.1739 | 1.1588 | 0.3826 | 1.4060 | 0.6183 | 1.1695 | 0.4072 | 1.0375 | 0.8092 | 0.6081 | 0.1532 | 1.0471 | 0.7184 | 0.9290 | 0.8167 | 0.8166 | 0.7953 |
| 965 | Q02790 | Peptidyl-prolyl cis-trans isomerase FKBP4 (PPIase FKBP4) (EC 5.2.1.8) (51 kDa FK506-binding protein) (FKBP51) (52 kDa FK506-binding protein) (52 kDa FKBP) (FKBP-52) (59 kDa immunophilin) (p59) (FK506-binding protein 4) (FKBP-4) (FKBP59) (HSP-binding immunophilin) (HBI) (Immunophilin FKBP52) (Rotamase) [Cleaved into: Peptidyl-prolyl cis-trans isomerase FKBP4, N-terminally processed] | FKBP4 FKBP52 | 6 | 7 | 0.9817 | 0.9262 | 0.6427 | 0.4722 | 0.8710 | 0.3922 | 0.6026 | 0.2352 | 0.8630 | 0.5693 | 1.1803 | 0.3565 | 0.7586 | 0.4006 | 0.4742 | 0.1165 | 0.8017 | 0.1637 | 0.7244 | 0.5053 | 1.0093 | 0.9472 | 0.7798 | 0.3197 | 1.0965 | 0.7146 | 0.6607 | 0.3791 |
[truncated: 302,037 more chars]
